# Supplementary material for: 2019 HRS/EHRA/APHRS/LAHRS expert consensus statement on catheter ablation of ventricular arrhythmias
Source: Heart Rhythm. Author manuscript; Available in PMC 2021 Sep 21. (PMC8453449; doi:10.1016/j.hrthm.2019.03.002)
Supplement: Supplementary material Appendix 3 [file NIHMS1737000-supplement-Supplementary_material_Appendix_3.pdf]

Appendix 3 Evidence table

Diagnostic Evaluation

Study name or acronym; Author; Year published; PMID	Aim of study; Endpoints; Study type (RCT, observational — multicenter or single center; case series or other [if RCT, include intervention and comparator]); Study size (N)	Patient population with inclusion and exclusion criteria	Results (absolute event rates, *P* values; OR or RR; 95% CI)	Other relevant findings or adverse events	Limitations; Other comments; Conclusions
**12-Lead ECG**					
Brugada et al. A new approach to the differential diagnosis of a regular tachycardia with a wide QRS complex.<br>• Year published: 1991<br>• PMID: 2022022	Aim: To incorporate new and simpler ECG criteria for differential diagnosis of WCT.<br>Study type: Prospective<br>Size: 554	Inclusion criteria: WCT with EPS-proven tachycardia mechanism<br><br>Exclusion criteria: Treatment with AAD	New stepwise approach had sensitivity 0.987 and specificity 0.965.		These new criteria incorporated in a stepwise approach could help prevent the frequent errors made in the differential diagnosis of tachycardias with a wide QRS complex.
Wellens et al. The value of the electrocardiogram in the differential diagnosis of a tachycardia with a widened QRS complex.<br>• Year published: 1978<br>• PMID: 623134	Aim: To determine the value of the electrocardiogram for differentiating aberrant conduction from ventricular ectopy.<br>Study type: Retrospective<br>Size: 122	Inclusion criteria: WCT with EPS-proven tachycardia mechanism.<br><br>Exclusion criteria: Treatment with AAD	QRS width >0.14 sec, left axis deviation, QRS morphology in V1 & V6 and AV dissociation suggested VT.		The observations mentioned on QRS width, configuration, and axis are helpful in differentiating between a supraventricular and a ventricular origin of the tachycardia.
Verekei et al. New algorithm using only lead aVR for differential diagnosis of wide QRS complex tachycardia.<br>• Year published: 2008<br>• PMID: 18180024	Aim: To simplify the WCT diagnostic algorithm by omitting the complicated morphologic criteria and restricting the analysis to lead aVR.<br>Study type: Prospective<br>Size: 483	Inclusion criteria: Regular WCT with EPS-proven tachycardia mechanism	New aVR algorithm: Sensitivity 95.6; Specificity 75; PPV 92.7; NPV 86.6		The new aVR algorithm proved to be a reasonably rapid, easy, and accurate means for obtaining the correct diagnosis in the differential diagnosis of wide QRS complex tachycardias.

Study name or acronym; Author; Year published; PMID	Aim of study; Endpoints; Study type (RCT, observational — multicenter or single center; case series or other [if RCT, include intervention and comparator]); Study size (N)	Patient population with inclusion and exclusion criteria	Results (absolute event rates, *P* values; OR or RR; 95% CI)	Other relevant findings or adverse events	Limitations; Other comments; Conclusions
Berruezo et al. Electrocardiographic recognition of the epicardial origin of ventricular tachycardias.<br>• Year published: 2004<br>• PMID: 15078793	Aim: To define the ECG characteristics that can identify an epicardial VT origin.<br>Study type: Retrospective<br>Size: 69	Inclusion criteria: VTs with RBBB morphology ablated from endocardium or epicardium	A pseudodelta wave of ≥34 ms has a sensitivity of 83% and a specificity of 95%; an intrinsicoid deflection time of ≥85 ms, has a sensitivity of 87% and a specificity of 90%; and an RS complex duration of ≥121 ms has a sensitivity of 76% and a specificity of 85% in identifying an epicardial VT origin.		Analysis of the conventional ECG suggests VTs originating from the epicardium and those with an unsuccessful ablation from the endocardium, with a high sensitivity and specificity.
Daniels et al. Idiopathic epicardial left ventricular tachycardia originating remote from the sinus of Valsalva: electrophysiological characteristics, catheter ablation, and identification from the 12-lead electrocardiogram.<br>• Year published: 2006<br>• PMID: 16567566	Aim: To define the electrophysiological features, anatomic substrate, and approach to catheter ablation of epicardial idiopathic LV tachycardia not originating adjacent to the ASOV.<br>Study type: Retrospective<br>Size: 12	Inclusion criteria: Idiopathic VT with an epicardial LV site of origin identified >10 mm from the ASOV	A delayed precordial maximum deflection index ≥0.55 identified epicardial VT remote from the ASOV with a sensitivity of 100% and a specificity of 98.7%.		Recognition of a prolonged precordial maximum deflection index and early use of transvenous epicardial mapping are critical to prevent protracted and unsuccessful ablation elsewhere in the ventricles.

Study name or acronym; Author; Year published; PMID	Aim of study; Endpoints; Study type (RCT, observational — multicenter or single center; case series or other [if RCT, include intervention and comparator]); Study size (N)	Patient population with inclusion and exclusion criteria	Results (absolute event rates, *P* values; OR or RR; 95% CI)	Other relevant findings or adverse events	Limitations; Other comments; Conclusions
Bazan et al. Site-specific twelve-lead ECG features to identify an epicardial origin for left ventricular tachycardia in the absence of myocardial infarction.<br>• Year published: 2007<br>• PMID: 17954399	Aim: To assess whether previously reported ECG criteria to distinguish an epicardial LV-VT origin apply uniformly to all LV regions and in the absence of MI.<br>Study type: Prospective<br>Size: 15	Inclusion criteria: Patients undergoing endocardial and epicardial catheter mapping and ablation for drug refractory ventricular arrhythmias	Basal and apical superior pace mapping sites showed a Q wave in lead I more commonly from epicardial vs corresponding endocardial sites (90% vs 16%, 88% vs 26%, respectively; *P*<.001. The absence of a Q wave in leads II-III-aVF identified epicardial basal superior sites, *P*=.002. Basal and apical inferior epicardial sites showed a Q wave in leads II-III-aVF (81% vs 37%, 92% vs 33%; *P*<.001).	The QRS duration was longer from the epicardium, 213 ± 45 ms vs 191 ± 41 ms, *P*<.001.	ECG features distinguishing epicardial LV-VT are site specific, including the presence or absence of a Q wave in leads that reflect local ventricular activation.
Vallès et al. ECG criteria to identify epicardial ventricular tachycardia in nonischemic cardiomyopathy.<br>• Year published: 2010<br>• PMID: 20008307	Aim: To assess the value of published interval and morphological criteria for identifying an epicardial origin and to determine whether a more effective algorithm using modified criteria for identifying an epicardial origin in this setting could be established.<br>Study type: Prospective<br>Size: 14	Inclusion criteria: Patients with NICM undergoing endocardial and epicardial catheter mapping and ablation for drug-refractory VAs	A Q wave in lead I was observed in epicardial but not endocardial pace maps (91% vs 4%; *P*<.001), identified 14 of 16 epicardial VTs (sensitivity, 88%), and was observed in 1 of 8 endocardial VTs (specificity, 88%).	The 4-step algorithm identified the origin in 109 of 115 pace maps (95%), in 21 of 24 VTs (88%) in the study population, and in 19 of 21 VTs (90%) in the validation cohort.	Morphological ECG features that describe the initial QRS vector can help identify basal-superior/lateral epicardial VTs in nonischemic cardiomyopathy.

Study name or acronym; Author; Year published; PMID	Aim of study; Endpoints; Study type (RCT, observational — multicenter or single center; case series or other [if RCT, include intervention and comparator]); Study size (N)	Patient population with inclusion and exclusion criteria	Results (absolute event rates, *P* values; OR or RR; 95% CI)	Other relevant findings or adverse events	Limitations; Other comments; Conclusions
Bazan et al. Twelve-lead ECG features to identify ventricular tachycardia arising from the epicardial right ventricle.<br>• Year published: 2006<br>• PMID: 17018339	Aim: To investigate the hypothesis that specific ECG features identify an epicardial origin for RV VT.<br>Study type: Prospective<br>Size: 13	Inclusion criteria: Patients undergoing endocardial and epicardial catheter mapping and ablation for drug-refractory VAs	A Q wave in lead II, III, or aVF was more likely noted from inferior epicardial vs endocardial sites (53 of 73 vs 16 of 43; *P*<.01). A Q wave in lead I was more frequently present from epicardial vs endocardial anterior RV sites (30 of 82 vs 5 of 52; *P*<.001).	Reported cutoff values for identifying epicardial LV origin did not apply to the RV. In the RV outflow tract, no ECG feature distinguishing epicardial/endocardial origin reached statistical significance.	A Q wave or QS in leads that best reflect local activation suggest an epicardial origin for RV depolarization and could help in identifying a probable epicardial site of origin for RV VT.
Pérez-Rodon et al. Prognostic value of the electrocardiogram in patients with syncope: data from the group for syncope study in the emergency room (GESINUR).<br>• Year published: 2014<br>• PMID: 24993462	Aim: To study the association between specific ECG abnormalities and mortality in patients with syncope from the GESINUR study.<br>Endpoints: All-cause mortality<br>Study type: Observational retrospective<br>Size: 344	Inclusion criteria: Patients with syncope and available ECG and 12-month follow-up data	Some 6.3% of patients died. AF (OR 6.8; 95% CI 2.8–16.3; *P*<.001), intraventricular conduction disturbances (OR 3.8; 95% CI 1.7–8.3; *P*=.001), LVH ECG criteria (OR 6.3; 95% CI 1.5–26.3; *P*=.011), and ventricular pacing (OR 21.8; 95% CI 4.1–115.3; *P*<.001) were the only independent ECG predictors of all-cause mortality.		Although an abnormal ECG in patients with syncope is a common finding, only the presence of AF, intraventricular conduction delay, LVH ECG criteria, and ventricular pacing are associated with 1-year all-cause mortality.

Study name or acronym; Author; Year published; PMID	Aim of study; Endpoints; Study type (RCT, observational — multicenter or single center; case series or other [if RCT, include intervention and comparator]); Study size (N)	Patient population with inclusion and exclusion criteria	Results (absolute event rates, *P* values; OR or RR; 95% CI)	Other relevant findings or adverse events	Limitations; Other comments; Conclusions
Ohe et al. Idiopathic sustained left ventricular tachycardia: clinical and electrophysiologic characteristics.<br>• Year published: 1988<br>• PMID: 3342487	Aim: To investigate the origin, electrophysiologic characteristics of VT, the efficacy of AAD therapy and long-term follow-up.<br>Study type: Observational prospective<br>Size: 16	Inclusion criteria: Consecutive patients with sustained VT from LV without overt structural heart disease	The earliest site of activation was at the apical inferior portion of the left ventricle in 14 patients whose QRS morphology during VT showed a RBBB pattern and left-axis deviation, but at the apical anterosuperior portion of the left ventricle in two patients whose QRS morphology during VT showed an RBBB and right-axis deviation.		(1) Idiopathic LV tachycardia has unique electrocardiographic, electrophysiologic, and electropharmacological properties; (2) the electrophysiologic characteristics suggest that the mechanism is reentry; and (3) verapamil is effective in both the short- and long-term treatment of VT.
Dixit et al. Electrocardiographic patterns of superior right ventricular outflow tract tachycardias: distinguishing septal and free-wall sites of origin.<br>• Year published: 2003<br>• PMID: 12625602	Aim: To analyze ECG patterns of superior RV outflow tract tachycardias.<br>Study type: Observational prospective<br>Size: 14	Inclusion criteria: Patients with structurally normal hearts undergoing ablation for outflow tract VT	Monophasic R waves in inferior leads for septal sites were taller and narrower compared with free-wall sites, lacked "notching," and showed early precordial transition. A positive R wave in lead I also distinguished posterior from anterior septal and free-wall sites.		Despite overlap in QRS amplitude and duration, in the majority of patients a combination of ECG features can serve as a useful template in accurately predicting the site of origin of clinical arrhythmias arising from this region.

Study name or acronym; Author; Year published; PMID	Aim of study; Endpoints; Study type (RCT, observational — multicenter or single center; case series or other [if RCT, include intervention and comparator]); Study size (N)	Patient population with inclusion and exclusion criteria	Results (absolute event rates, *P* values; OR or RR; 95% CI)	Other relevant findings or adverse events	Limitations; Other comments; Conclusions
Callans et al. Repetitive monomorphic tachycardia from the left ventricular outflow tract: electrocardiographic patterns consistent with a left ventricular site of origin.<br>• Year published: 1997<br>• PMID: 9120154	Aim: To assess the incidence of LV sites of origin in patients with clinical repetitive monomorphic VT and to identify ECG patterns consistent with an LV origin.<br>Study type: Observational prospective<br>Size: 33	Inclusion criteria: Patients with sinus rhythm on ECG and normal echocardiographic findings presenting with monomorphic VT	Pace maps identical in configuration to the induced tachycardia were obtained from the RVOT in 29 of 33 patients. In 4 (12%) patients, pace maps obtained from the RVOT did not match the induced tachycardia. All 4 patients had a QRS configuration during repetitive monomorphic VT with precordial R wave transitions at or before lead V2.		Repetitive monomorphic VT can arise from the outflow tract of both the right and left ventricles. Repetitive monomorphic VTs with a precordial R wave transition at or before lead V2 are consistent with an LV origin.
Kanagaratnam et al. Ventricular tachycardias arising from the aortic sinus of Valsalva: an under-recognized variant of left outflow tract ventricular tachycardia.<br>• Year published: 2001<br>• PMID: 11300454	Aim: To describe a normal heart LBBB inferior axis VT that could not be ablated from the right or left ventricular outflow tracts.<br>Study type: Observational retrospective<br>Size: 12	Inclusion criteria: Patients with normal heart LBBB, inferior axis VT, and previously failed ablation	All patients were successfully ablated from the ASOVs. The electrocardiographic pattern associated with this VT was LBBB, inferior axis and early precordial transition with Rs or R in V2 or V3. VT from the left sinus had an rS pattern in lead I, and VT from the noncoronary sinus had a notched R wave in lead I. None of the patients had complications, and all remained arrhythmia-free at a mean follow-up of 8 months.		Normal heart VT with LBBB, inferior axis, and early precordial transition can be ablated in the majority of patients from either the left or the noncoronary ASOV.

Study name or acronym; Author; Year published; PMID	Aim of study; Endpoints; Study type (RCT, observational — multicenter or single center; case series or other [if RCT, include intervention and comparator]); Study size (N)	Patient population with inclusion and exclusion criteria	Results (absolute event rates, *P* values; OR or RR; 95% CI)	Other relevant findings or adverse events	Limitations; Other comments; Conclusions
Crawford et al. Ventricular arrhythmias originating from papillary muscles in the right ventricle.<br>• Year published: 2010<br>• PMID: 20206325	Aim: To describe a series of 8 patients in whom VAs were mapped to one of the right-sided papillary muscles.<br>Study type: Observational retrospective<br>Size: 8	Inclusion criteria: Patients with frequent PVCs or both PVCs and VT who had been referred for catheter ablation and whose arrhythmia was mapped to one of the RV papillary muscles	The QRS complex was broader in the RV PAP group compared with the control group. RV PAP arrhythmias originating from the posterior or anterior RV PAPs more often had a superior axis with late R-wave transition compared with septal RV RAP arrhythmias, which more often had an inferior axis with an earlier R-wave transition in the precordial leads.		PVCs and VT can originate in the RV PAPs. RF ablation is effective in eliminating these arrhythmias.
Yamada et al. Idiopathic focal ventricular arrhythmias originating from the anterior papillary muscle in the left ventricle.<br>• Year published: 2009<br>• PMID: 19298560	Aim: To describe a distinct subgroup of idiopathic VAs that arise from the APM in the left ventricle.<br>Study type: Observational retrospective<br>Size: 6	Inclusion criteria: Patients with VT or PVC with the earliest site of ventricular activation localized to the LV APM	No Purkinje potentials were recorded at the ablation site during sinus rhythm or during the VAs. The VAs exhibited an RBBB and right inferior axis QRS morphology in all patients. VT was not inducible by programmed electrical stimulation in any of the patients.		VAs may arise from the base or middle portion of the APM and are characterized by an RBBB, right inferior access QRS morphology, and a focal mechanism. Catheter ablation of APM VAs is typically challenging, and creation of a deep radiofrequency lesion might be necessary for long-term success.

Study name or acronym; Author; Year published; PMID	Aim of study; Endpoints; Study type (RCT, observational — multicenter or single center; case series or other [if RCT, include intervention and comparator]); Study size (N)	Patient population with inclusion and exclusion criteria	Results (absolute event rates, *P* values; OR or RR; 95% CI)	Other relevant findings or adverse events	Limitations; Other comments; Conclusions
Li et al. Surface electrocardiography characteristics and radiofrequency catheter ablation of idiopathic ventricular arrhythmias originating from the left infero-septal papillary muscles: differences from those originating from the left posterior fascicle.<br>• Year published: 2018<br>• PMID: 28449078	Aim: To report the ECG characteristics and RF catheter ablation of LV infero-septal papillary muscles and LPF VAs.<br>Study type: Observational retrospective<br>Size: 127	Inclusion criteria: Patients undergoing catheter ablation of idiopathic VAs originating from the LPF and papillary muscles	Papillary muscle VAs had a longer QRS duration than LPF VAs. All 7 VAs with QRS duration >160 ms originated from the papillary muscles, whereas all 87 VAs with QRS duration <130 ms arose from the LPF. In 33 VAs with QRS 130–160 ms, all 13 with Vi/Vt ≤0.85 originated from the papillary muscles, and in 19 of 20 with Vi/Vt >0.85 arose from the LPF.		Papillary muscle VAs could be identified from LPF VAs by calculation of QRS duration combined with Vi/Vt using ECG.
**Assessment of Structural Heart Disease and Myocardial Ischemia**					
Solomon et al. Sudden death in patients with myocardial infarction and left ventricular dysfunction, heart failure, or both.<br>• Year published: 2005<br>• PMID: 15972864	Aim: To assess the risk and time course of sudden death in high-risk patients after MI.<br>Endpoints: Sudden unexpected death or cardiac arrest with resuscitation.<br>Study type: Substudy of randomized clinical trial (VALIANT).<br>Size: 14609	Inclusion criteria: Acute MI with heart failure and LV dysfunction<br><br>Exclusion criteria: Intolerance to study drugs; significant valvular disease	Patients with an LVEF of 30% or less were at highest risk of sudden death in this early period post-MI (2.3% per month; 95% CI 1.8%–2.8%). Each decrease of 5 percentage points in LVEF was associated with a 21% adjusted increase in the risk of sudden death in the first 30 days post-MI.		The risk of sudden death is highest in the first 30 days after MI among patients with LV dysfunction, heart failure, or both.

Study name or acronym; Author; Year published; PMID	Aim of study; Endpoints; Study type (RCT, observational — multicenter or single center; case series or other [if RCT, include intervention and comparator]); Study size (N)	Patient population with inclusion and exclusion criteria	Results (absolute event rates, *P* values; OR or RR; 95% CI)	Other relevant findings or adverse events	Limitations; Other comments; Conclusions
Gula et al. Ejection fraction assessment and survival: an analysis of the Sudden Cardiac Death in Heart Failure Trial (SCD-HeFT).<br>• Year published: 2008<br>• PMID: 19033019	Aim: To examine whether the modality of assessing EF affected the likelihood of survival.<br>Endpoints: All-cause mortality<br>Study type: Substudy of randomized clinical trial (SCD-HeFT).<br>Size: 2521	Inclusion criteria:<br>Patients with NYHA class II or III congestive heart failure and LVEF ≤35%	No significant difference in survival between patients enrolled based on radionuclide study versus echocardiography (HR 1.06; 95% CI 0.88–1.28), radionuclide study versus angiography (HR 1.25; 95% CI 0.97–1.62), or echocardiography versus angiography (HR 1.18; 95% CI 0.94–1.48).		Among patients enrolled in SCD-HeFT, the distribution of EFs measured by radionuclide angiography differed from those measured by echocardiography or contrast angiograms. Survival did not differ according to modality of EF assessment.
Yoon et al. Prognostic value of unrecognised myocardial infarction detected by late gadolinium-enhanced MRI in diabetic patients with normal global and regional left ventricular systolic function.<br>• Year published: 2013<br>• PMID: 23553584	Aim: To determine whether the detection of unrecognized MI using LGE-MRI can provide prognostic information in patients with diabetes.<br>Endpoints: MACE<br>Study type: Retrospective<br>Size: 128	Inclusion criteria:<br>Patients with type 2 diabetes who underwent resting 12-lead ECG, cine- and LGE-MRI<br><br>Exclusion criteria:<br>Ischemic ECG changes and abnormal cine MRI findings	Six patients with LGE (33.3%) and 4 patients without LGE (3.9%) experienced MACE, resulting in an annualized event rate of 7.7% and 0.9%, respectively (log-rank *P* <.001). The presence of LGE was associated with an 8-fold increased hazard for MACE (HR, 8.84; *P*=.001).		LGE-MRI can detect unrecognized MI and could improve the risk stratification of patients with diabetes with no coronary artery disease history, normal ECG, and normal LV systolic function.

Study name or acronym; Author; Year published; PMID	Aim of study; Endpoints; Study type (RCT, observational — multicenter or single center; case series or other [if RCT, include intervention and comparator]); Study size (N)	Patient population with inclusion and exclusion criteria	Results (absolute event rates, *P* values; OR or RR; 95% CI)	Other relevant findings or adverse events	Limitations; Other comments; Conclusions
Olivotto et al. Assessment and significance of left ventricular mass by cardiovascular magnetic resonance in hypertrophic cardiomyopathy.<br>• Year published: 2008<br>• PMID: 18687251	Aim: To assess the distribution and clinical significance of LV mass in patients with HCM.<br>Study type: Prospective<br>Size: 264	Inclusion criteria: Patients with HCM referred for CMR	The LV mass index in patients with HCM significantly exceeded that of the control subjects. However, values were within the normal range in 21% and only mildly increased in 16%. The LV mass index showed a modest relationship to maximal LV thickness.		LV mass index was normal in approximately 20% of patients with a definite HCM phenotype. Therefore, increased LV mass is not a requirement for establishing the clinical diagnosis of HCM.
Desjardins et al. Characteristics of intramural scar in patients with nonischemic cardiomyopathy and relation to intramural ventricular arrhythmias.<br>• Year published: 2013<br>• PMID: 23985383	Aim: To assess the value of voltage mapping to detect MRI-defined intramural scar and to correlate the scar with ventricular arrhythmias.<br>Study type: Prospective<br>Size: 15	Inclusion criteria: Patients with NICM referred for RF catheter ablation of symptomatic VAs in whom a predominant intramural scar was detected on delayed-enhancement MRI	Using ROC curves, a unipolar cutoff value of 6.78 mV (AUC 0.78) and a bipolar cutoff value of 1.55 mV (AUC 0.69) better separated endocardial measurements overlying a scar compared with areas not overlying a scar.		Intramural scar can be detected by unipolar and bipolar voltage, unipolar voltage being more useful.

Study name or acronym; Author; Year published; PMID	Aim of study; Endpoints; Study type (RCT, observational — multicenter or single center; case series or other [if RCT, include intervention and comparator]); Study size (N)	Patient population with inclusion and exclusion criteria	Results (absolute event rates, *P* values; OR or RR; 95% CI)	Other relevant findings or adverse events	Limitations; Other comments; Conclusions
Dweck et al. Hybrid magnetic resonance imaging and positron emission tomography with fluorodeoxyglucose to diagnose active cardiac sarcoidosis.<br>• Year published: 2018<br>• PMID: 28624396	Aim: To explore the diagnostic usefulness of hybrid CMR and PET using FDG for active cardiac sarcoidosis.<br>Study type: Prospective<br>Size: 25	Inclusion criteria: Patients with clinical suspicion of active cardiac sarcoidosis due to established extracardiac involvement and/or clinical presentation suggestive of the disease<br><br>Exclusion criteria: Insulin-dependent diabetes, blood glucose >200 mmol/dL before scanning, claustrophobia, pregnancy/nursing, presence of pacemaker or automatic ICD, and impaired renal function	Eight patients were MRI+PET+, suggestive of active cardiac sarcoidosis; 1 was MRI+PET-, consistent with inactive cardiac sarcoidosis; and 8 were MRI-PET-, with no imaging evidence of cardiac sarcoidosis. Eight patients were MRI-PET+.	Maximum target-to-normal myocardium ratio values were higher in the active cardiac sarcoidosis+ group (*P*<.001), demonstrating an area under the curve of 0.98 on receiver operating characteristic analysis for the detection of active cardiac sarcoidosis, with an optimal maximum target-to-normal myocardium ratio threshold of 1.2 (Youden index: 0.94).	CMR/PET imaging holds major promise for the diagnosis of active cardiac sarcoidosis.

Study name or acronym; Author; Year published; PMID	Aim of study; Endpoints; Study type (RCT, observational — multicenter or single center; case series or other [if RCT, include intervention and comparator]); Study size (N)	Patient population with inclusion and exclusion criteria	Results (absolute event rates, *P* values; OR or RR; 95% CI)	Other relevant findings or adverse events	Limitations; Other comments; Conclusions
Piers et al. Contrast-enhanced MRI-derived scar patterns and associated ventricular tachycardias in nonischemic cardiomyopathy: implications for the ablation strategy.<br>• Year published: 2013<br>• PMID: 24036134	Aim: (1) To identify typical MRI-derived scar patterns and the associated 12-lead VT morphologies in consecutive patients with NICM who underwent contrast-enhanced MRI and VT ablation; (2) to evaluate its implications for the ablation strategy; and (3) to analyze the value of bipolar and unipolar endocardial voltage mapping to detect the contrast-enhanced MRI-derived VT substrate in patients with NICM.<br>Study type: Observational<br>Size: 19	Inclusion criteria: Patients with NICM who underwent contrast-enhanced MRI and VT ablation	On the basis of 3D contrast-enhanced MRI-derived scar reconstructions, 8 (42%) patients had predominant basal anteroseptal scar, 9 (47%) patients had predominant inferolateral scar, and 2 (11%) patients had other scar types.		Basal anteroseptal and inferolateral scars account for 89% of arrhythmogenic substrates in patients with NICM.

Study name or acronym; Author; Year published; PMID	Aim of study; Endpoints; Study type (RCT, observational — multicenter or single center; case series or other [if RCT, include intervention and comparator]); Study size (N)	Patient population with inclusion and exclusion criteria	Results (absolute event rates, *P* values; OR or RR; 95% CI)	Other relevant findings or adverse events	Limitations; Other comments; Conclusions
Brugada et al. Coronary artery revascularization in patients with sustained ventricular arrhythmias in the chronic phase of a myocardial infarction: effects on the electrophysiologic substrate and outcome.<br>• Year published: 2001<br>• PMID: 11216974	Aim: To analyze the influence of coronary artery revascularization in patients with VAs.<br>Study type: Prospective<br>Size: 64	Inclusion criteria: Patients presenting with sustained VAs in the chronic phase of an MI and in whom coronary artery revascularization was indicated after clinical and angiographic evaluation<br><br>Exclusion criteria: Patients in whom clinical, electrocardiographic, or enzymatic data suggested an acute ischemic event as a trigger for the arrhythmia	After revascularization, in 62 survivors, 52 of 59 patients previously inducible were still inducible (group A), and 10 patients were noninducible (group B). During follow-up, 54% of the patients in group A and 40% of the patients in group B had arrhythmic events (*P*=.46). An EF <30% predicted recurrent arrhythmic events (*P*=.02), but not the presence of demonstrable ischemia before revascularization (*P*=.42).	Amiodarone or beta-adrenergic blocking agent therapy did not predict recurrent arrhythmic events.	In patients with VAs in the chronic phase of MI, probability of recurrence is high despite coronary artery revascularization; however, mortality is low if combined with appropriate antiarrhythmic therapy. Recurrences are related to the presence of a low EF but not to demonstrable ischemia before revascularization, amiodarone, or beta-blocker therapy, nor are they the result of electrophysiological testing after revascularization.

Study name or acronym; Author; Year published; PMID	Aim of study; Endpoints; Study type (RCT, observational — multicenter or single center; case series or other [if RCT, include intervention and comparator]); Study size (N)	Patient population with inclusion and exclusion criteria	Results (absolute event rates, *P* values; OR or RR; 95% CI)	Other relevant findings or adverse events	Limitations; Other comments; Conclusions
Nageh et al. Implantable defibrillators for secondary prevention of sudden cardiac death in cardiac surgery patients with perioperative ventricular arrhythmias.<br>• Year published: 2014<br>• PMID: 25146702	Aim: To study the potential role of ICDs in patients with VAs <3 months postrevascularization, compare the outcomes between the groups with pre- versus postoperative arrhythmias, as well as assess the value of clinical markers such as degree of revascularization in predicting the patients at higher risk for the outcomes of total mortality and appropriate ICD therapy.<br>Endpoints: The primary end point was total mortality and/or appropriate ICD therapy, and secondary end points are total mortality and ICD therapy.<br>Study type: Retrospective<br>Size: 164	Inclusion criteria: Patients admitted with VT/VF, who had CABG (alone or combined with valve surgery) during that admission, and who had an ICD within 3 months of cardiac surgery, as well as all post-CABG patients (alone or combined with valve surgery) who developed VT/VF postoperatively and who had an ICD within 3 months of cardiac surgery<br><br>Exclusion criteria: Patients in whom a reversible cause was identified, such as electrolyte imbalance or medications	During the mean follow-up of 49 months, the primary endpoint of TM and ICD therapy and individual end points of TM and ICD therapy were observed in 52 (56%), 35 (38%), and 28 (30%) patients, respectively, with 55% of TM, and 23% of ICD therapy occurring within 2 years of implant. In a multivariate risk analysis, none of the following was associated with any of the end points: incomplete revascularization, presenting VA, and timing of arrhythmias.		The data support the recent guidelines for ICD in this cohort of patients, given the presence of irreversible substrate and triggers of VAs cannot be reliably excluded even with complete revascularization.

Study name or acronym; Author; Year published; PMID	Aim of study; Endpoints; Study type (RCT, observational — multicenter or single center; case series or other [if RCT, include intervention and comparator]); Study size (N)	Patient population with inclusion and exclusion criteria	Results (absolute event rates, *P* values; OR or RR; 95% CI)	Other relevant findings or adverse events	Limitations; Other comments; Conclusions
Elsokkari et al. Effect of coronary revascularization on long-term clinical outcomes in patients with ischemic cardiomyopathy and recurrent ventricular arrhythmia.<br>• Year published: 2018<br>• PMID: 29750365	Aim: To examine the impact of prior coronary revascularization on clinical outcomes in patients with ICM and VT.<br>Endpoints: The primary outcome was a composite of death, appropriate ICD shock, or VT storm. The secondary outcomes included elements of the primary outcome, hospitalization, and any VA.<br>Study type: Substudy of randomized clinical trial (VANISH).<br>Size: 259	Inclusion criteria: Patients with ICM and an ICD who had VT despite the use of AADs	There was a trend toward fewer hospitalizations in the revascularization group (64% vs 77%; *P*=.07); there were no differences in the individual outcomes of mortality, VT storm, ICD shocks, recurrent MI, or cardiac failure.		In this cohort of patients with an ischemic cause for VT, a history of prior coronary revascularization was not associated with a reduction in VA or mortality.
**Risk Stratification**					
Scott et al. Late gadolinium enhancement cardiac magnetic resonance imaging for the prediction of ventricular tachyarrhythmic events: a meta-analysis.<br>• Year published: 2013<br>• PMID: 23558217	Aim: To better gauge the predictive accuracy and therefore the potential clinical utility of LGE-CMR for SCD risk stratification.<br>Endpoints: VA events (SCD, resuscitated cardiac arrest, the occurrence of VAs, or appropriate ICD therapy)<br>Study type: Meta-analysis<br>Size: 1105	Inclusion criteria: Studies evaluating the association between the extent of LV scar on LGE-CMR and VA events<br><br>Exclusion criteria: Studies that used a nonarrhythmic endpoint	VA events were more common in patients with a greater extent of LV scar: RR 4.33 (95% CI 2.98–6.29), positive LR 1.98 (95% CI 1.66–2.37), and negative LR 0.33 (95% CI 0.24–0.46).		The extent of LGE on CMR is strongly associated with the occurrence of VAs in patients with reduced LVEF.

Study name or acronym; Author; Year published; PMID	Aim of study; Endpoints; Study type (RCT, observational — multicenter or single center; case series or other [if RCT, include intervention and comparator]); Study size (N)	Patient population with inclusion and exclusion criteria	Results (absolute event rates, *P* values; OR or RR; 95% CI)	Other relevant findings or adverse events	Limitations; Other comments; Conclusions
Kuruvilla et al. Late gadolinium enhancement on cardiac magnetic resonance predicts adverse cardiovascular outcomes in nonischemic cardiomyopathy: a systematic review and meta-analysis.<br>• Year published: 2014<br>• PMID: 24363358	Aim: To evaluate the prognostic role of LGE-CMR imaging in patients with NICM.<br>Endpoints: All-cause mortality, HFH, and a composite end point of SCD or aborted SCD.<br>Study type: Meta-analysis<br>Size: 1488	Inclusion criteria: Studies that evaluated myocardial fibrosis in patients with NICM using LGE-CMR that included hard end points, such as all-cause mortality, SCD or aborted SCD, and HFH<br><br>Exclusion criteria: Studies that evaluated ischemic cardiomyopathies, acute myocarditis, and hypertrophic and infiltrative cardiomyopathies	Patients with LGE had increased overall mortality (OR 3.27; 95% CI 1.94–5.51; *P*<.00001), HFH (OR 2.91; 95% CI 1.16–7.27; *P*=.02), and SCD/aborted SCD (OR 5.32; 95% CI 3.45–8.20; *P*<.00001) compared with those without LGE.		LGE in patients with NICM is associated with increased risk of all-cause mortality, HFH, and SCD.
Di Marco et al. Late gadolinium enhancement and the risk for ventricular arrhythmias or sudden death in dilated cardiomyopathy: systematic review and meta-analysis.<br>• Year published: 2017<br>• PMID: 28017348	Aim: To evaluate the association between LGE on CMR imaging and VAs or SCD in patients with DCM.<br>Endpoints: Arrhythmic endpoint (sustained VA, appropriate ICD therapy, or SCD).<br>Study type: Meta-analysis<br>Size: 2948	Inclusion criteria: Prospective and retrospective observational cohort studies that reported the rate of arrhythmic events in adult patients with DCM and that provided information about the presence or absence of LGE<br><br>Exclusion criteria: Studies including patients with CAD	LGE was significantly associated with the arrhythmic endpoint in the overall population (OR 4.3; 95% CI 3.3–5.8; *P*<.001).	The association between LGE and the arrhythmic endpoint remained significant among studies with mean LVEF >35% (OR 5.2; *P*<.001) and was maximal in studies that included only patients with primary prevention ICDs (OR 7.8; *P*=.008).	Across a wide spectrum of patients with DCM, LGE is strongly and independently associated with VA or SCD.

Study name or acronym; Author; Year published; PMID	Aim of study; Endpoints; Study type (RCT, observational — multicenter or single center; case series or other [if RCT, include intervention and comparator]); Study size (N)	Patient population with inclusion and exclusion criteria	Results (absolute event rates, *P* values; OR or RR; 95% CI)	Other relevant findings or adverse events	Limitations; Other comments; Conclusions
Ganesan et al. Impact of late gadolinium enhancement on mortality, sudden death and major adverse cardiovascular events in ischemic and nonischemic cardiomyopathy: A systematic review and meta-analysis.<br>• Year published: 2018<br>• PMID: 29407096	Aim: To evaluate the prognostic importance of LGE in ICM and NICM.<br>Endpoints: All-cause mortality, cardiovascular mortality, and sudden death.<br>Study type: Meta-analysis<br>Size: 7882	Inclusion criteria: Studies in which dichotomized clinical outcome data were reported in patients with ICM or NICM, stratified by either the presence or threshold of LGE-CMR<br><br>Exclusion criteria: Studies exclusively of patients in individual disease-specific subpopulations of NICM (eg, HCM, arrhythmogenic RV cardiomyopathy, sarcoidosis, amyloidosis, myocarditis);<br>review articles, letters to the editor, commentary, conference papers, and case reports	LGE was strongly associated with all-cause mortality (HR 2.96; 95% CI 2.37–3.70; *P* <.001), cardiovascular mortality (HR 3.27; 95% CI 2.05–5.22; *P* <.001), VA and SCD (HR 3.76; 95% CI 3.14–4.52; *P* <.001), and major adverse cardiovascular events (HR 3.24; 95% CI 2.32–4.52; *P* <.001).	In subgroup analyses, LGE was associated with all-cause mortality and cardiovascular mortality in both LVEF ≤35% and LVEF N 35% patients (*P* <.001 all endpoints), as well as in ICM and NICM.	LGE in CMR predicts all-cause mortality, cardiovascular mortality, VA, sudden death, and MACE, independent of LVEF.

Study name or acronym; Author; Year published; PMID	Aim of study; Endpoints; Study type (RCT, observational — multicenter or single center; case series or other [if RCT, include intervention and comparator]); Study size (N)	Patient population with inclusion and exclusion criteria	Results (absolute event rates, *P* values; OR or RR; 95% CI)	Other relevant findings or adverse events	Limitations; Other comments; Conclusions
Becker et al. The prognostic value of late gadolinium-enhanced cardiac magnetic resonance imaging in nonischemic dilated cardiomyopathy: a review and meta-analysis.<br>• Year published: 2018<br>• PMID: 29680351	Aim: To study the additional value of CMR in determining the prognosis in DCM.<br>Endpoints: Cardiovascular mortality, major VA events, rehospitalization for heart failure, and LV reverse remodeling.<br>Study type: Meta-analysis<br>Size: 4554	Inclusion criteria: Studies assessing the predictive value of LGE-CMR in patients with DCM	Patients with LGE had increased cardiovascular mortality (OR 3.40; 95% CI 2.04–5.67), VA events (OR 4.52; 95% CI 3.41–5.99), and rehospitalization for heart failure (OR 2.66; 95% CI 1.67–4.24) compared with those without LGE.	The absence of LGE predicted LV reverse remodeling (OR 0.15; 95% CI 0.06–0.36).	The presence of LGE on CMR substantially worsens prognosis for adverse cardiovascular events in patients with DCM, and its absence indicates LV reverse remodeling.
Disertori et al. Myocardial fibrosis assessment by LGE is a powerful predictor of ventricular tachyarrhythmias in ischemic and nonischemic LV dysfunction: a meta-analysis.<br>• Year published: 2016<br>• PMID: 27450871	Aim: To evaluate the predictive value of LGE-CMR for VT in patients with ICM or NICM patients with ventricular dysfunction.<br>Endpoints: Composite arrhythmic end point of sudden death, aborted sudden death, VT/VF, and appropriate ICD therapy, including ATP.<br>Study type: Meta-analysis<br>Size: 2850	Inclusion criteria: Studies assessing the predictive value of LGE-CMR in the prognostic stratification of ventricular tachyarrhythmias in patients with cardiomyopathy and ventricular dysfunction. Both ischemic and nonischemic etiologies were considered.<br><br>Exclusion criteria: Other cardiomyopathies	The composite arrhythmic endpoint was reached in 23.9% of patients with a positive LGE test (annualized event rate of 8.6%) vs 4.9% of patients with a negative LGE test (annualized event rate of 1.7%; *P*<.0001). The pooled OR was 5.62 (95% CI 4.20–7.51).	No significant differences between patients with ICM or NICM. LGE predicted arrhythmic events even in patients with LVEF >30. The pooled ORs were 9.56 (95% CI 5.63–16.23) in the subgroup of studies with EF ≤30% versus 4.48 (95% CI 3.17–6.33) in the subgroup with EF >30% (*P*=.02).	LGE is a powerful predictor of ventricular arrhythmic risk in patients with ventricular dysfunction, irrespective of ICM and NICM etiology. The prognostic power of LGE is particularly strong in patients with severely depressed EF.

Study name or acronym; Author; Year published; PMID	Aim of study; Endpoints; Study type (RCT, observational — multicenter or single center; case series or other [if RCT, include intervention and comparator]); Study size (N)	Patient population with inclusion and exclusion criteria	Results (absolute event rates, *P* values; OR or RR; 95% CI)	Other relevant findings or adverse events	Limitations; Other comments; Conclusions
Hasdemir et al. Late gadolinium enhancement CMR in patients with tachycardia-induced cardiomyopathy caused by idiopathic ventricular arrhythmias.<br>• Year published: 2012<br>• PMID: 22303908	Aim: To determine the LGE prevalence in patients with tachycardia-induced cardiomyopathy caused by idiopathic VAs.<br>Study type: Prospective<br>Size: 298	Inclusion criteria: Patients having frequent, monomorphic PVCs and/or VTs<br><br>Exclusion criteria: Known structural disease	LGE-CMR was present in 1 of 19 (5%) patients with tachycardia-induced cardiomyopathy and in 4 of 5 (80%) patients with primary cardiomyopathy.		LGE is a rare finding in patients with tachycardia-induced cardiomyopathy caused by idiopathic VAs.
Aquaro et al. Cardiac magnetic resonance predicts outcome in patients with premature ventricular complexes of left bundle branch block morphology.<br>• Year published: 2010<br>• PMID: 20883930	Aim: To evaluate the relationship between RV abnormalities detected by CMR and the clinical end points.<br>Endpoints: Cardiac death, resuscitated cardiac arrest, and appropriate ICD shock.<br>Study type: Prospective<br>Size: 440	Inclusion criteria: Consecutive patients with frequent (>1000) PVCs of LBBB morphology and inferior axis on referring clinical exam of CMR<br><br>Exclusion criteria: Claustrophobia, body dimension greater than the scanner diameter, and frequent PVCs despite AADs during CMR	Patients with multiple RV abnormalities (RVA-2 group) had poorer outcomes than the non-RVA group (*P*<.001). Of the 61 patients in the RVA-2 group, only 6 had a definite diagnosis of ARVD/C according to Task Force Criteria. Also, patients with a single imaging criterion (RVA-1 group) had poorer outcomes than the non-RVA group (*P*=.01).	Patients with only WM abnormalities had higher prevalence of cardiac events than no-RVA (*P*=.03).	In patients with frequent PVC of LBBB morphology, CMR allows risk stratification. RV abnormalities were associated with poorer outcome.

Study name or acronym; Author; Year published; PMID	Aim of study; Endpoints; Study type (RCT, observational — multicenter or single center; case series or other [if RCT, include intervention and comparator]); Study size (N)	Patient population with inclusion and exclusion criteria	Results (absolute event rates, *P* values; OR or RR; 95% CI)	Other relevant findings or adverse events	Limitations; Other comments; Conclusions
Yokokawa et al. Value of cardiac magnetic resonance imaging and programmed ventricular stimulation in patients with frequent premature ventricular complexes undergoing radiofrequency ablation.<br>• Year published: 2017<br>• PMID: 28688990	Aim: To prospectively assess the use of cardiac MRI and programmed ventricular stimulation to identify patients with PVCs undergoing RF ablation at risk for adverse long-term outcomes.<br>Endpoints: VT/VF or death<br>Study type: Retrospective<br>Size: 321	Inclusion criteria: Consecutive patients undergoing PVC ablation, preceded by cardiac MRI to assess for SHD.	SHD was identified by MRI in 64 (20%) patients, and sustained monomorphic VT was inducible in 15 (5%) patients. The combination of SHD by MRI and VT inducibility independently conferred an increased risk of adverse outcome (multivariate HR 25.73; 95% CI 6.74–98.20; *P*<.001.		Preablation cardiac MRI and programmed stimulation can be useful for risk stratification in patients with frequent PVCs. Patients with inducible VT in the setting of SHD might benefit from ICD implantation after ablation regardless of LVEF.
**Longitudinal Follow-up**					
Niwano et al. Prognostic significance of frequent premature ventricular contractions originating from the ventricular outflow tract in patients with normal left ventricular function.<br>• Year published: 2009<br>• PMID: 19429571	Aim: To clarify the prognosis in asymptomatic or less symptomatic patients with frequent PVCs and a normal LV function.<br>Endpoints: VT/VF or death<br>Study type: Prospective, observational<br>Size: 239	Inclusion criteria: Consecutive patients presenting with frequent PVCs (>1000 beats/day) originating from the RV or LV outflow tract without any detectable heart disease<br><br>Exclusion criteria: Patients with syncope or faintness due to nonsustained VT or slight LV dysfunction	There was a significant negative correlation between the PVC prevalence and the change in LVEF (*P*<.001) and positive correlation between the PVC prevalence and the change in LVDd (*P*<.001).		Although the prognosis in patients with frequent PVCs was considered relatively benign, attention should be paid to the progression of the LV dysfunction during a long-term observation, especially in patients with a high PVC prevalence.

Idiopathic Outflow Tract Ventricular Arrhythmias

Study name or acronym; Author; Year published; PMID	Aim of study; Endpoints; Study type (RCT, observational — multicenter or single center; case series or other [if RCT include intervention and comparator]); Study size (N)	Patient population with inclusion and exclusion criteria	Results (absolute event rates, *P* values; OR or RR; 95% CI)	Other relevant findings or adverse events	Limitations; Other comments; Conclusions
Ling et al. Radiofrequency ablation versus antiarrhythmic medication for treatment of ventricular premature beats from the right ventricular outflow tract: prospective randomized study.<br>• Year published: 2014<br>• PMID: 24523413	Study type: Randomized<br>Size: 330<br><br>The purpose of this study was to compare the efficacy of RFCA with AADs for treatment of patients with frequent VPBs originating from the RVOT.	The inclusion criteria were (1) frequent symptomatic VPBs from the RVOT documented by 12-lead ECG to have inferior axis and LBBB QRS morphology; and (2) >6000 VPBs per 24 h on Holter monitoring.<br><br>The exclusion criteria included (1) the presence of non-RVOT origin for VPBs indicated by an S wave in lead I, an R-wave duration index in V1 and V2 ≥0.5, and an RS wave amplitude index in V1 and V2 ≥0.311; (2) previous AAD therapy; (3) evidence of any SHD; (4) hyperthyroidism or electrolyte disturbance; (5) drug toxicity; (6) diabetes mellitus; (7) blood pressure >165/100 mm Hg; (8) significant impairment of renal function; (9) QT interval >450 ms in the absence of bundle-branch block; and (10) significant atrioventricular conduction disease and LBBB or RBBB.	Results: Catheter ablation is more efficacious than AADs for preventing VPB recurrence in patients with frequent VPBs originating from the RVOT. QS morphology in lead I was associated with better outcome after ablation.	AAD	

Study name or acronym; Author; Year published; PMID	Aim of study; Endpoints; Study type (RCT, observational — multicenter or single center; case series or other [if RCT include intervention and comparator]); Study size (N)	Patient population with inclusion and exclusion criteria	Results (absolute event rates, *P* values; OR or RR; 95% CI)	Other relevant findings or adverse events	Limitations; Other comments; Conclusions
Zhang et al. Magnetic versus manual catheter navigation for mapping and ablation of right ventricular outflow tract ventricular arrhythmias: a randomized controlled study.<br>• Year published: 2013<br>• PMID: 23692891	Study type: Randomized<br>Size: 30	Inclusion criteria: Patients with symptomatic RVOT PVC/VT.<br><br>Exclusion criteria: None	Results: Remote magnetic control navigation significantly reduces patients' and physicians' fluoroscopic times by 50.5% and 68.6%, respectively, when used in conjunction with a noncontact mapping system to guide ablation of RVOT VPC/VT.		
Krittayaphong et al. Electrocardiographic predictors of long-term outcomes after radiofrequency ablation in patients with right-ventricular outflow tract tachycardia.<br>• Year published: 2006<br>• PMID: 16772366	Study type: Single-center observational<br>Size: 144	Patients with symptomatic RVOT PVC/VT	Monophasic R-wave in lead I during RVOT tachycardia is associated with unfavorable outcomes after RF ablation. This finding can help clinicians in the selection of patients for RF ablation and for the prediction of RF ablation outcome.		
Vestal et al. Electrocardiographic predictors of failure and recurrence in patients with idiopathic right ventricular outflow tract tachycardia and ectopy who underwent radiofrequency catheter ablation.<br>• Year published: 2003<br>• PMID: 14661169	Study type: Single-center observational<br>Size: 91	Symptomatic RVOT PVC/VT	Ablation with ectopy over VT as template arrhythmia, presence of QRS morphologic variation, wider mean QRS width, and taller mean R-wave amplitude in lead II were identified ECG predictors of failed RVOT VT/ectopy ablation. The only ECG predictor of recurrence was the presence of RVOT VT or ectopy QRS morphologic variation.		

Study name or acronym; Author; Year published; PMID	Aim of study; Endpoints; Study type (RCT, observational — multicenter or single center; case series or other [if RCT include intervention and comparator]); Study size (N)	Patient population with inclusion and exclusion criteria	Results (absolute event rates, *P* values; OR or RR; 95% CI)	Other relevant findings or adverse events	Limitations; Other comments; Conclusions
Miyazawa et al. Rapid mapping and differentiation in ventricular outflow tract arrhythmia using non-contact mapping.<br>• Year published: 2017<br>• PMID: 28386822	Study type: Single-center observational<br>Size: 45	Patients with symptomatic RVOT PVC	The isopotential area of VOTAs originating from the RVOT, compared to the other sites, spread more elliptically and slowly. The propagation pattern obtained from NCM can provide useful information and efficient strategy for VOTA ablation.		
Akdeniz et al. Catheter ablation of idiopathic right ventricular arrhythmias in children with limited fluoroscopy.<br>• Year published: 2016<br>• PMID: 27184808	Study type: Single-center observational<br>Size: 35	Children with symptomatic RV arrhythmias	Catheter ablation of idiopathic right VA in children can be performed safely and effectively with limited fluoroscopy using the EnSite Velocity system.		
Morady et al. Long-term results of catheter ablation of idiopathic right ventricular tachycardia.<br>• Year published: 1990<br>• PMID: 2242533	Study type: Single-center observational<br>Size: 10	Patients with symptomatic RVOT VT	Catheter ablation can be performed safely and effectively.		
Liao et al. Idiopathic ventricular arrhythmias originating from the pulmonary sinus cusp: prevalence, electrocardiographic/electrophysiological characteristics, and catheter ablation.<br>• Year published: 2015<br>• PMID: 26670064	Study type: Single-center observational<br>Size: 218	VAs arising from the PSC	VAs arising from the PSC are not uncommon, and right-cusp VAs have unique electrocardiographic characteristics. These VAs can be successfully ablated within the PSC.		

**Study name or acronym; Author; Year published; PMID**	**Aim of study; Endpoints; Study type (RCT, observational — multicenter or single center; case series or other [if RCT include intervention and comparator]); Study size (N)**	**Patient population with inclusion and exclusion criteria**	**Results (absolute event rates, *P* values; OR or RR; 95% CI)**	**Other relevant findings or adverse events**	**Limitations; Other comments; Conclusions**
Bogun et al. Radiofrequency ablation of frequent, idiopathic premature ventricular complexes: comparison with a control group without intervention.<br>• Year published: 2007<br>• PMID: 17599667	Study type: Single-center Control group<br>Size: 60	Patients with symptomatic RVOT VT	LV dysfunction in the setting of frequent idiopathic PVCs might represent a form of cardiomyopathy that can be reversed by catheter ablation of the PVCs.		
Chen et al. Intramural outflow tract ventricular tachycardia: anatomy, mapping, and ablation.<br>• Year published: 2014<br>• PMID: 25336368	Study type: Single-center observational<br>Size: 2	Patients with symptomatic RVOT PVC	Multiple ECG algorithms have been devised to predict the site of origin for outflow tract tachycardias. However, there are different ECG characteristics in our 2 cases with the same origin. One reason might be preferential conduction to either aspect of the ventricular septum. For this reason, comprehensive mapping of the GCV and SPB veins can be useful to precisely delineate the location of origin.		
Teh et al. Bipolar radiofrequency catheter ablation for refractory ventricular outflow tract arrhythmias.<br>• Year published: 2014<br>• PMID: 24890707	Study type: Single-center observational<br>Size: 73	RVOT PVC refractory to conventional ablation	This report demonstrates the potential utility of bipolar RFCA in patients with outflow tract PVCs that fail unipolar RF.		

Study name or acronym; Author; Year published; PMID	Aim of study; Endpoints; Study type (RCT, observational — multicenter or single center; case series or other [if RCT include intervention and comparator]); Study size (N)	Patient population with inclusion and exclusion criteria	Results (absolute event rates, *P* values; OR or RR; 95% CI)	Other relevant findings or adverse events	Limitations; Other comments; Conclusions
Lamba et al. Radiofrequency catheter ablation for the treatment of idiopathic premature ventricular contractions originating from the right ventricular outflow tract: a systematic review and meta-analysis.<br>• Year published: 2014<br>• PMID: 23980900	Study type: Review and meta-analysis<br>Size: 70	RVOT PVC	RFCA reduces the number of PVCs and improves cardiac function in patients with idiopathic frequent PVCs originating from the RVOT.		
Frey et al. Successful treatment of idiopathic left ventricular outflow tract tachycardia by catheter ablation or minimally invasive surgical cryoablation.<br>• Year published: 2000<br>• PMID: 10833708	Study type: Single-center observational<br>Size: 37	LVOT PVC	Successful treatment is achieved by RF catheter ablation or minimally invasive surgical cryoablation.		
Krebs et al. Ventricular tachycardias mimicking those arising from the right ventricular outflow tract.<br>• Year published: 2000<br>• PMID: 10695461	Study type: Single-center observational<br>Size: 29	LVOT PVC	The absence of an R wave in lead V1 and a late precordial transition zone suggest an RVOT origin of VT, whereas an early precordial transition zone characterizes VTs that mimic an RVOT origin. The latter VTs occasionally can be ablated from the LVOT. Recognition of these ECG features could help the physician advise patients and direct one's approach to ablation.		

Study name or acronym; Author; Year published; PMID	Aim of study; Endpoints; Study type (RCT, observational — multicenter or single center; case series or other [if RCT include intervention and comparator]); Study size (N)	Patient population with inclusion and exclusion criteria	Results (absolute event rates, *P* values; OR or RR; 95% CI)	Other relevant findings or adverse events	Limitations; Other comments; Conclusions
Kumagai et al. Electrocardiographic characteristics of the variants of idiopathic left ventricular outflow tract ventricular tachyarrhythmias.<br>• Year published: 2008<br>• PMID: 18266673	Study type: Single-center observational<br>Size: 45	LVOT VTs	Despite many morphological similarities, the LVOT-VTs originating from the AMC, anterior MA, and ASC could be identified by our proposed electrocardiographic characteristics in order to safely perform RFCA.		
Latchamsetty et al. Multicenter outcomes for catheter ablation of idiopathic premature ventricular complexes.<br>• Year published: 2015<br>• PMID: 29759353	Study type: Multicenter observational<br>Retrospective<br>Size: 1185 (55% female)	Idiopathic PVCs	Catheter ablation of frequent PVCs is a low-risk and often effective treatment strategy to eliminate PVCs and associated symptoms. In patients with PVC-induced cardiomyopathy, cardiac function is frequently restored after successful ablation.		
Kamakura et al. Localization of optimal ablation site of idiopathic ventricular tachycardia from right and left ventricular outflow tract by body surface ECG.<br>• Year published: 1998<br>• PMID: 9769306	Study type: Single-center observational<br>Size: 40	RVOT-LVOT PVCs	The origin or the optimum ablation site of idiopathic VT from RVOT and LVOT can be localized with the use of indexes obtained with a BSM or 12-lead ECG.		

Study name or acronym; Author; Year published; PMID	Aim of study; Endpoints; Study type (RCT, observational — multicenter or single center; case series or other [if RCT include intervention and comparator]); Study size (N)	Patient population with inclusion and exclusion criteria	Results (absolute event rates, *P* values; OR or RR; 95% CI)	Other relevant findings or adverse events	Limitations; Other comments; Conclusions
Yamada et al. Electrocardiographic characteristics of ventricular arrhythmias originating from the junction of the left and right coronary sinuses of Valsalva in the aorta: the activation pattern as a rationale for the electrocardiographic characteristics.<br>• Year published: 2008<br>• PMID: 18242537	Study type: Single-center observational<br>Size: 155	RVOT-LVOT PVCs	This study revealed that a qrS pattern in leads V1–V3 suggests a site of origin at the L-RCC.		
Tada et al. Significance of two potentials for predicting successful catheter ablation from the left sinus of Valsalva for left ventricular epicardial tachycardia.<br>• Year published: 2004<br>• PMID: 15305952	Study type: Single-center observational<br>Size: 23	LSOV<br><br>PVC-VT	The appearance of the P2 potential or a delay in the preexisting P2 potential after application of RF energy was observed only at the successful ablation sites (*P*<.001). In 18 control individuals who had no LV-VT, no P2 potential was recorded within the LSOV. Although the P1 potential might be useful for identifying the successful ablation site, its sensitivity is low. The appearance of the P2 potential or an increasingly delayed P2 potential after application of RF energy might be more useful than the P1 potential for predicting successful ablation.		

Study name or acronym; Author; Year published; PMID	Aim of study; Endpoints; Study type (RCT, observational — multicenter or single center; case series or other [if RCT include intervention and comparator]); Study size (N)	Patient population with inclusion and exclusion criteria	Results (absolute event rates, *P* values; OR or RR; 95% CI)	Other relevant findings or adverse events	Limitations; Other comments; Conclusions
Ouyang et al. Repetitive monomorphic ventricular tachycardia originating from the aortic sinus cusp: electrocardiographic characterization for guiding catheter ablation.<br>• Year published: 2002<br>• PMID: 11823089	Study type: Single-center observational<br>Size: 15	VT originating from the aortic sinus cusp	On the surface ECG, RMVT from the ASC has a QRS morphology similar to that of RVOT arrhythmias. The indexes of R-wave duration and RS-wave amplitude can be used to differentiate between the two origins. RFCA can be safely performed within the left ASC with a catheter cannulating the LMCA.		
Tada et al. Left ventricular epicardial outflow tract tachycardia: a new distinct subgroup of outflow tract tachycardia.<br>• Year published: 2001<br>• PMID: 11502049	Study type: Single-center observational<br>Size: 31	OT-VT from the LV epicardium	OT-VT originating from the LV epicardium is not uncommon and has characteristic ECG findings. Some of them can be ablated from the LSOV.		
Baman et al. Mapping and ablation of epicardial idiopathic ventricular arrhythmias from within the coronary venous system.<br>• Year published: 2010<br>• PMID: 20400776	Study type: Single-center observational<br>Size: 27	Idiopathic VAS epicardial origin	Almost 15% of idiopathic VAs have an epicardial origin. ECG characteristics help to differentiate epicardial arrhythmias from endocardial VAs. The SOO of epicardial arrhythmias can be ablated from within the CVS in approximately 70% of patients.		

Study name or acronym; Author; Year published; PMID	Aim of study; Endpoints; Study type (RCT, observational — multicenter or single center; case series or other [if RCT include intervention and comparator]); Study size (N)	Patient population with inclusion and exclusion criteria	Results (absolute event rates, *P* values; OR or RR; 95% CI)	Other relevant findings or adverse events	Limitations; Other comments; Conclusions
Carrigan et al. Anatomic relationships between the coronary venous system, surrounding structures, and the site of origin of epicardial ventricular arrhythmias.<br>• Year published: 2014<br>• PMID: 25066476	Study type: Single-center observational<br>Size: 17	Patients with idiopathic epicardial VAs	The CVS is closer to the SOO of epicardial idiopathic VAs than the pericardial space, the ventricular endocardium, and the aortic cusps. Given the proximity to coronary arteries at the SOO, RF energy often cannot be safely delivered to eliminate a VA, and ablation might also need to be performed from adjacent structures. A subxiphoid pericardial ablation procedure has a low probability of success in patients with idiopathic epicardial VAs.		
Santangeli et al. Percutaneous epicardial ablation of ventricular arrhythmias arising from the left ventricular summit: outcomes and electrocardiogram correlates of success.<br>• Year published: 2015<br>• PMID: 25637596	Study type: Single-center observational<br>Size: 23	Twenty-three patients with VAs arising from the LV summit	Epicardial instrumentation for mapping and ablation of VAs arising from the LV summit is successful only in a minority of patients because of close proximity to major coronary arteries and epicardial fat.		

Study name or acronym; Author; Year published; PMID	Aim of study; Endpoints; Study type (RCT, observational — multicenter or single center; case series or other [if RCT include intervention and comparator]); Study size (N)	Patient population with inclusion and exclusion criteria	Results (absolute event rates, *P* values; OR or RR; 95% CI)	Other relevant findings or adverse events	Limitations; Other comments; Conclusions
Nagashima et al. Ventricular arrhythmias near the distal great cardiac vein: challenging arrhythmia for ablation.<br>• Year published: 2014<br>• PMID: 25110163	Study type: Single-center observational<br>Size: 30	Catheter ablation for VA near the distal great cardiac vein.	Ablation for this arrhythmia is challenging and often limited by the adjacent coronary vessels.		
Callans et al. Repetitive monomorphic tachycardia from the left ventricular outflow tract: electrocardiographic patterns consistent with a left ventricular site of origin.<br>• Year published: 1997<br>• PMID: 9120154	Study type: Single-center observational<br>Size: 33	This study sought to characterize the electrocardiographic patterns predictive of LV sites of origin of RMVT.	RMVT can arise from the outflow tract of both the right and left ventricles. RMVTs with a precordial R wave transition at or before lead V2 are consistent with an LV origin.		
Bala et al. Electrocardiographic and electrophysiologic features of ventricular arrhythmias originating from the right/left coronary cusp commissure.<br>• Year published: 2010<br>• PMID: 20097621	Study type: Single-center observational<br>Size: 37	The purpose of this study was to identify the characteristics associated with VAs originating from the RCC-LCC commissure.	RCC-LCC aortic cusp ventricular arrhythmias are common and have a QS morphology in lead V(1) with notching on the downward deflection with precordial transition at lead V(3).		

Study name or acronym; Author; Year published; PMID	Aim of study; Endpoints; Study type (RCT, observational — multicenter or single center; case series or other [if RCT include intervention and comparator]); Study size (N)	Patient population with inclusion and exclusion criteria	Results (absolute event rates, *P* values; OR or RR; 95% CI)	Other relevant findings or adverse events	Limitations; Other comments; Conclusions
Calkins et al. Relation between efficacy of radiofrequency catheter ablation and site of origin of idiopathic ventricular tachycardia.<br>• Year published: 1993<br>• PMID: 8456762	Study type: Observational study<br>Size: 18 patients<br>Endpoint: Noninducibility	Inclusion: Idiopathic VT from RV and LV<br><br>Exclusion: VTs in patients with structural heart disease	All (100%) RVOT VTs were ablated, 5 of 10 (50%) VTs from other sites were ablated, in 3 RV and 2 LV VTs RF failed, and there was no matching PM in 4 of 5 failures.	RVOT VT ablation was effective; other sites were less effective; (*P*=.03).<br>No complications	Limitations: No rigorous R/o of ARVC.<br><br>Conclusions: Idiopathic RV and LV VT can be eliminated in most patients.<br><br>Pace mapping to select adequate target site is predictive of acute success rate.
Rodriguez et al. Predictors for successful ablation of right- and left-sided idiopathic ventricular tachycardia.<br>• Year published: 1997<br>• PMID: 9036750	Study type: Observational study<br>Size: 48 patients<br>Endpoint: Noninducibility +/- isoproterenol	Inclusion: Idiopathic VT from RV and LV<br><br>Exclusion: VTs in patients with SHD	Some 29 of 35 (83%) RVOT VTs were ablated,<br>12 of 13 (92%) LV VTs were ablated, 5 of 13 VTs with PP failed in procedures of patients with >1 VT.	See left column, no complications indicated.	Idiopathic RV and LV VT can be eliminated in most patients.<br><br>PM predicts acute outcomes; inadequate PM and delta-wave at QRS onset predicts RF failure.

Study name or acronym; Author; Year published; PMID	Aim of study; Endpoints; Study type (RCT, observational — multicenter or single center; case series or other [if RCT include intervention and comparator]); Study size (N)	Patient population with inclusion and exclusion criteria	Results (absolute event rates, *P* values; OR or RR; 95% CI)	Other relevant findings or adverse events	Limitations; Other comments; Conclusions
Coggins et al. Radiofrequency catheter ablation as a cure for idiopathic tachycardia of both left and right ventricular origin.<br>• Year published: 1994<br>• PMID: 8176091	Study type: Observational study<br>Size: 28 patients<br>Endpoint: Noninducibility +/- isoproterenol	Inclusion: Idiopathic VT with RBBB + superior axis and LBBB + inferior axis.<br><br>Exclusions: Other morphologies than the included VT.	Ablation effective in 25 of 28 (89%) patients.<br><br>RVOT: Effective in 17 of 20 (85%) patients.<br><br>LV: Effective in 8 of 8 (100%) patients.	Complications: 1 patient with new aortic regurgitation; 1 patient with RBBB postablation in RVOT; 1 patient died after RV perforation	Limitations: Not all idiopathic VTs were included; only patients with RVOT VTs and patients with RBBB superior axis VT and 3 patients with concomitant SHD were included.<br>Conclusions: Idiopathic RV and LV VT can be eliminated in most patients. Pace-mapping helps to identify site of origin.
Wen et al. Radiofrequency ablation therapy in idiopathic left ventricular tachycardia with no obvious structural heart disease.<br>• Year published: 1994<br>• PMID: 8149535	Study type: Observational study<br>Size: 20 patients<br>Endpoint: Noninducibility +/- isoproterenol	Inclusion: Idiopathic LV VT<br><br>Exclusion: Right sided VTs	Ablation effective in 17 of 20 (85%) patients.<br><br>PP preceded successful ablation sites.<br><br>No complications.	NA	Limitations: Only fascicular VTs included.<br>Conclusions: Idiopathic LV VT can be eliminated in most patients. Matching pace maps not necessary to eliminate these VTs.

Study name or acronym; Author; Year published; PMID	Aim of study; Endpoints; Study type (RCT, observational — multicenter or single center; case series or other [if RCT include intervention and comparator]); Study size (N)	Patient population with inclusion and exclusion criteria	Results (absolute event rates, *P* values; OR or RR; 95% CI)	Other relevant findings or adverse events	Limitations; Other comments; Conclusions
Movsowitz et al. Idiopathic right ventricular outflow tract tachycardia: narrowing the anatomic location for successful ablation.<br>• Year published: 1996<br>• PMID: 8615312	Study type: Observational study<br>Size: 18 patients<br>Endpoint: Noninducibility +/- isoproterenol	Inclusion: Idiopathic RVOT VTs<br><br>Exclusion: Left-sided VTs and right-sided VTs from origins other than the RVOT.	Ablation effective in 16 of 18 patients.<br><br>Five patients with recurrence (3 within 24 hours) RVOT separated in regions with distinctive ECG features.	NA	Limitations: Only RVOT VTs included.<br>Conclusions: Idiopathic RV VT can be eliminated in most patients. ECG-guided catheter manipulation in RVOT helps to target VT origins.

Idiopathic Nonoutflow Tract Ventricular Arrhythmias

Study name or acronym; Author; Year published; PMID	Aim of study; Endpoints; Study type (RCT, observational — multicenter or single center; case series or other [if RCT include intervention and comparator]); Study size (N)	Patient population with inclusion and exclusion criteria	Results (absolute event rates, *P* values; OR or RR; 95% CI)	Other relevant findings or adverse events	Limitations; Other comments; Conclusions
Van Herendael et al. Idiopathic right ventricular arrhythmias not arising from the outflow tract: prevalence, electrocardiographic characteristics, and outcome of catheter ablation.<br>• Year published: 2011<br>• PMID: 21129502	Study type: Single-center review<br>Size: 29 patients	Inclusion criteria: Among 278 patients who underwent ablation for idiopathic VT or VPDs arising from the right ventricle, 29 (10%) had VT/VPDs from the lower RV body<br><br>Exclusion criteria: (1) Evidence of ARVD/C; (2) Presence of fractionated diastolic electrograms during VT or sinus rhythm; (3) Regions of low amplitude and prolonged duration on intracardiac electrograms	Results: Fourteen (48%) patients had VT/VPDs within 2 cm of the TVA, 8 (28%) from the basal and 7 (24%) from the apical RV segments. Among the VT/VPDs from the TVA, 8 (57%) originated from the free wall and 6 (43%) from the septum. All but one RV basal or apical VT/VPD originated from the free wall.<br><br>Clinical presentation: All VT/VPDs had an LBBB pattern. VT/VPDs from the free wall had longer QRS duration (*P*=.0032) and deeper S wave in lead V2 (*P*=.042) and V3 (*P*=.046) than those from the septum. Apical VT/VPDs more often had precordial R wave transition >V6 (*P*=.0001), smaller R wave in lead II (*P*=.024), and smaller S wave in lead aVR (*P*=.001) compared with VT/VPDs from basal RV or TVA.	RF catheter ablation eliminated VT and VPDs in 96% of patients. No complications were observed.	During median follow-up of 27 months (range 4–131 months), 81% of patients had elimination of all symptomatic VTs and VPDs. Nineteen percent had rare symptoms (8% without medications, 11% on beta-blocker).

Study name or acronym; Author; Year published; PMID	Aim of study; Endpoints; Study type (RCT, observational — multicenter or single center; case series or other [if RCT include intervention and comparator]); Study size (N)	Patient population with inclusion and exclusion criteria	Results (absolute event rates, *P* values; OR or RR; 95% CI)	Other relevant findings or adverse events	Limitations; Other comments; Conclusions
Sadek et al. Idiopathic ventricular arrhythmias originating from the moderator band: electrocardiographic characteristics and treatment by catheter ablation.<br>• Year published: 2015<br>• PMID: 25240695	Study type: Single-center observational<br>Size: 10 patients	Inclusion criteria: 10 patients with VAs mapped to moderator band in the RV undergoing catheter ablation<br><br>Exclusion criteria: None	Results: VF was clinical arrhythmia in 7 patients and monomorphic VT in 3 patients.	Six patients required a repeat procedure.<br><br>After mean follow-up of 21.5 ± 11.6 months, all patients were free of sustained VAs, with only 1 patient requiring antiarrhythmic drug therapy and 1 patient having isolated PVCs no longer inducing VF. There were no procedural complications.	VAs originating from the moderator band might present with VF.<br><br>Catheter ablation is effective, though the risk of requiring more than one procedure could be higher than for other sites.
Crawford et al. Ventricular arrhythmias originating from papillary muscles in the right ventricle.<br>• Year published: 2010<br>• PMID: 20206325	Study type: Single-site observational<br>Size: 8 patients	Inclusion criteria: VAs mapped to the papillary muscles in the right ventricle<br><br>Exclusion criteria: None	Results: A total of 15 distinct PAP VAs were mapped to the posterior (N=3), anterior (N=4), or septal (N=8) papillary muscles.	Successful ablation was achieved in all 8 patients.<br><br>The PVC burden was reduced from 17% ± 20% preablation to 0.6% ± 0.8% postablation.	PVCs and VTs can originate in the RV PAPs. RF ablation is effective in eliminating these arrhythmias, with low risk of complications.

Study name or acronym; Author; Year published; PMID	Aim of study; Endpoints; Study type (RCT, observational — multicenter or single center; case series or other [if RCT include intervention and comparator]); Study size (N)	Patient population with inclusion and exclusion criteria	Results (absolute event rates, *P* values; OR or RR; 95% CI)	Other relevant findings or adverse events	Limitations; Other comments; Conclusions
Tada et al. Idiopathic ventricular arrhythmias originating from the tricuspid annulus: prevalence, electrocardiographic characteristics, and results of radiofrequency catheter ablation.<br>• Year published: 2007<br>• PMID: 17198982	Study type: Single-center observational<br>Size: 38 patients	Inclusion criteria: Consecutive patients with idiopathic VAs mapped and ablated on the tricuspid annulus<br><br>Exclusion criteria: N/A	Results: Among 454 consecutive patients with idiopathic VAs, 38 (8%) VAs were found to originate from the tricuspid annulus; 28 (74%) originated from the septal tricuspid annulus; and 10 (26%) from the free-wall portion of the annulus. Catheter ablation eliminated 90% of free-wall VAs, but only 57% of septal tricuspid annular VAs. There were no complications.	Tricuspid annular VAs are not rare, and ablation has a higher efficacy for free-wall than septal sites.	
Santoro et al. Ventricular tachycardia originating from the septal papillary muscle of the right ventricle: electrocardiographic and electrophysiological characteristics.<br>• Year published: 2015<br>• PMID: 25229319	Study type: Single-center observational<br>Size: 8 patients	Inclusion criteria: Consecutive patients with idiopathic VAs mapped and ablated on the septal papillary muscle in the right ventricle<br><br>Exclusion criteria: N/A	Results: Among 155 consecutive patients without SHD who underwent catheter ablation of PVC/VT, 8 patients with PVC or VT from the septal RV PAP muscle were identified. The SOO of the arrhythmias was identified through activation/pace mapping and intracardiac echocardiography.	Septal RV PAP arrhythmias had a left superior axis and negative concordance or late R-wave transition in precordial leads. PVCs were spontaneous in 5 cases, were induced by isoproterenol in 2 cases, and induced by isoproterenol plus phenylephrine in 1 case. PVCs were never induced with calcium bolus and only rarely with burst pacing. Adenosine never terminated VT or suppressed the VT or PVC.	RF ablation was successful in all 8 patients. During a mean follow-up of 8 ± 4 months, mean PVC burden was reduced from 14% ± 3% preablation to 0.1% ± 0.2% postablation.

Study name or acronym; Author; Year published; PMID	Aim of study; Endpoints; Study type (RCT, observational — multicenter or single center; case series or other [if RCT include intervention and comparator]); Study size (N)	Patient population with inclusion and exclusion criteria	Results (absolute event rates, *P* values; OR or RR; 95% CI)	Other relevant findings or adverse events	Limitations; Other comments; Conclusions
Sasaki et al. Catheter ablation of ventricular arrhythmias arising from the basal septum of the right ventricle: characteristics and significance of junctional rhythm appearing during ablation.<br>• Year published: 2016<br>• PMID: 26739485	Study type: Single-center observational<br>Size: 86 patients	Inclusion criteria: Consecutive patients with VA origin in the basal septum of the RV, defined as the region from 1 to 5 o'clock of the TA in the left anterior oblique view and extending 2 cm anteriorly from the TA<br><br>Exclusion criteria: N/A	Results: Among 86 consecutive patients undergoing RFCA for VA from the RV, 12 (14%) (mean age, 71 ± 7 years).<br>The mean QRS duration of VA was 137 ± 8 ms with normal (10 of 12; 83 %) or left-deviated axis (2 of 12; 17%). Five patients (41%) had SHD, including three DCMs. RF energy was applied to the sites showing the earliest activation during VA and/or the best pace map. Complete elimination of VA was achieved in 11 (92%) patients. The successful ablation site was 12 ± 4 mm away from the HB electrogram recording site. Among 11 patients with successful ablation, 10 (91 %) exhibited junctional rhythm (mean cycle length, 638 ± 172 ms) during ablation without subsequent AV block.	All these patients were free from VA during 32 ± 21 months.	VA originating from the basal septum of the RV can be ablated effectively. Junctional rhythm appears in most cases without causing AV conduction disturbance.

Study name or acronym; Author; Year published; PMID	Aim of study; Endpoints; Study type (RCT, observational — multicenter or single center; case series or other [if RCT include intervention and comparator]); Study size (N)	Patient population with inclusion and exclusion criteria	Results (absolute event rates, *P* values; OR or RR; 95% CI)	Other relevant findings or adverse events	Limitations; Other comments; Conclusions
Yue-Chun et al. Idiopathic premature ventricular contractions and ventricular tachycardias originating from the vicinity of tricuspid annulus: results of radiofrequency catheter ablation in thirty-five patients.<br>• Year published: 2012<br>• PMID: 22551200	Study type: Single-center observational<br>Size: 35 patients	Inclusion criteria: Idiopathic VAs arising from the TVA<br><br>Exclusion criteria: N/A	Results: Among 35 patients with VAs arising from the TA, RFCA was successful in 32 (91.4%) over a median follow-up of 21 months. An rS pattern was recorded in lead V1 in 93.1% of patients with PVCs or IVTs from the free wall of the TA, vs 16.7% of patients with PVCs or IVTs from the septal TA, whereas a QS pattern in lead V1 occurred in 83.3% of patients with PVCs or IVTs from the septal TA vs 6.9% of patients with PVCs from the free wall of the TA. The precordial R wave transition occurred in lead V3 or earlier in all patients with PVCs or IVTs originating from the septal portion of the TA compared with transition beyond V3 in all patients with PVCs or IVTs from the free wall of the TA.	RFCA is an effective curative therapy for symptomatic PVCs or IVTs originating from the vicinity of the TA.	There are specific characteristics in ECG and the ablation site can be predicted by the surface ECG.

Study name or acronym; Author; Year published; PMID	Aim of study; Endpoints; Study type (RCT, observational — multicenter or single center; case series or other [if RCT include intervention and comparator]); Study size (N)	Patient population with inclusion and exclusion criteria	Results (absolute event rates, *P* values; OR or RR; 95% CI)	Other relevant findings or adverse events	Limitations; Other comments; Conclusions
Enriquez et al. Inferior lead discordance in ventricular arrhythmias: A specific marker for certain arrhythmia locations.<br>• Year published: 2017<br>• PMID: 28677887	Study type: Single-center observational<br>Size: 25 patients	Inclusion criteria: Patients with RV VAs demonstrating ILD, with a positive QRS in lead II and negative QRS in lead III, or vice versa<br><br>Exclusion criteria: Patients without ILD	Results: Of 281 patients, 25 (8.9%) exhibited ILD. In patients with positive/negative discordance (n=18), the source was mapped to the para-Hisian region in 14 cases and to the RV MB or PAP in 4, whereas all those with negative/positive discordance (n=7) were mapped to the anterolateral PM.<br><br>In the group with positive/negative discordance, a later precordial transition (>V4), wider QRS duration, and the presence of notch in the inferior leads pointed toward an RV MB or PAP origin. Complete PVC/VT suppression was achieved in 72%. In 2 patients with para-Hisian PVCs, ablation was not attempted due to risk of heart block.	The presence of ILD is associated with particular anatomical locations, namely, the para-Hisian region, RV MB/PAP, and ALPAP.	The outcomes of ablation are more modest compared with other idiopathic VAs, reflecting the technical difficulties associated with these anatomical locations, such as the proximity to the conduction system in para-Hisian VAs or stability issues when ablating the PAPs or MB.

Study name or acronym; Author; Year published; PMID	Aim of study; Endpoints; Study type (RCT, observational — multicenter or single center; case series or other [if RCT include intervention and comparator]); Study size (N)	Patient population with inclusion and exclusion criteria	Results (absolute event rates, *P* values; OR or RR; 95% CI)	Other relevant findings or adverse events	Limitations; Other comments; Conclusions
Yamada et al. Idiopathic ventricular arrhythmias originating from the parietal band: electrocardiographic and electrophysiological characteristics and outcome of catheter ablation.<br>• Year published: 2017<br>• PMID: 28794085	Study type: Single-center observational<br>Size: 14 patients	Inclusion criteria: Consecutive patients with idiopathic RV VAs with SOO in the RV parietal band<br><br>Exclusion criteria: None	Results: Parietal band VAs had a LBBB and left inferior (n=12) or superior (n=2) axis pattern with the presence of a notch in the middle of the QRS in all cases, precordial transition at ≤lead V3 in 7 patients, and a slow QRS onset in 4 patients.<br><br>During parietal band VAs, a far-field ventricular electrogram with an early activation was always recorded in the HB region, regardless of the location of the VA origins.<br><br>During the catheter ablation, a mean number of 10.4 ± 7.4 RF applications were delivered. Catheter ablation was successful in 10 patients, and VAs recurred in 4 during a mean follow-up period of 41 ± 24 months.	Idiopathic VAs from the parietal band are rare. Catheter ablation of parietal band VAs is challenging, requiring a large amount of RF energy delivery for successful ablation, with a relatively high recurrence rate.	

Study name or acronym; Author; Year published; PMID	Aim of study; Endpoints; Study type (RCT, observational — multicenter or single center; case series or other [if RCT include intervention and comparator]); Study size (N)	Patient population with inclusion and exclusion criteria	Results (absolute event rates, *P* values; OR or RR; 95% CI)	Other relevant findings or adverse events	Limitations; Other comments; Conclusions
Ceresnak et al. Characteristics of ventricular tachycardia arising from the inflow region of the right ventricle.<br>• Year published: 2012<br>• PMID: 22554461	Study type: Single-center observational<br>Size: 9 patients	Inclusion criteria: Idiopathic VAs arising from the inflow tract of the right ventricle<br>Exclusion criteria: N/A	Results: Among 70 patients undergoing EPS for VT arising from the right ventricle, 9 (13%) patients met the inclusion criteria for RVI VT. The median age was 46 years (range, 14–71), and VT cycle length was 295 milliseconds (range, 279–400 milliseconds). All VTs had an LBBB morphology. An inferior QRS axis was noted in 7 (78%) of 9 patients and a left superior axis in 2 (22%) patients. A QS or rS pattern was noted in all patients in aVR and V1. A transition from S to R wave occurred in V3 to V5 in all patients, with 78% of the patients transitioning in V4 or V5. Ablation was attempted in 8 (89%) of 9 patients and was successful in 6 (67%) of 9 patients. Ablation was limited in all unsuccessful patients due to proximity to the HB and risk of complete heart block.	Electrocardiographic findings of an LBBB with a normal QRS axis, QS or rS patterns in aVR and V1, and late S to R transition (V4/V5) are commonly found in RVI VT.	Because of the proximity to the HB, ablation of RVI VT might be more challenging than that of RVOT VT.

Study name or acronym; Author; Year published; PMID	Aim of study; Endpoints; Study type (RCT, observational — multicenter or single center; case series or other [if RCT include intervention and comparator]); Study size (N)	Patient population with inclusion and exclusion criteria	Results (absolute event rates, *P* values; OR or RR; 95% CI)	Other relevant findings or adverse events	Limitations; Other comments; Conclusions
Li et al. Combined approach improves the outcomes of catheter ablation of idiopathic ventricular arrhythmias originating from the vicinity of tricuspid annulus.<br>• Year published: 2014<br>• PMID: 24456278	Study type: Single-center observational<br>Size: 36 patients	Inclusion criteria: VAs arising from the vicinity of the TA<br><br>Exclusion Criteria: N/A	Results: Of 486 consecutive patients who underwent catheter ablation of idiopathic VAs, 36 patients with TA origin were identified. A transfemoral vein approach was attempted first. If patients had a failed prior ablation or if VAs recurred during follow-up, they were referred for repeat ablation via the subclavian vein approach (11 patients, 30.6%).<br><br>After the final procedure, VAs recurred in 2 patients and the success rate increased from 69.5% (25 of 36) to 94.4% (34 of 36). Amplitudes of the atrial electrograms of all successful ablation sites via the subclavian vein approach was <0.036 mV.	The subclavian vein approach plus transfemoral vein approach improves the outcomes of catheter ablation of idiopathic VAs originating from the vicinity of the TA.	The subclavian vein approach is a feasible alternative for VAs, which has been refractory to ablation via the inferior approach.

Study name or acronym; Author; Year published; PMID	Aim of study; Endpoints; Study type (RCT, observational — multicenter or single center; case series or other [if RCT include intervention and comparator]); Study size (N)	Patient population with inclusion and exclusion criteria	Results (absolute event rates, *P* values; OR or RR; 95% CI)	Other relevant findings or adverse events	Limitations; Other comments; Conclusions
Lian-Pin et al. Catheter ablation of idiopathic premature ventricular contractions and ventricular tachycardias originating from right ventricular septum.<br>• Year published: 2013<br>• PMID: 23825610	Study type: Single-center observational<br>Size: 29 patients	Inclusion criteria: Consecutive patients with idiopathic RV VAs with SOO in the RV septum<br><br>Exclusion criteria: None	Results: Of 581 patients with idiopathic VAs, 29 had symptomatic PVCs or IVTs originating from the RV septum (5%). Twenty (69%) had PVCs or IVTs from the septal portion of the tricuspid valvular RV region (3 from superoseptum, 15 from midseptum, 2 from inferoseptum), and 9 (31%) from the septal portion of the basal RV (1 from superoseptum, 4 from midseptum, 4 from inferoseptum). There were different characteristics of ECG of PVCs/VT originating from the different portions of the RV septum. Twenty-seven of 29 patients with PVCs or IVTs arising from the RV septum were successfully ablated (93.1% acute success).	RFCA was effective and safe for the PVCs and IVTs arising from the RV septum.	

Study name or acronym; Author; Year published; PMID	Aim of study; Endpoints; Study type (RCT, observational — multicenter or single center; case series or other [if RCT include intervention and comparator]); Study size (N)	Patient population with inclusion and exclusion criteria	Results (absolute event rates, *P* values; OR or RR; 95% CI)	Other relevant findings or adverse events	Limitations; Other comments; Conclusions
Wei et al. Safety and efficacy of catheter ablation of ventricular arrhythmias with para-Hisian origin via a systematic direct approach from the aortic sinus cusp.<br>• Year published: 2018<br>• PMID: 29803021	Study type: Single-center observational<br>Size: 21 patients	Inclusion criteria: Twenty-one consecutive patients with VAs of para-Hisian origin were included<br><br>Exclusion criteria: N/A	Results: RF energy resulted in elimination of VAs in 17 of 21 (81%) patients. In the remaining 4 patients, RF application was performed at the RV septum target site around the HB region, and the clinical VAs were finally successfully eliminated without junctional rhythm in 2 of 4 patients. During a mean follow-up of 34.8 ± 11.3 months, 1 of 19 patients had VA recurrence. No procedural-related complications occurred.	Catheter ablation of the VAs originating from para-Hisian area via a direct approach can be safe and effective in most unselected patients.	
Komatsu et al. Catheter ablation of ventricular arrhythmias arising from the right ventricular septum close to the His bundle: features of the local electrogram at the optimal ablation site.<br>• Year published: 2011<br>• PMID: 21332864	Study type: Single-center observational<br>Size: 16 patients	Inclusion criteria: Consecutive patients with idiopathic RV VAs with SOO in the RV para-Hisian region<br><br>Exclusion criteria: None	Results: Among 190 consecutive patients with idiopathic VAs with LBBB morphology and inferior-axis deviation, 16 were found to have a successful ablation site in the right ventricle close to the HB region (para-Hisian group). These were compared with RVOT patients. The bipolar electrogram at the successful ablation sites in the para-Hisian group exhibited a significantly greater R-wave duration, lower R-wave amplitude, and slower upright	The successful ablation site of the para-Hisian VAs had distinctive local electrogram characteristics.	A longer R-wave duration of the bipolar electrogram with high-frequency potentials could be a novel predictor of a successful ablation.

Study name or acronym; Author; Year published; PMID	Aim of study; Endpoints; Study type (RCT, observational — multicenter or single center; case series or other [if RCT include intervention and comparator]); Study size (N)	Patient population with inclusion and exclusion criteria	Results (absolute event rates, *P* values; OR or RR; 95% CI)	Other relevant findings or adverse events	Limitations; Other comments; Conclusions
			deflection of the initial R wave than the RVOT group (all *P*<.001). In the para-Hisian group, a total of 56 RF energy applications were delivered, of which the local electrograms at 16 successful and 40 unsuccessful ablation sites were reviewed. High-frequency R-wave potentials from the bipolar electrogram were present in 14 (88%) of the successful ablation sites. An R-wave duration of greater than 34 ms had a discriminatory power for indicating the site of a successful ablation (area under the ROC curve 0.90, sensitivity 94%, specificity 80%).		
**Nonrandomized Trials of LV Summit and Aortomitral Continuity VA Ablation**					
Yamada et al. Idiopathic ventricular arrhythmias originating from the left ventricular summit: anatomic concepts relevant to ablation.<br>• Year published: 2010<br>• PMID: 20855374	Study type: Single-center observational<br>Size: 27 patients	Inclusion criteria: Among 221 consecutive patients with LV idiopathic VAs, 27 patients had VAs mapped and ablated on the Summit of the LV<br><br>Exclusion criteria: N/A	Results: Successful ablation from the GCV occurred in 14 patients and ablation on the epicardial surface of the LV occurred in 4. In 5 patients, ablation was abandoned because of origin in the inaccessible region. In 4 patients, ablation was abandoned due to close proximity to epicardial coronary artery.	LV summit VAs can be ablated within the GCV or inferior to the GCV on the epicardial surface, although sites superior to the GCV are often inaccessible to ablation.	

Study name or acronym; Author; Year published; PMID	Aim of study; Endpoints; Study type (RCT, observational — multicenter or single center; case series or other [if RCT include intervention and comparator]); Study size (N)	Patient population with inclusion and exclusion criteria	Results (absolute event rates, *P* values; OR or RR; 95% CI)	Other relevant findings or adverse events	Limitations; Other comments; Conclusions
Mountantonakis et al. Ventricular arrhythmias from the coronary venous system: prevalence, mapping, and ablation.<br>• Year published: 2015<br>• PMID: 25766774	Study type: Single-center observational<br>Size: 47 patients	Inclusion criteria: Among 511 consecutive patients with nonscar-related VAs, 47 were found to have a SOO within the CVS<br><br>Exclusion criteria: N/A	Results: Twenty-five (53%) were in the GCV, 19 (40%) in the anterior interventricular vein, and 3 (7%) in the middle cardiac vein. Successful ablation was achieved in 17 of 18 (94%) ablated at the earliest CVS site and in 16 of 29 (55%) ablated at adjacent CVS or non-CVS sites.	Although ablation at the earliest CVS site is effective, it is often (62%) precluded, mainly because of proximity to coronary arteries. Ablation at adjacent CVS and non-CVS sites can be successful in 55% of these anatomically challenging cases, for an overall ablation success rate of 70%.	
Nagashima et al. Ventricular arrhythmias near the distal great cardiac vein: challenging arrhythmia for ablation.<br>• Year published: 2014<br>• PMID: 25110163	Study type: Single-site observational<br>Size: 30 patients	Inclusion criteria: 30 patients with VAs with early activation within the GCV<br><br>Exclusion criteria: N/A	Results: Angiography in 27 patients showed earliest GCV site within 5 mm of a coronary artery in 20 (74%). Ablation was performed in the GCV in 15 patients, which abolished VA in 8. Ablation was attempted at adjacent non-GCV sites in 19 patients, which abolished VA in 5 patients (4 from the left ventricular endocardium and 1 from the left coronary cusp). After a median of 2.8 months, 13 patients remained free of VA. Major complications occurred in 4 patients, including coronary injury requiring stenting.	Ablation within the GCV requires careful attention to the proximity of coronary arteries, with the potential for coronary arterial injury.	

Study name or acronym; Author; Year published; PMID	Aim of study; Endpoints; Study type (RCT, observational — multicenter or single center; case series or other [if RCT include intervention and comparator]); Study size (N)	Patient population with inclusion and exclusion criteria	Results (absolute event rates, *P* values; OR or RR; 95% CI)	Other relevant findings or adverse events	Limitations; Other comments; Conclusions
Yamada et al. Radiofrequency catheter ablation of idiopathic ventricular arrhythmias originating from intramural foci in the left ventricular outflow tract: efficacy of sequential versus simultaneous unipolar catheter ablation.<br>• Year published: 2015<br>• PMID: 25637597	Study type: Single-center observational study<br>Size: 64 patients	Inclusion criteria: 64 consecutive patients with symptomatic idiopathic sustained VTs (N=14), NSVT (N=15), or PVCs (N=35), which presumed origins identified in the AMC, LV summit, or intramural sites between the endocardium and epicardium<br><br>Exclusion criteria: N/A	Results: Of 64 patients, 14 were identified with intramural foci between the endocardium and epicardium that required sequential or simultaneous irrigated unipolar RFCA from the endocardial and epicardial sides for their elimination. Simultaneous ablation was most likely to be required when the distance between the endocardial and epicardial ablation sites was >8 mm and the earliest local ventricular activation time relative to the QRS onset during the VAs was <30 ms at both ablation sites.	LVOT VAs originating from intramural foci could usually be eliminated by sequential unipolar radiofrequency ablation and sometimes required simultaneous ablation from both the endocardial and epicardial sides.	
Tada et al. Idiopathic ventricular arrhythmia arising from the mitral annulus: a distinct subgroup of idiopathic ventricular arrhythmias.<br>• Year published: 2005<br>• PMID: 15766824	Study type: Single-center observational<br>Size: 19 patients	Inclusion criteria: Consecutive patients with VAs mapped to the mitral valve annulus<br><br>Exclusion criteria: N/A	Results: Of 352 patients with idiopathic VAs, 19 (5%) had mitral annular VAs; 11 (58%) originated from the anterolateral mitral annulus, 2 from the posterior mitral annulus, and 6 from the posteroseptal mitral annulus. Successful ablation was achieved in 19 of 19 (100%) patients. No complications were observed.	VAs can arise from the anterolateral, posterior, and posteroseptal regions of the mitral annulus and can be effectively and safely ablated with RF current.	

Study name or acronym; Author; Year published; PMID	Aim of study; Endpoints; Study type (RCT, observational — multicenter or single center; case series or other [if RCT include intervention and comparator]); Study size (N)	Patient population with inclusion and exclusion criteria	Results (absolute event rates, *P* values; OR or RR; 95% CI)	Other relevant findings or adverse events	Limitations; Other comments; Conclusions
			Over a follow-up period of 21 ± 15 months, there were no recurrences of VAs after ablation.		
Doppalapudi et al. Idiopathic focal epicardial ventricular tachycardia originating from the crux of the heart.<br>• Year published: 2009<br>• PMID: 19121799	Study type: Single-center observational<br>Size: 4 patients	Inclusion criteria: Among 340 patients with idiopathic VT referred for ablation, 4 were identified with VT that was mapped to the epicardium at the crux<br><br>Exclusion criteria: N/A	Results: VT was sustained and rapid (mean cycle length 264 ms) in all patients and was associated with syncope or presyncope in 3. VT was induced with programmed stimulation or burst pacing in all 4 patients but required isoproterenol infusion in 3.	Idiopathic VT can arise by a focal mechanism from the epicardium at the crux in close proximity to the posterior descending coronary artery. This syndrome can result in rapid, catecholamine-sensitive VT and requires careful attention to the posterior descending coronary artery during ablation.	

Study name or acronym; Author; Year published; PMID	Aim of study; Endpoints; Study type (RCT, observational — multicenter or single center; case series or other [if RCT include intervention and comparator]); Study size (N)	Patient population with inclusion and exclusion criteria	Results (absolute event rates, *P* values; OR or RR; 95% CI)	Other relevant findings or adverse events	Limitations; Other comments; Conclusions
Yamada et al. Idiopathic left ventricular arrhythmias originating adjacent to the left aortic sinus of Valsalva: electrophysiological rationale for the surface electrocardiogram.<br>• Year published: 2010<br>• PMID: 19804552	Study type: Single-center observational study<br>Size: 21 patients	Inclusion criteria: All patients who underwent successful catheter ablation of VAs at the AMC<br><br>Exclusion criteria: N/A	Results: 48 consecutive patients undergoing successful catheter ablation of idiopathic VAs originating from the LCC (N= 29), AMC (N=10), and GCV or anterior interventricular cardiac vein (Epi, N= 9). An S wave in lead V5 or V6 occurred significantly more often during both the VAs and the pacing from the AMC than during that from the LCC and Epi (*P*<.05 vs *P*=.0001). For discriminating whether VA origins can be ablated endocardially or epicardially, the MDI (the shortest time to the maximum deflection in any precordial lead or QRS duration) was reliable for VAs arising from the AMC (100%) but was less reliable for the LCC (73%) and Epi (67%) VAs. In 3 (33%) of the Epi VAs, the site of an excellent pace map was located transmurally opposite to the successful ablation site (LCC=1 and AMC=2).	The MDI has limited value for discriminating endocardial from epicardial VA origins in sites adjacent to the LSOV, probably due to preferential conduction, intramural VA origins, or myocardium in contact with the LCC.	

**Study name or acronym; Author; Year published; PMID**	**Aim of study; Endpoints; Study type (RCT, observational — multicenter or single center; case series or other [if RCT include intervention and comparator]); Study size (N)**	**Patient population with inclusion and exclusion criteria**	**Results (absolute event rates, *P* values; OR or RR; 95% CI)**	**Other relevant findings or adverse events**	**Limitations; Other comments; Conclusions**
Hai et al. Electrophysiologic characteristics of ventricular arrhythmias arising from the aortic mitral continuity: potential role of the conduction system.<br>• Year published: 2015<br>• PMID: 25425429	Study type: Single-center observational study<br>Size: 21 patients	Inclusion criteria: All patients undergoing catheter ablation of idiopathic VAs from the AMC<br><br>Exclusion criteria: N/A	Results: Of the 21 patients (mean age 53.2 ± 13.4 years, 47.6% men) who underwent VA ablation at the AMC with acute success, prepotentials (PPs) were found at the ablation sites preceding the VEGM during arrhythmias in 13 (61.9%) patients and during sinus rhythm in 7 (53.8%) patients. VAs with prepotentials were associated with a significantly higher burden of PVCs (26.1% ± 10.9% vs 14.9% ± 10.1%; *P*=.03), shorter VEGM to QRS intervals (9.0 ± 28.5 milliseconds vs 33.1 ± 8.8 milliseconds; *P*=.03), lower pace map scores (8.7 ± 1.6 vs 11.4 ± 0.8, *P*=.001), and a trend toward shorter V-H intervals during VA (32.1 ± 38.6 milliseconds vs 76.3 ± 11.1 milliseconds; *P*=.06) compared with those without prepotentials. A strong and positive correlation was found between V-H interval and QRS duration during arrhythmia in those with prepotentials (B=2.11, R2=0.97, t =13.7, *P*<.001), but not in those without.	Local EGM characteristics and relative activation time of the HB suggest the possibility of conduction tissue as the origin for VA arising from the fibrous AMC. Specific identification and targeting of PPs when ablating VAs at this location might improve procedural success.	

**Study name or acronym; Author; Year published; PMID**	**Aim of study; Endpoints; Study type (RCT, observational — multicenter or single center; case series or other [if RCT include intervention and comparator]); Study size (N)**	**Patient population with inclusion and exclusion criteria**	**Results (absolute event rates, *P* values; OR or RR; 95% CI)**	**Other relevant findings or adverse events**	**Limitations; Other comments; Conclusions**
Chen et al. Ventricular arrhythmias originating from the aortomitral continuity: an uncommon variant of left ventricular outflow tract tachycardia.<br>• Year published: 2012<br>• PMID: 21979993	Study type: Single-center observational<br>Size: 10 patients	Inclusion criteria: All patients with VAs arising from the aortomitral continuity undergoing catheter ablation.<br><br>Exclusion criteria: N/A	Results: In 4 patients with anterior AMC location, early RS wave transition was found in the precordial leads, with equal R and S amplitudes in V2, rS in V1, and R in V3. In 6 patients whose VT arose from the middle part of the AMC, we demonstrated a special ("rebound") transition pattern, with which equal R and S amplitudes occurred in V2, and high R waves in V1 and V3. In the anterior AMC location, the S/R ratios in leads V1 and V2 were >1 and statistically significantly higher than those located in the middle (V1: 1.59 vs 0.23, *P*<.001; V2: 1.52 vs 0.41, *P*<.01).	The RS pattern in the precordial leads might predict an anterior versus a middle site of origin for AMC VAs.	

Study name or acronym; Author; Year published; PMID	Aim of study; Endpoints; Study type (RCT, observational — multicenter or single center; case series or other [if RCT include intervention and comparator]); Study size (N)	Patient population with inclusion and exclusion criteria	Results (absolute event rates, *P* values; OR or RR; 95% CI)	Other relevant findings or adverse events	Limitations; Other comments; Conclusions
**Nonrandomized Trials, Observational Studies, and/or Registries of Catheter Ablation in Papillary Muscle VAs**					
Doppalapudi et al. Ventricular tachycardia originating from the posterior papillary muscle in the left ventricle: a distinct clinical syndrome.<br>• Year published: 2008<br>• PMID: 19808390	Study type: Single-site observational<br>Size: 7 patients	Inclusion criteria: VT mapped to the posterior papillary muscle of the LV<br><br>Exclusion criteria: None	Of 290 patients with idiopathic VAs, 7 were found to have origin in the posteromedial PAP. All patients had RBBB and superior QRS axis. No patient had SHD. VT had a focal mechanism, sensitive to catecholamines.<br>Results: Successful catheter ablation in all patients without complications.	Posteromedial papillary muscle VT is catecholamine-sensitive, with a focal mechanism that is amendable to catheter ablation. Catheter stability might be difficult, and multiple RF applications are usually required.	
Yamada et al. Electrocardiographic and electrophysiological characteristics in idiopathic ventricular arrhythmias originating from the papillary muscles in the left ventricle: relevance for catheter ablation.<br>• Year published: 2010<br>• PMID: 20558848	Study type: Single-site observational<br>Size: 19 patients	Inclusion criteria: VT mapped to the posteromedial or anterolateral PAPs of the LV<br><br>Exclusion criteria: None	Of 159 consecutive patients with idiopathic VAs mapped to the LV, the site of origin was in the posteromedial PAP in 12 and in the anterolateral PAP in 7.<br>Results: Successful ablation was achieved in 19 of 19 patients. Multiple QRS morphologies were observed in 47% of patients, and ablation on both sides of the PAP was required for 7 patients. No complications were observed. Recurrence of PAP VAs was observed in 2 of 19 patients.	VT of focal origin can occur in either the posteromedial or the anterolateral PAPs of the LV. Catheter ablation often requires multiple RF applications over a wide area, suggesting an origin deep within the PAP. The recurrence risk after initially successful ablation is higher than for many other forms of idiopathic VT.	

Study name or acronym; Author; Year published; PMID	Aim of study; Endpoints; Study type (RCT, observational — multicenter or single center; case series or other [if RCT include intervention and comparator]); Study size (N)	Patient population with inclusion and exclusion criteria	Results (absolute event rates, *P* values; OR or RR; 95% CI)	Other relevant findings or adverse events	Limitations; Other comments; Conclusions
Yokokawa et al. Predictors of successful catheter ablation of ventricular arrhythmias arising from the papillary muscles.<br>• Year published: 2010<br>• PMID: 20637311	Study type: Single-site observational<br>Size: 40 patients	Inclusion criteria: VT mapped to the posteromedial or anterolateral PAPs of the LV<br><br>Exclusion criteria: None	Results: Forty consecutive patients were referred for ablation of symptomatic PVCs (N=19) or VT (N=21) originating from a PAP in the LV (N=32) or RV (N=8). AADs failed to control the VAs in 24 patients. Some 20 of 40 patients (50%) had SHD: prior MI in 10 patients, dilated cardiomyopathy in 9, and valvular heart disease in 1.<br><br>Catheter ablation was acutely successful in 33 of 40 patients (83%).<br><br>Pleomorphic QRS morphologies were observed in 31 of 40 patients. By MRI, the mass of the arrhythmogenic PAP was greater in patients with failed than successful ablations. In follow-up, the PVC burden was reduced from 15% ± 11% to 3% ± 3%; *P*<.01) after successful ablation.	VAs can originate in the papillary muscles of both the LV and the RV. PVCs from the PAPs are often pleomorphic. Catheter ablation is successful in over 80% of cases, with greater mass of the PAP predicting lower efficacy of ablation.	

Study name or acronym; Author; Year published; PMID	Aim of study; Endpoints; Study type (RCT, observational — multicenter or single center; case series or other [if RCT include intervention and comparator]); Study size (N)	Patient population with inclusion and exclusion criteria	Results (absolute event rates, *P* values; OR or RR; 95% CI)	Other relevant findings or adverse events	Limitations; Other comments; Conclusions
Ban et al. Electrophysiological characteristics related to outcome after catheter ablation of idiopathic ventricular arrhythmia originating from the papillary muscle in the left ventricle.<br>• Year published: 2013<br>• PMID: 24385992	Study type: Single-site observational<br>Size: 12 patients	Inclusion criteria: Of 284 patients with idiopathic VAs undergoing ablation, 12 patients were identified with VAs originating from the PAPs of the LV.	Results: Successful catheter ablation was achieved in 7 of 8 (87.5%) patients with high amplitude electrograms at the earliest site of origin. The 4 patients with low amplitude and fractionated electrograms had VA recurrences after ablation.<br><br>The mean duration from onset to peak downstroke (Δt) on the unipolar electrogram was significantly longer in the successful group than in the recurrence group (58 ± 8 ms vs 37 ± 9 ms, *P*=.04). A slow downstroke >50 ms from the initial Q wave on the unipolar electrogram at ablation sites was also significantly associated with successful outcome (85.7% vs 25.0%, *P*=.03).	In PAP VT, a high-amplitude, discrete potential before the QRS and slow down-stroke of the initial Q wave on the unipolar electrogram at ablation sites are related to favorable outcome after RF catheter ablation.	

Study name or acronym; Author; Year published; PMID	Aim of study; Endpoints; Study type (RCT, observational — multicenter or single center; case series or other [if RCT include intervention and comparator]); Study size (N)	Patient population with inclusion and exclusion criteria	Results (absolute event rates, *P* values; OR or RR; 95% CI)	Other relevant findings or adverse events	Limitations; Other comments; Conclusions
Yamada et al. Idiopathic focal ventricular arrhythmias originating from the anterior papillary muscle in the left ventricle.<br>• Year published: 2009<br>• PMID: 19298560	Study type: Single-site observational<br>Size: 6 patients	Inclusion criteria: Of 432 patients with idiopathic VAs undergoing ablation, 6 patients were identified with VAs originating from the APM of the LV.	Results: Six patients were identified with VT (n=1) or PVCs (n=5), with the earliest site of ventricular activation localized to the base (n=3) or middle portion (n=3) of the LV APM. No Purkinje potentials were recorded at the ablation site during sinus rhythm or the VAs. All patients had a normal baseline ECG and normal LV systolic function. The VAs exhibited an RBBB and right inferior axis QRS morphology in all patients. Oral verapamil and/or Na+ channel blockers failed to control the VAs in 4 patients. VT was not inducible by programmed electrical stimulation in any patient. In 4 patients, RF current with an irrigated or conventional 8-mm-tip ablation catheter was required to achieve a lasting success. Two patients had recurrent PVCs after a conventional RFCA with a 4-mm-tip ablation catheter had initially suppressed the arrhythmia.	VAs can arise from the base or middle portion of the APM and are characterized by an RBBB and right inferior access QRS morphology and focal mechanism. Catheter ablation of APM VAs is typically challenging, and creation of a deep RF lesion might be necessary for long-term success.	

Study name or acronym; Author; Year published; PMID	Aim of study; Endpoints; Study type (RCT, observational — multicenter or single center; case series or other [if RCT include intervention and comparator]); Study size (N)	Patient population with inclusion and exclusion criteria	Results (absolute event rates, *P* values; OR or RR; 95% CI)	Other relevant findings or adverse events	Limitations; Other comments; Conclusions
Proietti et al. Intracardiac echo-facilitated 3D electroanatomical mapping of ventricular arrhythmias from the papillary muscles: assessing the 'fourth dimension' during ablation.<br>• Year published: 2017<br>• PMID: 27485578	Study type: Single-site observational<br>Size: 16 patients	Inclusion criteria: Sixteen patients who underwent catheter ablation for VT or symptomatic PVCs originating from LV PAPs	Results: A total of 24 procedures (mean 1.5 per patient) were performed: 15 using a retrograde aortic approach and 9 using a transseptal approach. Integrated intracardiac ultrasound for 3D electroanatomical mapping was used in 15 of the 24 procedures. The posteromedial PAP was the most frequent culprit for the clinical arrhythmia, and the body was the part of the PAP most likely to be the successful site for ablation. The ablation site was identified based on the best pace map matching the clinical arrhythmia and the site of earliest activation. At a mean follow-up of 10.5 ± 7 months, only 2 patients had recurrent arrhythmias following a repeat ablation procedure.	An echo-facilitated 3D electroanatomical mapping allows for real-time creation of precise geometries of cardiac chambers and endocavitary structures. This is useful during catheter ablation of VAs originating from PAPs.	

Study name or acronym; Author; Year published; PMID	Aim of study; Endpoints; Study type (RCT, observational — multicenter or single center; case series or other [if RCT include intervention and comparator]); Study size (N)	Patient population with inclusion and exclusion criteria	Results (absolute event rates, *P* values; OR or RR; 95% CI)	Other relevant findings or adverse events	Limitations; Other comments; Conclusions
Rivera et al. Results of cryoenergy and radiofrequency-based catheter ablation for treating ventricular arrhythmias arising from the papillary muscles of the left ventricle, guided by intracardiac echocardiography and image integration.<br>• Year published: 2016<br>• PMID: 27069089	Study type: Single-site observational<br>Size: 21 patients	Inclusion criteria: Sixteen patients who underwent RFCA or cryoablation for VT or symptomatic PVCs originating from LV PAPs	Results: VAs were localized using 3D mapping, multidetector CT, and intracardiac echocardiography, with arrhythmia foci being mapped at either the anterolateral PAP or the posteromedial PAP of the left ventricle. Focal ablation was performed using an 8-mm cryoablation catheter or a 4-mm open-irrigated RF catheter, via transmitral approach. Acute success rate was 100% for cryoenergy (n=12) and 78% for RF (n=9; *P*=.08). Catheter stability was achieved in all (100%) patients treated with cryoenergy, and only in 2 (25%) patients treated with RF (*P*=.001). Incidence of multiple VA morphologies was observed in 7 patients treated with RF (77.7%), whereas none was observed in those treated with cryoenergy (*P*=.001). VA recurrence at the 6-month follow-up was 0% for cryoablation and 44% for RF (*P*=.03).	Cryoablation was associated with higher success rates and lower recurrence rates than radiofrequency catheter ablation, better catheter stability, and lesser incidence of polymorphic arrhythmias.	

Study name or acronym; Author; Year published; PMID	Aim of study; Endpoints; Study type (RCT, observational — multicenter or single center; case series or other [if RCT include intervention and comparator]); Study size (N)	Patient population with inclusion and exclusion criteria	Results (absolute event rates, *P* values; OR or RR; 95% CI)	Other relevant findings or adverse events	Limitations; Other comments; Conclusions
Wo et al. Circumferential ablation at the base of the left ventricular papillary muscles: a highly effective approach for ventricular arrhythmias originating from the papillary muscles.<br>• Year published: 2016<br>• PMID: 27400187	Study type: Single-site observational<br>Size: 16 patients	Inclusion criteria: 16 consecutive patients who received RFCA for VAs from LV PAPs	Results: RFCA was performed first at the earliest activation site or at the best pace map site. The ablation energy was delivered continuously for 60–120 s. Additional ablation was then circumferentially delivered at the base of the PAPs.<br><br>RFCA was successfully performed in all 16 patients, with no cases of VA recurrence after a mean follow-up of 20 ± 12 months. VAs originated from the anterior (n=8) and posterior (n=8) PAPs. Purkinje potentials were identified at the target sites in seven patients. All VAs were temporarily suppressed by one to two long-duration applications of RFCA at the initial targeted site, but recurrence was subsequently noted. In 6 patients, the QRS morphologies of the VAs changed after the initial RFCA. A subsequent circumferential ablation approach around the base of the PAPs completely eliminated all VAs.	Circumferential RFCA at the base of the PAPs leads to a high success rate for VAs from LV PAPs.	

Study name or acronym; Author; Year published; PMID	Aim of study; Endpoints; Study type (RCT, observational — multicenter or single center; case series or other [if RCT include intervention and comparator]); Study size (N)	Patient population with inclusion and exclusion criteria	Results (absolute event rates, *P* values; OR or RR; 95% CI)	Other relevant findings or adverse events	Limitations; Other comments; Conclusions
Itoh and Yamada. Usefulness of pace mapping in catheter ablation of left ventricular papillary muscle ventricular arrhythmias with a preferential conduction.<br>• Year published: 2018<br>• PMID: 29537721	Study type: Single-site observational<br>Size: 34 patients	Inclusion criteria: 34 consecutive patients undergoing RFCA of 40 LVPM VAs.	Results: Among 78 QRS morphologies, pace mapping was performed for 67 QRS morphologies during 37 VAs and revealed VA-MPMs with a latency for 14 QRS morphologies during 11 VAs (30%). Of 47 QRS morphologies with activation mapping, RFCA at the earliest activation site was successful in 39, but not successful in 8, despite MPMs with no latency. In these cases, RFCA was successful at remote sites of the MPMs with latency (n=6) and a site located between the earliest activation site and a site with latency (n=1). Among the remaining 31 QRS morphologies with pace mapping only, RFCA was successful at MPM sites with no latency in 17, and at MPM sites with latency in 7. In 3 of those 7 QRS morphologies, MPMs were recorded at multiple remote sites, and RFCA was not successful at MPM sites with no latency (n=2) or a shorter latency (n=1).	When an MPM with latency was recorded in LVPM VAs, RFCA at that site was highly successful. Attention should be paid to latency as well as the score during pace mapping of LVPM VAs.	

Study name or acronym; Author; Year published; PMID	Aim of study; Endpoints; Study type (RCT, observational — multicenter or single center; case series or other [if RCT include intervention and comparator]); Study size (N)	Patient population with inclusion and exclusion criteria	Results (absolute event rates, *P* values; OR or RR; 95% CI)	Other relevant findings or adverse events	Limitations; Other comments; Conclusions
Bassil et al.<br>Comparison of robotic magnetic navigation-guided and manual catheter ablation of ventricular arrhythmias arising from the papillary muscles.<br>• Year published: 2018<br>• PMID: 29722854	Study type: Single-site observational<br>Size: 35 patients	Inclusion criteria: 35 consecutive patients undergoing RFCA of LV and RV papillary muscle VAs. Remote magnetic guidance was compared with manual catheter guidance.	Results: Catheter ablation was initially performed using RMN guidance in 24 (69%) patients and manual guidance in 11 (31%) patients. The VA sites of origin were mapped to 20 (53%) anterolateral LV papillary muscles, 14 (37%) posteromedial LV papillary muscles, and 4 (11%) RV papillary muscles. Acute successful ablation was achieved for 20 (74%) VAs using RMN-guided ablation and 8 (73%) VAs using manual ablation (*P*=1.000). Fluoroscopy times were significantly lower among patients undergoing RMN ablation compared with patients undergoing manual ablation [median 7.3 (IQR 3.9–18) vs 24 (IQR 16–44) minutes; *P*=.005]. No procedural complications were observed in the study patients.	Use of an RMN-guided approach to target papillary muscle VAs results in comparable success rates observed with manual ablation, but with lower fluoroscopy times and decreased use of transaortic retrograde access.	

Study name or acronym; Author; Year published; PMID	Aim of study; Endpoints; Study type (RCT, observational — multicenter or single center; case series or other [if RCT include intervention and comparator]); Study size (N)	Patient population with inclusion and exclusion criteria	Results (absolute event rates, *P* values; OR or RR; 95% CI)	Other relevant findings or adverse events	Limitations; Other comments; Conclusions
Lee et al. Catheter ablation of papillary muscle arrhythmias: Implications of mitral valve prolapse and systolic dysfunction.<br>• Year published: 2018<br>• PMID: 29732567	Study type: Single-site observational<br>Size: 23 patients	Inclusion criteria: 23 consecutive patients undergoing RFCA of LV papillary muscle VAs. Echocardiography was used to assess for MVP.	Results: Of 23 patients with LV PAP VAs, 9 (39%) had MVP compared with none of the 129 (0%) patients with idiopathic VAs undergoing catheter ablation at other sites (*P*<.001). Acute procedural success was achieved in 60% and 80% of those with and without MVP, respectively (*P*=.28). Medium-term outcomes were comparable (*P*=.75). In patients with cardiomyopathy, the median LVEF improved from 40% to 54% following ablation (*P*=.007).	Although MVP is strongly associated with LV papillary muscle VAs, MVP does not adversely affect the acute or medium-term outcomes of ablation.	Systolic function can improve following ablation in patients with ectopy-mediated cardiomyopathy due to papillary muscle VAs.

Study name or acronym; Author; Year published; PMID	Aim of study; Endpoints; Study type (RCT, observational — multicenter or single center; case series or other [if RCT include intervention and comparator]); Study size (N)	Patient population with inclusion and exclusion criteria	Results (absolute event rates, *P* values; OR or RR; 95% CI)	Other relevant findings or adverse events	Limitations; Other comments; Conclusions
Yamada et al. Challenging radiofrequency catheter ablation of idiopathic ventricular arrhythmias originating from the left ventricular summit near the left main coronary artery.<br>• Year published: 2016<br>• PMID: 27729345	Study type: Single-center observational<br>Size: 45 patients	A total of 45 consecutive patients with VAs arising from the LV summit undergoing mapping with intention to perform catheter ablation.	RFCA was successful within the main trunk of the GCV in 16 patients and within a branch of the GCV traversing the basal LV summit in 7. Transpericardial RFCA was successful on the epicardial surface in the apical LV summit in 6 patients and was abandoned in 14 patients with basal LV summit VAs because of the close proximity to the coronary arteries and thick fat pads. RFCA was successful at the aortomitral continuity in 3 patients (2 with a failed transpericardial RFCA), and at the LCC in 1. The RFCA success rate of the apical LV summit VAs including the GCV VAs was 100% (22 of 22), whereas that of the basal LV summit VAs was 48% (11 of 23).	The basal LV summit VAs could be differentiated from the apical LV summit VAs by LBBB pattern, QRS duration ≤175 ms, precordial transition ≥V1, and maximum deflection index of ≥0.55.	This study revealed that ≈50% of the basal LV summit VAs could be eliminated by a direct approach through a GCV branch running below the proximal left coronary arteries and a remote approach from the adjacent endocardial sites.

Study name or acronym; Author; Year published; PMID	Aim of study; Endpoints; Study type (RCT, observational — multicenter or single center; case series or other [if RCT include intervention and comparator]); Study size (N)	Patient population with inclusion and exclusion criteria	Results (absolute event rates, *P* values; OR or RR; 95% CI)	Other relevant findings or adverse events	Limitations; Other comments; Conclusions
Zhang et al. Common and distinctive electrocardiographic characteristics and effective catheter ablation of idiopathic ventricular arrhythmias originating from different areas of ventricular septum adjacent to atrioventricular annulus.<br>• Year published: 2018<br>• PMID: 29782689	Study type: Single-center observational<br>Size: 106 patients	Of 1505 consecutive patients with PVC or IVT, 106 (7.04%) were confirmed as originating from the ventricular septum adjacent to the AV valve annulae.	The overall success rate for RFCA of PVCs or IVTs originating from the ventricular septum AVA was 82.08% (87 of 106). Tricuspid annular VAs were found in 87 patients and mitral annular VAs were found in 19 patients. Ablation was unsuccessful in 16 TA cases and 3 MA cases due to HB potentials at the site of earliest activation.	During ablation, accelerated junctional tachycardia developed in 44 patients, with transient third-degree AVB in 6, and 2:1 AVB in 1. One patient with sustained VT developed persistent third-degree AVB after successful ablation at a site of earliest ventricular activation 5 mm from the HB, and VVI pacemaker was implanted.	VAs can originate from the interventricular septum along either the tricuspid or mitral annulus. Catheter ablation might be limited by proximity to the AV conduction system.
Al'Aref et al. Differentiation of papillary muscle from fascicular and mitral annular ventricular arrhythmias in patients with and without structural heart disease.<br>• Year published: 2015<br>• PMID: 25925230	Study type: Single-center observational<br>Size: 51 patients	52 VAs in 51 patients including papillary muscle (n=18), fascicular (n=15), and mitral annular (n=19) origins	Papillary muscle VAs were distinguished electrocardiographically from fascicular VAs by longer QRS duration and lower prevalence of r<R' V1 QRS morphology, and from mitral annular VAs by lower prevalence of positive precordial lead concordance. Using a stepwise electrocardiographic algorithm, the accuracy rates for the diagnosis of papillary muscle VAs, fascicular VAs, and mitral annular VAs were 83%, 87%, and 89%, respectively.	Specific electrocardiographic characteristics, including QRS morphology and precordial lead morphology, can help distinguish between papillary muscle, fascicular, and mitral annular VAs.	

Study name or acronym; Author; Year published; PMID	Aim of study; Endpoints; Study type (RCT, observational — multicenter or single center; case series or other [if RCT include intervention and comparator]); Study size (N)	Patient population with inclusion and exclusion criteria	Results (absolute event rates, *P* values; OR or RR; 95% CI)	Other relevant findings or adverse events	Limitations; Other comments; Conclusions
Yue-Chun et al. Catheter ablation of idiopathic premature ventricular contractions and ventricular tachycardias originating from the vicinity of endocardial and epicardial mitral annulus.<br>• Year published: 2013<br>• PMID: 24312241	Study type: Single-center observational<br>Size: 21 patients	Of 597 consecutive patients with idiopathic VAs undergoing catheter ablation, 21 had VA origin at the MA.	Eleven (52%) from the ENDO MA, and 10 (48%) from the EPI MA. Different ECG characteristics of PVCs and VTs originated from the ENDO and EPI MA. The prolonged pseudodelta wave time and intrinsicoid deflection time in lead V2 and the precordial maximum deflection index reliably differentiated EPI MA VAs from ENDO MA VAs with high sensitivity and specificity. Successful acute RFCA was achieved in 18 (85.7%) patients.	ECG characteristics of PVCs and VTs originating from the different portions of the MA are different and can help regionalize the origin of these arrhythmias. RFCA within the coronary venous system was relatively effective and safe for the PVCs/IVTs and should be considered an alternative approach when MA PVCs/IVTs cannot be eliminated by RFCA from the endocardium.	
Wasmer et al. Ventricular arrhythmias from the mitral annulus: patient characteristics, electrophysiological findings, ablation, and prognosis.<br>• Year published: 2013<br>• PMID: 23416375	Study type: Single-center observational<br>Size: 22 patients	Among consecutive patients with idiopathic VAs undergoing catheter ablation, 22 had VA origin at the mitral valve annulus.	Sites of origin were distributed around the MA with no preferential area. Ablation was successful in 13 of 16 (81%) patients. One 28-year-old woman patient with normal MRI and no SHD died suddenly 3 months after ablation.	One patient with no structural heart disease died suddenly 3 months after ablation.	

Study name or acronym; Author; Year published; PMID	Aim of study; Endpoints; Study type (RCT, observational — multicenter or single center; case series or other [if RCT include intervention and comparator]); Study size (N)	Patient population with inclusion and exclusion criteria	Results (absolute event rates, *P* values; OR or RR; 95% CI)	Other relevant findings or adverse events	Limitations; Other comments; Conclusions
Kumagai et al. Idiopathic left ventricular tachycardia originating from the mitral annulus.<br>• Year published: 2005<br>• PMID: 16191111	Study type: Single-center observational<br>Size: 35 patients	MA VT was identified in 35 of 72 patients with consecutive LV RMVTs from May 2000 to June 2004. All patients underwent an EP study and RFCA.	The sites of origin of the MA VT were grouped into 4 groups according to the successful ablation sites around the MA. Group I included the anterior sites (n=11), group II the anterolateral sites (n=9), group III the lateral sites (n=6), and group IV the posterior sites (n=9). RFCA successfully eliminated the MA VT in all patients with 1 ± 0.5 RF applications.	There were no complications; however, 3 patients (8%) had recurrence over a mean follow-up period of 24 months.	
Yui et al. Electrophysiological characteristics and radiofrequency catheter ablation treatment of idiopathic ventricular arrhythmias successfully ablated from the ostium of the coronary sinus.<br>• Year published: 2017<br>• PMID: 28652528	Study type: Single-center observational<br>Size: 6 patients	Six patients with VAs with origin at the coronary sinus ostium undergoing catheter ablation	Of 309 patients with idiopathic VAs treated with RFCA, 6 (1.94%; 3 men; age: 66.3 ± 9.7 years) had VAs successfully ablated from the coronary sinus ostium.	There were no complications except for AF in 1 patient during RFCA. There were no recurrences.	

Study name or acronym; Author; Year published; PMID	Aim of study; Endpoints; Study type (RCT, observational — multicenter or single center; case series or other [if RCT include intervention and comparator]); Study size (N)	Patient population with inclusion and exclusion criteria	Results (absolute event rates, *P* values; OR or RR; 95% CI)	Other relevant findings or adverse events	Limitations; Other comments; Conclusions
Baman et al. Mapping and ablation of epicardial idiopathic ventricular arrhythmias from within the coronary venous system.<br>• Year published: 2010<br>• PMID: 20400776	Study type: Single-center observational<br>Size: 27 patients	In 27 of 189 patients (14% +/- 5%; 95% CI), the SOO of the ventricular arrhythmia was identified from within the coronary venous system, either in the GCV (n=26) or the MCV (n=1).	Two patients had recurrent PVCs within 2 weeks after ablation, and no recurrences occurred in the remaining patients during a median follow-up of 13 months (range, 25). In the 7 patients with unsuccessful ablation, failure was due to the inability of the ablation catheter to be advanced to the SOO within the GCV (n=4), inadequate power delivery at the SOO (n=1), proximity to the phrenic nerve (n=1), or proximity of the SOO to a major coronary artery (n=1). Transcutaneous epicardial ablation was effective in 1 of 2 patients in whom it was attempted.	Almost 15% of IVAs have an epicardial origin. ECG characteristics help to differentiate epicardial arrhythmias from endocardial VAs. Epicardial arrhythmias can be ablated from within the CVS in approximately 70% of patients.	

Study name or acronym; Author; Year published; PMID	Aim of study; Endpoints; Study type (RCT, observational — multicenter or single center; case series or other [if RCT include intervention and comparator]); Study size (N)	Patient population with inclusion and exclusion criteria	Results (absolute event rates, *P* values; OR or RR; 95% CI)	Other relevant findings or adverse events	Limitations; Other comments; Conclusions
Kawamura et al. Idiopathic ventricular arrhythmia originating from the cardiac crux or inferior septum: epicardial idiopathic ventricular arrhythmia.<br>• Year published: 2014<br>• PMID: 25225238	Study type: Single-center observational<br>Size: 18 patients	A total of 18 pts with VAs originating at the crux of the heart undergoing attempted catheter ablation. The SOO was at the apical crux in 9 patients and at the basal crux in the other 9.	Fifteen (83%) patients with crux VA had sustained VT, and 3 patients required ICD implantation because of syncope. All patients had a left superior axis, and 16 patients had an R>S wave in V2. In apical crux VA, all patients had a deep S wave in V6, and 8 (89%) patients had an R>S wave in aVR. All apical crux patients underwent attempted ablation in the MCV without success. In 4 of these patients, epicardial ablation with subxiphoid approach was performed successfully. All basal crux VA patients had either negative or isoelectric pattern in V1 and had R>S in V6. Patients had successful ablation within the MCV.	Apical vs basal crux VA is identified as a new category of IVA with distinctive electrocardiographic characteristics; ablation via the MCV is effective for eliminating basal crux VA, whereas apical crux VA often requires a subxiphoid epicardial approach.	

Study name or acronym; Author; Year published; PMID	Aim of study; Endpoints; Study type (RCT, observational — multicenter or single center; case series or other [if RCT include intervention and comparator]); Study size (N)	Patient population with inclusion and exclusion criteria	Results (absolute event rates, *P* values; OR or RR; 95% CI)	Other relevant findings or adverse events	Limitations; Other comments; Conclusions
Wu et al. Radiofrequency catheter ablation of idiopathic ventricular tachycardia and symptomatic premature ventricular contraction originating from valve annulus.<br>• Year published: 2008<br>• PMID: 19080325	Study type: Single-site observational<br>Size: 16 patients	A total of 16 consecutive patients with PVCs originating from the MVA (n=12) or TVA (n=4).	The MVA site was the anterolateral annulus in 6 patients, the lateral annulus in 6 patients, and the inferoposterior annulus in 2 patients. There were 4 sites in the Tricuspid annulus. Successful ablation was achieved in 15 of 16 patients, with 2 recurrences that were later successfully ablated.	No complications were reported.	Small study and electroanatomic mapping used in only 2 patients.

Premature Ventricular Complexes With or Without Left Ventricle Dysfunction

Study name or acronym; Author; Year published; PMID	Aim of study; Endpoints; Study type (RCT, observational — multicenter or single center; case series or other [if RCT include intervention and comparator]); Study size (N)	Patient population with inclusion and exclusion criteria	Results (absolute event rates, *P* values; OR or RR; 95% CI)	Other relevant findings or adverse events	Limitations; Other comments; Conclusions
Ling et al. Radiofrequency ablation versus antiarrhythmic medication for treatment of ventricular premature beats from the right ventricular outflow tract: prospective randomized study.<br>• Year published: 2014<br>• PMID: 24523413	Aim: To compare efficacy of ablation to AADs for treatment of RVOT PVCs<br>Endpoints: Recurrence of RVOT PVCs<br>Study type: Prospective, RCT, single center<br>Size: 330 patients, 165 randomized to ablation, 165 randomized to AADs	Inclusion criteria: VPBs from RVOT by 12-lead ECG (inferior axis and LBBB), >6000 VPBs per 24 hours.<br>Exclusion criteria: Non-RVOT origin, previous AAD therapy, evidence of SHD, hyperthyroidism, or electrolyte disturbance, drug toxicity, diabetes, blood pressure >165/100 mm HG, significant impairment of renal function, QT >450 ms without BBB, significant AV conduction disease, and left or right BBB.	Recurrence rate with ablation vs AADs was 19.4% vs 88.6%, respectively (*P*<.001).	QS morphology in lead I was associated with improved outcome after ablation.	Single center, only 1-year follow-up, AADs limited to metoprolol and propafenone.
Latchamsetty et al. Multicenter outcomes for catheter ablation of idiopathic premature ventricular complexes.<br>• Year published: 2015<br>• PMID: 29759353	Aim: To quantify efficacy of ablation of idiopathic PVCs from multiple locations and evaluate change in EF in patients with PVC-induced cardiomyopathy<br>Endpoints: 80% reduction in PVC burden, EF in patients with cardiomyopathy<br>Study type: Multicenter, retrospective<br>Size: 1185 patients, 245 patients with PVC cardiomyopathy	Inclusion criteria: Patients undergoing ablation for idiopathic PVCs<br>Exclusion criteria: Prior MI, presence of scar by MRI	Acute success in 84%, long-term success without AADs was 71%, long-term success including AADs was 85%.<br><br>In 245 patients with cardiomyopathy, mean EF improved from 38% to 50% (*P*<.01).Of these patients, 85% experienced some improvement in EF.	Predictors of acute success: RVOT location (+), fewer PVC foci (+), epicardial location (-)<br>Predictors of long-term success: RVOT location<br><br>Predictors of cardiomyopathy: PVC burden, asymptomatic, epicardial origin, male sex<br><br>There were 5.2% overall complications, 2.4% major complications, and no mortality noted.	Retrospective study

Study name or acronym; Author; Year published; PMID	Aim of study; Endpoints; Study type (RCT, observational — multicenter or single center; case series or other [if RCT include intervention and comparator]); Study size (N)	Patient population with inclusion and exclusion criteria	Results (absolute event rates, *P* values; OR or RR; 95% CI)	Other relevant findings or adverse events	Limitations; Other comments; Conclusions
Yamada et al. Efficacy of electroanatomic mapping in the catheter ablation of premature ventricular contractions originating from the right ventricular outflow tract.<br>• Year published: 2007<br>• PMID: 17891452	Aim: To compare eletroanatomic mapping to conventional mapping for ablation of RVOT PVCs<br>Endpoints: Successful ablation, procedure time, fluoroscopy time<br>Study type: Prospective, RCT, single center<br>Size: 100 patients (50 randomized to eletroanatomic mapping and 50 randomized to conventional mapping)	Inclusion criteria: Symptomatic and drug-refractory PVCs, RVOT origin, >10,000 PVCs per day<br><br>Exclusion criteria: 3 or more beats NSVT, SHD by echo	Successful ablation at follow-up was similar with or without eletroanatomic mapping (96% vs 88%, *P*=.14).<br>Fluoroscopy times and RF times were significantly shorter with eletroanatomic mapping (*P*<.0001)	Overall ablation success rates were high, complications were reported as none, and mapping did not improve efficacy but decreased fluoroscopy and procedure time.	Single center, relatively small sample
Wang et al. Voltage combined with pace mapping is simple and effective for ablation of noninducible premature ventricular contractions originating from the right ventricular outflow tract.<br>• Year published: 2016<br>• PMID: 28026917	Aim: To describe outcomes for ablation of noninducible PVCs using voltage and pace mapping<br>Endpoints: Successful ablation, complications<br>Study type: Retrospective, single center<br>Size: 148 patients (21 noninducible)	Inclusion criteria: Recurrent symptomatic PVCs, RVOT origin by ECG, drug-refractory<br><br>Exclusion criteria: SHD by echo and angiogram, ablation attempted in the LVOT/aortic cusp/epicardium	Successful ablation (total 94%) and PVC burden following ablation (0.55% vs 0.40%, *P*=.210) were similar between the noninducible and inducible groups. PVC recurrence was also similar (9.5% vs 5.5%, *P*=.826).	Complication rates were similar between the noninducible and inducible group (4.8% vs 3.9%, *P*=.922); these were all local vascular events.	Single center, small sample size

Study name or acronym; Author; Year published; PMID	Aim of study; Endpoints; Study type (RCT, observational — multicenter or single center; case series or other [if RCT include intervention and comparator]); Study size (N)	Patient population with inclusion and exclusion criteria	Results (absolute event rates, *P* values; OR or RR; 95% CI)	Other relevant findings or adverse events	Limitations; Other comments; Conclusions
Lamba et al. Radiofrequency catheter ablation for the treatment of idiopathic premature ventricular contractions originating from the right ventricular outflow tract: a systematic review and meta-analysis.<br>• Year published: 2014<br>• PMID: 23980900	Aim: To evaluate the efficacy of ablation of idiopathic RVOT PVCs<br>Endpoints: Change in PVC burden, change in LVEF<br>Study type: Meta-analysis<br>Size: 14 total studies, 6 studies (70 patients) to evaluate success of ablation and 5 studies (108 patients) to evaluate change in EF	Inclusion criteria: RFCA for frequent idiopathic PVCs from RVOT; reported pre- and postablation PVC burden, LVEF, LVEDD, LVESD<br><br>Exclusion criteria: Structural abnormalities (eg, MI), sustained VT, PVCs from other locations	The 24-hour PVC burden decreased by a mean of –30,089 (95% CI –31,658.47 to –28,520.40, *P*<.00001). LVEF improved by a mean of 10.36% (95% CI 8.75–11.97, *P*<.00001)		Significant heterogeneity between studies, studies varied significantly in follow-up times.
Zhong et al. Relative efficacy of catheter ablation vs antiarrhythmic drugs in treating premature ventricular contractions: a single-center retrospective study.<br>• Year published: 2014<br>• PMID: 24157533	Aim: Retrospective study comparing efficacy of catheter ablation with AADs in treating frequent PVCs<br>Endpoints: Decrease in PVC burden, change in EF<br>Study type: Retrospective, single center<br>Size: 510 patients (215 with RFCA, 295 with AADs)	Inclusion criteria: >1000 PVCs per 24 hours, baseline Holter and echo within 6 months of PVC diagnosis, follow-up Holter recordings, first time RFCA or first drug therapy in the respective groups	PVC frequency reduction greater with RFCA than AADs (–21,799 per 24 hours vs –8,376 per 24 hours; *P*<.001). LVEF was restored in 47% of the RFCA group with cardiomyopathy vs 21% of the AAD group (*P*<.001).	The RFCA group had more RVOT PVCs and the AAD group had more LV PVCs. Class I and III AADs were more effective (82%) than beta-blockers (36%, *P*<.001) and CCBs (43%, *P*<.001). Acute RFCA success was 94%, with 9.3% requiring repeat ablation and 6% needing AADs. Overall drug efficacy was 49%, with a 5.6% complication rate; all were managed successfully with no mortality in the RFCA group.	Single center, retrospective, selection bias between 2 groups.

Study name or acronym; Author; Year published; PMID	Aim of study; Endpoints; Study type (RCT, observational — multicenter or single center; case series or other [if RCT include intervention and comparator]); Study size (N)	Patient population with inclusion and exclusion criteria	Results (absolute event rates, *P* values; OR or RR; 95% CI)	Other relevant findings or adverse events	Limitations; Other comments; Conclusions
Singh et al. Amiodarone in patients with congestive heart failure and asymptomatic ventricular arrhythmia. Survival Trial of Antiarrhythmic Therapy in Congestive Heart Failure.<br>• Year published: 1995<br>• PMID: 7539890	Aim: To evaluate the effect on mortality of amiodarone in patients with CHF and asymptomatic VAs.<br>Endpoints: Overall mortality<br>Study type: Double blind, placebo controlled, RCT<br>Size: 24 centers, 674 patients (336 randomized to amiodarone, 338 randomized to placebo)	Inclusion criteria: CHF, at least 10 PVCs per hour, LV enlargement, EF 40% or less<br><br>Exclusion criteria: Women of childbearing age, MI within 3 months, symptomatic VA, history of SCD or sustained VT, uncontrolled thyroid disease, need for AADs, significant ECG changes of QRS interval or QTc interval, life threatening noncardiac disease, hypotension	No significant difference in mortality (survival 69.4% vs 70.8%; *P*=.6).<br>At 2 years, sudden death with amiodarone vs placebo was 15% vs 19%, respectively (*P*=.43).<br>Amiodarone had a trend toward reduction in overall mortality in those with NICM (*P*=.07) at 2 years, which does not appear to be the case at 30 months.	Amiodarone was more effective in suppressing VA and increased EF by 42% at 2 years. The drug was discontinued in 27% of the amiodarone group vs 23% of the placebo group due to intolerable adverse effects (*P*=.1)	>40% discontinued amiodarone or were lost to follow-up.
Mountantonakis et al. Reversal of outflow tract ventricular premature depolarization-induced cardiomyopathy with ablation: effect of residual arrhythmia burden and preexisting cardiomyopathy on outcome.<br>• Year published: 2011<br>• PMID: 21699837	Aim: To evaluate the effect of ablation of outflow tract PVCs in patients with PVC-induced cardiomyopathy<br>Endpoints: Successful ablation, change in EF, change in LVDD<br>Study type: Retrospective, single center<br>Size: 69 patients	Inclusion criteria: Frequent (>5,000 PVCs per 24 hours), LVCM (EF <50%), referred for catheter ablation, predominant outflow tract origin PVC (right or left bundle branch morphology, inferior axis, negative in aVL).<br><br>Exclusion criteria: Active ischemia, prior infarction	At 11 months after ablation: 44 (66%) had rare PVCs, 15 (22%) had >80% PVC reduction, and 8 (12%) had no improvement. After ablation, patients with rare PVCs, decreased PVCs (>80% reduction), and no change in PVC burden had an LVEF change of 14%, 13%, and –3%, respectively (*P*<.001). Changes in LVDD for the 3 groups were –4, –2, and 0, respectively (*P*=.38).	Magnitude of LVEF improvement correlated with decline in residual PVC burden (r=0.475, *P*=.007).<br><br>Predictors of LVEF improvement: ablation outcome, higher LVEF, absence of preexisting LVCM.	Single center, relatively small sample size

Study name or acronym; Author; Year published; PMID	Aim of study; Endpoints; Study type (RCT, observational — multicenter or single center; case series or other [if RCT include intervention and comparator]); Study size (N)	Patient population with inclusion and exclusion criteria	Results (absolute event rates, *P* values; OR or RR; 95% CI)	Other relevant findings or adverse events	Limitations; Other comments; Conclusions
Zang et al. Beneficial effects of catheter ablation of frequent premature ventricular complexes on left ventricular function.<br>• Year published: 2014<br>• PMID: 24670420	Aim: To evaluate the effect of ablation of frequent PVCs in patients with PVC-induced cardiomyopathy<br>Endpoints: Changes following ablation in LVEF and LVEDD<br>Study type: Meta-analysis<br>Size: 15 studies with 712 patients; 336 patients with baseline LV dysfunction	Inclusion criteria: Cohort studies for catheter ablation of frequent PVCs (sample size at least 10 patients). Echo parameters present at baseline and postablation.	Ablation success rate was 66%–90%.<br>Overall mean increase in LVEF was 7.7% (95% CI 6.1%–9.4%).<br>Overall mean decrease in LVEDD was -4.6 mm (95% CI -6.0 to -3.1 mm).<br>In patients with LV dysfunction LVEF increased 12.4% (95% CI 8.1%–16.6%).<br>Mean decrease in LVEDD was –4.8 mm (95% CI –6.2 to –3.4 mm).	Included both patients with idiopathic PVCs and patients with SHD.	Substantial heterogeneity existed
Lee et al. Ventricular ectopy in the context of left ventricular systolic dysfunction: risk factors and outcomes following catheter ablation.<br>• Year published: 2019<br>• PMID: 29482954	Aim: To evaluate risk factors for developing PVC-induced cardiomyopathy and outcomes following PVC ablation<br>Endpoints: EF, elimination of VA<br>Study type: Single center, retrospective<br>Size: 152 patients	Inclusion criteria: Patients undergoing ablation for idiopathic PVCs, EF <50%	Mean EF improved from 40% to 52% (*P*<.001).	85% of patients were free of VA at 7–12 months.<br>Predictors of cardiomyopathy in a single variate analysis were age, male sex, PVC burden, non-RVOT sites, PVC QRS duration, and PVC minimum coupling interval. Only male sex was predictive in a multivariate analysis.	Single center, retrospective

Study name or acronym; Author; Year published; PMID	Aim of study; Endpoints; Study type (RCT, observational — multicenter or single center; case series or other [if RCT include intervention and comparator]); Study size (N)	Patient population with inclusion and exclusion criteria	Results (absolute event rates, *P* values; OR or RR; 95% CI)	Other relevant findings or adverse events	Limitations; Other comments; Conclusions
Bogun et al. Radiofrequency ablation of frequent, idiopathic premature ventricular complexes: comparison with a control group without intervention.<br>• Year published: 2007<br>• PMID: 17599667	Aim: To compare the effects of ablation on patients with PVC-induced cardiomyopathy with a control group without ablation<br>Endpoints: PVC burden following ablation, EF following ablation<br>Study type: Single center, retrospective<br>Size: 60 patients who underwent ablation, 22 patients with cardiomyopathy	Inclusion criteria: Patients with frequent PVCs (>10 per hour) undergoing ablation, refractory to AADs, no SHD, ambulatory monitoring before and after ablation<br><br>Exclusion criteria: Coronary disease, valvular disease, CHD, LVH, RV dysfunction, sustained VT or frequent NSVT	Successful ablation in 18 patients with cardiomyopathy showed improvement in EF from 34% to 59% +/- 7% (*P*<.0001).	A control group of 11 patients with similar PVC burden and cardiomyopathy who did not undergo ablation had no change in EF; one underwent cardiac transplantation.<br><br>One patient who underwent ablation had complete heart block and required PPM.	Small study size, single center
Takemoto et al. Radiofrequency catheter ablation of premature ventricular complexes from right ventricular outflow tract improves left ventricular dilation and clinical status in patients without structural heart disease.<br>• Year published: 2005<br>• PMID: 15837259	Aim: To evaluate outcomes following ablation of PVCs from the RVOT<br>Endpoints: procedural success, effect on cardiac function<br>Study type: Single center, retrospective<br>Size: 40 patients	Inclusion criteria: Monomorphic PVCs from RVOT, no SHD<br><br>Exclusion criteria: VT, atrial tachyarrhythmias	Procedural success in 93%	Only the group with PVC burden >20% showed improved EF, decreased LVDD, decreased LV dilation, and decreased mitral regurgitation following successful ablation.	Small sample size, short follow-up period (6 months), limited to RVOT PVCs.

Study name or acronym; Author; Year published; PMID	Aim of study; Endpoints; Study type (RCT, observational — multicenter or single center; case series or other [if RCT include intervention and comparator]); Study size (N)	Patient population with inclusion and exclusion criteria	Results (absolute event rates, *P* values; OR or RR; 95% CI)	Other relevant findings or adverse events	Limitations; Other comments; Conclusions
Baman et al. Relationship between burden of premature ventricular complexes and left ventricular function.<br>• Year published: 2010<br>• PMID: 20348027	Aim: To correlate PVC burden with likelihood of developing PVC-induced cardiomyopathy.<br>Endpoints: To estimate a PVC burden most predictive of developing a cardiomyopathy<br>Study type: Single center, retrospective<br>Size: 57 patients with cardiomyopathy, 117 controls without cardiomyopathy	Inclusion criteria: Idiopathic frequent PVCs<br><br>Exclusion criteria: CAD	PVC burden >24% best separated patients with and without cardiomyopathy (sensitivity 79%, specificity 78%, AUC 0.89). Lowest PVC burden resulting in reversible cardiomyopathy was 10%.	PVC burden associated with cardiomyopathy (HR 1.12; 95% CI 1.08–1.16).	Single-center retrospective study, small size, only included patients referred for ablation
Yarlagadda et al. Reversal of cardiomyopathy in patients with repetitive monomorphic ventricular ectopy originating from the right ventricular outflow tract.<br>• Year published: 2005<br>• PMID: 16103234	Aim: To evaluate whether successful ablation of frequent RVOT PVCs can improve cardiac function in patients with cardiomyopathy<br>Endpoints: Successful ablation, EF<br>Study type: Single center, retrospective<br>Size: 27 patients, 8 with decreased LV function	Inclusion criteria: Monomorphic PVCs, left bundle inferior axis morphology<br><br>Exclusion criteria: Predominant runs of VT or history of sustained VT	Ablation was successful in 85%. In patients with cardiomyopathy, EF improved from 39% to 62%, *P*=.017.	Older age was a risk factor for cardiomyopathy; all 7 patients with successful ablation and CM had normalization of EF.	Small study size, retrospective observational study

Study name or acronym; Author; Year published; PMID	Aim of study; Endpoints; Study type (RCT, observational — multicenter or single center; case series or other [if RCT include intervention and comparator]); Study size (N)	Patient population with inclusion and exclusion criteria	Results (absolute event rates, *P* values; OR or RR; 95% CI)	Other relevant findings or adverse events	Limitations; Other comments; Conclusions
Wijnmaalen et al. Beneficial effects of catheter ablation on left ventricular and right ventricular function in patients with frequent premature ventricular contractions and preserved ejection fraction.<br>• Year published: 2010<br>• PMID: 20659945	Aim: To evaluate the benefit of ablation of frequent PVCs in patients with preserved LVEF on other parameters of cardiac function<br>Endpoints: LV volumes, LVEF, LV strain, RV strain<br>Study type: Single center, retrospective<br>Size: 49 patients, 34 of whom were successfully ablated; 25 additional healthy controls without SHD	Inclusion criteria: Frequent PVCs (>5%), symptomatic, preserved LVEF<br><br>Exclusion criteria: SHD	Following successful ablation, LV end systolic volume decreased (56 to 49 ml, *P*=.018), LV strain showed significant improvement in all 3 dimensions, and RV longitudinal strain improved. No significant changes were observed in LVEF and RVEF.	Compared with controls, patients with frequent PVCs had decreased strain at a median symptom duration of 13 months (LV volumes, RV dimensions, and LV/RV systolic function were normal).	Small study size, retrospective analysis, single center<br><br>Suggested that recent-onset, frequent PVCs can result in reversible biventricular dysfunction not detected by conventional echocardiographic parameters.
Sarrazin et al. Impact of radiofrequency ablation of frequent post-infarction premature ventricular complexes on left ventricular ejection fraction.<br>• Year published: 2009<br>• PMID: 19879531	Aim: To evaluate changes in LVEF following ablation of frequent PVCs in patients with a prior history of MI who were referred for ICD implantation<br>Endpoints: Change in PVC burden, change in EF<br>Study type: Single center, retrospective<br>Size: 15 patients who underwent ablation, 15 controls	Inclusion criteria: Patients referred for ICD, remote MI, frequent PVCs (>5%)	All 15 patients had successful ablation, with PVC burden decreasing from 22% to 2.6% (*P*<.001). LVEF increased from 38% to 51% (*P*=.0001). EF in the control group was unchanged (34% vs 33%; *P*=.6)	Mean LVEDD decreased following ablation (56 vs. 51 mm; *P*=.030); 5 patients in the ablation group required ICD (4 for inducible VT and 1 for persistent LV dysfunction).	Small study size, nonrandomized.<br><br>EF following successful ablation of frequent PVCs can improve even in post-MI patients with evidence of scar.

Study name or acronym; Author; Year published; PMID	Aim of study; Endpoints; Study type (RCT, observational — multicenter or single center; case series or other [if RCT include intervention and comparator]); Study size (N)	Patient population with inclusion and exclusion criteria	Results (absolute event rates, *P* values; OR or RR; 95% CI)	Other relevant findings or adverse events	Limitations; Other comments; Conclusions
El Kadri et al. Effect of ablation of frequent premature ventricular complexes on left ventricular function in patients with nonischemic cardiomyopathy.<br>• Year published: 2015<br>• PMID: 25527251	Aim: To evaluate the change in LVEF following ablation of frequent PVCs in patients with preexisting nonischemic cardiomyopathy<br>Endpoints: Procedural success, mean EF, PVC burden<br>Study type: Single center, retrospective<br>Size: 30 patients	Inclusion criteria: NICM (scar on MRI prior to ablation or cardiomyopathy prior to PVCs), PVC burden >5%<br><br>Exclusion criteria: CAD	Successful ablation achieved in 60% of patients. Following successful ablation, mean EF improved from 33.9% to 45.7% (*P*<.0001) and PVC burden decreased from 23.1% to 1.0% (*P*<.0001).	Mean EF did not change if ablation was unsuccessful (44.4 vs 43.5%; *P*=.85).	Small study size, retrospective analysis, single center<br><br>Demonstrated improvement (although not necessarily normalization) of EF in patients with pre-existing nonischemic cardiomyopathy following successful ablation of frequent PVCs.
Haïssaguerre et al. Mapping and ablation of idiopathic ventricular fibrillation.<br>• Year published: 2002<br>• PMID: 12186801	Aim: To assess the efficacy of ablation of PVCs triggering VF<br>Endpoints: No recurrence of VF<br>Study type: Multicenter, retrospective<br>Size: 27 patients	Inclusion criteria: Apparently normal hearts<br><br>Exclusion criteria: Long QT, CPVT, Brugada syndrome	89% with no recurrence off drug therapy at 24 ± 28 months	PVCs were elicited from the Purkinje system in 23 patients. QRS was prolonged by 20 ms in 3 patients.	Observational study without control group. Mean of 3.6 drugs were unsuccessfully tried first. Some 23 patients had ICDs.

Study name or acronym; Author; Year published; PMID	Aim of study; Endpoints; Study type (RCT, observational — multicenter or single center; case series or other [if RCT include intervention and comparator]); Study size (N)	Patient population with inclusion and exclusion criteria	Results (absolute event rates, *P* values; OR or RR; 95% CI)	Other relevant findings or adverse events	Limitations; Other comments; Conclusions
Knecht et al. Long-term follow-up of idiopathic ventricular fibrillation ablation: a multicenter study.<br>• Year published: 2009<br>• PMID: 19643313	Aim: To assess the long-term efficacy of ablation of PVCs triggering VF<br>Endpoints: No recurrence of VF<br>Study type: Multicenter, retrospective<br>Size: 38 patients from 6 centers	Inclusion criteria: Patients undergoing ablation for idiopathic VF; at least 1 event with PVC triggered VF on a 12-lead ECG<br><br>Exclusion criteria: SHD, MI, CPVT, long QT, BrS, channelopathies	At median follow-up of 63 months, 7 (18%) patients had VF recurrence at a median of 4 months. Five of these had no recurrence after repeat ablation.	Triggering PVCs were from the Purkinje system in 33 patients. One patient had transient LBBB. Six patients developed nonspecific IVCD.	Observational study, no control group. Refractory to a mean of 2 AADs prior to ablation. Of 38 patients, 37 had or received ICDs (1 refused). ICD still recommended, particularly as some recurrences were of a different PVC morphology.
Peichl et al. Catheter ablation of arrhythmic storm triggered by monomorphic ectopic beats in patients with coronary artery disease.<br>• Year published: 2010<br>• PMID: 19937101	Aim: To assess the efficacy of ablation of PVCs triggering VF in patients with a history of MI<br>Endpoints: Abolishment of VT/VF<br>Study type: Single center, retrospective<br>Size: 9 patients	Inclusion criteria: Monomorphic PVCs triggering frequent polymorphic VT/VF, history of MI, intravenous amiodarone and intravenous metoprolol ineffective	Successful in 8 out of 9 patients; remaining patient had continued VT/VF and died.	Triggering PVCs were in the periphery of the conduction system in 8 patients, scar border in the lateral wall in one.	Small study size, retrospective analysis, single center

Study name or acronym; Author; Year published; PMID	Aim of study; Endpoints; Study type (RCT, observational — multicenter or single center; case series or other [if RCT include intervention and comparator]); Study size (N)	Patient population with inclusion and exclusion criteria	Results (absolute event rates, *P* values; OR or RR; 95% CI)	Other relevant findings or adverse events	Limitations; Other comments; Conclusions
Lakkireddy et al. Radiofrequency ablation of premature ventricular ectopy improves the efficacy of cardiac resynchronization therapy in nonresponders.<br>• Year published: 2012<br>• PMID: 22999718	Aim: To evaluate the efficacy of ablation of frequent PVCs in CRT nonresponders<br>Endpoints: Ejection fraction, end-systolic diameter, NYHA class<br>Study type: Prospective, multicenter, nonrandomized, observational<br>Size: 65 patients	Inclusion criteria: Nonresponders to CRT (<5% EF improvement, <10% LV end-systolic volume improvement, no improvement in clinical status), >10,000 PVCs in 24 hours<br><br>Exclusion criteria: Sustained VT requiring device therapy, atrial fibrillation burden >1%	Mean EF improved (26.2% ± 5.5% to 32.7% ± 6.7%; *P*<.001); LV end-systolic diameter improved (5.83 ± 0.55 cm to 5.62 ± 0.32 cm; *P*<.001); LV end-diastolic diameter improved (6.83 ± 0.83 cm to 6.51 ± 0.91 cm; *P*<.001); and median NYHA class improved (3.0 to 2.0; *P*<.001).	86% had single PVC focus, 75% of PVCs were from LV, acute success in 91%, 2 complications (tamponade and TIA).	No control group. EF improved significantly when PVC burden was >22%.
Haïssaguerre et al. Mapping and ablation of ventricular fibrillation associated with long-QT and Brugada syndromes.<br>• Year published: 2003<br>• PMID: 12925452	Aim: To evaluate the efficacy of ablating PVCs triggering VF or PMVT in patients with long-QT or BrS<br>Endpoints: Recurrence of VAs<br>Study type: Retrospective, single center, observational<br>Size: 7 patients	Inclusion criteria: Patients with long-QTs or BrS with documented PMVT or VF, presence of PVCs triggering VF or PMVT	All 7 patients had successful ablation of PVCs. During a follow-up of 17 ± 17 months, there were no patients with recurrence of VF or PMVT. One patient had continued PVCs.	Among the 3 patients with BrS, 2 patients had PVCs localized to the RVOT and one patient had PVCs localized to the right Purkinje conduction system.<br><br>Among the 4 patients with LQTS, 3 patients had PVCs localized to the left Purkinje conduction system and one patient had PVCs localized to the RVOT.	Small study size, retrospective analysis, single center

Study name or acronym; Author; Year published; PMID	Aim of study; Endpoints; Study type (RCT, observational — multicenter or single center; case series or other [if RCT include intervention and comparator]); Study size (N)	Patient population with inclusion and exclusion criteria	Results (absolute event rates, *P* values; OR or RR; 95% CI)	Other relevant findings or adverse events	Limitations; Other comments; Conclusions
Sadek et al. Idiopathic ventricular arrhythmias originating from the moderator band: electrocardiographic characteristics and treatment by catheter ablation.<br>• Year published: 2015<br>• PMID: 25240695	Aim: To define ECG characteristics, describe procedural technique, and report outcomes of ablation of moderate band PVCs/VT<br>Endpoints: Freedom from ventricular arrhythmias<br>Study type: Retrospective, single center, observational	Inclusion criteria: Patients undergoing catheter ablation of PVCs or VT originating from the moderator band	All 10 patients had initial acutely successful ablation; 7 patients had recurrence, 1 had PVCs controlled with AAD, and 6 underwent repeat ablation.<br>During mean follow-up of 21.5 ± 11.6 months, 9 of 10 patients were free of VAs and all were free of sustained VT or ICD shocks.	ECG characteristics of moderator band VAs include LBBB morphology, left superior frontal plane axis, sharp precordial QRS downstroke, relatively narrow QRS (mean 152.7 ± 15.2 ms), and later transition than sinus QRS. In 7 of 10 patients, PVCs from the moderator band initiated VF. ICE guidance facilitates mapping and ablation of moderator band VAs.	Small study size, retrospective analysis, single center

Ventricular Arrhythmia in Ischemic Heart Disease

Study name or acronym; Author; Year published; PMID	Aim of study; Endpoints; Study type (RCT, observational — multicenter or single center; case series or other [if RCT include intervention and comparator]); Study size (N)	Patient population with inclusion and exclusion criteria	Results (absolute event rates, *P* values; OR or RR; 95% CI)	Other relevant findings or adverse events	Limitations; Other comments; Conclusions
Reddy et al. Prophylactic catheter ablation for the prevention of defibrillator therapy.<br>• Year published: 2007<br>• PMID: 18160685	Aim: Comparison of prophylactic substrate ablation versus control.<br>Primary endpoint: survival free from any appropriate ICD therapy (shocks or ATP); secondary endpoints: freedom from appropriate ICD shock, death, and ICD storm<br>Study type: RCT<br>Size: 128	Inclusion criteria: ≥18 years of age, MI >1 month before, planned or recent (≤6 months) secondary prevention ICD implant, primary prevention ICD with subsequent single appropriate ICD therapy<br><br>Exclusion criteria: Treated with a class I or III AAD, VA not due to MI, active cardiac ischemia, incessant VT necessitating immediate treatment	Primary endpoint: At 2 years, 8 (12%) patients in the ablation group and 21 (33%) in the control group received at least one appropriate ICD therapy (ATP or shock) (HR 0.35; 95% CI 0.15–0.78; *P*=.007).<br><br>Secondary endpoints: ICD shock (HR 0.27; 95% CI 0.11–0.67; *P*=.003); VT storm (HR 0.30; 95% CI 0.09–1.00; *P*=.06).	Mean age 67, and 87% were male. The index arrhythmia was VF in 18%, VT in 49%, syncope with inducible VT in 21%, and an ICD therapy for VT/VF in 12%.<br>Three patients assigned to the ablation group did not undergo the procedure. An additional 3 (5%) patients did not have any ablation lesions placed due to lack of scar.<br>Nonirrigated ablation catheter was used in 16%, irrigated ablation catheter in 79%.<br>Substantial ablation-related complications:<br>1 pericardial effusion managed conservatively;<br>1 exacerbation of congestive heart failure requiring prolonged hospitalization;<br>1 deep venous thrombosis requiring prolonged anticoagulation.<br>The 30-day mortality rate was zero, with no evidence of an adverse effect of catheter ablation on ventricular function or change in NYHA functional class.	Comparison of prophylactic substrate ablation versus control, no AADs. ICD programming not specified. No screening data. No QOL data.

Study name or acronym; Author; Year published; PMID	Aim of study; Endpoints; Study type (RCT, observational — multicenter or single center; case series or other [if RCT include intervention and comparator]); Study size (N)	Patient population with inclusion and exclusion criteria	Results (absolute event rates, *P* values; OR or RR; 95% CI)	Other relevant findings or adverse events	Limitations; Other comments; Conclusions
Kuck et al. Catheter ablation of stable ventricular tachycardia before defibrillator implantation in patients with coronary heart disease (VTACH): a multicentre randomised controlled trial.<br>• Year published: 2010<br>• PMID: 20109864	Aim: Comparison of prophylactic ablation with none in patients with stable VT and prior MI<br>Endpoints: Time to recurrence of sustained VT or VF<br>Study type: RCT<br>Size: 107	Inclusion criteria: Aged 18–80 years, secondary prevention ICD indication, stable clinical VT, CAD and prior MI, LVEF ≤50%<br><br>Exclusion criteria: MI within 1 month, cardiac surgery within 2 months, protruding LV thrombus, valvular heart disease that precluded LV access, unstable angina, incessant VT, bundle-branch re-entry tachycardia, contraindication to heparin, serum creatinine >220 µmol/L, heart failure class IV, or life expectancy less than 12 months	Primary endpoint: Time to first VT or VF (mean SD) 15.9 months (1.7) vs 11.3 (1.5); median (IQR) 18.6 (2.4-upper quartile not determinable) vs 5.9 (0.8–26.7); HR 0.61 (95% CI 0.37–0.99); *P*=.045, log-rank test. Appropriate ICD shock n (%) 14 (26.9%) vs 26 (47.3%), *P*=.045.	Only stable VT included. Antiarrhythmic drug therapy in both groups, including amiodarone in 35% of each at baseline.	Comparison of prophylactic ablation vs none in patients with stable VT and prior MI. Reduction in primary endpoint observed in patients with EF >30%. Ablation interrupted due to transient ischemic ST-segment elevation (1) and TIA (1).

Study name or acronym; Author; Year published; PMID	Aim of study; Endpoints; Study type (RCT, observational — multicenter or single center; case series or other [if RCT include intervention and comparator]); Study size (N)	Patient population with inclusion and exclusion criteria	Results (absolute event rates, *P* values; OR or RR; 95% CI)	Other relevant findings or adverse events	Limitations; Other comments; Conclusions
Al-Khatib et al. Catheter ablation for ventricular tachycardia in patients with an implantable cardioverter defibrillator (CALYPSO) pilot trial.<br>• Year published: 2015<br>• PMID: 25332150	Aim: Feasibility<br>Endpoints: Feasibility; secondary endpoints included death, time to first recurrent therapy for VT, recurrent VT, adverse events related to ablation or to antiarrhythmic medications, hospitalizations for VT<br>Size: 27	Inclusion criteria: ≥18 years of age, ICD for primary or secondary prevention, ischemic heart disease, >1 ICD shock or >3 ATP therapies for VT within 6 months<br><br>Exclusion criteria: Incessant VT, contraindication to VT ablation, nonischemic substrates, valvular disease requiring surgery or inoperable, heart transplant or imminent cardiac transplantation within 12 months, an LV assist device, heritable arrhythmias or increased risk for TdP with class III drugs, end-stage renal disease, estimated life expectancy of <1 year from a noncardiac cause, and women who are pregnant or who have childbearing potential and are not using a reliable method of contraception	The 3 most common reasons for failing screening were existing antiarrhythmic therapy, reversible cause, and incessant VT. Median time (25th, 75th percentiles) to recurrent VT was 75 days (51, 89) in the ablation arm and 57 days (30, 145) in the antiarrhythmic arm.	Study terminated prematurely	Serious adverse events: 3 (23%) in the ablation arm and 5 (36%) in the AAD arm

Study name or acronym; Author; Year published; PMID	Aim of study; Endpoints; Study type (RCT, observational — multicenter or single center; case series or other [if RCT include intervention and comparator]); Study size (N)	Patient population with inclusion and exclusion criteria	Results (absolute event rates, *P* values; OR or RR; 95% CI)	Other relevant findings or adverse events	Limitations; Other comments; Conclusions
Sapp et al. Ventricular tachycardia ablation versus escalation of antiarrhythmic drugs.<br>• Year published: 2016<br>• PMID: 27149033	Aim: To compare VT ablation to escalated medical therapy in patients who experienced a VT event while taking an antiarrhythmic drug<br>Endpoints: Primary outcome: composite of death, VT storm, or appropriate ICD shock after a 30-day treatment period<br>Study type: RCT<br>Size: 259	Inclusion criteria: Prior myocardial infarction, ICD, or an episode of VT during treatment with amiodarone or another class I or class III AAD within 6 months<br><br>Exclusion criteria: Included ACS, ineligible for amiodarone, protruding LV thrombus, creatinine clearance <15 mL/min, NYHA class IV or CCS class IV angina, recent STEMI, CABG, or PCI, and prior ablation	The rate of the primary outcome was significantly lower in the ablation group than in the escalated-therapy group (HR 0.72; 95% CI 0.53–0.98; *P*=.04).	Adverse events: In the escalated-therapy group, 3 deaths were attributed to AAD therapy (2 pulmonary toxicity and 1 hepatic dysfunction).<br>Other adverse events included hepatic dysfunction, tremor, ataxia, and drug adverse effects leading to therapy changes. Procedural complications among the patients in the ablation group included major bleeding (3), vascular injury (3), cardiac perforation (2), and heart block (1). In the escalated therapy group, treatment-related adverse events were more frequent (51 vs 22; *P*=.002) and occurred in more patients (39 vs 20; *P*=.003).	ICD programming was per protocol relatively conservative (VT zone of 150 bpm), but characteristic of the time. The rate of the primary outcome did not differ significantly in the subgroup of patients who were not taking amiodarone at baseline.

Study name or acronym; Author; Year published; PMID	Aim of study; Endpoints; Study type (RCT, observational — multicenter or single center; case series or other [if RCT include intervention and comparator]); Study size (N)	Patient population with inclusion and exclusion criteria	Results (absolute event rates, *P* values; OR or RR; 95% CI)	Other relevant findings or adverse events	Limitations; Other comments; Conclusions
Kuck et al. Impact of substrate modification by catheter ablation on implantable cardioverter-defibrillator interventions in patients with unstable ventricular arrhythmias and coronary artery disease: results from the multicenter randomized controlled SMS (substrate modification study).<br>• Year published: 2017<br>• PMID: 28292751	Aim: To compare VT ablation plus an ICD to an ICD only in patients with IHD and unstable VT<br>Primary endpoint: time to first recurrence of VT/VF<br>Secondary endpoints: appropriate ICD therapies, quality of life, cardiac readmissions, and severe clinical events (death, syncope, and electrical storm)<br>Study type: RCT<br>Size: 111	Inclusion criteria: Age 18 to 80, CAD, LVEF ≤40%, unstable spontaneous VT, or cardiac arrest or syncope with inducible unstable VT<br><br>Exclusion criteria: Left ventricular thrombus, NYHA functional class IV, acute MI within the preceding 2 months, valvular heart disease or a mechanical heart valve, unstable angina, cardiac surgery within the past 2 months, serum creatinine >220 mEq/L (>2.5 mg/dL), thrombocytopenia or coagulopathy, a contraindication to heparin, pregnancy, or participation in another investigational study	The primary end point was reached by the 25 patients in the ablation group and 26 ICD-only patients. Two-year estimates of freedom from VT or VF: 49.0% (95% CI 33.3%–62.9%) in the ablation group and 52.4% (95% CI 36.7%–65.9%) in the ICD-only group, log-rank *P*=.84.	There was no significant difference in the primary endpoint, and no difference in the secondary endpoints. There was a significant reduction in total number of VT/VF episodes in the ablation group.	Enrollment was slow and took 7 years.<br>Included only unstable VT.

Study name or acronym; Author; Year published; PMID	Aim of study; Endpoints; Study type (RCT, observational — multicenter or single center; case series or other [if RCT include intervention and comparator]); Study size (N)	Patient population with inclusion and exclusion criteria	Results (absolute event rates, *P* values; OR or RR; 95% CI)	Other relevant findings or adverse events	Limitations; Other comments; Conclusions
Stevenson et al. Irrigated radiofrequency catheter ablation guided by electroanatomic mapping for recurrent ventricular tachycardia after myocardial infarction: the multicenter thermocool ventricular tachycardia ablation trial.<br>• Year published: 2008<br>• PMID: 19064682	Aim: To evaluate the safety and efficacy of a RFAC with external irrigation combined with an EAM system for ablation of postinfarction VT<br>Endpoints: No recurrence of sustained monomorphic VT (in patients with incessant VT, no recurrence of incessant VT)<br>Study type: Observational multicenter study<br>Size: 231	Inclusion criteria: Sustained monomorphic VT requiring termination by cardioversion or AAD administration with 4 episodes in the previous 6 months despite an ICD or antiarrhythmic drug therapy<br><br>Exclusion criteria: Serum creatinine 2.5 mg/dL, LVEF ≤0.10, mobile LV thrombus, absence of vascular access, likely survival <12 months, NYHA class IV heart failure, recent cardiac surgery, unstable angina, severe valve disease, pregnancy	Primary endpoint of freedom from recurrent incessant VT or intermittent VT after 6 months was achieved in 123 patients (53%).	Seven patients (3%) died within 7 days of the procedure. Major complications related to the procedure occurred in 24 patients (7.3%); vascular access complications in 4.7%. No strokes or TIA.	In 142 patients with an ICD before and after ablation who survived 6 months, VT episodes were reduced from a median of 11.5 to 0 (*P*<.0001). The frequency of VT episodes was reduced by 75% in 67% of patients.

Study name or acronym; Author; Year published; PMID	Aim of study; Endpoints; Study type (RCT, observational — multicenter or single center; case series or other [if RCT include intervention and comparator]); Study size (N)	Patient population with inclusion and exclusion criteria	Results (absolute event rates, *P* values; OR or RR; 95% CI)	Other relevant findings or adverse events	Limitations; Other comments; Conclusions
Marchlinski et al. Long-term success of irrigated radiofrequency catheter ablation of sustained ventricular tachycardia: post-approval THERMOCOOL VT trial.<br>• Year published: 2016<br>• PMID: 26868693	Aim: To evaluate long-term safety and effectiveness of RF catheter VT ablation using an open-irrigated catheter<br>Endpoints: All-cause mortality at 12 months postablation<br>Study type: Prospective, nonrandomized, single-arm study<br>Size: 249	Inclusion criteria: ≥18 years of age, LVEF ≥10%, with recurrent or incessant VT due to MI ≥3 weeks previously<br><br>Exclusion criteria: Mobile LV thrombus; MI within the preceding 2 months (except incessant VT, MI ≥3 weeks previously); idiopathic VT; expected survival <12 months; NYHA class IV heart failure; serum creatinine ≥2.5 mg/dL; thrombocytopenia or coagulopathy; contraindications to heparin; pregnancy; recent cardiac surgery; acute illness or active systemic infection; unstable angina; severe aortic stenosis; or flail mitral valve	The CSAE rate was 3.9%; 114 of 184 (62%) patients had no VT recurrence for 6 months.<br>Median VT frequency was reduced between 6 months preablation and 6 months postablation from 13 to 0. The reduction in VT frequency was ≥75% in 82.0%.	Major complications in 3.9%: cardiac perforation (1), complete heart block (2), pericardial effusion (3), and death (3).<br><br>All deaths within 7 days of ablation were preceded by recurrent VT and patients had an LVEF <25%.	There was a significant reduction in the percentage of patients taking amiodarone postablation (55% to 31.3%; *P*<.0001), which continued to drop for 3 years.

Study name or acronym; Author; Year published; PMID	Aim of study; Endpoints; Study type (RCT, observational — multicenter or single center; case series or other [if RCT include intervention and comparator]); Study size (N)	Patient population with inclusion and exclusion criteria	Results (absolute event rates, *P* values; OR or RR; 95% CI)	Other relevant findings or adverse events	Limitations; Other comments; Conclusions
Tanner et al. Catheter ablation of recurrent scar-related ventricular tachycardia using electroanatomical mapping and irrigated ablation technology: results of the prospective multicenter Euro-VT-study.<br>• Year published: 2010<br>• PMID: 19656251	Aim: To investigate efficacy and safety of electroanatomical mapping in combination with used open irrigated ablation for ablation of recurrent mappable and unmappable VT in remote MI<br>Endpoints: Acute success: noninducibility of all clinically relevant VTs on hospital discharge; chronic success: no recurrence of VT at 6- and 12-month follow-up<br>Study type: Prospective, nonrandomized, observational<br>Size: 63	Inclusion criteria: Recurrent drug and/or device refractory sustained VT due to prior MI<br><br>Exclusion criteria: Age <18 years; protruding LV thrombus; MI within the preceding 2 months, or within the preceding 3 weeks in case of incessant VT; unstable angina; severe aortic stenosis or mitral regurgitation; unwillingness to participate in the study; or unavailable for follow-up visits	Acute success was achieved in 51 (81%) patients. During a mean follow-up of 12 ± 3 months, 31 (49%) developed VT recurrence. In 79% of the patients with VT recurrence, a significant reduction of device therapies (antitachycardia pacing and shocks) was observed. The mean number of therapies in the 6 months pre- and postablation was reduced from 60 ± 70 to 14 ± 15 (*P*=.02).	There was 1 adverse event: cardiac arrest requiring CPR. No death within 30 days. Standardized ablation technique. LVEF pre- and postablation was similar (both 30% ± 13%).	Patients who had only unmappable VT had equivalent acute success rates as those with only mappable VT.

Study name or acronym; Author; Year published; PMID	Aim of study; Endpoints; Study type (RCT, observational — multicenter or single center; case series or other [if RCT include intervention and comparator]); Study size (N)	Patient population with inclusion and exclusion criteria	Results (absolute event rates, *P* values; OR or RR; 95% CI)	Other relevant findings or adverse events	Limitations; Other comments; Conclusions
Carbucicchio et al. Catheter ablation for the treatment of electrical storm in patients with implantable cardioverter-defibrillators: short- and long-term outcomes in a prospective single-center study.<br>• Year published: 2008<br>• PMID: 18172038	Aim: Catheter ablation has been proposed for treating ES, but its long-term effect in a large population has never been verified<br>Study type: Prospective<br>Size: 95	Ninety-five consecutive patients with CAD (72 patients), idiopathic DCM (10 patients), and arrhythmogenic RV dysplasia or cardiomyopathy (13 patients) undergoing catheter ablation for drug-refractory ES were prospectively evaluated.	Pleomorphic or nontolerated VTs required electroanatomic and noncontact mapping in 48 and 22 patients, respectively, and percutaneous cardiopulmonary support in 10 patients. An epicardial approach was used in 10 patients. After 1 to 3 procedures, induction of any clinical VT(s) by programmed electrical stimulation was prevented in 85 (89%) patients. ES was acutely suppressed in all patients; a minimum period of 7 days with stable rhythm was required before hospital discharge. At a median follow-up of 22 months (range, 1–43 months), 87 (92%) patients were free of ES and 63 (66%) patients were free of VT recurrence. Eight of 10 patients with persistent inducibility of clinical VT(s) had ES recurrence; 4 of them died suddenly despite appropriate ICD intervention. Altogether, 11 of 95 (12%) patients died of cardiac-related reasons.	In the group of patients presenting with all clinical VTs acutely abolished, no ES recurrence was documented, and cardiac mortality was significantly lower compared with the group of patients showing ≥1 clinical VT still inducible after catheter ablation.	Advanced strategies of catheter ablation applied to a large population of patients are effective in the short-term treatment of ES.<br>By preventing ES recurrence, catheter ablation could play a protective role over the long term and, together with long-term pharmacological therapy, might favorably affect cardiac mortality.

Study name or acronym; Author; Year published; PMID	Aim of study; Endpoints; Study type (RCT, observational — multicenter or single center; case series or other [if RCT include intervention and comparator]); Study size (N)	Patient population with inclusion and exclusion criteria	Results (absolute event rates, *P* values; OR or RR; 95% CI)	Other relevant findings or adverse events	Limitations; Other comments; Conclusions
Deneke et al. Catheter ablation of electrical storm in a collaborative hospital network.<br>• Year published: 2011<br>• PMID: 21529742	Aim: To evaluate the effect of catheter ablation strategies in the setting of an interhospital collaborative network on the recurrence of VA episodes and mortality in patients with ES.<br>Size: 32	Consecutive patients presenting for invasive treatment of ES from December 2007 to December 2009 were included. All patients underwent catheter ablation of VA. The strategies were adapted to the individual cardiac pathologic features. The follow-up examination constituted periodic ICD interrogation. A total of 32 patients were included.	Of the 32 patients, 29 (91%) had monomorphic VT and 3 VF. The mean number of ICD-treated episodes within 7 days before ablation was 16 ± 11. Of the 32 patients, 27 underwent ablation within 24 hours after admission, and 5 underwent acute ablation within 8 hours. In 3 patients, epicardial ablation was performed. In all but 2 (6%) patients, the clinical arrhythmia was successfully ablated. During a median follow-up of 15 months, 10 (31%) patients had recurrences of sustained VA, including 2 (6%) patients with recurrent ES. Three (9%) patients died during the follow-up period.	Catheter ablation effectively suppressed VA midterm recurrences in patients presenting with ES.	Catheter ablation is complex in these severely sick patients. The VA recurrence rate appears to be 31%, and the mortality rate 9%.<br><br>Collaborative hospital networks to increase the prompt availability of ES ablation might help to optimize the ES outcome.

Study name or acronym; Author; Year published; PMID	Aim of study; Endpoints; Study type (RCT, observational — multicenter or single center; case series or other [if RCT include intervention and comparator]); Study size (N)	Patient population with inclusion and exclusion criteria	Results (absolute event rates, *P* values; OR or RR; 95% CI)	Other relevant findings or adverse events	Limitations; Other comments; Conclusions
Muser et al. Long-term outcomes of catheter ablation of electrical storm in nonischemic dilated cardiomyopathy compared with ischemic cardiomyopathy.<br>• Year published: 2017<br>• PMID: 29759543	Aim: To determine the long-term outcomes of catheter ablation of ES in patients with NIDCM compared with patients with ICM<br>Size: 267	The study included 267 consecutive patients with NIDCM (n=71; EF 32% ± 14%) and ICM (n=196; EF 28% ± 12%). Endo-epicardial catheter ablation was performed in 59 (22%) patients. Catheter ablation was guided by activation and entrainment mapping for tolerated VT and pace mapping/targeting of abnormal substrate for unmappable VT.	After a median follow-up of 45 (25th to 75th percentile: 9 to 71) months and 1 (25th to 75th percentile: 1 to 8) procedures, 76 (29%) patients died, 25 (9%) underwent heart transplantation, 87 (33%) experienced VT recurrence, and 13 (5%) had recurrence of ES. Overall VT-free survival was 54% at 60 months (48% in NIDCM and 54% in ICM; *P*=.128). Patients with VT recurrence experienced a median of 2 (1 to 10) VT episodes in the 5 (1 to 14) months after the procedure. Death/transplantation-free survival was 62% at 60 months (53% in NIDCM and 64% in ICM; *P*=.067). Persistent inducibility of any VT with cycle length ≥250 ms at programmed stimulation at the end of the procedure was the only independent predictor of VT recurrence.	Low EF, NYHA functional class, and VT recurrence over follow-up independently predicted death/transplantation.	Catheter ablation of ES was similarly effective in patients with NIDCM compared with patients with ICM, with elimination of ES in 95% of cases and achievement of complete VT control at long-term follow-up in most patients.

Study name or acronym; Author; Year published; PMID	Aim of study; Endpoints; Study type (RCT, observational — multicenter or single center; case series or other [if RCT include intervention and comparator]); Study size (N)	Patient population with inclusion and exclusion criteria	Results (absolute event rates, *P* values; OR or RR; 95% CI)	Other relevant findings or adverse events	Limitations; Other comments; Conclusions
Kumar et al. Beyond the storm: comparison of clinical factors, arrhythmogenic substrate, and catheter ablation outcomes in structural heart disease patients with versus those without a history of ventricular tachycardia storm.<br>• Year published: 2017<br>• PMID: 27781325	Aim: To compare the clinical factors, substrate, and outcome differences in patients with SMVT who present for catheter ablation with VT storm versus those with a nonstorm presentation<br>Size: Consecutive ischemic (ICM; n = 554) or nonischemic cardiomyopathy patients (NICM; n = 369) with a storm versus nonstorm presentation were studied (ICM storm 186; NICM storm 101)	Consecutive patients with ICM (554) or NICM (369) with a storm versus nonstorm presentation were studied (ICM storm 186; NICM storm 101)	In patients with ICM, storm compared with nonstorm patients had significantly lower LVEF, greater number of AAD failures, slower VTs, greater number of scarred LV segments, and higher incidence of anterior, septal, and apical endocardial LV scar (all *P*<.05). However, outcomes in follow-up were similar (12-month VA-free survival: 51% vs 52%, *P*=.6; survival free of death or transplant 75% vs 87%; *P*=.7). In addition to the above differences, patients with NICM storm were also older; however, the extent and distribution of scar was similar except for a higher incidence of lateral endocardial scar in patients with storm (*P*=.05). VA-free survival (36% vs 47%, *P*=.004) and survival free of death/transplant, however, were poorer in patients with NICM storm than nonstorm (72% vs 88%, *P*=.001).	Patients with NICM storm had poorer VA-free survival than patients with ICM storm.	There are differences in clinical factors and scar patterns in patients undergoing VT ablation who present with VT storm versus those with a nonstorm presentation. Clinical outcomes are poorer in patients with NICM storm.

Study name or acronym; Author; Year published; PMID	Aim of study; Endpoints; Study type (RCT, observational — multicenter or single center; case series or other [if RCT include intervention and comparator]); Study size (N)	Patient population with inclusion and exclusion criteria	Results (absolute event rates, *P* values; OR or RR; 95% CI)	Other relevant findings or adverse events	Limitations; Other comments; Conclusions
Nayyar et al. Venturing into ventricular arrhythmia storm: a systematic review and meta-analysis.<br>• Year published: 2013<br>• PMID: 23264584	Aim: To systematically synthesize the available literature to appreciate the efficacy and safety of ablation in the setting of VA storm<br>Study type: Systematic review and meta-analysis<br>Size: 471	VA storm was defined as recurrent (≥3 episodes or defibrillator therapies in 24 hours) or incessant (continuous >12 hours) VA. Studies reporting data on patients with VA storm at the individual or study level were included. A total of 471 patients with VA storm from 39 publications were analyzed.	All VAs were successfully ablated in 72% (95% CI 71%–89%), and 9% (95% CI 3%–10%) had a failed procedure. Procedure-related mortality occurred in 3 patients (0.6%). Only 6% of patients had a recurrence of VA storm. The recurrence of VA was significantly higher after ablation for arrhythmic storm of monomorphic VT relative to ventricular fibrillation or polymorphic VT with underlying cardiomyopathy (OR 3.76; 95% CI 1.65–8.57; *P*=.002). During the follow-up (61 ± 37 weeks), 17% of patients died (heart failure 62%, arrhythmias 23%, and noncardiac 15%), with 55% of deaths occurring within 12 weeks of intervention. The odds of death were 4 times higher after a failed procedure compared with those with a successful procedure (95% CI 2.04–8.01; *P*<.001).	VA storm ablation has high acute success rates, with a low rate of recurrent storms.	Heart failure is the dominant cause of death in the long term. Failure of the acute procedure carries high mortality.

Study name or acronym; Author; Year published; PMID	Aim of study; Endpoints; Study type (RCT, observational — multicenter or single center; case series or other [if RCT include intervention and comparator]); Study size (N)	Patient population with inclusion and exclusion criteria	Results (absolute event rates, *P* values; OR or RR; 95% CI)	Other relevant findings or adverse events	Limitations; Other comments; Conclusions
Martinez et al. Systematic review and meta-analysis of catheter ablation of ventricular tachycardia in ischemic heart disease.<br>• Year published: 2019	Aim: To perform a systematic review and meta-analysis of RCTs of catheter ablation of VT in patients with IHD<br>Study type: Systematic review and meta-analysis<br>Size: 635	A total of 5 RCTs (N=635 patients) were included, with duration of follow-up ranging from 6–27.9 months.	Patients who underwent catheter ablation experienced decreased odds of appropriate ICD therapies (OR 0.49; 95% CI 0.28–0.87), appropriate ICD shocks (OR 0.52; 95% CI 0.28–0.96), VT storm (OR 0.64, 95% CI 0.42–0.98), and cardiac hospitalization (OR 0.67, 95% CI 0.46–0.97) versus those who did not undergo ablation. Numerical reductions in the odds of recurrent VT/VF (OR 0.87, 95% CI 0.41–1.85) and all-cause mortality (OR 0.89, 95% CI 0.60–1.34) were observed, but 95% CIs included 1.0.	Catheter ablation was associated with a significant reduction in the odds of appropriate ICD therapies, appropriate ICD shocks, VT storm, and cardiac hospitalizations in patients with IHD.	

Study name or acronym; Author; Year published; PMID	Aim of study; Endpoints; Study type (RCT, observational — multicenter or single center; case series or other [if RCT include intervention and comparator]); Study size (N)	Patient population with inclusion and exclusion criteria	Results (absolute event rates, *P* values; OR or RR; 95% CI)	Other relevant findings or adverse events	Limitations; Other comments; Conclusions
Littmann et al. Functional role of the epicardium in postinfarction ventricular tachycardia.<br>• Year published: 1991<br>• PMID: 2022017	Aim: To prospectively evaluate the functional role of the epicardium in postinfarction VT with complex intraoperative techniques, including computerized electrical activation mapping, entrainment, observation of changes in activation pattern during successful epicardial laser photoablation, and histological study<br>Size: 10	Five of 10 consecutive patients undergoing intraoperative computerized activation mapping had 10 VT morphologies displaying epicardial diastolic activation. These 10 "epicardial" VTs revealed the following global activation patterns: monoregional spread (2), figure-8 activation (5), and circular macroreentry (3).	Entrainment of VT using epicardial stimulation was successfully performed from an area of slow diastolic conduction in four tachycardia morphologies. During entrainment, global activation remained undisturbed, with recordings showing a long stimulus to QRS interval, unchanged QRS morphology, and pacing capture of all components of the reentry circuit. Neodymium:yttrium-aluminum-garnet laser photocoagulation was delivered during VT to epicardial sites of presumed reentry. Epicardial photoablation terminated 5 of 5 figure-8 tachycardias and 2 of 3 circular macroreentry tachycardias, but not the monoregional tachycardias.	Electrophysiological recordings during epicardial laser photocoagulation demonstrated progressive prolongation of VT cycle length and apparent interruption of the presumed reentrant circuit.	Chronic postinfarction VT can result from subepicardial macroreentry; slow conduction within the reentry circuit can be localized by computerized mapping and epicardial entrainment; and VT interruption by laser photocoagulation results from conduction delay and block within critical elements of the reentrant pathway. Viable subepicardial muscle fibers might constitute the underlying pathology.

Study name or acronym; Author; Year published; PMID	Aim of study; Endpoints; Study type (RCT, observational — multicenter or single center; case series or other [if RCT include intervention and comparator]); Study size (N)	Patient population with inclusion and exclusion criteria	Results (absolute event rates, *P* values; OR or RR; 95% CI)	Other relevant findings or adverse events	Limitations; Other comments; Conclusions
Sosa et al. Nonsurgical transthoracic epicardial catheter ablation to treat recurrent ventricular tachycardia occurring late after myocardial infarction.<br>• Year published: 2000<br>• PMID: 10807445	Aim: To evaluate feasibility, safety, and results of transthoracic epicardial catheter ablation in patients with VT occurring late after an inferior wall MI<br>Size: 14	Fourteen consecutive patients aged 53.6 ± 14.5 years with postinfarction VT related to the inferior wall were studied. The VT cycle length was 412 ± 51 ms. Two patients had previously undergone unsuccessful standard endocardial RFCA. The VT was incessant in one patient. LV angiography showed inferior akinesia in 13 patients and an inferior aneurysm in 1 patient. Ablation was performed with a regular steerable catheter placed into the pericardial sac by pericardial puncture.	The pericardial space was reached in all patients. Electrophysiologic evidence of an epicardial circuit was present in 7 of 30 VTs. Due to a high stimulation threshold, empirical thermal mapping was the only criterion used to select the site for ablation. Three VTs were interrupted during the first RF pulse. Two pulses were necessary to render it noninducible in 3 patients (1 VT per patient). In the remaining 4 VTs, 3, 3, 4 and 5 RF pulses, respectively, were used. The overall success was 37.14% (95% CI 11.83%–62.45%). Patients were asymptomatic for 14 ± 2 months.	Postinfarction pericardial adherence does not preclude epicardial mapping and ablation to control VT related to an epicardial circuit in postinferior wall MI.	

Study name or acronym; Author; Year published; PMID	Aim of study; Endpoints; Study type (RCT, observational — multicenter or single center; case series or other [if RCT include intervention and comparator]); Study size (N)	Patient population with inclusion and exclusion criteria	Results (absolute event rates, *P* values; OR or RR; 95% CI)	Other relevant findings or adverse events	Limitations; Other comments; Conclusions
Schmidt et al. Catheter ablation for ventricular tachycardia after failed endocardial ablation: epicardial substrate or inappropriate endocardial ablation?<br>• Year published: 2010<br>• PMID: 20709191	Aim: To assess the incidence of epicardial substrates in patients with a previously failed endocardial ablation attempt for VT as well as the safety and effectiveness of epicardial ablation<br>Size: 59	A total of 59 patients with or without structural heart disease underwent epicardial VT ablation.	Pericardial access failed in 3 (5%) of these patients. Of the remaining 56, an epicardial substrate was found in 41 (73%). Overall, acute success was achieved in 46 (78%) of 59 patients, with complete VT abolition in 27 (46%) and partial abolition in 19 (32%). Successful outcomes were the result of endocardial ablation only in 14 (24%) patients, epicardial ablation in 21 (36%), and endocardial/epicardial in 11 (19%). Ablation failed to prevent reinduction in 8 (13%) patients, and VTs were noninducible prior to ablation in 5 (8%). Two periprocedural deaths occurred, one after RV perforation and one due to electromechanical dissociation. Hepatic bleeding occurred in two patients. Recurrence of any VT occurred in 27 (47%) of 57 surviving patients during median follow-up of 362 days (q1-q3; 180–468 days). Repeat epicardial mapping was not feasible due to adhesions in 3 (25%) of 12 patients.	In patients with a previously failed endocardial VT ablation, epicardial mapping reveals a VT substrate in nearly three-fourths of all patients, and epicardial ablation is required for successful VT abolition in more than half of patients. However, life-threatening complications can occur. Repeat epicardial access was not possible in 25% due to local pericardial adhesions.	

Study name or acronym; Author; Year published; PMID	Aim of study; Endpoints; Study type (RCT, observational — multicenter or single center; case series or other [if RCT include intervention and comparator]); Study size (N)	Patient population with inclusion and exclusion criteria	Results (absolute event rates, *P* values; OR or RR; 95% CI)	Other relevant findings or adverse events	Limitations; Other comments; Conclusions
Di Biase et al. Endo-epicardial homogenization of the scar versus limited endocardial substrate ablation for the treatment of electrical storms in patients with ischemic cardiomyopathy.<br>• Year published: 2012<br>• PMID: 22766340	Aim: To investigate the impact on recurrences of two different substrate approaches for the treatment of these arrhythmias<br>Size: 92	Ninety-two consecutive patients (81% male, age 62 ± 13 years) with ICM and ES underwent catheter ablation. Patients were treated either by confining the RF lesions to the endocardial surface with limited substrate ablation (Group 1, n=49) or underwent endocardial and epicardial ablation of abnormal potentials within the scar (homogenization of the scar, Group 2, n=43).	Epicardial access was obtained in all Group 2 patients, and epicardial ablation was performed in 33% (14) of these patients. Mean EF was 27% ± 5%. During a mean follow-up of 25 ± 10 months, the VAs recurrence rate of any VT was 47% (23 of 49 patients) in Group 1 and 19% (8 of 43 patients) in Group 2 (log-rank *P*=.006). One patient in Group 1 and 1 patient in Group 2 died at follow-up for noncardiac reasons.	Our study demonstrates that ablation using endo-epicardial homogenization of the scar significantly increases freedom from VAs in patients with ICM.	

Study name or acronym; Author; Year published; PMID	Aim of study; Endpoints; Study type (RCT, observational — multicenter or single center; case series or other [if RCT include intervention and comparator]); Study size (N)	Patient population with inclusion and exclusion criteria	Results (absolute event rates, *P* values; OR or RR; 95% CI)	Other relevant findings or adverse events	Limitations; Other comments; Conclusions
Izquierdo et al. Endo-epicardial versus only-endocardial ablation as a first line strategy for the treatment of ventricular tachycardia in patients with ischemic heart disease.<br>• Year published: 2015<br>• PMID: 26056239	Aim: To compare the efficacy of endocardial plus epicardial ablation vs only endocardial ablation in the first procedure in patients with IHD<br>Size: 53	Fifty-three patients with IHD, referred for a first VT ablation to our institution, from 2012 to 2014, were included. They were divided into 2 groups according to enrollment time: from May 2013, we started to systematically perform endo-epicardial access (Epi-Group) as a first-line approach in consecutive patients with IHD (n=15). Patients who underwent only an endocardial VT ablation in their first procedure (Endo-Group) included patients with previous cardiac surgery and the historical (before May 2013; n=35). All late potentials in the scar zone were eliminated, and if VT was tolerated, critical isthmuses were also approached. The end point was the noninducibility of any VT.	During a median follow-up of 15 ± 10 months, the combined end point (hospital or emergency admission because of a VT or reablation) occurred in 14 patients of the Endo-group and in one patient in the Epi-group (event-free survival curves by Grey-test, *P*=.03). Ventricular arrhythmia recurrences occurred in 16 and in 3 patients in the Endo and Epi-Group, respectively (Grey-test, *P*=.2).	A combined endocardial–epicardial ablation approach for initial VT ablation was associated with fewer readmissions for VT and repeat ablations.	

Nonischemic Cardiomyopathy

Study name or acronym; Author; Year published; PMID	Aim of study; Endpoints; Study type (RCT, observational — multicenter or single center; case series or other [if RCT include intervention and comparator]); Study size (N)	Patient population with inclusion and exclusion criteria	Results (absolute event rates, *P* values; OR or RR; 95% CI)	VT recurrence-free	Survival	Other relevant findings or adverse events
Muser et al. Long-term outcomes of catheter ablation of electrical storm in nonischemic dilated cardiomyopathy compared with ischemic cardiomyopathy.<br>• Year published: 2017<br>• PMID: 29759543	Aim: Outcome of catheter ablation in patients with ES<br>Endpoints: Mortality, ES, and VT recurrence<br>Study type: Retrospective single center<br>Size: 71 NICM vs 186 ICM	Inclusion criteria: ICM and NICM with ES	ns vs ICM in VT recurrences and mortality (53% and 54% free, respectively, in NICM).	0.53	0.54	No VT recurrence in most cases.
Muser et al. Long-term outcome after catheter ablation of ventricular tachycardia in patients with nonischemic dilated cardiomyopathy.<br>• Year published: 2016<br>• PMID: 27733494	Aim: Long-term follow-up after VT ablation in a large series of patients with NICM<br>Endpoints: VT recurrence and mortality<br>Study type: Retrospective single center<br>Size: 282 patients with DCM	Inclusion criteria: NICM with VT	Long-term success 69%, survival 76% at 60 months; decrease in VT recurrence for patients with recurring VT.	69% at 60 months	76% at 60 months	VT recurrences linked to LVEF and success at NIPS; mortality linked to LVEF, recurrences of VT, and NYHA score, less AADs during follow-up.

Study name or acronym; Author; Year published; PMID	Aim of study; Endpoints; Study type (RCT, observational — multicenter or single center; case series or other [if RCT include intervention and comparator]); Study size (N)	Patient population with inclusion and exclusion criteria	Results (absolute event rates, *P* values; OR or RR; 95% CI)	VT recurrence-free	Survival	Other relevant findings or adverse events
Hu et al. Can ventricular tachycardia non-inducibility after ablation predict reduced ventricular tachycardia recurrence and mortality in patients with non-ischemic cardiomyopathy? A meta-analysis of twenty-four observational studies.<br>• Year published: 2016<br>• PMID: 27521538	Aim: To evaluate programmed ventricular stimulation after VT ablation in patients with NICM.<br>Endpoints: VT recurrence and mortality<br>Study type: Meta-analysis<br>Size: 736 patients with NICM (ARVD included)	Inclusion criteria: NICM with VT and undergoing PVS after ablation (included ARVD).	Higher risk of VT recurrence (OR 5.83; 95% CI 4.07–8.37; *P*<.00001) and higher all-cause mortality (OR 3.55; 95% CI 1.62–7.78; *P*=.002) for patients with inducible VT after catheter ablation in NICM.			Combined endo-epicardial ablation significantly reduced the risk of VT recurrence compared with endocardial-only ablation (OR 2.02; 95% CI 1.19–3.44; *P*=.009).
Proietti et al. Substrate-guided ablation of haemodynamically tolerated and untolerated ventricular tachycardia in patients with structural heart disease: effect of cardiomyopathy type and acute success on long-term outcome.<br>• Year published: 2015<br>• PMID: 25488957	Aim: To evaluate substrate ablation in patients with ICM and NICM.<br>Endpoints: VT recurrence and mortality<br>Study type: Retrospective single center but prospectively acquired data<br>Size: 87 ICM and 55 NICM	Inclusion criteria: Patients with ICM and NICM undergoing substrate ablation	More acute success in ICM (641 ± 301 days follow-up; 74% VT-free in ICM vs 49% in NICM; *P*=.03), less hospitalization (15% ICM vs 31% NICM; *P*=.03), and mortality unchanged (9% vs 11% in NICM; *P*=ns).	49% at almost 2 years	89% at almost 2 years	Recurrences linked to acute success in NICM; fewer scars in NICM.

**Study name or acronym; Author; Year published; PMID**	**Aim of study; Endpoints; Study type (RCT, observational — multicenter or single center; case series or other [if RCT include intervention and comparator]); Study size (N)**	**Patient population with inclusion and exclusion criteria**	**Results (absolute event rates, *P* values; OR or RR; 95% CI)**	**VT recurrence-free**	**Survival**	**Other relevant findings or adverse events**
Dinov et al. Early referral for ablation of scar-related ventricular tachycardia is associated with improved acute and long-term outcomes: results from the Heart Center of Leipzig ventricular tachycardia registry.<br>• Year published: 2014<br>• PMID: 25262159	Aim: To compare early and late referral for VT ablation in NICM.<br>Endpoints: VT recurrence and mortality<br>Study type: Retrospective single center<br>Size: 204 ICM and 96 NICM	Inclusion criteria: ICM and NICM with VT and early or late referral for catheter ablation	Early ablation leads to better outcome in terms of acute success or VT recurrence but not in cardiac mortality.	NA for NICM	NA for NICM	Poorer outcome for DCM vs ICM; no specific data for early vs late NICM ablation.
Dinov et al. Outcomes in catheter ablation of ventricular tachycardia in dilated nonischemic cardiomyopathy compared with ischemic cardiomyopathy: results from the Prospective Heart Centre of Leipzig VT (HELP-VT) Study.<br>• Year published: 2014<br>• PMID: 24211823	Aim: To compare results of VT ablation in patients with ICM and NICM.<br>Endpoints: VT recurrence and mortality<br>Study type: Prospective single center<br>Size: 63 NICM vs 164 ICM	Inclusion criteria: VT ablation in NICM and ICM	Similar acute success rate (two-thirds full success), VT recurrence higher for NICM (1.62; 95% CI 1.12–2.34; *P*=.01), mortality similar.	23% at 20 months	87% at 20 months	NICM: acute success rate related to epicardial ablation and nb of VT; recurrences linked to acute success; few AADs on follow-up.

Study name or acronym; Author; Year published; PMID	Aim of study; Endpoints; Study type (RCT, observational — multicenter or single center; case series or other [if RCT include intervention and comparator]); Study size (N)	Patient population with inclusion and exclusion criteria	Results (absolute event rates, *P* values; OR or RR; 95% CI)	VT recurrence-free	Survival	Other relevant findings or adverse events
Tokuda et al. Catheter ablation of ventricular tachycardia in nonischemic heart disease.<br>• Year published: 2012<br>• PMID: 22942218	Aim: To evaluate the results of VT ablation in different NICMs<br>Endpoints: Mortality and combined index (death, transplantation, or readmission because of VT recurrence) and hospitalization for VT recurrences<br>Study type: Retrospective single center<br>Size: 226 patients with NICM (119 DCM and 13 sarcoidosis)	Inclusion criteria: NICM with VT	There was 43% acute success in DCM and 62% in sarcoidosis; outcome not related to acute results; DCM and sarcoidosis had poorest outcomes.	82% at 1 year (NICM NA)	74% at 4.4 years (with transplant) for NICM.	
Tung et al. Freedom from recurrent ventricular tachycardia after catheter ablation is associated with improved survival in patients with structural heart disease: an international VT ablation center collaborative group study.<br>• Year published: 2015<br>• PMID: 26031376	Aim: Long-term results of VT ablation in a wide multicenter population with SHD<br>Endpoints: Acute success, long-term recurrence, and mortality<br>Study type: Retrospective multicenter<br>Size: 2061 patients (966 NICM, 72% dilated idiopathic)	Inclusion criteria: SHD with VT and scar	There were 32% recurrences in DCM (> than ICM), with mortality or transplantation in 15%.	68% at 527 days	85% at 1 year	2/3 acute success in the whole group; survival linked to lack of VT recurrences also in NICM 92% vs 72%, *P*<.001). (81% vs 53%, adjusted HR of 6.746 (4.211-10.807); *P*<.001 for EF < 30% and 96% vs 84%, adjusted HR 9.293 (4.867-17.743); *P*<.001 for EF >30%

Study name or acronym; Author; Year published; PMID	Aim of study; Endpoints; Study type (RCT, observational — multicenter or single center; case series or other [if RCT include intervention and comparator]); Study size (N)	Patient population with inclusion and exclusion criteria	Results (absolute event rates, *P* values; OR or RR; 95% CI)	VT recurrence-free	Survival	Other relevant findings or adverse events
Arya et al. Catheter ablation of electrical storm due to monomorphic ventricular tachycardia in patients with nonischemic cardiomyopathy: acute results and its effect on long-term survival.<br>• Year published: 2010<br>• PMID: 20636312	Aim: Results of catheter ablation in ES in NICM<br>Endpoints: Mortality and VT recurrences<br>Study type: Retrospective single center<br>Size: 13 patients	Inclusion criteria: NICM and ES	There were 61% complete acute success, 69% survival, and 61% with no recurring VT.	61% at 2 years	69% at 2 years	
Marchlinski et al. Linear ablation lesions for control of unmappable ventricular tachycardia in patients with ischemic and nonischemic cardiomyopathy.<br>• Year published: 2000<br>• PMID: 10725289	Aim: Results of VT ablation in ICM and NICM for unmappable VT<br>Endpoints: VT recurrences<br>Study type: Retrospective single center<br>Size: 16 patients (7 NICM)	Inclusion criteria: NICM and ICM and unmappable VT	75% no recurrence	0.75	NA	

Study name or acronym; Author; Year published; PMID	Aim of study; Endpoints; Study type (RCT, observational — multicenter or single center; case series or other [if RCT include intervention and comparator]); Study size (N)	Patient population with inclusion and exclusion criteria	Results (absolute event rates, *P* values; OR or RR; 95% CI)	VT recurrence-free	Survival	Other relevant findings or adverse events
Delacretaz et al. Mapping and radiofrequency catheter ablation of the three types of sustained monomorphic ventricular tachycardia in nonischemic heart disease.<br>• Year published: 2000<br>• PMID: 10695454	Aim: Characterization of VT mechanisms in NICM<br>Endpoints: VT recurrences and mechanisms<br>Study type: Retrospective single center<br>Size: 26 patients	Inclusion criteria: Monomorphic VT in NICM	There was 19% BBB, 27% focal, otherwise scar reentry; 77% long-term success.	0.77	NA	
Soejima et al. Endocardial and epicardial radiofrequency ablation of ventricular tachycardia associated with dilated cardiomyopathy: the importance of low-voltage scars.<br>• Year published: 2004<br>• PMID: 15145109	Aim: Characterization of scars and VT in NICM<br>Endpoints: VT recurrences and mechanisms<br>Study type: Retrospective single center<br>Size: 28 patients	Inclusion criteria: Mappable VT in NICM	There was 7% BBB, 18% focal and 78% scar reentry; 54% long-term success in scar VT.	0.54	NA	

Study name or acronym; Author; Year published; PMID	Aim of study; Endpoints; Study type (RCT, observational — multicenter or single center; case series or other [if RCT include intervention and comparator]); Study size (N)	Patient population with inclusion and exclusion criteria	Results (absolute event rates, *P* values; OR or RR; 95% CI)	VT recurrence-free	Survival	Other relevant findings or adverse events
Carbucicchio et al. Catheter ablation for the treatment of electrical storm in patients with implantable cardioverter-defibrillators: short- and long-term outcomes in a prospective single-center study.<br>• Year published: 2008<br>• PMID: 18172038	Aim: Prognosis of ES ablation in SHD<br>Endpoints: VT recurrences and mortality<br>Study type: Retrospective single center but prospectively acquired data<br>Size: 72 ICM, 13 ARVD, 10 NICM	Inclusion criteria: ES in SHD	NICM data NA, but better survival compared with ICM.	NA for NICM	NA for NICM	
Hsia et al. Characterization of endocardial electrophysiological substrate in patients with nonischemic cardiomyopathy and monomorphic ventricular tachycardia.<br>• Year published: 2003<br>• PMID: 12885746	Aim: Characterization of endocardial mapping in NICM<br>Endpoints: Endocardial scar characteristics<br>Study type: Retrospective single center<br>Size: 19 patients with NICM	Inclusion criteria: NICM with VT and endocardial mapping	Modest-sized basal area of endocardial electrogram abnormalities in 100%.	47% at 22 months	79% at 22 months	
Sacher et al. Ventricular tachycardia ablation: evolution of patients and procedures over 8 years.<br>• Year published: 2008<br>• PMID: 19808409	Aim: Evolution of VT ablation in many settings over time<br>Endpoints: Evolution of ablation and mortality<br>Study type: Retrospective single center<br>Size: 493 patients (149 with NICM)	Inclusion criteria: Any VT ablation performed between 1999 and 2006.	NICM VT ablation increased from 27% to 35% (*P*=.06) between the years 1999–2002 and 2003–2006.	61% at 3.3 years (median 13 months)	83% at 3.3 years (median 13 months)	

Study name or acronym; Author; Year published; PMID	Aim of study; Endpoints; Study type (RCT, observational — multicenter or single center; case series or other [if RCT include intervention and comparator]); Study size (N)	Patient population with inclusion and exclusion criteria	Results (absolute event rates, *P* values; OR or RR; 95% CI)	VT recurrence-free	Survival	Other relevant findings or adverse events
Della Bella et al. Management of ventricular tachycardia in the setting of a dedicated unit for the treatment of complex ventricular arrhythmias: long-term outcome after ablation.<br>• Year published: 2013<br>• PMID: 23439513	Aim: Results of VT ablation in various SHD according to risk stratification and acute results.<br>Endpoints: VT recurrence and mortality<br>Study type: Retrospective single center<br>Size: 616 patients (109 with NICM)	Inclusion criteria: Any VT ablation in SHD.	More VT recurrence in NICM (multivariate HR 1.747 [1.039–2.939]; *P*=.035) but no change in mortality; NICM predictive of recurrence in high risk patients; mortality linked to acute results of ablation (no data for NICM).	NA for NICM	93% at 26 months	
Komatsu et al. Impact of substrate-based ablation of ventricular tachycardia on cardiac mortality in patients with implantable cardioverter-defibrillators.<br>• Year published: 2015<br>• PMID: 26332030	Aim: Role of VT ablation in reducing mortality<br>Endpoints: VT recurrence and mortality<br>Study type: Retrospective multicenter<br>Size: 195 patients (51 with NICM)	Inclusion criteria: SHD with VT ablation (substrate mapping)	Successful ablation (LAVA + noninducibility) linked to lower mortality (for NICM, HR 0.033; 95% CI 0.11–0.97; *P*=.04).	NA for NICM	NA for NICM	
Kottkamp et al. Radiofrequency catheter ablation of sustained ventricular tachycardia in idiopathic dilated cardiomyopathy.<br>• Year published: 1995<br>• PMID: 7648661	Aim: First description of VT ablation in NICM<br>Endpoints: Recurrence and mortality<br>Study type: Retrospective single center<br>Size: 9 patients	Inclusion criteria: VT ablation in NICM		25% at 8 months	87.5% at 8 months	

Study name or acronym; Author; Year published; PMID	Aim of study; Endpoints; Study type (RCT, observational — multicenter or single center; case series or other [if RCT include intervention and comparator]); Study size (N)	Patient population with inclusion and exclusion criteria	Results (absolute event rates, *P* values; OR or RR; 95% CI)	VT recurrence-free	Survival	Other relevant findings or adverse events
Della Bella et al. Epicardial ablation for ventricular tachycardia: a European multicenter study.<br>• Year published: 2011<br>• PMID: 21841191	Aim: Epicardial ablation in SHD<br>Endpoints: Recurrence and mortality<br>Study type: Retrospective multicenter<br>Size: 222 patients (67 with NICM)	Inclusion criteria: Epicardial VT ablation		61% at 17 months	93% at 17 months	
Sacher et al. Epicardial ventricular tachycardia ablation: a multicenter safety study.<br>• Year published: 2010<br>• PMID: 20488308	Aim: Epicardial ablation in SHD<br>Endpoints: Recurrence and mortality<br>Study type: Retrospective multicenter<br>Size: 134 patients (39 with NICM)	Inclusion criteria: Epicardial VT ablation		NA	NA	
Cano et al. Electroanatomic substrate and ablation outcome for suspected epicardial ventricular tachycardia in left ventricular nonischemic cardiomyopathy.<br>• Year published: 2009<br>• PMID: 19695457	Aim: Epicardial ablation in NICM<br>Endpoints: Recurrence and mortality<br>Study type: Retrospective single center<br>Size: 22 patients	Inclusion criteria: Epicardial VT ablation		71% at 18 months	82% at 1 year	

Study name or acronym; Author; Year published; PMID	Aim of study; Endpoints; Study type (RCT, observational — multicenter or single center; case series or other [if RCT include intervention and comparator]); Study size (N)	Patient population with inclusion and exclusion criteria	Results (absolute event rates, *P* values; OR or RR; 95% CI)	VT recurrence-free	Survival	Other relevant findings or adverse events
Frankel et al. Ventricular tachycardia ablation remains treatment of last resort in structural heart disease: argument for earlier intervention.<br>• Year published: 2011<br>• PMID: 21539642	Aim: To compare early with late referral for VT ablation in SHD<br>Endpoints: Recurrence and mortality<br>Study type: Retrospective single center<br>Size: 98 patients (37 NICM)	Inclusion criteria: VT ablation in SHD with VT.	Early referral for VT ablation was an independent factor for fewer VT recurrences (mortality unchanged, NICM data NA).	NA	NA	Less amiodarone use after ablation.
Furushima et al. Ventricular tachyarrhythmia associated with cardiac sarcoidosis: its mechanisms and outcome.<br>• Year published: 2004<br>• PMID: 15119697	Aim: Study mechanisms of VT in cardiac sarcoidosis<br>Endpoints: VT mechanisms<br>Study type: Retrospective single center<br>Size: 8 patients	Inclusion criteria: Cardiac sarcoidosis and VT	There were multiple inducible VTs, with demonstrable reentry in 70%.	NA	NA	
Koplan et al. Refractory ventricular tachycardia secondary to cardiac sarcoid: electrophysiologic characteristics, mapping, and ablation.<br>• Year published: 2006<br>• PMID: 16876741	Aim: Study mechanisms and outcome of refractory VT in cardiac sarcoidosis<br>Endpoints: VT mechanisms, recurrence and mortality<br>Study type: Retrospective single center<br>Size: 8 pts	Inclusion criteria: Cardiac sarcoidosis and refractory VT	There was 12% complete success (75% with at least one VT ablated). Recurrent VT in most at short term, then 50% VT-free with AADs and immunosuppression.	50% long-term follow-up	62% transplantation	

Study name or acronym; Author; Year published; PMID	Aim of study; Endpoints; Study type (RCT, observational — multicenter or single center; case series or other [if RCT include intervention and comparator]); Study size (N)	Patient population with inclusion and exclusion criteria	Results (absolute event rates, *P* values; OR or RR; 95% CI)	VT recurrence-free	Survival	Other relevant findings or adverse events
Uusimaa et al. Ventricular tachyarrhythmia as a primary presentation of sarcoidosis.<br>• Year published: 2008<br>• PMID: 18456644	Aim: Outcome of VT in cardiac sarcoidosis as an initial presentation<br>Endpoints: VT mechanisms, recurrence, and mortality<br>Study type: Retrospective single center<br>Size: 9 patients	Inclusion criteria: Cardiac sarcoidosis and VT as an initial presentation.	Ablation in 2 with incessant VT transplantation in 1.	NA	NA	
Jefic et al. Role of radiofrequency catheter ablation of ventricular tachycardia in cardiac sarcoidosis: report from a multicenter registry.<br>• Year published: 2009<br>• PMID: 19187909	Aim: Outcome of recurrent VT in cardiac sarcoidosis after VT ablation<br>Endpoints: Recurrence<br>Study type: Multicenter registry<br>Size: 9 of 24 patients with cardiac sarcoidosis	Inclusion criteria: Cardiac sarcoidosis and recurrent VT	There was 70% acute success; most patients had dramatic reduction of recurrences: from 271 ± 363 episodes preablation to 4.0 ± 9.7 episodes postablation; complete VT elimination occurred in 5 of 9 patients.	55% at 20 months	NA	
Naruse et al. Systematic treatment approach to ventricular tachycardia in cardiac sarcoidosis.<br>• Year published: 2014<br>• PMID: 24837644	Aim: Management of VT in cardiac sarcoidosis<br>Endpoints: Recurrence and outcome<br>Study type: Retrospective multicenter<br>Size: 14 of 37 patients with VT and cardiac sarcoidosis	Inclusion criteria: Cardiac sarcoidosis and VT	> 80% acute success	58% at 33 months	95% at 39 months (most with ICD)	

Study name or acronym; Author; Year published; PMID	Aim of study; Endpoints; Study type (RCT, observational — multicenter or single center; case series or other [if RCT include intervention and comparator]); Study size (N)	Patient population with inclusion and exclusion criteria	Results (absolute event rates, *P* values; OR or RR; 95% CI)	VT recurrence-free	Survival	Other relevant findings or adverse events
Kumar et al. Ventricular tachycardia in cardiac sarcoidosis: characterization of ventricular substrate and outcomes of catheter ablation.<br>• Year published: 2015<br>• PMID: 25527825	Aim: Outcome and characteristics of VT ablation in cardiac sarcoidosis<br>Endpoints: Recurrence and outcome<br>Study type: Retrospective single center<br>Size: 21 patients (5% of all NICM)	Inclusion criteria: Cardiac sarcoidosis and VT	Complete acute success in 43% (at least one VT eliminated in 90%, clinical VT eliminated in 90%), reduction of VT burden after several procedures (median 3 to 0; *P*<.0001).	37% at 1 year	91% at 1 year (76% death and transplantation)	Cessation of VT storm in 7 of 9.
Bandyopadhyay et al. Outcome of cardiac sarcoidosis after radiofrequency ablation and placement of AICD: a propensity matched analysis.<br>• Year published: 2015<br>• PMID: 26237358	Aim: Comparison of VT ablation plus ICD versus ICD alone in cardiac sarcoidosis.<br>Endpoints: Recurrence and outcome<br>Study type: Retrospective single center<br>Size: 20 patients with ablation (13 ICD) and 33 patients with ICD alone	Inclusion criteria: Cardiac sarcoidosis and VT	No advantage was found for RFCA plus ICD vs ICD alone; the requirement for ICD therapy increased over time following RFCA, especially after 12 months.	30% at 48 months (ICD th, mortality, new CA, and transplantation)	30% at 48 months (ICD th, mortality, new CA, and transplantation)	
Muser et al. Long-term outcomes of catheter ablation of ventricular tachycardia in patients with cardiac sarcoidosis.<br>• Year published: 2016<br>• PMID: 27516457	Aim: Outcome and characteristics of refractory VT ablation in cardiac sarcoidosis<br>Endpoints: Recurrence and outcome<br>Study type: Retrospective single center<br>Size: 31 patients	Inclusion criteria: Cardiac sarcoidosis and refractory VT	There were 80% acute successes (90% clinical VT eliminated), multiple ablations required in one-third, and decrease in VT burden (median from 10 [3–22] episodes to 1 [0–5] per patient).	55% at 2 years	83% at 2 years (death and transplantation)	

Study name or acronym; Author; Year published; PMID	Aim of study; Endpoints; Study type (RCT, observational — multicenter or single center; case series or other [if RCT include intervention and comparator]); Study size (N)	Patient population with inclusion and exclusion criteria	Results (absolute event rates, *P* values; OR or RR; 95% CI)	VT recurrence-free	Survival	Other relevant findings or adverse events
Yalagudri et al. Tailored approach for management of ventricular tachycardia in cardiac sarcoidosis.<br>• Year published: 2017<br>• PMID: 28429512	Aim: To compare management of VT according to PET<br>Endpoints: Recurrence<br>Study type: Retrospective single center<br>Size: 4 patients with RFCA of scar VT (PET negative) vs 14 patients with active disease.	Inclusion criteria: Cardiac sarcoidosis and VT (active on PET or not)	Good RFCA result in the nonactive phase.	NA	NA	
Dechering et al. Electrophysiological characteristics of ventricular tachyarrhythmias in cardiac sarcoidosis versus arrhythmogenic right ventricular cardiomyopathy.<br>• Year published: 2013<br>• PMID: 23070261	Aim: To compare management of VT in ARVD or sarcoidosis.<br>Endpoints: Ablation success and characteristics<br>Study type: Retrospective single center<br>Size: 8 patients with sarcoidosis and 10 with ARVD	Inclusion criteria: Cardiac sarcoidosis and ARVD	Similar acute RF success	NA	NA	
Muser et al. Prognostic role of serial quantitative evaluation of 18F-fluorodeoxyglucose uptake by PET/CT in patients with cardiac sarcoidosis presenting with ventricular tachycardia.<br>• Year published: 2018<br>• PMID: 29610956	Aim: To compare outcome after VT ablation in cardiac sarcoidosis according to change in PET<br>Endpoints: Recurrence and outcome<br>Study type: Retrospective single center<br>Size: 20 patients	Inclusion criteria: Cardiac sarcoidosis and VT	Outcome linked to change in PET (20-fold higher risk of MACE at follow-up in nonresponders)	NA	NA	

Study name or acronym; Author; Year published; PMID	Aim of study; Endpoints; Study type (RCT, observational — multicenter or single center; case series or other [if RCT include intervention and comparator]); Study size (N)	Patient population with inclusion and exclusion criteria	Results (absolute event rates, *P* values; OR or RR; 95% CI)	VT recurrence-free	Survival	Other relevant findings or adverse events
Papageorgiou et al. Catheter ablation for ventricular tachycardia in patients with cardiac sarcoidosis: a systematic review.<br>• Year published: 2018<br>• PMID: 28444174	Aim: Review of available studies on catheter ablation for VT in cardiac sarcoidosis<br>Endpoints: Recurrent VT and outcome<br>Study type: Meta-analysis<br>Size: 83 patients (5 studies)	Inclusion criteria: Cardiac sarcoidosis and VT	Reduction in VT burden (0.33; 95% CI 0.108–0.551; *P*<.004), 88% of patients improved.	46% at around 2 years	NA	
Kumar et al. Multicenter experience with catheter ablation for ventricular tachycardia in Lamin A/C cardiomyopathy.<br>• Year published: 2016<br>• PMID: 27506821	Aim: Outcome of VT ablation in NICM with LMNA mutations<br>Endpoints: Characteristics, recurrent VT, and outcome<br>Study type: Multicenter retrospective<br>Size: 25 patients	Inclusion criteria: LMNA mutations, NICM and VT	Acute complete success 25%; 25% complications; 17% VT free after multiple procedures; decrease in ICD therapy not significant; borderline decrease in AADs; 44% progression to heart failure; 31% mortality and transplantation.	17% at 7 months	69% at 7 months (death and transplantation)	

Ventricular Arrhythmia Involving the His-Purkinje System, Bundle Branch Reentrant Ventricular Tachycardia, and Fascicular Ventricular Tachycardia

Study name or acronym; Author; Year published; PMID	Aim of study; Endpoints; Study type (RCT, observational — multicenter or single center; case series or other [if RCT include intervention and comparator]); Study size (N)	Patient population with inclusion and exclusion criteria	Results (absolute event rates, *P* values; OR or RR; 95% CI)	Other relevant findings or adverse events	Limitations; Other comments; Conclusions
**BBRT**					
Pathak et al. Long-term outcome of catheter ablation for treatment of bundle branch re-entrant tachycardia<br>• Year published: 2018<br>• PMID: 30089558	Size: 32<br>Single-center observational	Patients with bundle branch reentrant ventricular tachycardia (BBRVT) who underwent catheter ablation for VT.	Recurrent VT due to BBRVT did not occur in any patient. Ten (31%) patients had normal LV function (LVEF ≥50%), and 22 (69%) had depressed LV function (LVEF <50%). No deaths were recorded in patients with normal LV function (5 with no implantable cardioverter-defibrillator) compared with 6 deaths among patients with depressed LV function (n=22; *P*=.07).		RFCA of the bundle branch is an effective therapy for treatment of BBRVT. The long-term outcome depends on the underlying cardiac disease.

Study name or acronym; Author; Year published; PMID	Aim of study; Endpoints; Study type (RCT, observational — multicenter or single center; case series or other [if RCT include intervention and comparator]); Study size (N)	Patient population with inclusion and exclusion criteria	Results (absolute event rates, *P* values; OR or RR; 95% CI)	Other relevant findings or adverse events	Limitations; Other comments; Conclusions
Cohen et al. Radiofrequency catheter ablation for treatment of bundle branch reentrant ventricular tachycardia: results and long-term follow-up.<br>• Year published: 1991<br>• PMID: 1960328	Size: 7<br>Single-center observational	Patients had BBRVT and underwent RFCA of the right bundle branch.	After RFCA, none had inducible BBRVT on restudy. On restudy, 3 of the 7 patients had VT of myocardial origin (not BBR). One patient required no therapy; drug or defibrillator therapy was used in the others. After a mean follow-up interval of 12 ± 3 months (range 6 to 29) complete RBBB persisted, there were no spontaneous episodes of VT, and no patient required a permanent pacemaker.		Catheter ablation is useful. A permanent device (pacemaker/CRT/ICD) might be required due to conduction system impairment, progressive HF, or other VT.

Study name or acronym; Author; Year published; PMID	Aim of study; Endpoints; Study type (RCT, observational — multicenter or single center; case series or other [if RCT include intervention and comparator]); Study size (N)	Patient population with inclusion and exclusion criteria	Results (absolute event rates, *P* values; OR or RR; 95% CI)	Other relevant findings or adverse events	Limitations; Other comments; Conclusions
Blanck et al. Bundle branch reentrant ventricular tachycardia: cumulative experience in 48 patients.<br>• Year published: 1993<br>• PMID: 8269297	Size: 48<br>Single-center observational	Forty-eight patients were identified in whom a diagnosis of BBR tachycardia was made during electrophysiologic evaluation.	SHD was present in 45 patients and no organic heart disease was identified in 3. All 48 patients had evidence of HPS disease. Management of BBR tachycardia included transcatheter bundle branch ablation in 28 patients, and AAD therapy in 16 patients. Four patients were treated with ICDs. After a mean follow-up of 15.8 months in 42 patients, there were 13 deaths due to CHF, 4 SCDs, 3 nonsudden cardiac deaths, and 3 noncardiac-related deaths.		Catheter ablation of the right bundle branch can be easily performed and effectively eliminates BBR. During follow-up, CHF is the most common cause of death in this population.

Study name or acronym; Author; Year published; PMID	Aim of study; Endpoints; Study type (RCT, observational — multicenter or single center; case series or other [if RCT include intervention and comparator]); Study size (N)	Patient population with inclusion and exclusion criteria	Results (absolute event rates, *P* values; OR or RR; 95% CI)	Other relevant findings or adverse events	Limitations; Other comments; Conclusions
Mehdirad et al. Long-term clinical outcome of right bundle branch radiofrequency catheter ablation for treatment of bundle branch reentrant ventricular tachycardia.<br>Year published: 1995<br>PMID: 8771124	Size: 16<br>Single-center observational	Patients undergoing RFCA of the right bundle for BBRVT.	After ablation, RBBB developed in 15 patients. One patient developed complete heart block, which was anticipated. One patient died of HF 9 months after ablation. Two patients were successfully bridged to heart transplantation at 0.5 and 13 months, respectively, after ablation. Two patients received ICDs for other VTs. One patient had syncope 11 months after ablation, but there was no evidence of VT or heart block in a repeat electrophysiology study. This patient died suddenly 29 months after ablation. The remaining 9 patients were alive and well for a mean follow-up of 19 ± 10 months.		RFCA of the right bundle branch is an effective therapy for treatment of BBRVT.<br><br>This technique could be helpful in management of patients who have unacceptable frequent shocks from their implanted defibrillators.<br><br>In some patients with terminal HF and incessant VT, this procedure can function as a bridge to cardiac transplantation.

Study name or acronym; Author; Year published; PMID	Aim of study; Endpoints; Study type (RCT, observational — multicenter or single center; case series or other [if RCT include intervention and comparator]); Study size (N)	Patient population with inclusion and exclusion criteria	Results (absolute event rates, *P* values; OR or RR; 95% CI)	Other relevant findings or adverse events	Limitations; Other comments; Conclusions
Schmidt et al. Left bundle branch-Purkinje system in patients with bundle branch reentrant tachycardia: lessons from catheter ablation and electroanatomic mapping.<br>• Year published: 2009<br>• PMID: 19121800	Size: 13<br>Single-center observational	Thirteen consecutive male patients (age 62 ± 12 years) with sustained BBR tachycardia were included in the study.	Surface ECG before ablation showed LBBB in 10 patients and a narrow QRS in 3 patients.<br><br>Ablation of the right bundle branch resulted in RBBB on surface ECG in 8 of 9 patients and total AV block with preserved retrograde conduction over the left bundle branch in 1 of 9 patients. The left bundle branch was successfully ablated in another 4 patients.<br><br>During mean follow-up of 48 ± 29 months, 3 patients died, but BBR tachycardia did not recur in any patient.		Catheter ablation is useful.<br><br>Patients with normal heart and BBR tachycardia have a good prognosis and might not require further intervention.<br><br>In patients with LBBB and BBR tachycardia, anterograde slow conduction over the left bundle branch is present. Ablation of the left bundle branch is feasible and could be an alternative approach for BBR tachycardia.

Study name or acronym; Author; Year published; PMID	Aim of study; Endpoints; Study type (RCT, observational — multicenter or single center; case series or other [if RCT include intervention and comparator]); Study size (N)	Patient population with inclusion and exclusion criteria	Results (absolute event rates, *P* values; OR or RR; 95% CI)	Other relevant findings or adverse events	Limitations; Other comments; Conclusions
Blanck et al. Bundle branch reentry: a mechanism of ventricular tachycardia in the absence of myocardial or valvular dysfunction.<br>• Year published: 1993<br>• PMID: 8227845	Size: 3<br>Single-center observational	Three patients underwent noninvasive and invasive cardiac evaluation and electrophysiologic studies to identify the substrate and mechanism of tachycardia. Catheter ablation of the right bundle branch using RF current was performed for each patient.	Electrocardiography during SR revealed nonspecific intraventricular conduction delay in all 3 patients. Cardiac evaluation revealed no evidence of myocardial or valvular dysfunction in any patient. The baseline HV interval was prolonged in each patient. Catheter ablation of the right bundle branch using RF current abolished BBR in all 3 patients. After 26-, 13- and 8-month follow-up periods, complete RBBB persisted, and all 3 patients remained asymptomatic without AADs.		Sustained BBR can be a clinical arrhythmia in patients with no identifiable myocardial or valvular dysfunction except for isolated conduction abnormalities in the HPS.<br><br>RF ablation is effective.
Li et al. Bundle branch reentrant tachycardia in patients with apparent normal His-Purkinje conduction: the role of functional conduction impairment.<br>• Year published: 2002<br>• PMID: 12521339	Size: 13<br>Single-center observational	Patients have BBRVT as the underlying electrophysiologic mechanism.	Of these 13 patients, 6 had an HV interval ≤55 ms (group A), and 7 had a prolonged HV interval (>55 ms; group B) during SR. Successful ablation of the right bundle branch was performed in all 13 patients without deteriorating AV block. Two patients died in each group, and VTs (other than BBRVT) or VF were documented by ICD electrogram storages in 4 patients during follow-up of 27 ± 17 months.		A prolonged HV interval during SR is not a prerequisite for BBRVT. Functional HPS abnormalities appear to be the electrophysiologic substrate for this specific type of BBRVT.<br><br>RF ablation is effective.

Study name or acronym; Author; Year published; PMID	Aim of study; Endpoints; Study type (RCT, observational — multicenter or single center; case series or other [if RCT include intervention and comparator]); Study size (N)	Patient population with inclusion and exclusion criteria	Results (absolute event rates, *P* values; OR or RR; 95% CI)	Other relevant findings or adverse events	Limitations; Other comments; Conclusions
Narasimhan et al. Ventricular tachycardia in valvular heart disease: facilitation of sustained bundle-branch reentry by valve surgery.<br>• Year published: 1997<br>• PMID: 9416897	Size: 31<br>Single-center observational	Some 31 patients (30 men and 1 woman) who had undergone valve surgery were found to have inducible SMVT.	Nine (29%) patients had sustained VT due to BBR (group 1). Group 2 included 20 patients with inducible myocardial (ie, non-BBR) VT. Two patients had both inducible sustained BBR and myocardial VT (group 3). Sustained BBRVT occurred significantly earlier after valve surgery (median, 10 days) than the onset of postoperative myocardial VT (median, 72 months; *P*<.005).<br>In group 1, 8 patients were treated with catheter ablation of the right bundle branch, and 1 patient received an ICD at another institution. The mean follow-up was 30 ± 29 months. Two patients treated with right bundle branch ablation died from progressive HF. The other 7 patients are alive and well. Only 1 patient is on AAD therapy (amiodarone for AF).<br>In group 3, both patients received appropriate ICD discharges for recurrent VT. One patient subsequently underwent cardiac transplantation for progressive HF. Both are alive and well.		Myocardial VT was the most common type of inducible SMVT in patients with valvular heart disease. However, in almost one-third of the patients, sustained BBRVT was the only type of inducible SMVT. This type of VT was facilitated by the valve procedure occurring within 4 weeks after surgery in most patients. Because a curative therapy can be offered to these patients (ie, bundle-branch ablation), BBR should be seriously considered as the VT mechanism in patients with valvular heart disease, particularly if the arrhythmia occurs soon after valve surgery.

Study name or acronym; Author; Year published; PMID	Aim of study; Endpoints; Study type (RCT, observational — multicenter or single center; case series or other [if RCT include intervention and comparator]); Study size (N)	Patient population with inclusion and exclusion criteria	Results (absolute event rates, *P* values; OR or RR; 95% CI)	Other relevant findings or adverse events	Limitations; Other comments; Conclusions
Chen et al.<br>Electrophysiological characteristics of bundle branch reentry ventricular tachycardia in patients without structural heart disease.<br>• Year published: 2018<br>• PMID: 29986947	Size: 9<br>Single-center observational	Nine patients (mean age, 29.6 years) with normal LV function were enrolled. BBRVT with RBBB and LBBB patterns was induced in 1 and 9 patients, respectively.	Ablation was applied in the distal left bundle branch in patients with baseline left BBB and in one narrow QRS patient with sustained Purkinje-related VT, whereas the right bundle branch was targeted in other patients. During a mean follow-up of 31.4 months, frequent PVCs occurred in one patient, and new VT developed in the other patient. Seven patients remained free from VT.		Ablation targeting at the distal left bundle branch, which bifurcates into the left posterior and anterior fascicle, can preserve the residual atrioventricular conduction; however, intensive follow-up is needed.
**Fascicular VT in Adults**					
Liu et al.<br>Catheter ablation of fascicular ventricular tachycardia: long-term clinical outcomes and mechanisms of recurrence.<br>• Year published: 2015<br>• PMID: 26386017	Size: 120<br>Single-center observational	Consecutive 120 patients undergoing IFLVT ablation at a single center were enrolled.	Ablation was acutely successful in 117 patients. With a median follow-up of 55.7 months, VT of a similar ECG morphology recurred in 17 patients, and repeat procedure confirmed FVT recurrence involving the same fascicle. Shorter VT cycle length was the only significant predictor of FVT recurrence (*P*=.03). Six other patients developed new-onset upper septal FVT that was successfully ablated.<br>Ablation of FVT guided by activation mapping is associated with a single procedural success rate without the use of AADs of 80.3%.		Catheter ablation is an effective therapy for treatment of IFLVT.

Study name or acronym; Author; Year published; PMID	Aim of study; Endpoints; Study type (RCT, observational — multicenter or single center; case series or other [if RCT include intervention and comparator]); Study size (N)	Patient population with inclusion and exclusion criteria	Results (absolute event rates, *P* values; OR or RR; 95% CI)	Other relevant findings or adverse events	Limitations; Other comments; Conclusions
Chen et al. Non-contact mapping and linear ablation of the left posterior fascicle during sinus rhythm in the treatment of idiopathic left ventricular tachycardia.<br>• Year published: 2005<br>• PMID: 15763527	Size: 6<br>Single-center observational	Six patients with ILVT, consisting of one case in which conventional mapping failed 3 times, 1 recurrent case, 1 noninducible case, and 3 common cases, were included in the study.	After a mean of 5.5 ± 1.6 RF deliveries, the clinical tachycardias could not be induced, and the 12-lead surface ECG showed right QRS axis deviation (mean 39.7 ± 26.0 degrees) in all patients. The total procedure time was 160.0 ± 32.2 minutes, with a fluoroscopic time of 26.0 ± 6.8 min. No ILVT was inducible during control stimulation, and none recurred during a mean follow-up of 13.0 ± 4.8 months.		Mapping and linear ablation of the Purkinje network in the LPF area guided by noncontact mapping is an effective and safe treatment of ILVT with RF energy, especially for those ILVTs that were unsuccessfully treated by conventional means or were noninducible or nonsustained during the procedure.
Lin et al. Idiopathic fascicular left ventricular tachycardia: linear ablation lesion strategy for noninducible or nonsustained tachycardia.<br>• Year published: 2005<br>• PMID: 16171747	Size: 6<br>Single-center observational	Of 122 consecutive patients who underwent ablation of idiopathic VT from 1999 to 2003, 15 had IFLVT. Six (40%) of the 15 patients had nonsustained or noninducible VT in the EP lab.	A linear ablation lesion strategy was used to treat noninducible or nonsustained IFLVT. Development of LPF block was noted in 2 of the 6 patients. However, despite the absence of development of LPF block in the other 4 patients, no VT or premature ventricular beats could be induced after ablation using the same provocation maneuvers as performed in the baseline state. No spontaneous arrhythmias occurred during follow-up to 16 ± 8 months.		For patients with difficult to induce or nonsustained IFLVT, linear ablation is safe and effective for VT control.

Study name or acronym; Author; Year published; PMID	Aim of study; Endpoints; Study type (RCT, observational — multicenter or single center; case series or other [if RCT include intervention and comparator]); Study size (N)	Patient population with inclusion and exclusion criteria	Results (absolute event rates, *P* values; OR or RR; 95% CI)	Other relevant findings or adverse events	Limitations; Other comments; Conclusions
Nakagawa et al. Radiofrequency catheter ablation of idiopathic left ventricular tachycardia guided by a Purkinje potential.<br>• Year published: 1993<br>• PMID: 8252671	Size: 8<br>Single-center observational	Eight patients (mean age, 26 ± 10 years) with ILVT (cycle length, 346 ± 59 ms) were studied.	During follow-up (1 to 67 months; median, 10.5) ILVT recurred only in the latter patient.		Catheter ablation is an effective therapy for treatment of IFLVT.
Liu et al. Macroreentrant loop in ventricular tachycardia from the left posterior fascicle: new implications for mapping and ablation.<br>• Year published: 2016<br>• PMID: 27635071	Size: 14<br>Single-center observational	Fourteen consecutive patients with LPFVT underwent electrophysiology study and RFCA.	RFCA focused on the P1 potentials (9 patients with a recorded P1) or the earliest P2 (5 patients without a recorded P1) was successful in all 14 patients. After 4.5 ± 3.0 months of follow-up, no patients had recurrence of LPFVT.		Catheter ablation is an effective therapy for treatment of LPFVT.
Guo et al. Clinical, electrocardiographic, and electrophysiological characteristics of left upper septal fascicular ventricular tachycardia.<br>• Year published: 2018<br>• PMID: 28160481	Size: 11<br>Single-center observational	Eleven consecutive patients with LUSVT were identified among 196 patients with left fascicular VT.	Ten patients were managed successfully by 11 ablation sessions, and 1 patient declined ablation. After ablation, 2 (10%) cases developed new left anterior hemiblock or incomplete LBBB. No VT recurred during a median follow-up period of 3.2 (range 1.0–12.7) years.		LUSVT can be managed successfully by focal ablation at the left upper septum with a mild risk of fascicular injury.

Study name or acronym; Author; Year published; PMID	Aim of study; Endpoints; Study type (RCT, observational — multicenter or single center; case series or other [if RCT include intervention and comparator]); Study size (N)	Patient population with inclusion and exclusion criteria	Results (absolute event rates, *P* values; OR or RR; 95% CI)	Other relevant findings or adverse events	Limitations; Other comments; Conclusions
Nogami et al. Demonstration of diastolic and presystolic Purkinje potentials as critical potentials in a macroreentry circuit of verapamil-sensitive idiopathic left ventricular tachycardia.<br>• Year published: 2000<br>• PMID: 10987604	Size: 20<br>Single-center observational	A total of 20 patients with verapamil-sensitive ILVT.	In 15 of 20 patients, both P1 and P2 were recorded during VT from the midseptal region. RFCA was successfully performed at this site in all 15 patients. In the remaining five patients, the diastolic potential could not be detected, and a single fused P2 was recorded only at the VT exit site. Successful ablation was performed at this site in all 5 patients.		This study demonstrates that P1 and P2 are critical potentials in a circuit of verapamil-sensitive ILVT and suggests the presence of a macroreentry circuit involving the normal Purkinje system and the abnormal Purkinje tissue with decremental property and verapamil sensitivity.
Ouyang et al. Electroanatomic substrate of idiopathic left ventricular tachycardia: unidirectional block and macroreentry within the Purkinje network.<br>• Year published: 2002<br>• PMID: 11815429	Size: 9<br>Single-center observational	A total of 9 patients with ILVT and 6 control patients who underwent mapping of the left ventricle during SR using 3D EAM.	ILVT was noninducible in 3 patients after SR mapping. Diastolic potentials critical for ILVT during ILVT coincided with the earliest retroPP during SR in 7 patients. Mechanical termination of ILVT occurred in 5 patients. A single RF pulse was applied at the site with mechanical translation in 5 patients and at the site with diastolic potential in 2 patients; 3 RF pulses were delivered to the site with the earliest retroPP in the other 3 patients without inducible ILVT after SR mapping. No ILVT was inducible during control stimulation, and none was recurred during follow-up of 9.1 ± 5.1 months.		In patients with ILVT, abnormal retroPP within the posterior Purkinje fiber network is a common finding. The earliest retroPP critical for ILVT substrate can be used for guiding successful ablation.

Study name or acronym; Author; Year published; PMID	Aim of study; Endpoints; Study type (RCT, observational — multicenter or single center; case series or other [if RCT include intervention and comparator]); Study size (N)	Patient population with inclusion and exclusion criteria	Results (absolute event rates, *P* values; OR or RR; 95% CI)	Other relevant findings or adverse events	Limitations; Other comments; Conclusions
Kottkamp et al. Idiopathic left ventricular tachycardia: new insights into electrophysiological characteristics and radiofrequency catheter ablation.<br>• Year published: 1995<br>• PMID: 7659584	Size: 5<br>Single-center observational	Five patients (3 males and 2 females, mean age 31 ± 10 years) with ILVT (cycle length 376 ± 72 ms). The patients had a history of recurrent palpitations of 4 ± 1 years and had been treated unsuccessfully with 2 ± 1 AADs.	In 4 of 5 patients, RFCA (median number of pulses 4, range 1–9) resulted in complete abolition of ILVT during a follow-up of 4–43 months (median 10) without AADs.		RFCA was an effective and safe treatment modality in most of these patients.
Tada et al. Retrograde Purkinje potential activation during sinus rhythm following catheter ablation of idiopathic left ventricular tachycardia.<br>• Year published: 1998<br>• PMID: 9835267	Size: 2<br>Single-center observational	Two patients with ILVT that were cured by RFCA.	Two distinct presystolic potentials (P1 and P2) were recorded during tachycardia in the midseptal or inferoapical area, but only one potential (P2) was recorded during SR. After catheter ablation at this site, the P1 potential was noted after the QRS complex during SR, whereas the P2 was still observed before the QRS complex. The P1 potential showed a decremental property during atrial or ventricular pacing.		These data suggest that Purkinje tissue with decremental properties was responsible for the tachycardia mechanism, and that the reentry circuit involving this tissue is likely to be of considerable size.

Study name or acronym; Author; Year published; PMID	Aim of study; Endpoints; Study type (RCT, observational — multicenter or single center; case series or other [if RCT include intervention and comparator]); Study size (N)	Patient population with inclusion and exclusion criteria	Results (absolute event rates, *P* values; OR or RR; 95% CI)	Other relevant findings or adverse events	Limitations; Other comments; Conclusions
Tsuchiya et al. Significance of late diastolic potential preceding Purkinje potential in verapamil-sensitive idiopathic left ventricular tachycardia.<br>• Year published: 1999<br>• PMID: 10318662	Size: 16<br>Single-center observational	A total of 16 consecutive patients with this specific VT were studied (12 men and 4 women; mean age, 32 years).	In 3 patients, the pressure applied to the catheter tip at the late diastolic potential region resulted in conduction block between late diastolic potential and PP, and in VT termination. RF energy application at the late diastolic potential recording site successfully eliminated VT.		Late diastolic potential can be a useful marker for successful RFCA for this VT.
Wen et al. Successful radiofrequency ablation of idiopathic left ventricular tachycardia at a site away from the tachycardia exit.<br>• Year published: 1997<br>• PMID: 9316534	Size: 27<br>Single-center observational	A total of 27 consecutive patients with verapamil-responsive ILVT.	The potential ablation site, other than the tachycardia exit site, was identified in 7 male patients (mean [± SD] age 31 ± 12 years, range 13–52). Application of the RF current at this site resulted in termination of the tachycardia within 1 to 5 seconds (mean 2.9 ± 1.6), and successful ablation of the tachycardia was achieved in all 7 patients (success rate 100%, 95% exact CI 0.5898–1).		Successful ablation of ILVT can be achieved at sites away from the tachycardia exit site in some patients.

Study name or acronym; Author; Year published; PMID	Aim of study; Endpoints; Study type (RCT, observational — multicenter or single center; case series or other [if RCT include intervention and comparator]); Study size (N)	Patient population with inclusion and exclusion criteria	Results (absolute event rates, *P* values; OR or RR; 95% CI)	Other relevant findings or adverse events	Limitations; Other comments; Conclusions
Arya et al. Comparison of presystolic Purkinje and late diastolic potentials for selection of ablation site in idiopathic verapamil sensitive left ventricular tachycardia.<br>• Year published: 2004<br>• PMID: 15383777	Size: 15<br>Single-center observational	Between June 2002 and February 2004, 15 patients (12 men; age 28 ± 11 years, range 12–51) with ILVT underwent RFCA.	Fewer applications were needed in those for whom RFCA was initially targeted to the PP (with or without diastolic potential) recording site (10 patients, 4.7 ± 1.8) compared with those targeted to the diastolic potential recording site (5 patients, 12.2 ± 3.3) (*P*<.05).		Compared to diastolic potential alone, earliest PP (with or without concomitant diastolic potential) might be superior for selection of RFCA target site in patients with ILVT.
**Fascicular VT in Pediatric Patients**					
Collins et al. Fascicular and nonfascicular left ventricular tachycardias in the young: an international multicenter study.<br>• Year published: 2013<br>• PMID: 23437865	Size: 129<br>Multicenter observational	This international multicenter retrospective study including 152 patients (age 10.0 ± 5.1 years, 62% male), divided into those with fascicular VT (85%, 129 of 152) and nonfascicular LV VT (15%, 23 of 152).	In fascicular VT, calcium channel blockers were effective in 80% (74 of 92); however, when administered orally, there was a 21% (13 of 62) recurrence rate. Ablation procedures were successful in 71% (72 of 102) of fascicular VT. After a follow-up period of 2 years (1 day to 15 years), 72% of all patients with fascicular VT were off medications with no tachycardia recurrence. One patient died of noncardiac causes.		Catheter ablation procedures can be curative for pediatric patients with fascicular VT.

Study name or acronym; Author; Year published; PMID	Aim of study; Endpoints; Study type (RCT, observational — multicenter or single center; case series or other [if RCT include intervention and comparator]); Study size (N)	Patient population with inclusion and exclusion criteria	Results (absolute event rates, *P* values; OR or RR; 95% CI)	Other relevant findings or adverse events	Limitations; Other comments; Conclusions
Suzuki et al. Radiofrequency catheter ablation of idiopathic left anterior fascicular ventricular tachycardia in children.<br>• Year published: 2014<br>• PMID: 24954241	Size: 6<br>Single-center observational	Of 537 pediatric cases of RFCA, 6 had IVT-LAF; 4 had anterior fascicular involvement only, whereas 2 had both anterior and posterior fascicular involvement. All 6 of them underwent RFCA at the median age of 8.8 years (range 4.3–14.3 years).	RFCA was successful in all patients, but 4 had recurrence and underwent 1–3 additional sessions of RFCA. In a total of 10 RFCA sessions, the overall recurrence rate was 50%. During the median follow-up period of 33 months, no further recurrence was reported except for 1 patient, who had a recurrence and was scheduled for additional sessions at the time of this report. Major complications included 1 case of complete AV block and 1 case of complete LBBB.		Despite a high recurrence rate and a few complications, RFCA at the site of isolated delayed potential or diastolic potential, if applied cautiously, is a possible treatment of choice for pediatric IVT-LAF.
Fishberger et al. Creation of partial fascicular block: an approach to ablation of idiopathic left ventricular tachycardia in the pediatric population.<br>• Year published: 2015<br>• PMID: 25469902	Size: 6<br>Single-center observational	Six patients (aged 3–17 years) with ILVT, 5 originating from the posterior fascicle and one from the anterior fascicle.	All patients had a QRS axis shift following ablation, although none met criteria for fascicular block. At follow- up (7–49 months, mean 27 months), all patients had persistence of this shift. There were no recurrences of VT and none of the patients were taking AADs.		The technique of creating partial fascicular block appears to be a safe and effective approach to ablation of ILVT in children.

Study name or acronym; Author; Year published; PMID	Aim of study; Endpoints; Study type (RCT, observational — multicenter or single center; case series or other [if RCT include intervention and comparator]); Study size (N)	Patient population with inclusion and exclusion criteria	Results (absolute event rates, *P* values; OR or RR; 95% CI)	Other relevant findings or adverse events	Limitations; Other comments; Conclusions
**Fascicular VT in Neonates**					
Ozer et al. Adenosine- and verapamil-sensitive ventricular tachycardia in the newborn.<br>• Year published: 2001<br>• PMID: 11388113	Size: 2<br>Single-center observational	Two neonates presented with sustained, monomorphic VT.	Transesophageal electrophysiological studies demonstrated that the VTs were initiated with burst atrial pacing in one and were noninducible in the other; both terminated with burst atrial pacing and adenosine. Oral verapamil suppressed the VTs in both. Following discontinuation of verapamil at 1 year of age, both children remain free of tachycardia recurrence at 3 and 4 years of age.		Infants with ILVT frequently experience resolution with time; catheter ablation might be not necessary.
**Focal Fascicular VT**					
Talib et al. Non-reentrant fascicular tachycardia: clinical and electrophysiological characteristics of a distinct type of idiopathic ventricular tachycardia.<br>• Year published: 2016<br>• PMID: 27729344	Size: 15<br>Single-center observational	Among 530 idiopathic VT patients who were referred for ablation, we identified 15 (2.8%) with nonreentrant fascicular tachycardia (11 men, 45 ± 21 years).	VT recurrence was observed in 4 (27%) patients; among these, 3 underwent pace map-guided ablation during the first session. A second ablation with activation mapping guidance eliminated the VT during the 88- ± 8-month follow-up.		Among idiopathic VT cases referred for ablation, 2.9% were focal nonreentrant fascicular tachycardia. Catheter ablation is effective, whereas a pace map-guided approach is less efficacious.

Study name or acronym; Author; Year published; PMID	Aim of study; Endpoints; Study type (RCT, observational — multicenter or single center; case series or other [if RCT include intervention and comparator]); Study size (N)	Patient population with inclusion and exclusion criteria	Results (absolute event rates, *P* values; OR or RR; 95% CI)	Other relevant findings or adverse events	Limitations; Other comments; Conclusions
Lopera et al. Identification and ablation of three types of ventricular tachycardia involving the His-Purkinje system in patients with heart disease.<br>• Year published: 2004<br>• PMID: 15028072	Size: 2<br>Single-center observational	Involvement of the HPS was sought during electrophysiologic study with catheter mapping in 234 consecutive patients referred for catheter ablation of recurrent VT associated with heart disease. HPS VT was observed in 20 (8.5%) patients. Two patients had VT consistent with a focal origin in the distal HPS.	Lopera et al. reported two focal Purkinje VT cases with IHD in whom complete AV block occurred after the successful ablation of the VT.		Ablation often is not sufficient as the sole therapy due to other induced VTs and conduction abnormalities, requiring pacemaker and/or defibrillator implantation.
Gonzalez et al. Clinical and electrophysiologic spectrum of fascicular tachycardias.<br>• Year published: 1994<br>• PMID: 8017268	Size: 8<br>Single-center observational	Eight patients with fascicular tachycardia, defined as tachycardia with an HV interval during tachycardia less than that of the HV of conducted impulses.	In 3 patients, entrainment as well as the ability to initiate and terminate the tachycardia favored a reentrant mechanism. In others, tachycardia initiation only over a critical range of paced cycle lengths and the incessant nature of the tachycardia or the presence of other atrial or ventricular foci favored a mechanism of either abnormal automaticity or triggered rhythms. Catheter ablation was successful in 2 of 5 patients in whom it was attempted.		A variety of mechanisms and different foci might be associated with FT. Selected individuals might respond to catheter ablative therapy.

Study name or acronym; Author; Year published; PMID	Aim of study; Endpoints; Study type (RCT, observational — multicenter or single center; case series or other [if RCT include intervention and comparator]); Study size (N)	Patient population with inclusion and exclusion criteria	Results (absolute event rates, *P* values; OR or RR; 95% CI)	Other relevant findings or adverse events	Limitations; Other comments; Conclusions
Nogami. Purkinje-related arrhythmias part I: monomorphic ventricular tachycardias.<br>• Year published: 2011<br>• PMID: 21410719	Size: 14<br>Single-center observational	A total of 14 patients with idiopathic focal Purkinje VT.	In all 14 patients, the VT and ventricular premature complexes were suppressed by the catheter ablation; however, the true success rate is unclear because this VT is difficult to be induced. Therefore, the recurrence rate is high (29%). LPF block occurred in two patients after the ablation; however, no AV block occurred in any of the patients.		
**Purkinje Fiber-Mediated VT**					
Hayashi et al. Novel mechanism of postinfarction ventricular tachycardia originating in surviving left posterior Purkinje fibers.<br>•Year published: 2006<br>• PMID: 16876739	Size: 4<br>Single-center observational	Four patients who had reentrant VT arising from the left posterior Purkinje fibers in patients with a prior MI.	RF energy delivered at the site exhibiting a Purkinje-QRS interval of 58 ± 26 ms successfully eliminated the VTs without provoking any conduction disturbances.		Reentrant monomorphic VT originating from the left posterior Purkinje fibers, which is analogous to ILVT, can develop in the acute or chronic phase of MI. Catheter ablation is highly effective in eliminating this VT without affecting LV conduction.

Study name or acronym; Author; Year published; PMID	Aim of study; Endpoints; Study type (RCT, observational — multicenter or single center; case series or other [if RCT include intervention and comparator]); Study size (N)	Patient population with inclusion and exclusion criteria	Results (absolute event rates, *P* values; OR or RR; 95% CI)	Other relevant findings or adverse events	Limitations; Other comments; Conclusions
Bogun et al. Role of Purkinje fibers in post-infarction ventricular tachycardia.<br>• Year published: 2006<br>• PMID: 17174189	Size: 9<br>Single-center observational	From among a group of 81 consecutive patients with post-infarction monomorphic VT referred for catheter ablation, 9 patients were identified in whom the clinical VT had a QRS duration ≤145 ms.	Mapping during VT demonstrated reentry involving the inferior LV wall. In each of the VTs, a PP was present at the exit site of the VT reentry circuit. Single RFCA lesions were successful in eliminating these VTs in all patients.		The Purkinje system might be part of the reentry circuit in patients with post-infarction monomorphic VT, resulting in a type of VT with a relatively narrow QRS complex that mimics fascicular VT. Catheter ablation is effective.
**Premature Ventricular Complex from Either Right or Left Fascicular Tissue**					
Haïssaguerre et al. Role of Purkinje conducting system in triggering of idiopathic ventricular fibrillation.<br>• Year published: 2002<br>• PMID: 11879868	Size: 16<br>Single-center observational	Sixteen patients were investigated by electrography and RFCA after resuscitation from recurrent idiopathic VF.	The accuracy of mapping was confirmed by acute elimination of triggers by RF delivery, and there was no recurrence of VF in 14 patients.		Long-term follow-up is necessary to establish that ablation is curative and avoids use of a defibrillator.

Study name or acronym; Author; Year published; PMID	Aim of study; Endpoints; Study type (RCT, observational — multicenter or single center; case series or other [if RCT include intervention and comparator]); Study size (N)	Patient population with inclusion and exclusion criteria	Results (absolute event rates, *P* values; OR or RR; 95% CI)	Other relevant findings or adverse events	Limitations; Other comments; Conclusions
Knecht et al. Long-term follow-up of idiopathic ventricular fibrillation ablation: a multicenter study.<br>• Year published: 2009<br>• PMID: 19643313	Size: 38<br>Multicenter observational	From January 2000, 38 consecutive patients from 6 different centers underwent ablation of primary idiopathic VF initiated by short coupled VPB.	Triggering VPBs originated from the right (n=16), the left (n=14), or both (n=3) Purkinje systems and from the myocardium (n=5). During a median postprocedural follow-up of 63 months, 7 (18%) of 38 patients experienced VF recurrence at a median of 4 months. Five of these 7 patients underwent repeat ablation without VF recurrence. Survival free of VF was predicted only by transient BBB in the originating ventricle during the electrophysiological study (*P*<.0001). The number of significant events (confirmed VF or aborted sudden death) was reduced from 4 (IQR 3–9) before to 0 (IQR 0–4) after ablation (*P*=.01).		Ablation for idiopathic VF that targets short coupled VPB triggers is associated with a long-term freedom from VF recurrence.

Congenital Heart Disease

Study name or acronym; Author; Year published; PMID	Aim of study; Endpoints; Study type (RCT, observational — multicenter or single center; case series or other [if RCT include intervention and comparator]); Study size (N)	Patient population with inclusion and exclusion criteria	Results (absolute event rates, *P* values; OR or RR; 95% CI)	Other relevant findings or adverse events	Limitations; Other comments; Conclusions
Gatzoulis et al. Mechano-electric interaction in tetralogy of Fallot: QRS prolongation relates to right ventricular size and predicts malignant ventricular arrhythmias and sudden death.<br>• Year published: 1995<br>• PMID: 7600655	Aim: To identify ECG risk factors for SCD in patients with repaired ToF<br>Endpoints: SCD<br>Study type: Single-center retrospective<br>Size: 41	Inclusion criteria: ToF survivors	Of 178 patients, 41 were evaluated serially and reviewed for SCD. QRS duration correlated with RV size on ECG and heart size on chest X-ray. VT was found in 9 patients: mean QRS 199 ms, CTR .67; significantly different from those without VT. Chronic RV volume overload related to diastolic dysfunction.	All patients with ToF with documented SVT and patients with SCD had QRS duration ≥180 ms (100% sensitivity).	ToF with QRS duration ≥180 ms predicts VT and SCD.
Gonska et al. Radiofrequency catheter ablation of right ventricular tachycardia late after repair of congenital heart defects.<br>• Year published: 1996<br>• PMID: 8873666	Aim: VT Ablation results repaired CHD.<br>Endpoints: Noninducible VT<br>Study type: Observational case series<br>Size: 16	Inclusion criteria: SVT (n=8) or NSVT (n=7) late after CHD repair (15), 1 asymptomatic. There were 9 ToF, 4 VSD, 2 PS, and 1 each TGA and VSD. Mean age 34 ± 13 years, postoperative interval 28 (11–42) years.	Right VT induced in all, sustained in 11, nonsustained in 5. Ablation at site of earliest activation or good pace map; VT CL 377 ms, hemodynamically stable.<br><br>Acutely successful in 96% (15 of 16); repeat EPS 5–7 days later, noninducibility in 14 (88%). Some 11 patients had no medications.<br><br>Follow-up at 16 ± 9 months, recurrence was 13% (2/15).	No RVH or hemodynamic abnormalities.<br><br>Demonstrated inducibility of clinical VT.<br><br>No SCD	Early endocardial activation not predictive of success.<br><br>Ablation approximately 85% successful.<br><br>No SCD.

Study name or acronym; Author; Year published; PMID	Aim of study; Endpoints; Study type (RCT, observational — multicenter or single center; case series or other [if RCT include intervention and comparator]); Study size (N)	Patient population with inclusion and exclusion criteria	Results (absolute event rates, *P* values; OR or RR; 95% CI)	Other relevant findings or adverse events	Limitations; Other comments; Conclusions
Morwood et al. Radiofrequency catheter ablation of ventricular tachycardia in children and young adults with congenital heart disease.<br>• Year published: 2004<br>• PMID: 15851174	Aim: Ablation of VT in a total of 62 young patients<br>Endpoints: Successful ablation of SVT<br>Study type: Observational, case series, single center, Boston Children's 1990–2003.<br>Size: 14 patients with CHD (40 structurally normal, 8 cardiomyopathy)	Inclusion criteria: 14 of 62 patients had CHD: 8 ToF, 3 VSD, 1 Ebstein, 1 single ventricle, 1 aortic stenosis<br><br>Mean age 21.6 years (range 0.1–47 years)	Acute success of ablation: 43% intention to treat; 60% success in mappable VT in safe location; 1.4 ± 0.6 procedures per patient; recurrence 40% during mean follow-up of 3.8 years.	Unstable VT, limited access in 36%; no inducible VT in 14%. (One postprocedural death in neonate with Ebstein).	Lower success rate and higher recurrence compared with patients with no CHD (>87% acute success, 30%–43% recurrence).
Furushima et al. Ventricular tachycardia late after repair of congenital heart disease: efficacy of combination therapy with radiofrequency catheter ablation and class III antiarrhythmic agents and long-term outcome.<br>• Year published: 2006<br>• PMID: 16580423	Aim: Rx VT in repaired CHD<br>Endpoints: No recurrent VT, ablation and drugs<br>Study type: Observational, case series<br>Size: 7	Inclusion criteria: 7 consecutive patients with stable monomorphic SVT; mean age 25 ± 7 years (16–35 years); 4 ToF, 3 DORV; postoperative 18 years (9–27 years); mean 2.8 AADs per patient prior to ablation.	RV VT induced, mean CL 346 ms. Ablation of 14 VTs per 7 patients attempted; acute success 50% of VTs, although 6 of 7 patients with inducible VT at EPS 1–2 weeks later; all 7 prescribed AADs (amiodarone, sotalol); 1 ICD. No recurrence on medications for 61 ± 29 months.	Multiple reentrant circuits of inducible VT, 3 circuits in RVOT, 4 RV septal surface.	Low success rates of ablation.<br><br>Note no recurrent VT, no SCD on AADs.

Study name or acronym; Author; Year published; PMID	Aim of study; Endpoints; Study type (RCT, observational — multicenter or single center; case series or other [if RCT include intervention and comparator]); Study size (N)	Patient population with inclusion and exclusion criteria	Results (absolute event rates, *P* values; OR or RR; 95% CI)	Other relevant findings or adverse events	Limitations; Other comments; Conclusions
Kriebel et al. Noncontact mapping and radiofrequency catheter ablation of fast and hemodynamically unstable ventricular tachycardia after surgical repair of tetralogy of Fallot.<br>• Year published: 2007<br>• PMID: 18036455	Aim: Efficacy of VT ablation in patients with ToF<br>Endpoints:<br>Study type: Observational, case series<br>Size: 10	Inclusion criteria: Repaired ToF, 4 NSVT, 4 SVT, 2 ICD discharges; noncontact mapping using EnSite; mean age 29 years	A total of 13 VTs in 10 patients. Macroreentrant in 11 of 13, focal in 2. RFCA attempted in 8, during SR, 100% success, 80% total. Targeted shortest isthmus of macroreentry, 10–40 mm; focal 5–8 mm length. Recurrence 20% during 35 months follow-up: different CL or morphology. ICD recommended for all: implanted in 3 postablation.	Two patients had multiple VTs.<br><br>Sites: Right ventricle in all: 2 anterior free wall, 2 TVA-RVOT; 4 RVOT; 3 VSD patch-TVA; 1 VSD patch-free wall.<br><br>Two VTs near the His bundle were not attempted; mean VT CL 269 ms.	Substrate-based ablation for fast VT with 80% success.
Zeppenfeld et al. Catheter ablation of ventricular tachycardia after repair of congenital heart disease: electroanatomic identification of the critical right ventricular isthmus.<br>• Year published: 2007<br>• PMID: 17967973	Aim: To identify anatomic isthmus of unexcitable VT tissue in patients with repaired CHD<br>Endpoints: Abolish VT<br>Study type: Observational, Two centers, Boston and Leiden, case series<br>Size: 11	Inclusion: Clinical SVT after repair of CHD<br><br>Mean age 43.7 ± 10.6 years<br><br>There were 9 ToF, 1 TGA/VSD, and 1 AVSD<br><br>Postoperative interval 33 years	Voltage map during sinus; entrainment during VT.<br><br>Major sites of anatomic barriers: TVA to RVOT patch (11), RV incision to PV (4); VSD patch to PV (10) or to TAs (3).<br><br>RFCA during VT or sinus (5), target isthmus to connect boundaries. Acute success 100%. No complications. AADs in 5 of 11 maintained (amiodarone 2, sotalol 3); ICD in 5. No documented VT during mean 30 months follow-up, although 1 patient had palpitations and inducible VT.	Moderate-severe RV dilatation in 4 of 11, 2 with PR moderate-severe; LVEF 50%–67%; Mean QRS duration ToF 154 ± 44 ms (100–220); mean VT CL 276 ms.	Map-guided ablation using anatomic isthmus approach highly successful.

Study name or acronym; Author; Year published; PMID	Aim of study; Endpoints; Study type (RCT, observational — multicenter or single center; case series or other [if RCT include intervention and comparator]); Study size (N)	Patient population with inclusion and exclusion criteria	Results (absolute event rates, *P* values; OR or RR; 95% CI)	Other relevant findings or adverse events	Limitations; Other comments; Conclusions
Knauth et al. Ventricular size and function assessed by cardiac MRI predict major adverse clinical outcomes.<br>• Year published: 2008<br>• PMID: 17135219	Aim: To correlate cardiac MRI findings with MACE during follow-up<br>Endpoints: Death, SVT, NYHA Class III–IV<br>Study type: Single center, retrospective<br>Size: 88	Inclusion criteria: Patients with repaired ToF undergoing cardiac MRI	Median postoperative interval 21 years. MACE: 20.5%: death 5%, SVT 10%, worsening NYHA class 11% QRS duration ≥180 ms correlated with RV size.	ToF adverse outcomes predictors:<br>RVEDV z score ≥7, OR 4.55;<br>LVEF <55%, OR 8.05;<br>RVEF <45%;<br>QRS duration ≥180 ms	Patients with ToF with ventricular dilatation, decreased function, or QRS prolongation are at increased risk for adverse events.
Koyak et al. Sudden cardiac death in adult congenital heart disease.<br>• Year published: 2012<br>• PMID: 22991410	Aim: To identify types of CHD associated with SCD<br>Endpoints: SCD<br>Study type: Multicenter, retrospective case control<br>Size: 213	Inclusion: Database of 25,790 patients with ACHD identified 1189 deaths; of these, 213 patients died suddenly	Arrhythmic deaths occurred in 171 of 1189 patients with SCD. Patients with SCD were assessed for diagnosis, age, sex, and type of surgical repair. Some 5% of the 1189 deaths were sudden. Incidence of SCD varied by complexity of heart disease: mild, 12%; moderate, 33%; and severe, 55% of SCD cases. Variables associated with SCD: SVT (OR 3.5); moderate to severe systemic ventricular dysfunction (OR 3.4); and moderate to severe subpulmonary ventricular dysfunction (OR 3.4), QRS prolongation (OR 1.34 per 10 ms increase), and QT dispersion (OR 1.22).	Arrhythmic causes of SCD in 14% of the patients with ACHD. For 37 patients with rhythm documentation at time of death: VF 62%, VT 11%, VT and VF 11%, SVT 8%, bradycardia 8%. For all patients, mean age at death was 36 ± 15 years; 38% had prior symptoms of HF; 17% had other prior symptoms.	Risk of SCD increases with SVT, ventricular dysfunction, QRS prolongation, and severity of CHD.

Study name or acronym; Author; Year published; PMID	Aim of study; Endpoints; Study type (RCT, observational — multicenter or single center; case series or other [if RCT include intervention and comparator]); Study size (N)	Patient population with inclusion and exclusion criteria	Results (absolute event rates, *P* values; OR or RR; 95% CI)	Other relevant findings or adverse events	Limitations; Other comments; Conclusions
Diller et al. Left ventricular longitudinal function predicts life threatening ventricular arrhythmia and death in adults with repaired tetralogy of Fallot.<br>• Year published: 2012<br>• PMID: 22496160	Aim: Does ventricular function correlate with risk of adverse events in ToF?<br>Endpoints: ACA/SCD, SVT, appropriate ICD shock<br>Study type: Single center retrospective<br>Size: 413	Inclusion: Patients with repaired ToF undergoing echocardiographic evaluations	There was 4.6% SVT/SCD/ACA (SCD 1.2%, SVT, 2.2%, ICD shock 1.2%). Combination echo variables correlate with poor outcome: RA area, RV fractional area change, LV global longitudinal strain, mitral annular systolic excursion.	ToF: SVT/SCD1.2/ACA 4.6%	LV longitudinal function associated with greater risk of SCD or VT in patients with repaired ToF.
Miyazaki et al. Efficacy of hemodynamic-based management of tachyarrhythmia after repair of tetralogy of Fallot<br>• Year published: 2012<br>• PMID: 22893279	Aim: Hemodynamic rx of arrhythmias after repaired ToF<br>Endpoints: No recurrent VT<br>Study type: Observational, single-center series, Japan<br>Size: 21 patients with VT (total 66)	Inclusion criteria: NSVT (16), SVT (4); induced VT 1; mean age 23 years	Ablation in 20 patients: ? VT or SVT?<br><br>Five patients underwent PVR ± RVOT resection, ± TV replacement. Incidence of arrhythmia types: VT 32%, SVT 44%, SVT + VT 24%.	Study used medicines, ablation, pacers or ICDs, or surgery; not distinguished between SVT or VT.<br><br>Ablation ± meds controlled 82% of patients; surgery or catheter rx controlled 71%.<br><br>There was 1 late SCD in a patient with NSVT and RVOT obstruction, and 2 SCDs in SVT.	Emphasizes the need to evaluate hemodynamics to decide whether to perform ablation or recommend surgery.

Study name or acronym; Author; Year published; PMID	Aim of study; Endpoints; Study type (RCT, observational — multicenter or single center; case series or other [if RCT include intervention and comparator]); Study size (N)	Patient population with inclusion and exclusion criteria	Results (absolute event rates, *P* values; OR or RR; 95% CI)	Other relevant findings or adverse events	Limitations; Other comments; Conclusions
Tokuda et al. Catheter ablation of ventricular tachycardia in nonischemic heart disease.<br>• Year published: 2012<br>• PMID: 22942218	Aim: VT ablation in nonischemic heart disease<br>Endpoints: Primary: death or transplantation; secondary: death, transplantation, or readmission with recurrent VT within <1 year of discharge<br>Study type: Single center, observational, case series<br>Size: 16 of 891 patients with CHD	Inclusion criteria: 891 consecutive patients undergoing ablation for VT without IHD; 16 prior repairs of CHD (1.7% of series): 8 ToF, 1 DORV, 1 PS, 3 VSD, 1 TGA, and 2 "other"; mean age 45 ± 15 years	In patients with CHD, the number of induced VTs was 2.5 ± 1.5; 69% with at least one unmappable VT; epicardial ablation in 1.<br><br>Acute success 75%, modified 13%, one major complication. Freedom from death or OHT approximately 90%; secondary: approximately 90%. ICD in 63% (10).	Primary outcomes: ARVC > CHD > DCM	Most patients included in report of Zeppenfeld Circ 2007
Kapel et al. Left-sided ablation of ventricular tachycardia in adults with repaired tetralogy of Fallot: a case series.<br>• Year published: 2014<br>• PMID: 25151630	Aim: Ablation for VT in repaired ToF from left ventricle<br>Endpoints: VT recurrence<br>Study type: Two centers: Leiden, Boston; observational, case series<br>Size: 4	Inclusion criteria: 4 of 28 consecutive patients with ToF undergoing VT ablation: failed right-sided ablation, mapped part of circuit to left ventricle, left-sided ablation prevented VT induction	Ablation successful in 4 of 4 patients: 3 in aortic cusp; 1 at left septal surface in proximity to His bundle (complete AV block).<br><br>Recurrence 25%, successfully ablated. No further recurrences during follow-up of 20 ± 15 months.	Right-sided ablation failure likely due to septal hypertrophy in 2, overlying pulmonary homograft in 1, overlying VSD patch in 1. Complete heart block in 1 patient with prior ICD.	Left-sided ablation for VT dependent on septal anatomic isthmus improves outcome in repaired ToF.

Study name or acronym; Author; Year published; PMID	Aim of study; Endpoints; Study type (RCT, observational — multicenter or single center; case series or other [if RCT include intervention and comparator]); Study size (N)	Patient population with inclusion and exclusion criteria	Results (absolute event rates, *P* values; OR or RR; 95% CI)	Other relevant findings or adverse events	Limitations; Other comments; Conclusions
Lin et al. Major adverse cardiovascular events in adult congenital heart disease: a population-based follow-up study from Taiwan.<br>• Year published: 2014<br>• PMID: 24655794	Aim: To identify MACE in patients with ACHD in Taiwan<br>Endpoints: MACE: myocardial infarction, heart failure, percutaneous cardiac intervention, CA bypass grafting, malignant dysrhythmia, cardiac chock, ICD implant, death<br>Study type: National database review with control population<br>Size: 3267	Inclusion criteria: Adults with CHD identified between 2000 and 2003 from the National Health Insurance Research Database 1997–2010; median follow-up 11 years, until end of 2010	Most common types of heart disease: atrial septal defects, ventricular septal defects, patent ductus arteriosus, ToF, and pulmonary stenosis.<br>Incidence of MACE: 4-fold higher in patients with ACHD. After adjustment for age and sex, patients with ACHD had increased risk of heart failure, malignant dysrhythmia, coronary syndrome, and stroke.	Heart failure more common than arrhythmia. Patients with cyanotic heart disease have higher risk of MACE than noncyanotic.	Patients with ACHD had decreased lifelong risk of MACE if they received surgical correction.

Study name or acronym; Author; Year published; PMID	Aim of study; Endpoints; Study type (RCT, observational — multicenter or single center; case series or other [if RCT include intervention and comparator]); Study size (N)	Patient population with inclusion and exclusion criteria	Results (absolute event rates, *P* values; OR or RR; 95% CI)	Other relevant findings or adverse events	Limitations; Other comments; Conclusions
Kapel et al. Re-entry using anatomically determined isthmuses: a curable ventricular tachycardia in repaired congenital heart disease.<br>• Year published: 2015<br>• PMID: 25422392	Aim: Efficacy of isthmus block in ablation of VT in patients with repaired CHD<br>Endpoints: Noninducibility of VT and transection of AI<br>Study type: Observational, 2 centers (Leiden, Boston)<br>Size: 34 (9 patients included in prior 2007 report)	Inclusion criteria: Consecutive patients with repaired CHD undergoing ablation of VT aiming to target AI between 2001 and 2012. Mean age 49 ± 13 years, 74% male; median age of repair 10 years; ToF 28, TGA 2, VSD 2, PS 1, AVSD 1	Preserved LV and RV function: 65%.<br><br>Procedural acute success 74% (25 of 34).<br><br>ICD implanted in 18 of 25 with successful ablations; 7 of 18 had ICD complications.<br><br>No VT recurrence; however, there was 1 VF episode in a patient with poor ventricular function. VT recurred in 44% (4 of 9 patients) without successful ablation. Mean follow-up 46 ± 29 months.<br><br>Majority of VT used either a TA-RVOT patch or VSD patch to the PV.	ICD complications 39%. Due to these complications, consider no ICD implantation in those with procedural success and preserved ventricular function.<br><br>VT CL 295 ms median (IQR 242–346)<br><br>No patient with procedural success and preserved cardiac function experienced any VA.	For patients with repaired CHD, preserved ventricular function, and isthmus-dependent VT, VT isthmus ablation can be curative.

Study name or acronym; Author; Year published; PMID	Aim of study; Endpoints; Study type (RCT, observational — multicenter or single center; case series or other [if RCT include intervention and comparator]); Study size (N)	Patient population with inclusion and exclusion criteria	Results (absolute event rates, *P* values; OR or RR; 95% CI)	Other relevant findings or adverse events	Limitations; Other comments; Conclusions
van Zyl et al. Mechanism and outcomes of catheter ablation for ventricular tachycardia in adults with repaired congenital heart disease.<br>• Year published: 2016<br>• PMID: 26961296	Aim: To evaluate efficacy of isthmus block for VT ablation in CHD<br>Endpoints: Primary: in hospital arrhythmic death; SCD, or appropriate ICD therapy<br>Study type: Single center observational, Mayo<br>Size: 21	Inclusion criteria: 21 consecutive patients with repaired CHD undergoing ablation for VT 2004–2015. Mean ages 45 ± 3 years, 72% male; 10 ToF, 4 TGA, 2 VSD, 2 Ebstein, 3 other; 52% with ICD prior to referral. There were 10 SVT; 1 syncope, 4 presyncope, and 6 palpitations. ICD was implanted in 11.	Macroreentry VT through an electroanatomic isthmus 67% (14 of 21); focal 33%. Isthmus conduction block confirmed in 8 of 14 patients (57%).<br><br>Acute success 81% (17 of 21).<br>In patients with confirmed isthmus block: no recurrent VT.<br><br>Recurrence 15% at follow-up of 33 ± 7 months; all 3 had noninducible VT postablation. ICD implanted in 4 (total 15).	Definition of complete procedural success differs between studies; here, it was noninducibility.	Unexpectedly high percentage of focal arrythmias, details of mapping results not provided.<br><br>Confirms importance of isthmus block as endpoint.
Laredo et al. Ten-year outcomes of monomorphic ventricular tachycardia catheter ablation in repaired tetralogy of Fallot.<br>• Year published: 2017<br>• PMID: 28347634	Aim: Outcome of ablation for VT in patients with repaired ToF<br>Endpoints: Primary: VT termination during ablation and noninducibility; secondary: death, arrhythmia recurrence<br>Study type: Observational series, single center, Paris<br>Size: 34	Inclusion criteria: 34 consecutive patients with repaired ToF undergoing ablation for symptomatic VT 1990–2012. Postoperative interval 21 ± 10 years. Mean age at ablation 31 ± 10 years.	RV dysfunction in 35%, LV dysfunction EF <60% in 21%. Moderate-severe PR in 29%, moderate-severe TR in 12%.<br><br>Mean LVEF 59% ± 9%. Mean of 2 inducible VTs per patient. Anatomic isthmus targeted in 13 patients. Repeat ablation within 3 months performed in 21%.<br>Mean follow-up 9.5 ± 5 years.	Ablation sites: RVOT 79%, mid-anterior RV free wall 21%.<br><br>Late deaths: 2 VF, both with poor EF.<br><br>8 patients with ICD’s, comps in 3 of 8.	Only study with long follow-up. Baseline LVEF <60% associated with arrhythmia recurrence, HR 16.4 (1.8–1.47, *P*=.01).<br><br>No SCD in patients with preserved biventricular function.

Study name or acronym; Author; Year published; PMID	Aim of study; Endpoints; Study type (RCT, observational — multicenter or single center; case series or other [if RCT include intervention and comparator]); Study size (N)	Patient population with inclusion and exclusion criteria	Results (absolute event rates, *P* values; OR or RR; 95% CI)	Other relevant findings or adverse events	Limitations; Other comments; Conclusions
Kapel et al. Arrhythmogenic anatomical isthmuses identified by electroanatomical mapping are the substrate for ventricular tachycardia in repaired tetralogy of Fallot.<br>• Year published: 2017<br>• PMID: 28182233	Aim: To identify anatomic isthmuses related to VT in patients with repaired ToF<br>Endpoints: No inducible VT and/or conduction block across isthmus<br>Study type: Observational, 2 centers (Leiden, Bordeaux)<br>Size: 74	Inclusion criteria: Patients with repaired ToF undergoing ventricular stimulation due to SVT (13) or at least one risk factor: syncope; QRS duration ≥180 ms; NSVT on Holter; at least moderate LV or RV dysfunction; age at repair ≥5 years; or presence of transannular patch. Mean age 40 ± 16 years; age at repair median 5.9 years. Some 28 patients had inducible VT: 13 of 28 had clinical VT; duration sustained or nonsustained not stated.	A total of 28 patients underwent ablation: acute success was 68% (19 of 28). Note that 2 patients without inducible VT underwent ablation. Some 28 patients had inducible VT: 24 of 28 macroreentry circuits identified; 7 focal, 2 of 28 uncertain; 37 of 41 had induced VT related to AI. Low-voltage AI was present in 26 of 28 with inducible VT versus 5 of 46 without VT; 16 of 26 with successful VT ablation; 8 of 26 not successful, 2 not attempted. Mean follow-up 50 ± 22 months: recurrent VT in 18% (5 of 28). ICD was implanted in 24 patients.	EAM with Biosense. Characterized AI as per prior publication. Measured length, width, conduction velocity index (length/conduction time). AI in patients with inducible VT were longer, narrower, lower conduction velocity index.<br><br>Outcomes difficult to understand.	Acute success 68%; recurrent VT in 18%. Patients with conduction block across AI did not have recurrent VT, versus 5 of 10 without successful isthmus block. Most common isthmus: between VSD patch and PV; followed by TA to PV.

Study name or acronym; Author; Year published; PMID	Aim of study; Endpoints; Study type (RCT, observational — multicenter or single center; case series or other [if RCT include intervention and comparator]); Study size (N)	Patient population with inclusion and exclusion criteria	Results (absolute event rates, *P* values; OR or RR; 95% CI)	Other relevant findings or adverse events	Limitations; Other comments; Conclusions
**Surgery and Surgical Ablation**					
Deal et al. Electrophysiologic drug testing in symptomatic ventricular arrhythmias after repair of tetralogy of Fallot.<br>• Year published: 1987<br>• PMID: 3591695	Aim: Management of VT in symptomatic ToF patients with clinical VT<br>Endpoints: Recurrent VT<br>Study type: Retrospective single center<br>Size: 9	Inclusion criteria: ToF patients with symptomatic VT, undergoing electrophysiologic testing	Mean 3.3 drugs tested/patient. Follow-up mean 2.2 years. All patients with clinical SVT had inducible SVT, 60% of patients with frequent PVC's had inducible SVT. Four patients underwent surgery: no recurrent VT.	In patients with repaired ToF, ventricular stimulation reproduces clinical SVT; patients with RV hypertension did not respond to any medications; intraoperative mapping (1) and VT resection performed in 1 of 3; VT too fast to map in 2.	Surgery performed in 3 patients with RV hypertension. Surgery to improve hemodynamics eliminated VT: none had recurrent VT.
Oechslin et al. Reoperation in adults with repair of tetralogy of Fallot: indications and outcomes.<br>• Year published: 1999<br>• PMID: 10424997	Aim: Outcomes of reoperation in patients with ToF<br>Endpoints: Death, recurrent VT<br>Study type: Retrospective single center<br>Size: 20 of 60 patients with VT	Inclusion criteria: 60 consecutive patients with ToF undergoing reoperation 1975–1997	SVT 33%; 16 of 20 with MVT at EPS. Some 11 of 16 underwent intraoperative mapping and cryoablation.<br>No mapping: noninducible/not performed 4, multifocal 1.<br>Outcomes of cryoablation: recurrent VT 18% (2 of 11); no cryoablation: 11% recurrent VT (1 of 9); 1 ICD, 3 AADs.	Sites of VT: infundibular septum adjacent to VSD patch, or RV free wall near ventriculotomy.<br><br>There were 33% preop VT, 7% postop VT (4 patients), and 3 of 4 with preoperative VT, one *de novo*. Ten-year survival was 92%; no operative mortality, and no late SCD.	Intraoperative mapping and cryoablation reduced recurrence of clinical SVT; recurrent VT 18%.

Study name or acronym; Author; Year published; PMID	Aim of study; Endpoints; Study type (RCT, observational — multicenter or single center; case series or other [if RCT include intervention and comparator]); Study size (N)	Patient population with inclusion and exclusion criteria	Results (absolute event rates, *P* values; OR or RR; 95% CI)	Other relevant findings or adverse events	Limitations; Other comments; Conclusions
Harrison et al. Sustained ventricular tachycardia in adult patients late after repair of tetralogy of Fallot.<br>• Year published: 1997<br>• PMID: 9350941	Aim: To determine features associated with SVT late after repair of ToF<br>Endpoints: SVT<br>Study type: Retrospective single center<br>Size: 18	Inclusion criteria: 18 adults with repaired ToF and VT compared with 192 repaired ToF without VT, from 1990–1994<br><br>Exclusion criteria: Treatment for atrial arrhythmias or unknown arrhythmia	EP map-guided operation for VT in 10 of 14 patients with VT undergoing reoperation; 10 with inducible VT in surgery: cryoablation performed at junction of infundibular septum with either VSD patch, parietal band, or RV free wall.<br><br>Surgery: 10 PVR, 1 repair, RVOT reconstruction with aneurysmectomy.<br><br>Mean follow-up 4 years.	Recurrent VT in 2 of 10 patients with map- guided VT surgery; 2 of 3 without ablation: no recurrent VT after surgery, although 1 received quinidine.	Intraoperative mapping and VT ablation success 80% for ToF.
Therrien et al. Impact of pulmonary valve replacement on arrhythmia propensity late after repair of tetralogy of Fallot.<br>• Year published: 2001<br>• PMID: 11369690	Aim: Effects of PVR or RVOT reconstruction in ToF on risk of VA, SCD<br>Endpoints: Death, SVT or sustained atrial flutter, AF<br>Study type: Multicenter retrospective<br>Size: 70<br>Control group: 30	Inclusion criteria: Consecutive patients >18 years of age undergoing PVR or RVOT reconstruction late after ToF; mean age 28 years	Preoperative SMVT (18 of 70); 86% with hemodynamic problems: ≥ moderate PR, 92%, ≥ moderate RV dilatation 71%; 18 patients with VT: 17 had EPS, 14 underwent surgery, 10 had inducible VT at surgery.<br>Ventricular cryoablation: operative mortality 4%; late death 3%, (one SCD, one HF).<br>Preoperative clinical VT: 2 of 6 patients without cryoablation had recurrent MVT (*P*<.001) (12 clinical preoperative atrial fibrillation or atrial flutter: cryoablation 0 of 6 with recurrent AT; 4 of 6 no cryoablation with recurrent AT).	Cryoablation performed with intraoperative mapping in 10 patients. Recurrent VT in 20% (2 of 10), and in 1 of 3 patients without cryoablation.<br>The 10-year survival was 86%; preoperative VT 26%, postoperative 9%; VT correlated with hemodynamic abnormalities in 86%; surgery without cryoablation: recurrent VT in 33%.	Cryoablation with intraoperative mapping reduced VT during follow-up from 22% to 9%.<br><br>PVR stabilized QRS duration.

Study name or acronym; Author; Year published; PMID	Aim of study; Endpoints; Study type (RCT, observational — multicenter or single center; case series or other [if RCT include intervention and comparator]); Study size (N)	Patient population with inclusion and exclusion criteria	Results (absolute event rates, *P* values; OR or RR; 95% CI)	Other relevant findings or adverse events	Limitations; Other comments; Conclusions
Therrien et al. Optimal timing for pulmonary valve replacement in adults after tetralogy of Fallot repair.<br>• Year published: 2005<br>• PMID: 15757612	Aim: To assess changes in RV size and function with cardiac MRI after PV replacement in adults with repaired ToF<br>Endpoints: Changes in RV size and function by cardiac MRI after surgery<br>Study type: Single center retrospective<br>Size: 17	Inclusion criteria: Pre- and postoperative cardiac MRI in adult ToF patients undergoing PV replacement	At 21 months after PV replacement, a statistically significant decrease in RV volume occurred at a mean follow-up of 21 months postsurgery. RV systolic function did not change.	Patients with marked RV enlargement did not show normalization of RV volumes after PVR. Rhythm was not assessed.	With PVR, patients with RV end diastolic volume >170 mL/m^2 preoperatively did not normalize RV volumes postoperatively.
Mavroudis et al. Arrhythmia surgery in patients with and without congenital heart disease.<br>• Year published: 2008<br>• PMID: 18721574	Aim: Efficacy of arrhythmia surgery in CHD and young patients<br>Endpoints: Mortality, arrhythmia recurrence.<br>Study type: Retrospective single center<br>Size: 13 of 89 young patients with VT	Inclusion: 613 of 211 consecutive patients undergoing arrhythmia surgery 1987–2007; mean age 16 years<br><br>Exclusion: 111 previously reported Fontan patients	VT patients: 6 ToF, 2 TGA, 1 ASD/Uhl's anomaly, 2 VSD, 1 absent PV, 1 no SHD.<br><br>Preoperative and intraoperative mapping; resection and cryoablation performed.	Two of 13 patients had recurrent VT; 3 patients had late AT.	There was 85% success with map-guided cryoablation of VT.

Study name or acronym; Author; Year published; PMID	Aim of study; Endpoints; Study type (RCT, observational — multicenter or single center; case series or other [if RCT include intervention and comparator]); Study size (N)	Patient population with inclusion and exclusion criteria	Results (absolute event rates, *P* values; OR or RR; 95% CI)	Other relevant findings or adverse events	Limitations; Other comments; Conclusions
Harrild et al. Pulmonary valve replacement in tetralogy of Fallot: impact on survival and ventricular tachycardia.<br>• Year published: 2009<br>• PMID: 19139389	Aim: Effects of late PVR for ToF<br>Endpoints: Death, sustained VT<br>Study type: Retrospective single center<br>Size: 98<br>Controls: 77	Inclusion: Patients with ToF undergoing PVR for RV dilatation; median age 26.6 years<br><br>Exclusion: PVR <5 years prior to study; AVSD; Ebstein anomaly	PVR group: 19% QRS >180 ms; ≥moderate RV dysfunction 33%; LV 2%; ≥moderate RV dilatation 89%.<br><br>Operative cryoablation of RVOT in 6 patients, 1997–2003. Five patients experienced VT during follow-up.<br><br>Median follow-up 1.1 years (0.1–25 years)<br><br>There were 13 late events: 5.3 per 100 patient years, PVR group, 2.9 for controls. Freedom from death, VT, or both: 5 years 80%, 10 years 41%.	Late PVR for symptomatic PR or RV dilatation did not reduce the incidence of VT or death. Cryoablation of RVOT: 5 of 6 experienced recurrent VT. Death or VT: 4.8 events per 100 patient years.	Five of 6 patients undergoing empiric cryoablation for VT had recurrent VT.
Adamson et al. Does pulmonary valve replacement post repair of tetralogy of Fallot improve right ventricular function?<br>• Year published: 2009<br>• PMID: 19567499	Aim: Does PVR after ToF improve RV function?<br>Endpoints: Effect of PVR on RV function, symptoms<br>Study type: meta-analysis<br>Size: 1070	Inclusion: An OvidSP search for PVR and ToF revealed 730 relevant papers: 19 selected as representing best evidence	Mortality: RV volume reduction reported in most studies; exercise capacity improved in some.	ECG QRS duration decreased or did not increase as controls; decreased incidence of ventricular and atrial arrhythmias reported in one study using operative arrhythmia surgery (Therrien 2001). Rare sudden deaths reported postoperatively.	PVR after ToF repairs offers symptomatic benefit, reduction in RV volume, and usually improved RV function, often with reduction in QRS duration.

Study name or acronym; Author; Year published; PMID	Aim of study; Endpoints; Study type (RCT, observational — multicenter or single center; case series or other [if RCT include intervention and comparator]); Study size (N)	Patient population with inclusion and exclusion criteria	Results (absolute event rates, *P* values; OR or RR; 95% CI)	Other relevant findings or adverse events	Limitations; Other comments; Conclusions
Sabate Rotes et al. Ventricular arrhythmia risk stratification in patients with tetralogy of Fallot at the time of pulmonary valve replacement.<br>• Year published: 2015<br>• PMID: 25416756	Aim: Risk stratification in ToF prior to PVR<br>Endpoints: VT, CA, appropriate ICD shock, or SCD<br>Study type: Retrospective single center, Mayo<br>Size: 205	Inclusion criteria: Patients with ToF PVR 1988–2010; mean age 33 years<br><br>Exclusion criteria: Pulmonary atresia, absent PV, AVSD, prior replacement of conduit or PV	Preoperative: SVT, 8%; 19 syncope; 21 NSVT; 16 clinical VT; 21 inducible VT.<br><br>Holters available in 22% (46 of 205), NSVT 46% (21 of 46 patients). EPS performed in 19% (40 of 205); positive in 52% (21 of 40); 5 patients underwent catheter ablation (5 of 21).<br><br>Indications for cryoablation: syncope, history of NSVT or SVT or inducible VT not undergoing RF ablation, unexplained syncope or near syncope.<br><br>Cryoablation in 22 patients; operative mortality 1.5%.<br><br>ICD placement in 23 patients (VT, cardiac arrest, LVEF <35%, unexplained syncope); appropriate shocks 30%; inappropriate shocks 26%.<br><br>Outcome: 9.4% (19 of 202) survivors; 5 SCD, 7 ICD, 3 CA, 4 sustained VT.	Cryo: Empiric lesions: VSD to PV, ± ventriculotomy to PV and/or TV. Not map guided, no block testing.<br><br>Cryoablation: 22; 4 of 16 SVT; 3 of 19 syncope; 5 of 15 with ICD; 10 of 40 with inducible VT.<br><br>Outcome of recurrent VT in 1 of 22 with cryoablation + ICD shock; 18 of 183 without cryoablation.<br><br>Outcomes not analyzed regarding preoperative EPS or catheter ablation of VT.<br><br>Event-free survival: 10 years, 85%; 15 years, 82%.<br><br>Ten patients had “normal” ECG and no events.	Patients with clinical history of sustained VT or LVEF <50% at highest risk of recurrent VT. Cryoablation as performed was not proarrhythmic; could have been protective.

Study name or acronym; Author; Year published; PMID	Aim of study; Endpoints; Study type (RCT, observational — multicenter or single center; case series or other [if RCT include intervention and comparator]); Study size (N)	Patient population with inclusion and exclusion criteria	Results (absolute event rates, *P* values; OR or RR; 95% CI)	Other relevant findings or adverse events	Limitations; Other comments; Conclusions
Sandhu et al. Perioperative electrophysiology study in patients with tetralogy of Fallot undergoing pulmonary valve replacement will identify those at high risk of subsequent ventricular tachycardia.<br>• Year published: 2018<br>• PMID: 29330130	Aim: Perioperative EPS in patients with ToF undergoing PVR to stratify risk<br>Endpoints: Recurrent VT, ICD shock<br>Study type: 2 centers in Colorado, prospective cohort<br>Size: 70	Inclusion criteria: Patients with ToF undergoing PVR 2006–2017. Median age 37 years, 50% women. Preoperative NSVT 14%; (10 of 70), although data on only 32 patients.<br><br>Exclusion criteria: Age <18 years, no preoperative EPS or LVOTO.	Preoperative EPS 49%; inducible SVT (34 of 70): monomorphic 74% (25), polymorphic 26% (9).<br><br>Positive preoperative VT: Males, *P*=.02; greater WT, *P*=.03; greater BMI, *P*=.05.<br><br>Cryoablation in 31 of 34 patients; postoperative EPS inducible SVT 45% (14 of 31); postoperative EPS timing not stated. All received ICD's.<br><br>Follow-up 6 ± 3 years.<br><br>Negative preoperative VT: subsequent VT 3%.<br><br>Negative postoperative EPS (on amiodarone, not stated why): 6% subsequent VT.<br><br>ICDs implanted: 16 total, 14 early.	Linear cryoablation: anterior ventriculotomy scar or RVOT patch to PV (18); VSD patch to PV or circumferential RVOT lesion.<br><br>Not map guided, no conduction block testing.<br><br>Appropriate ICD shocks, 21% (3 of 14); inappropriate 7% (1 of 14).<br><br>Patients with negative preoperative EPS did not undergo postoperative EPS.	Approximately 50% with inducible SVT pre-PVR: 55% noninducible VT after cryoablation, at least one on amiodarone.
**Repaired CHD and ICD Therapy, Risk VT/VF**					

Study name or acronym; Author; Year published; PMID	Aim of study; Endpoints; Study type (RCT, observational — multicenter or single center; case series or other [if RCT include intervention and comparator]); Study size (N)	Patient population with inclusion and exclusion criteria	Results (absolute event rates, *P* values; OR or RR; 95% CI)	Other relevant findings or adverse events	Limitations; Other comments; Conclusions
Khairy et al. Implantable cardioverter-defibrillators in tetralogy of Fallot.<br>• Year published: 2008<br>• PMID: 18172030	Aim: Value of ICDs in repaired ToF<br>Study type: Observational multicenter cohort<br>Size: 121	A total of 121 patients (median age 33.3 years; 59.5% male) were enrolled from 11 sites and followed up for a median of 3.7 years.	ICD indications: primary prevention 56%, secondary 44%.<br><br>Follow-up median 3.7 years.<br><br>Appropriate shocks: 7.7% primary vs 9.8% secondary; *P*=.011.<br><br>Predictors of appropriate shocks: higher LVEDP (HR 1.3 per mm Hg), NSVT (HR 3.7).<br><br>ICD complications 30%.<br><br>Highest rate of appropriate ICD shocks in patients with LVEDP ≥12, NSVT, prior ventriculotomy, and QRS duration ≥180 ms.	Primary prevention indications included patients presenting with syncope.<br><br>Some 55 patients with primary prevention ICD had VT stimulation: no difference in appropriate shocks in patients with inducible or noninducible VT.	Appropriate ICD therapy received in 7.7%–9.8% of patients.<br><br>Approximately 18% of patients with inducible SVT had appropriate ICD rx.
Valente et al. Contemporary predictors of death and sustained ventricular tachycardia in patients with repaired tetralogy of Fallot enrolled in the INDICATOR cohort<br>• Year published: 2014<br>• PMID: 24179163	Aim: To identify risk factors for SCD, VT in repaired ToF.<br>Endpoints: Death, SVT<br>Study type: Multicenter cohort<br>Size: 873	Inclusion criteria: Patients with repaired ToF enrolled in INDICATOR registry, CMR 1997–2010	Death or SVT: 3.7%; 14% of deaths were sudden. Median age 42 years at death, 29 years with VT. Multivariable risk factors: RV mass/volume ratio ≥0.3 g/ml, LVEF <54 (females), 55% (males) (z core < -2.0), history of atrial tachycardia.<br><br>QRS duration ≥180 ms; HR 3.17; CI 1.42–7.10; *P*=.005.	If none of 3 predictors present, 10-year survival 98%; with all 3 risk factors, 10-year survival 43%. Subgroup analysis of 315 patients with RVOT gradient measured: higher pressure significant risk; HR 1.39 (1.19–1.62); *P*<.001.	Risk for sudden death and VT: elevated RV mass, elevated RV pressure, history of atrial tachycardia, decreased LVEF <55%.

Study name or acronym; Author; Year published; PMID	Aim of study; Endpoints; Study type (RCT, observational — multicenter or single center; case series or other [if RCT include intervention and comparator]); Study size (N)	Patient population with inclusion and exclusion criteria	Results (absolute event rates, *P* values; OR or RR; 95% CI)	Other relevant findings or adverse events	Limitations; Other comments; Conclusions
Kella et al. Lesion-specific differences for implantable cardioverter defibrillator therapies in adults with congenital heart disease.<br>• Year published: 2014<br>• PMID: 24889130	Aim: ICD therapy versus specific type of CHD<br>Endpoints: ICD usage<br>Study type: Single center observational<br>Size: 59	Inclusion criteria: ACHD patients with ICDs, 2000–2011; mean age 36 years<br><br>Exclusion criteria: Long QT, ARVC, noncompaction	ToF 56%, TGA 25%, other 19%; ICD indications: primary, 53%; mean ventricular EF 38%; median follow-up 3.2 years; appropriate rx: 20%, inappropriate 22%.<br><br>ToF: appropriate shocks 27.3% vs 11.5% other lesions, *P*=.043.	No difference in inappropriate shocks by lesion.	Non-ToF patients less likely to receive appropriate ICD rx.
Koyak et al. Sudden cardiac death in adult congenital heart disease: can the unpredictable be foreseen?<br>• Year published: 2017<br>• PMID: 27247006	Aim: Can ECG and Echo findings predict risk of SCD?<br>Study type: Retrospective, case-controlled multicenter, CONCOR, in Toronto and Leuven.<br>Size: 131	Inclusion criteria: ACHD patients with SCD due to an arrhythmia, with 2 case controls per patient<br><br>Exclusion criteria: Insufficient ECG or echo data; 40 patients	SCD age 36 ± 14 years<br><br>Congenital lesions: ccTGA 16%, ToF 15%, left-sided obstruction 13%, septal defects 11%, Fontan 5%.<br><br>Increased SCD risk: Increased QRS duration ≥5 ms per year (OR 1.9; CI 1.1–3.3; *P*=.013); change in function to severe ventricular dysfunction (OR 16.9; CI 1.8-120.1; *P*=.008).		Predictors of SCD in ACHD: severe ventricular dysfunction or increasing QRS duration.

Study name or acronym; Author; Year published; PMID	Aim of study; Endpoints; Study type (RCT, observational — multicenter or single center; case series or other [if RCT include intervention and comparator]); Study size (N)	Patient population with inclusion and exclusion criteria	Results (absolute event rates, *P* values; OR or RR; 95% CI)	Other relevant findings or adverse events	Limitations; Other comments; Conclusions
Teuwen et al. Non-sustained ventricular tachycardia in patients with congenital heart disease: An important sign?<br>• Year published: 2016<br>• PMID: 26805391	Aim: NSVT as risk factor for SVT in patients with ACHD.<br>Endpoints: SVT or VF, cardiac arrest<br>Study type: Retrospective multicenter<br>Size: 145	Inclusion criteria: Patients with ACHD presenting with SVT or NSVT	ToF 29%, TGA 13%, single ventricle 12%, aortic valve disease 12%, others.<br><br>NSVT 71%, SVT 17%, VF 12%.<br><br>Mean age 40 years, median follow-up 5 years.<br><br>ICD 36%: 29% appropriate shocks, 23% inappropriate.<br><br>Some 5% of patients presenting with NSVT developed SVT or VF over time; total 3% resuscitated from SCA.	SVT or VF rarely occurred in patients with prior NSVT.<br><br>Patients presenting with SVT or VF had recurrent VT or VF. One patient presenting with SVT died without an ICD.	Some 5% of patients with ACHD presenting with NSVT developed SVT or VF during follow-up.
Backhoff et al. Internal cardioverter defibrillator indications and therapies after atrial baffle procedure for d-transposition of the great arteries: a multicenter analysis.<br>• Year published: 2016<br>• PMID: 27503213	Aim: ICD use in patients with TGA<br>Endpoints: Appropriate ICD therapy<br>Study type: Multicenter observational<br>Size: 33	Inclusion criteria: Adults with prior atrial baffle repairs for d-TGA from 4 German centers	Primary prevention: 88%<br><br>Some 64% had atrial tachycardia.<br><br>Median follow-up 4.8 years.<br><br>No appropriate shocks in patients with secondary prevention.<br><br>Complications: 21		ICDs for primary prevention in patients with TGA: 10% appropriate shocks, 24% inappropriate shocks.

Study name or acronym; Author; Year published; PMID	Aim of study; Endpoints; Study type (RCT, observational — multicenter or single center; case series or other [if RCT include intervention and comparator]); Study size (N)	Patient population with inclusion and exclusion criteria	Results (absolute event rates, *P* values; OR or RR; 95% CI)	Other relevant findings or adverse events	Limitations; Other comments; Conclusions
Probst et al. Prevention of sudden cardiac death in patients with tetralogy of Fallot: risk assessment and long-term outcome.<br>• Year published: 2018<br>• PMID: 29980366	Aim: To assess risk factors for SCD in ToF<br>Endpoints: Death<br>Study type: Single-center retrospective<br>Size: 174	Inclusion criteria: Adults with repaired ToF with sustained VT or CA (N=17) and without VT (n=157)	Aim to assess value of risk stratification score for prediction of ICD use or SCD. Patients with ICD for secondary prevention had a risk score of 6.3 ± 2.2, and it was 5.8 ± 2.4 for patients with ICD for primary prevention; not significantly different.	For the majority of patients, risk score variables were incomplete.	Risk score utility severely limited by lack of data.

Inherited Arrhythmia Syndromes

Study name or acronym; Author; Year published; PMID	Aim of study; Endpoints; Study type (RCT, observational — multicenter or single center; case series or other [if RCT include intervention and comparator]); Study size (N)	Patient population with inclusion and exclusion criteria	Results (absolute event rates, *P* values; OR or RR; 95% CI)	Other relevant findings or adverse events	Limitations; Other comments; Conclusions
Berruezo et al. Safety, long-term outcomes and predictors of recurrence after first-line combined endoepicardial ventricular tachycardia substrate ablation in arrhythmogenic cardiomyopathy. Impact of arrhythmic substrate distribution pattern. A prospective multicentre study.<br>• Year published: 2017<br>• PMID: 28431051	Aim: Procedural safety, outcomes, and predictors of recurrence after first-line combined ENDO-EPI VT ablation<br>Endpoints: Acute success, complications, recurrence<br>Study type: Prospective, multicenter observational study<br>Size: 41	Inclusion criteria:<br>- Intervention: First-line epi-endo ablation (substrate > conventional)<br>- Meet AC criteria<br>- ≥1 episode of sustained VT<br><br>Exclusion criteria: NA	Acute success (noninducible) of 90%.<br><br>VT recurrence of 26.8%.	Cohort: ARVC (83%), LV AC (17%); prior ablation: 9.6%: ENDO (4.8%), ENDO-EPI (4.8%); preprocedural medications 76%: sotalol (51.2%), amiodarone (14.6%), BB (9.7%); postprocedure medications 51.2%: sotalol (85%).<br><br>ICD Present 100%: preablation (75.6%), postablation (24.4%).<br><br>Indications: VT Storm (19.5%), recurrent ICD transplant (39%), documented MMVT (41.4%).<br><br>Follow-up: 32.2 ± 21.8 months<br><br>Complications: 5% - Tamponade (2), Death (1)<br><br>RFCA approach: Combined epi-endo (substrate > conventional)<br><br>Predominant LV involvement only independent predictor of VT recurrence (HR 3.28 [1–10.78], *P*=.05).<br><br>LV AC (71.4%) vs ARVC (17.6%), *P*<.005	Localization of recurrent VT unknown.<br><br>Prior ablation<br><br>Due to study design, it is unknown whether endo-only would have been as effective.

Study name or acronym; Author; Year published; PMID	Aim of study; Endpoints; Study type (RCT, observational — multicenter or single center; case series or other [if RCT include intervention and comparator]); Study size (N)	Patient population with inclusion and exclusion criteria	Results (absolute event rates, *P* values; OR or RR; 95% CI)	Other relevant findings or adverse events	Limitations; Other comments; Conclusions
Müssigbrodt et al. Should all patients with arrhythmogenic right ventricular dysplasia/cardiomyopathy undergo epicardial catheter ablation?<br>• Year published: 2017<br>• PMID: 27900527	Aim: To compare long-term effectiveness between ENDO and EPI-ENDO RFCA<br>Endpoints: VT Recurrence<br>Study type: Retrospective, observational, single-center study<br>Size: 45<br>Intervention: RFCA (ENDO, ENDO > EPI)	Inclusion criteria:<br>- Intervention: RFCA (ENDO, ENDO > EPI)<br>- Meet ARVC 2010 Criteria<br>- MMVT by ECG or ICD interrogation despite either AADs and/or beta-blocker<br><br>Exclusion criteria: NA	Acute success (noninducible) of 84.4%<br><br>VT recurrence 42.2%<br><br>VT recurrence: ENDO 43.5% vs ENDO-EPI 41.9%, NS<br><br>VT-free survival 57.8% with no difference between ENDO vs EPI-ENDO, NS, log-rank *P*=.550	Cohort: ARVC (100%)<br>Ablation: ENDO (51.1%), ENDO-EPI (48.9%)<br>Mean number of ablations per patient: 2 (1–6)<br><br>Postablation medications total 84.4%: BB only (55.6%), I (6.7%), III (31.1%)<br><br>ICD Present: NA<br><br>Indications: NA<br><br>Follow-up: 31.1 ± 27.4 months<br><br>Complications 5.4 %: TIA(1), pericardial effusions (2), PE (2), death (1, due to PE).<br><br>RFCA approach: ENDO > EPI (if failed ENDO) (conv)	Multiple procedures

Study name or acronym; Author; Year published; PMID	Aim of study; Endpoints; Study type (RCT, observational — multicenter or single center; case series or other [if RCT include intervention and comparator]); Study size (N)	Patient population with inclusion and exclusion criteria	Results (absolute event rates, *P* values; OR or RR; 95% CI)	Other relevant findings or adverse events	Limitations; Other comments; Conclusions
Orgeron et al. Implantable cardioverter-defibrillator therapy in arrhythmogenic right ventricular dysplasia/cardiomyopathy: predictors of appropriate therapy, outcomes, and complications.<br>• Year published: 2017<br>• PMID: 28588093	Aim: Clinical characteristics and outcomes of ARVC/D with ICD<br>Endpoints: Appropriate therapy, long-term outcome, and complications<br>Study type: Observational registry<br>Size: 312	Inclusion criteria:<br>- Meet ARVC 2010 Criteria<br>- ICD<br><br>Exclusion criteria: NA	Younger age at presentation (HR: 3.14; 95% CI, 1.32–7.48; *P*=.010).<br><br>High premature ventricular contraction burden (HR: 4.43; 95% CI, 1.35–14.57; *P*<.014).<br><br>Independent predictors of ventricular fibrillation or flutter on multivariate analysis.<br><br>Univariate: Male, syncope, ≤30 years at presentation, high burden PVC	Cohort: ARVC<br><br>Therapy: Appropriate (60%), 19% for VF/VFL<br><br>Complications: 21%<br><br>Inappropriate: 21%<br><br>Follow-up: 8.8 ± 7.33 years<br><br>Mortality: 4%<br><br>Inducibility at electrophysiology study (HR: 2.28; 95% CI, 1.10– 4.70; *P*=.025) only predictor after multivariable analysis of any appropriate ICD therapy.	

Study name or acronym; Author; Year published; PMID	Aim of study; Endpoints; Study type (RCT, observational — multicenter or single center; case series or other [if RCT include intervention and comparator]); Study size (N)	Patient population with inclusion and exclusion criteria	Results (absolute event rates, *P* values; OR or RR; 95% CI)	Other relevant findings or adverse events	Limitations; Other comments; Conclusions
Jiang et al. Catheter ablation for ventricular tachycardia in patients with arrhythmogenic right ventricular dysplasia/cardiomyopathy: a systematic review and meta-analysis.<br>• Year published: 2016<br>• PMID: 27920450	Aim: Acute and long-term efficacy of RFCA of VT — Comparing ENDO vs EPI/ENDO<br>Endpoints: Acute success, recurrence<br>Study type: Meta-analysis<br>Size: 181	Inclusion criteria:<br>- Published in Pubmed, EMBASE, Cochrane<br>- ARVC undergoing RFCA for VT<br>- Intervention: RFCA(ENDO, ENDO-EPI)<br>- Sample Size ≥10<br>- 3D EAM (Carto)<br>- Acute and long-term efficacy of VT ablation<br><br>Exclusion criteria: NA	Pooled estimated acute efficacy: Total 95% (95% CI 87%–100%), ENDO: 96% (95% CI 87%–100%), EPI-ENDO: 98% (95% CI 91%–100%).<br><br>Pooled estimated long-term efficacy: Total 71% (95% CI 61%–80%), ENDO: (63%, 95% CI 51%–74%), EPI-ENDO: (82%, 95% CI 72%–90%).	Cohort: 8 studies<br><br>Mean number of ablations: NA<br><br>Prior ablation: 26% noted in 3 studies<br><br>Preprocedure AADs 91.1%; postprocedure AADs 31% to 100% (median 64%–78%)<br><br>ICD present preablation: 84.0% in 5 studies<br><br>Follow-up: median 20 months (mean ranged from 13 to 41 months)<br><br>Complications 3.7 %: tamponade (1), pericarditis (1), RV perforation (2), PE (2), 1 death due to PE	In most cohort studies, ICD not used 100%; thus, asymptomatic VT unknown, center variations.

Study name or acronym; Author; Year published; PMID	Aim of study; Endpoints; Study type (RCT, observational — multicenter or single center; case series or other [if RCT include intervention and comparator]); Study size (N)	Patient population with inclusion and exclusion criteria	Results (absolute event rates, *P* values; OR or RR; 95% CI)	Other relevant findings or adverse events	Limitations; Other comments; Conclusions
Philips et al. Outcomes and ventricular tachycardia recurrence characteristics after epicardial ablation of ventricular tachycardia in arrhythmogenic right ventricular dysplasia/cardiomyopathy.<br>• Year published: 2015<br>• PMID: 25530221	Aim: Procedural strategy, safety, and efficacy of epicardial RFCA for recurrent VT<br>Endpoints: Acute success, recurrence, complications<br>Study type: Prospective, single-center observational study<br>Size: 62	Inclusion criteria:<br>- Intervention: RFCA (EPI only, ENDO-EPI)<br>- Meet ARVC 2010 Criteria<br>- Recurrent VT<br>- ≥1-year follow-up after last procedure<br><br>Exclusion criteria: NA	Acute success (noninducible) 97%<br><br>Recurrence 27%	Cohort: ARVC (100%)<br><br>Mean number of ablations: 1.9 (1–3)<br><br>Prior ablation: ENDO (50%)<br><br>Last ablation: 60% (EPI only), 40% (EPI-ENDO)<br><br>Preprocedure medications: total (80%): sotalol (57%), amiodarone (50%), mexiletine (27%); postprocedure meds: total (33%): sotalol (23%), flecainide (10%).<br><br>ICD present: 100%<br><br>Follow-up: 19.7 ± 11.7 months<br><br>Major complications: 0%, pericarditis (1)	Prior ENDO ablation in 50%

Study name or acronym; Author; Year published; PMID	Aim of study; Endpoints; Study type (RCT, observational — multicenter or single center; case series or other [if RCT include intervention and comparator]); Study size (N)	Patient population with inclusion and exclusion criteria	Results (absolute event rates, *P* values; OR or RR; 95% CI)	Other relevant findings or adverse events	Limitations; Other comments; Conclusions
Santangeli et al. Long-term outcome with catheter ablation of ventricular tachycardia in patients with arrhythmogenic right ventricular cardiomyopathy.<br>• Year published: 2015<br>• PMID: 26546346	Aim: Long-term outcomes after ENDO and adjunctive EPI VT RFCA<br>Endpoints: Acute success, recurrence<br>Study type: Observational, single-center prospective study<br>Size: 62	Inclusion criteria:<br>- Intervention: RFCA (ENDO, ENDO > EPI)<br>- Meet ARVC 2010 criteria<br>- Recurrent VT<br>- ≥ 1-year follow-up after last procedure<br><br>Exclusion criteria: NA	Acute success at last procedure (noninducible), 77%<br><br>Recurrence 29%	Cohort: ARVC (100%)<br><br>Median number of ablations: 2 (1–5)<br><br>Ablation: ENDO (37%) ENDO > EPI (63%). Single procedure (45%).<br><br>Preprocedure medications: none or BB (13%), Class I or sotalol (40%), amiodarone (47%); postprocedure medications: none or BB (63%), Class I or sotalol (34%), amiodarone (3%).<br><br>ICD present: 58%<br><br>Follow-up after last ablation: 56 ± 44 months<br><br>Major complications: 4% (n=5), DVT/PE (2), pericardial effusion (1), RV puncture (1), pericarditis (1).<br><br>Intervention: RFCA (ENDO, ENDO > EPI) Conv+Substrate	Staged EP (ENDO then EPI)

Study name or acronym; Author; Year published; PMID	Aim of study; Endpoints; Study type (RCT, observational — multicenter or single center; case series or other [if RCT include intervention and comparator]); Study size (N)	Patient population with inclusion and exclusion criteria	Results (absolute event rates, *P* values; OR or RR; 95% CI)	Other relevant findings or adverse events	Limitations; Other comments; Conclusions
Link et al. Ventricular arrhythmias in the North American multidisciplinary study of ARVC: predictors, characteristics, and treatment.<br>• Year published: 2014<br>• PMID: 25011714	Aim: Predictors, characteristics, and treatment of VAs in ARVC<br>Endpoints: ICD-treated VA, death, or transplantation. Potentially life-threatening arrhythmia (VT >240 bpm, VF)<br>Study type: Observational multicenter registry<br>Size: 137	Inclusion criteria: Meets ARVC 2010 criteria<br><br>Exclusion criteria: NA	ICD-treated VA, death, transplant: 48 (37%)<br><br>Potential life-threatening arrhythmia (VT >240, VF): 22 (17%)<br><br>Predictor of LAE: Younger age at enrollment<br><br>ATP successful in terminating 92% of VT<br><br>VT episodes: 502 (489 MMVT and 13 PMVT)	ICD 108 (79%); AAD use in ICD: 55%.<br><br>Syncope/SVT/CA (61%) in ICD group.<br><br>Fast VT/VF 20% in ICD group.<br><br>Follow-up 3.3 ± 1.7 years in ICD group.	

Study name or acronym; Author; Year published; PMID	Aim of study; Endpoints; Study type (RCT, observational — multicenter or single center; case series or other [if RCT include intervention and comparator]); Study size (N)	Patient population with inclusion and exclusion criteria	Results (absolute event rates, *P* values; OR or RR; 95% CI)	Other relevant findings or adverse events	Limitations; Other comments; Conclusions
Berruezo et al. Combined endocardial and epicardial catheter ablation in arrhythmogenic right ventricular dysplasia incorporating scar dechanneling technique.<br>• Year published: 2012<br>• PMID: 22205683	Aim: To determine EPI vs ENDO scar area in ARVC and efficacy of combined EPI-ENDO VT RFCA with conducting channel elimination<br>Endpoints: Area of scar, success, recurrence<br>Study type: Observational, single-center prospective<br>Size: 11	Inclusion criteria:<br>- Intervention: RFCA (ENDO-EPI)<br>- Meet ARVC 2010 Criteria<br>- Clinical VT<br><br>Exclusion criteria: NA	Scar Area: ENDO: 26 ± 18 cm^2; EPI: 94 ± 45 cm^2; *P*<.01.<br><br>Acute: Success (noninducible) 100%<br><br>Recurrence: 9%	Cohort: ARVC (100%)<br><br>Ablation: ENDO (14%), ENDO > EPI (86%)<br><br>Preprocedure medications: sotalol (64%), postprocedure medications: sotalol (64%)<br><br>ICD present: 100% (preablation 55%, postablation 45%)<br><br>Indications: SMVT (55%), storm (45%)<br><br>Follow-up after last ablation: 11 months<br><br>Major complications: 9%, RV puncture (1)<br><br>Intervention: RFCA (combined EPI-ENDO) substrate	EPI-ENDO first line

**Study name or acronym; Author; Year published; PMID**	**Aim of study; Endpoints; Study type (RCT, observational — multicenter or single center; case series or other [if RCT include intervention and comparator]); Study size (N)**	**Patient population with inclusion and exclusion criteria**	**Results (absolute event rates, *P* values; OR or RR; 95% CI)**	**Other relevant findings or adverse events**	**Limitations; Other comments; Conclusions**
Philips et al. Outcomes of catheter ablation of ventricular tachycardia in arrhythmogenic right ventricular dysplasia/cardiomyopathy.<br>• Year published: 2012<br>• PMID: 22492430	Aim: PVC burden association with VT; isuprel effect on VT induction<br>Endpoints: VT<br>Study type: Observational single-center prospective<br>Size: 16	Inclusion criteria:<br>- Intervention: RFCA (ENDO, ENDO > EPI)<br>- Meet ARVC 2010 criteria<br>- clinical VT<br>- RFCA<br><br>Exclusion criteria: NA	PVC burden associated with VT: median PVC 7275 per 24 hours<br><br>Isuprel-induced VT: 16 of 27 (59%)<br><br>Acute success 95%<br><br>Recurrence: 25%	Cohort: ARVC (100%)<br><br>Prior ablation: 25% (All ENDO)<br>Ablation: ENDO (50%), EPI (50%)<br><br>Preprocedure medications: BB (60%), AADs (33%); postprocedure medications: BB (60%), AADs (13%)<br><br>ICD present: 100% (implanted at RFCA)<br><br>Follow-up after last ablation: 15 months (7–30 months)<br><br>Major complications: 0%	Primary aim no success or recurrence

Study name or acronym; Author; Year published; PMID	Aim of study; Endpoints; Study type (RCT, observational — multicenter or single center; case series or other [if RCT include intervention and comparator]); Study size (N)	Patient population with inclusion and exclusion criteria	Results (absolute event rates, *P* values; OR or RR; 95% CI)	Other relevant findings or adverse events	Limitations; Other comments; Conclusions
Bai et al. Ablation of ventricular arrhythmias in arrhythmogenic right ventricular dysplasia/cardiomyopathy: arrhythmia-free survival after endo-epicardial substrate-based mapping and ablation.<br>• Year published: 2011<br>• PMID: 21665983	Aim: Long-term freedom from VA: comparing ENDO vs EPI/ENDO RFCA<br>Endpoints: VT recurrence<br>Study type: Observational single-center prospective<br>Comparator: Ablation approach (ENDO vs EPI/ENDO)<br>Size: 49	Inclusion criteria:<br>- Meet ARVC 2010 Criteria<br>- Clinical symptomatic sustained VT<br>- Intervention: RFCA<br><br>Exclusion criteria: NA	Acute success (noninducible MVT): 100%<br><br>Acute success (noninducible MVT, PVT/VF): 94%<br><br>Recurrence: ENDO: 47.8% vs EPI/ENDO: 15.4%<br><br>Freedom from VA or appropriate ICD therapy was 52.2% (12 of 23) in ENDO and 84.6% (22 of 26) in EPI-ENDO, respectively; log-rank *P*=.029	Cohort: ARVC (100%)<br><br>Prior ablation: 54% in EPI/ENDO had prior ENDO at other center<br><br>Preprocedure medications: sotalol (49%), amiodarone (71%), dofetilide (3%), BB (18%), Class I or other (27%); postprocedure medications: off medications ENDO 21.7%, off medications EPI 69.2%<br><br>ICD present: 100%<br><br>Follow-up after last ablation: 1224 ± 310 days for ENDO and 1175 ± 112 days for EPI/ENDO<br><br>Major complications: 0%<br><br>PVC associated with VA recurrence. Frequent PVCs after ablation vs no PVCs had higher VA recurrence/ICD therapy: 3 of 33 (9%) vs 12 of 16 (75%); log-rank *P*<.001	Some 53% of the endo-EPI group had previous ENDO ablation. AAD use after ablation varied.

Study name or acronym; Author; Year published; PMID	Aim of study; Endpoints; Study type (RCT, observational — multicenter or single center; case series or other [if RCT include intervention and comparator]); Study size (N)	Patient population with inclusion and exclusion criteria	Results (absolute event rates, *P* values; OR or RR; 95% CI)	Other relevant findings or adverse events	Limitations; Other comments; Conclusions
Della Bella et al. Epicardial ablation for ventricular tachycardia: a European multicenter study.<br>• Year published: 2011<br>• PMID: 21841191	Aim: Descriptive analysis of epicardial VT RFCA endpoints: acute success, complications, recurrence<br>Study type: Observational multicenter<br>Size: 218 (13 ARVC)	Inclusion criteria:<br>- Epicardial VT RFCA<br>- Intervention: RFCA (EPI)<br><br>Exclusion criteria: NA	Acute success (noninducible): 69.2%<br><br>Recurrence: 30.8% (n=4)<br><br>Of note, 3 of 4 had recurrence of VT with similar morphology.	Cohort: ARVC (6%)<br><br>Ablation: 15.4% in ENDO-EPI (first line), 84.6% (EPI after previous failed ENDO); ARVC-only data<br><br>Medications: NA<br><br>ICD Present: 64% (total cohort); ARVC: NA<br><br>Follow-up after last ablation: 117.3 ± 18.2 months (total cohort)<br><br>Major complications 4.1% (n=9 total cohort): tamponade (8), abdominal hemorrhage (1); none in ARVC patients	Heterogeneous and multicenter nonuniform ablation approach.
Pokushalov et al. Percutaneous epicardial ablation of ventricular tachycardia after failure of endocardial approach in the pediatric population with arrhythmogenic right ventricular dysplasia.<br>• Year published: 2010<br>• PMID: 20601157	Aim: Feasibility and outcomes of EPI VT RFCA in pediatrics.<br>Endpoints: Acute success and recurrence<br>Study type: Observational single-center retrospective<br>Size: 17	Inclusion criteria:<br>- Intervention: EPI VT RFCA after failed ENDO ablation<br>- Recurrent VT despite optimal medical management<br><br>Exclusion criteria: NA	Acute success (noninducible): 94%<br><br>Recurrence: 29.4%<br><br>Of note, 3 repeat procedures, 1 medication, 1 ICD.	Cohort: ARVC (100%)<br><br>Mean age at ablation: 14 ± 4 years<br>Prior ablation: 35% (all ENDO)<br>Ablation: EPI/ENDO (100%)<br><br>Indications: LV dysfunction (2), syncope (4), recurrent monomorphic SVT (11)<br>Preablation medications: 100% (n=2.2 ± 0.9)<br>Postablation medications: 6%<br>ICD Present: 29%<br><br>Follow-up after last ablation: 26 ± 15 months	

Study name or acronym; Author; Year published; PMID	Aim of study; Endpoints; Study type (RCT, observational — multicenter or single center; case series or other [if RCT include intervention and comparator]); Study size (N)	Patient population with inclusion and exclusion criteria	Results (absolute event rates, *P* values; OR or RR; 95% CI)	Other relevant findings or adverse events	Limitations; Other comments; Conclusions
Sacher et al. Epicardial ventricular tachycardia ablation: a multicenter safety study.<br>• Year published: 2010<br>• PMID: 20488308	Aim: Safety and midterm complications in EPI VT RFCA<br>Endpoints: Complications<br>Study type: Observational retrospective multicenter<br>Size: 134 (13 ARVC)	Inclusion criteria: Intervention: EPI VT RFCA<br><br>Exclusion criteria: NA	Epicardial procedural complications: 8 (5%) major complications (7 epicardial bleeding and 1 coronary stenosis).<br><br>Epicardial midterm complication: 3 (1 pericarditis, 1 delayed tamponade, and 1 coronary occlusion noted 2 weeks after the procedure).<br><br>Complications in ARVC: 1 epicardial complication out of 13 patients with ARVC (coronary occlusion with AMI noted 2 weeks after procedure).<br><br>Recurrence: 29%	Cohort: ARVC (10%)<br><br>Prior ENDO ablation (64%)<br><br>Ablation: EPI (100%)<br><br>Medications: NA<br><br>ICD Present: NA<br><br>Follow-up after last ablation: 23 ± 21 months (total cohort)<br><br>Major complications for EPI approach: 5% acute, majority RV puncture<br><br>Major complications for total cohort: 11% (EPI and ENDO)<br><br>Minor complications: 17% RV puncture without consequence	

Study name or acronym; Author; Year published; PMID	Aim of study; Endpoints; Study type (RCT, observational — multicenter or single center; case series or other [if RCT include intervention and comparator]); Study size (N)	Patient population with inclusion and exclusion criteria	Results (absolute event rates, *P* values; OR or RR; 95% CI)	Other relevant findings or adverse events	Limitations; Other comments; Conclusions
Corrado et al. Prophylactic implantable defibrillator in patients with arrhythmogenic right ventricular cardiomyopathy/dysplasia and no prior ventricular fibrillation or sustained ventricular tachycardia.<br>• Year published: 2010<br>• PMID: 20823389	Aim: Incidence, efficacy, and safety of ICD in ARVC/D without prior VF or sustained VT<br>Endpoints: ICD therapy for VAs<br>Study type: Observational multicenter registry<br>Size: 106	Inclusion criteria:<br>- Meet ARVC criteria<br>- ICD with 1 or more arrhythmogenic risk factors (syncope, NSVT, family history of sudden death, ventricular stimulation)<br><br>Exclusion criteria: NA	Appropriate ICD Therapy: 24%<br><br>Appropriate ICD therapy for VF or life-threatening VFL: 16%<br><br>Inappropriate ICD Therapy: 19%<br><br>ICD Related Complications: 17%	AAD use in ICD: 55%<br><br>Syncope predicted any appropriate ICD interventions (HR, 2.94; 95% CI, 1.83–4.67; *P*=.013); syncope predicted shocks for VF/ or ventricular flutter (HR, 3.16; 95% CI, 1.39–5.63; *P*=.005).<br><br>No asymptomatic patient (27) with isolated familial sudden death had appropriate ICD therapy.<br><br>Follow-up: 58 ± 35 months in ICD group.	

Study name or acronym; Author; Year published; PMID	Aim of study; Endpoints; Study type (RCT, observational — multicenter or single center; case series or other [if RCT include intervention and comparator]); Study size (N)	Patient population with inclusion and exclusion criteria	Results (absolute event rates, *P* values; OR or RR; 95% CI)	Other relevant findings or adverse events	Limitations; Other comments; Conclusions
Garcia et al. Epicardial substrate and outcome with epicardial ablation of ventricular tachycardia in arrhythmogenic right ventricular cardiomyopathy/dysplasia.<br>• Year published: 2009<br>• PMID: 19620503	Aim: To characterize electroanatomic substrate and outcome of EPI/ENDO RFCA after failed ENDO RFCA<br>Endpoints: EA scar area, acute success<br>Study type: Observational single-center retrospective<br>Size: 13	Inclusion criteria:<br>- Meets ARVC task force criteria<br>- Intervention: RFCA ENDO/EPI VT RFCA after failed ENDO ablation<br>- Recurrent drug refractory VTs<br><br>Exclusion criteria: NA	EA Scar: EPI 95 ± 47 cm^2 vs ENDO 38 ± 32 cm^2; $P<.001$.<br><br>Acute success (noninducible): 85%<br><br>Recurrence: 23%	Cohort: ARVC (100%)<br><br>Prior ablation: 100% (all ENDO)<br>Mean Prior Procedure: 2 (1–4) ENDO ablation<br>Failed ≥1 AAD (100%)<br>Ablation: EPI/ENDO (100%)<br><br>Indications: recurrent VTs (symptomatic tolerated VT, ICD shocks, or both)<br><br>Preablation medications 100%: sotalol (100%), propafenone (5), quinidine (1), lidocaine (2), mexiletine (4), flecainide (2), procainamide (1); postablation medications 25%: sotalol (1), sotalol or mexiletine (1), flecainide (1).<br><br>Postablation BB: Yes (9), No (3), NA (1)<br><br>ICD Present: 92%<br><br>Follow-up after last ablation: 18.3 ± 12.7 months<br><br>Complications: 23% pericardial effusion, 6% (n=1) had tamponade.	EPI after ENDO, cannot assess EPI only.<br><br>ICD not present in 1 patient, so recurrence might be underestimated.

Study name or acronym; Author; Year published; PMID	Aim of study; Endpoints; Study type (RCT, observational — multicenter or single center; case series or other [if RCT include intervention and comparator]); Study size (N)	Patient population with inclusion and exclusion criteria	Results (absolute event rates, *P* values; OR or RR; 95% CI)	Other relevant findings or adverse events	Limitations; Other comments; Conclusions
Nogami et al. Changes in the isolated delayed component as an endpoint of catheter ablation in arrhythmogenic right ventricular cardiomyopathy: predictor for long-term success.<br>• Year published: 2008<br>• PMID: 18284499	Aim: To assess use of change in the isolated delayed component as an endpoint of the RFCA<br>Endpoints: VT Recurrence<br>Study type: Observational retrospective single-center<br>Size: 18	Inclusion criteria:<br>- Intervention: RFCA (ENDO)<br>- Meets ARVC criteria<br>- Sustained monomorphic VT<br><br>Exclusion criteria: NA	Acute success (noninducible clinical VT): 72%<br>Acute success (noninducible nonclinical VT): 66%<br>Acute success (noninducible): 44%<br><br>Recurrence: 33%	Cohort: ARVC (100%)<br><br>Indications: NA<br>Preablation medications 67%: amiodarone (6), none (6), procainamide (2), sotalol (1), disopyramide (3), mexiletine (2), flecainide (1) BB (1); postablation medications 72%: amiodarone (5), none (6), disopyramide (2), cibenzoline (1), amiodarone/BB/mexiletine (1), pirmenol (1).<br><br>ICD present: 33 (before: 11%, after: 22%)<br><br>Follow-up after last ablation: 61 ± 38 months<br><br>Complications: 0%<br><br>Change in isolated delayed component: Lower VT recurrence than no isolated delayed component or unchanged (*P*<.02)	At follow-up, no ICD in majority.

Study name or acronym; Author; Year published; PMID	Aim of study; Endpoints; Study type (RCT, observational — multicenter or single center; case series or other [if RCT include intervention and comparator]); Study size (N)	Patient population with inclusion and exclusion criteria	Results (absolute event rates, *P* values; OR or RR; 95% CI)	Other relevant findings or adverse events	Limitations; Other comments; Conclusions
Dalal et al. Long-term efficacy of catheter ablation of ventricular tachycardia in patients with arrhythmogenic right ventricular dysplasia/cardiomyopathy.<br>• Year published: 2007<br>• PMID: 17662396	Aim: Long-term RFCA efficacy<br>Endpoints: Recurrence<br>Study type: Observational retrospective multicenter<br>Size: 24	Inclusion criteria:<br>- Intervention: RFCA<br>- Meets ARVC task force criteria<br>- ≥1 attempt at RFCA<br>- ≥1 episodes of sustained VT<br><br>Exclusion criteria: NA	Acute success (noninducible clinical VT): 77%<br>Acute success (noninducible clinical VT): 54% of patients<br><br>Of note, 46% of all induced VTs ablated<br><br>Recurrence: 85% of procedures<br><br>Incidence of VT recurrence: 0.88 (95% CI 0.39–1.19) episodes per person-year<br><br>VT recurrence-free survival after a single RFCA: 75% at 1.5 months, 50% at 5 months, and 25% at 14 months of follow-up	Cohort: ARVC (100%)<br><br>Ablation: ENDO (100%)<br>Mapping: 3D EAM (21%)<br><br>Sex: 46% (Male)<br><br>Indications: NA<br>Preablation medications at ablation: BB (32%), sotalol (23%), amiodarone (19%), flecainide (10%), calcium channel (6%), mexiletine (4%), procainamide (2%), dofetilide (2%); postablation medications at recurrence: BB (32%), sotalol (23%), amiodarone (19%), flecainide (10%), calcium channel (6%), mexiletine (4%) procainamide (2%), dofetilide (2%)<br><br>ICD present in 79% (prior to ablation 21%, after 58%)<br><br>Follow-up after last ablation: 32 ± 36 months<br><br>Complications: 1 death, hemodynamically unstable VT, emergent thoracotomy for suspected tamponade	Not all VTs approached at initial procedure. EP procedure performed at 29 different centers in the US

Study name or acronym; Author; Year published; PMID	Aim of study; Endpoints; Study type (RCT, observational — multicenter or single center; case series or other [if RCT include intervention and comparator]); Study size (N)	Patient population with inclusion and exclusion criteria	Results (absolute event rates, *P* values; OR or RR; 95% CI)	Other relevant findings or adverse events	Limitations; Other comments; Conclusions
Verma et al. Short- and long-term success of substrate-based mapping and ablation of ventricular tachycardia in arrhythmogenic right ventricular dysplasia.<br>• Year published: 2005<br>• PMID: 15956125	Aim: Efficacy of substrate-based mapping and RFCA.<br>Endpoints: Acute success, recurrence<br>Study type: Observational single-center retrospective<br>Size: 22	Inclusion criteria:<br>- Intervention: RFCA (ENDO)<br>- Meet ARVC Criteria<br>- Drug refractory, recurrent VT<br><br>Exclusion criteria: NA	Acute success (noninducible): 82%<br><br>Recurrence: 36% (all received ICD therapies)<br><br>Complications: 0%	Cohort: ARVC (100%)<br><br>Prefailed medications: sotalol (45%), flecainide (36%), amiodarone (27%), procainamide (27%), quinidine (14%), propafenone (14%); preablation medications 100%: sotalol (50%), flecainide (18%), amiodarone per oral or intravenous (50%), procainamide (9%), lidocaine intravenous (14%), mexiletine (14%), BB (54%), calcium channel (5%); postablation medications 100%: same as before ablation.<br><br>Indication: frequent ICD therapies (n=15, 68%), sustained VT with syncope (6, 27%), sustained Vt without syncope (1, 5%).<br><br>ICD Present: 100% (at time of ablation: 82%, after 18%).<br><br>Follow-up after last ablation: median 37 (25–44) months<br><br>Major complications 4.5%: tamponade (1)<br><br>Intervention: RFCA (ENDO) substrate	No block confirmation. No entrainment mapping.

Study name or acronym; Author; Year published; PMID	Aim of study; Endpoints; Study type (RCT, observational — multicenter or single center; case series or other [if RCT include intervention and comparator]); Study size (N)	Patient population with inclusion and exclusion criteria	Results (absolute event rates, *P* values; OR or RR; 95% CI)	Other relevant findings or adverse events	Limitations; Other comments; Conclusions
Marchlinski et al. Electroanatomic substrate and outcome of catheter ablative therapy for ventricular tachycardia in setting of right ventricular cardiomyopathy.<br>• Year published: 2004<br>• PMID: 15477406	Aim: Clinical and electroanatomic characteristics of AC and RFCA outcomes<br>Endpoints: Success, recurrence<br>Study type: Observational, single-center, prospective<br>Size: 19	Inclusion criteria:<br>- Consecutive patients<br>- RV enlargement by echo<br>- Detailed EAMs with an area of discrete contiguous bipolar electrogram voltage abnormalities (amplitude, 1.5 mV).<br>- ≥2 morphologies of LBBB VT<br>- Intervention: RFCA<br><br>Exclusion criteria: NA	Acute success at last procedure (noninducible): 74%<br><br>Recurrence: 16%	Cohort: RV cardiomyopathy (89%), LVCM (11%) excluded in outcomes<br><br>Ablation: ENDO (100%)<br><br>Preprocedure medications: NA<br>Postprocedure medications: NA<br>ICD Present: 100% (prior: 67%, after: 33%)<br><br>Follow-up after last ablation: 27 ± 22 months<br><br>Complications: 0 %	
Corrado et al. Implantable cardioverter-defibrillator therapy for prevention of sudden death in patients with arrhythmogenic right ventricular cardiomyopathy/dysplasia.<br>• Year published: 2003<br>• PMID: 14638546	Aim: Efficacy of ICD therapy for prevention of SCD<br>Endpoints: First ICD therapy for VF or ventricular flutter<br>Study type: Observational retrospective multicenter<br>Size: 132	Inclusion criteria:<br>- Meet ARVC criteria<br>- ICD<br>- ≥6 months follow-up<br><br>Exclusion criteria: NA	Appropriate ICD therapy: 48% (50% VT, 50% VF or ventricular flutter)<br>Inappropriate ICD therapy: 16%<br>Device-related complications: 14%	ICD: 100%<br><br>Indications: cardiac arrest (10%), VT with hemodynamic compromise (39%), VT without hemodynamic compromise (23%), unexplained syncope (16%), other (12%).<br>EPS: 84%<br>Inducible VA: 88% (VT 68%, VF 32%)<br>AAD prior to ICD: 83%<br><br>83% on AAD at time of first ICD transplant<br><br>ICD for VT without hemodynamic compromise had lower incidence of VF or ventricular flutter (log rank *P*=.01).	

Study name or acronym; Author; Year published; PMID	Aim of study; Endpoints; Study type (RCT, observational — multicenter or single center; case series or other [if RCT include intervention and comparator]); Study size (N)	Patient population with inclusion and exclusion criteria	Results (absolute event rates, *P* values; OR or RR; 95% CI)	Other relevant findings or adverse events	Limitations; Other comments; Conclusions
Pappone et al. Electrical substrate elimination in 135 consecutive patients with Brugada syndrome.<br>• Year published: 2017<br>• PMID: 28500178	Aim: Substrate-based mapping and RFCA to normalize ECG and eliminate arrhythmogenic substrate in BrS<br>Endpoints: ECG normalization and noninducibility by elimination of arrhythmogenic EP substrate<br>Study type: Observational two-center prospective<br>Size: 135	Inclusion criteria:<br>- Consecutive<br>- Symptomatic Type I BrS-ECG with spontaneous or ajmaline<br>- ICD<br><br>Exclusion criteria: NA	ECG normalization before and after ajmaline with no VAs and noninducible: 98.5%<br><br>ECG normalization but with drug-induced BrS: 1.5% (n=2)<br><br>One VF with appropriate ICD shock, repeat procedure, and ablation; one NSVT, repeat procedure, and ablation	Baseline: 47% BrS-related symptoms (documented VT/VF, syncope, cardiac arrest) (Group 1) vs 53% VT/VF inducibility with symptoms but no documented arrhythmia (Group 2)<br>EPS: 82% inducible VF, 18% inducible PMVT<br>EPI/ENDO 3D EAM mapping: local activation, potential duration, voltage mapping. EPI ablation in RVOT in sinus; elimination of all abnormal potentials before and after ajmaline.<br>EAM: EPI Activation (anterior RVOT and RV anterior FW)<br>ICD therapies pre-RFCA: Group 1 (49%) vs Group 2 (1.4%); ICD therapies post-RFCA: Group 1 (5%) vs Group 2 (0%).<br>ECG normalized: 42% sinus RFCA with negative ajmaline, 58% sinus RFCA with positive ajmaline additional RFCA<br>EPS post-RFCA: 100% noninducible<br>ICD Present: 100%<br>Indications: VT storm (19.5%), recurrent ICD treatment (39%), documented MVT (41.4%)<br>Follow-up: median based on group 9–12 months (range: 3–13).<br>Complications: 0% (5 self-limited pericardial effusions)<br>RFCA approach: combined EPI-ENDO (substrate > conventional)	Short follow-up; inducibility as endpoint; all had ICD; 77% of symptomatic group had no arrhythmias or Type I pattern. Normal ECG can be seen in patient with RFCA, so unclear clinical endpoint.

Study name or acronym; Author; Year published; PMID	Aim of study; Endpoints; Study type (RCT, observational — multicenter or single center; case series or other [if RCT include intervention and comparator]); Study size (N)	Patient population with inclusion and exclusion criteria	Results (absolute event rates, *P* values; OR or RR; 95% CI)	Other relevant findings or adverse events	Limitations; Other comments; Conclusions
Zhang et al. Characterization of the epicardial substrate for catheter ablation of Brugada syndrome.<br>• Year published: 2016<br>• PMID: 27453126	Aim: To characterize substrate and mechanism of VAs and outcome of RFCA in BrS<br>Endpoints: Recurrence<br>Study type: Observational two-center prospective<br>Size: 11	Inclusion criteria:<br>- Consecutive<br>- Symptomatic Type I BrS-ECG with spontaneous or drug<br>- Documented VT/VF or syncope<br>- Clinical BrS, 1 additional clinical feature<br><br>Exclusion criteria: NA	Recurrence (VT/VF): 27%	Baseline: Drug-induced (18), spontaneous (82%)<br>Indication: VT/VF (82%), syncope (18%)<br><br>ICD: 72%<br><br>EAM: Abnormal EPI signals RVOT (100%),<br>prolonged EGM durations, low voltage<br><br>Normalization of spontaneous Type 1 ECG : 100%<br><br>EPS: Preablation inducible (82%), postablation inducible (9%)<br><br>Follow-up: 25 ± 11 months<br><br>No ICD (n=3): 1 Death, 2 VT-free (35 and 42 months)<br><br>ICD (n=8): 1 PMVT, 7 VT-free<br><br>1 death postablation, noninducible	Limited follow-up

Study name or acronym; Author; Year published; PMID	Aim of study; Endpoints; Study type (RCT, observational — multicenter or single center; case series or other [if RCT include intervention and comparator]); Study size (N)	Patient population with inclusion and exclusion criteria	Results (absolute event rates, *P* values; OR or RR; 95% CI)	Other relevant findings or adverse events	Limitations; Other comments; Conclusions
Brugada et al. Brugada syndrome phenotype elimination by epicardial substrate ablation.<br>• Year published: 2015<br>• PMID: 26291334	Aim: Methodology, outcome, and complications of EPI RFCA in BrS<br>Endpoints: Noninducibility, recurrence<br>Study type: Observational, single-center prospective<br>Size: 14	Inclusion criteria:<br>- Documented spontaneous or flecainide-induced Type I BrS ECG and symptoms attributed to VA<br>- VA induction at EPS<br>- ICD<br><br>Exclusion criteria: NA	Acute success (noninducibility): 100%<br><br>VF/VT Recurrence: 0%	Baseline: Type 1 (86%), Type 2 (14%) Symptoms: Syncope (71%), dizziness (29%)<br><br>ICD: 100%<br><br>Events on ICD preablation: NSVT (36%), PMVT (14%), VT (21%), VT/VF (21%), VF (7%).<br><br>EPS preablation inducible 100%: VT (14%), VT/VF (14%), VF(72%); EPS postablation inducible (0%).<br><br>EAM: Abnormal EPI signals RVOT/RV free wall (100%).<br><br>Normalization of Type 1 ECG even with flecainide: 100%<br><br>Follow-up: 5 months (3–6 months)<br><br>Complications: 0%	Short follow-up; inducibility as endpoint; all had ICD; normal ECG can be seen in patient with CA, so no clear clinical endpoint.

Study name or acronym; Author; Year published; PMID	Aim of study; Endpoints; Study type (RCT, observational — multicenter or single center; case series or other [if RCT include intervention and comparator]); Study size (N)	Patient population with inclusion and exclusion criteria	Results (absolute event rates, *P* values; OR or RR; 95% CI)	Other relevant findings or adverse events	Limitations; Other comments; Conclusions
Nademanee et al. Prevention of ventricular fibrillation episodes in Brugada syndrome by catheter ablation over the anterior right ventricular outflow tract epicardium.<br>• Year published: 2011<br>• PMID: 21403098	Aim: To characterize substrate of VAs and outcome of RFCA in BrS<br>Endpoints: Death or VF recurrence<br>Study type: Observational, prospective single center<br>Size: 9	Inclusion criteria:<br>- Symptomatic BrS with Type I pattern, drug or spontaneous<br>- Out of hospital cardiac arrest<br>- ICD<br>- Failed amiodarone<br><br>Exclusion criteria: NA	Acute success (noninducible VF): 78%<br><br>VF Recurrence: 11%	Baseline: Type 1 (78%), drug induced (22%);<br>4 median VF per month.<br><br>ICD: 100%<br><br>EPS preablation inducible (100%);<br>EPS postablation inducible (22%);<br>Ablation: EPI/ENDO<br><br>EAM: normal ENDO (100%), abnormal EPI (100%); delayed depolarization: anterior RVOT EPI.<br><br>Normalization of type 1 ECG: 56% during procedure, 89% during follow-up.<br><br>Follow-up: 20 months (12–33 months)<br><br>Complications: 0%	Limited follow-up

Ventricular Arrhythmia in Hypertrophic Cardiomyopathy

Study name or acronym; Author; Year published; PMID	Aim of study; Endpoints; Study type (RCT, observational — multicenter or single center; case series or other [if RCT include intervention and comparator]); Study size (N)	Patient population with inclusion and exclusion criteria	Results (absolute event rates, *P* values; OR or RR; 95% CI)	Other relevant findings or adverse events	Limitations; Other comments; Conclusions
Dukkipati et al. Long-term outcomes of combined epicardial and endocardial ablation of monomorphic ventricular tachycardia related to hypertrophic cardiomyopathy.<br>• Year published: 2011<br>• PMID: 21270104	Aim: Catheter ablation for the treatment of MMVT in patients with HCM<br>Study type: Muticenter, case series<br>Size: 10 patients	Inclusion criteria: Male patients with HCM-related MMVT, candidates for catheter ablation	Electrophysiological-identified epicardial scar was present in 8 (80%) patients, endocardial scar in 6 (60%), and no scar in 1 (10%). In the 5 patients with inducible, stable MMVT, 3 cases were successfully terminated with ablation from the epicardium and 1 from the endocardium. The case that failed catheter ablation required surgical cryoablation to abolish the incessant VT. In the remaining 5 patients, 4 underwent epicardial and endocardial ablation of sites with good pace maps and late or fractionated potentials. No ablation was performed in the remaining patient because of noninducibility and lack of identifiable scar. After 37 ± 17 months (limits, 2 to 62 months; median, 37 months), the freedom from recurrent ICD shocks was 78% (7 of 9 patients) in those who underwent ablation.	In highly selected patients with HCM, combined epicardial and endocardial mapping and ablation is a feasible and reasonably efficacious option for MMVT in refractory to aggressive trials of AADs and antitachycardia pacing.	Group of patients highly selected, and caution must be exercised in extrapolating these results to the general HCM population.

Study name or acronym; Author; Year published; PMID	Aim of study; Endpoints; Study type (RCT, observational — multicenter or single center; case series or other [if RCT include intervention and comparator]); Study size (N)	Patient population with inclusion and exclusion criteria	Results (absolute event rates, *P* values; OR or RR; 95% CI)	Other relevant findings or adverse events	Limitations; Other comments; Conclusions
Santangeli et al. Radiofrequency catheter ablation of ventricular arrhythmias in patients with hypertrophic cardiomyopathy: safety and feasibility.<br>• Year published: 2010<br>• PMID: 20493276	Aim: RFCA for the treatment of VTs in the setting of HCM<br>Size: 22 patients	Inclusion criteria: Patients (18 with ICD) with HCM and multiple episodes of VTs resistant to medical therapy	Mean patient age was 50.4 ± 15.3 years, and mean EF was 34.3% ± 9.8%. RFCA was performed endocardially in all patients, whereas epicardial RF applications were needed in 13 patients. A previous endocardial ablation was unsuccessful in 6 patients. At 20 ± 9 months of follow-up, elimination of VTs reached 73%.	No major complication was observed during and after the procedures in all patients.	Catheter ablation of VTs in patients with HCM refractory to medical therapy is safe, feasible, and successful in eliminating VT. Epicardial VT mapping and ablation should be considered as an important access option for the treatment of these patients to increase the success rate.
Ueda et al. Clinical and electrophysiological characteristics in patients with sustained monomorphic reentrant ventricular tachycardia associated with dilated-phase hypertrophic cardiomyopathy.<br>• Year published: 2012<br>• PMID: 22048994	Aim: Clinical characteristics and effectiveness of catheter ablation for SMVTs in patients with DHCM<br>Size: 5 patients	Inclusion criteria: Patients with DHCM (mean age; 67.0 years, male) who underwent catheter ablation for drug-refractory SMVTs	Endocardial ablation successfully eliminated all VTs in 2 patients. The remaining 3 patients needed epicardial ablation, intracoronary ethanol ablation, and surgical cryoablation. All but one VT arose from the basal septum, basal anterior to anterolateral left ventricle. Although the ECGs demonstrated similar features of idiopathic outflow or mitral annulus VTs reflecting the origins, there were characteristic multiple QRS deflections. Following the ablation, 4 (80%) of the 5 patients are free from VT recurrence during 18 months of follow-up.	In patients with DHCM, VT circuits predominantly distributed in the basal septum and the basal anterior to anterolateral left ventricle. In addition to the endocardial ablation, alternative approaches were required in some patients.	This study enrolled only a limited number of patients and the follow-up period was not long. Second, the relationship between the location of VT circuits and pathological findings could not be fully clarified because the histopathological analysis or CMR imaging was not obtained for all patients.

Study name or acronym; Author; Year published; PMID	Aim of study; Endpoints; Study type (RCT, observational — multicenter or single center; case series or other [if RCT include intervention and comparator]); Study size (N)	Patient population with inclusion and exclusion criteria	Results (absolute event rates, *P* values; OR or RR; 95% CI)	Other relevant findings or adverse events	Limitations; Other comments; Conclusions
Inada et al. Substrate characterization and catheter ablation for monomorphic ventricular tachycardia in patients with apical hypertrophic cardiomyopathy.<br>• Year published: 2011<br>• PMID: 20807280	Aim: To define the substrate and role of catheter ablation for VT in apical HCM<br>Size: 4 patients	Four patients with apical HCM and frequent, drug-refractory VT (mean age of 46 ± 10 years, LVEF 54% ± 14%) underwent catheter ablation with the use of EAM. Endocardial mapping was performed in 4 patients, and 3 patients underwent epicardial mapping.	In 3 patients, VT was related to areas of scar in the apical left ventricle, where maximal apical wall thickness ranged from 14.5 to 17.8 mm, and 2 patients had apical aneurysms. Endocardial and epicardial substrate mapping revealed low voltage (<1.5 mV) scar in both endocardial and epicardial LV in 2 and only in the epicardium in 1 patient. Inducible VT was abolished with a combination of endocardial and epicardial ablation in 2 patients, but was ineffective in the third patient, who had intramural reentry that required transcoronary ethanol ablation of an obtuse marginal vessel for abolition. The fourth patient had focal nonsustained repetitive VT from RVOT, consistent with idiopathic RVOT-VT, which was successfully ablated.	During follow-ups of 3–9 months, all patients remained free from VT.	Monomorphic VT in apical HCM can be due to endocardial, epicardial, or intramural reentry in areas of apical scar. Epicardial ablation or transcoronary alcohol ablation is required in some cases.

Study name or acronym; Author; Year published; PMID	Aim of study; Endpoints; Study type (RCT, observational — multicenter or single center; case series or other [if RCT include intervention and comparator]); Study size (N)	Patient population with inclusion and exclusion criteria	Results (absolute event rates, *P* values; OR or RR; 95% CI)	Other relevant findings or adverse events	Limitations; Other comments; Conclusions
Igarashi et al. Radiofrequency catheter ablation of ventricular tachycardia in patients with hypertrophic cardiomyopathy and apical aneurysm.<br>• Year published: 2018<br>• PMID: 30089559	Aim: To evaluate the characteristics and results of RFCA of VT in patients with HCM and LV apical aneurysm<br>Size: 15 patients	Fifteen patients with HCM and apical aneurysm who underwent RFCA for VT at 5 different institutions were included in this study. The data were evaluated retrospectively.	Endocardial voltage mapping showed an LVA, and late potential in the apical aneurysm was recorded in 12 patients (80%). Although epicardial or intramural origin of VT was suspected in 7 patients, endocardial RFCA successfully suppressed the VT at the LVA border (n = 10) or within the LVA (n = 2). In 2 of 3 patients without LVA at the endocardial site, linear RFCA at the anterior wall of the aneurysmal neck side was successful. In the remaining patient, endocardial RFCA of apical aneurysm was not effective, and epicardial RFCA site was needed.	In all patients, clinical VT became noninducible after RFCA. VT recurrence was observed in 2 patients (13.3%) during the 12-month follow-up period. One patient underwent a second endocardial RFCA, and no VT recurrence was noted. In the other patient, VT recurred 3 months after RFCA and was successfully terminated by antitachycardia pacing of the ICD.	In patients with HCM and apical aneurysm, endocardial RFCA of apical aneurysm effectively suppressed MMVT, which was related to apical aneurysm, and resulted in satisfactory outcomes.

Preprocedural Imaging

Study name or acronym; Author; Year published; PMID	Aim of study; Endpoints; Study type (RCT, observational — multicenter or single center; case series or other [if RCT include intervention and comparator]); Study size (N)	Patient population with inclusion and exclusion criteria	Results (absolute event rates, *P* values; OR or RR; 95% CI)	Other relevant findings or adverse events	Limitations; Other comments; Conclusions
**MRI in NICM and ICM**					
Siontis et al. Association of preprocedural cardiac magnetic resonance imaging with outcomes of ventricular tachycardia ablation in patients with idiopathic dilated cardiomyopathy.<br>• Year published: 2017<br>• PMID: 28603002	Aim: To assess benefit of MRI in VT RFCA in patients with NICM<br>Endpoint: VT elimination<br>Observational, case series	96 patients<br>Inclusion: only NICM<br>Comparisons of outcomes before vs after institution of an MRI protocol for patients with CIEDs<br><br>Some 41 patients had MRI vs 55 patients without MRI	Patients with MRI had higher procedural success (63% vs 24%, *P*<.001) and better survival free of composite outcome (VT recurrence, heart transplantation, or death): 73% vs 40% (log-rank *P*=.02).	Longer RF time in imaging group, presumably for longer RF applications to reach IM scarring.	Historic comparison group<br>- Preprocedural MRI improves outcomes for VT RF in NICM<br>- Limitation: not randomized
Zghaib et al. Standard ablation versus magnetic resonance imaging-guided ablation in the treatment of ventricular tachycardia.<br>• Year published: 2018<br>• PMID: 29330333	Aim: To compare MRI vs standard ablation without MRI in patients with VT and SHD<br>Endpoint: VT elimination<br>Observational, case series	24 patients<br>Inclusion: 15 NICM, 9 ICM	VT recurrence in 73% (9 in control and 7 in study group)<br><br>MRI-guided ablation was associated with reduced VT recurrence rate (HR 0.12; 95% CI 0.02–0.75; *P*=.02).		MRI-guided VT ablation reduced VT recurrence<br><br>Limitation: not randomized

Study name or acronym; Author; Year published; PMID	Aim of study; Endpoints; Study type (RCT, observational — multicenter or single center; case series or other [if RCT include intervention and comparator]); Study size (N)	Patient population with inclusion and exclusion criteria	Results (absolute event rates, *P* values; OR or RR; 95% CI)	Other relevant findings or adverse events	Limitations; Other comments; Conclusions
Andreu et al. Cardiac magnetic resonance-aided scar dechanneling: influence on acute and long-term outcomes.<br>• Year published: 2017<br>• PMID: 28760258	Aim: To compare MRI vs standard ablation without MRI in patients with SHD<br>Endpoint: VT elimination<br>Observational, case series	159 patients<br><br>Inclusion: 54 patients for MRI "aided" group; remainder: control group<br><br>Exclusion from MRI group: ICD implantation, contraindication to MRI, bad MRI image quality (18%)	No difference in acute outcome: non-inducibility 82%<br><br>VT recurrence 35%; MRI-aided group 18.5%; and conventional group 44%; log-rank *P*=.017.<br><br>Substrate accessibility and presence of "good-quality" preprocedural MRI were independent predictors for outcomes (for MRI, HR 0.48; 95% CI 0.24–0.96; *P*=.03).	Reduced mortality in MRI-aided group (9 patients died during mean of 20 months' follow-up vs 0 in MRI group), *P*=.02; no procedural mortality.	MRI-aided VT ablation reduced VT recurrence and reduced mortality during follow-up.<br><br>Limitation: not randomized
Bogun et al. Delayed-enhanced magnetic resonance imaging in nonischemic cardiomyopathy: utility for identifying the ventricular arrhythmia substrate.<br>• Year published: 2009<br>• PMID: 19324259	Aim: Assessment of MRI-guided mapping and ablation in patients with NICM<br>Endpoint: Elimination of VA<br>Observational, case series	29 patients with NICM and VAs<br>Inclusion: NICM, decreased EF<br>Exclusion: ICM, idiopathic VAs	In 14 patients with DE, the location of DE predicted outcomes: endocardial DE (n=5) endocardial ablation effective; epicardial DE (n=2) epicardial ablation effective; intramural DE (n=5) ablation ineffective.		DE MRI predicts location of arrhythmogenic tissue.<br><br>Limitation: not all forms of NICM were investigated

Study name or acronym; Author; Year published; PMID	Aim of study; Endpoints; Study type (RCT, observational — multicenter or single center; case series or other [if RCT include intervention and comparator]); Study size (N)	Patient population with inclusion and exclusion criteria	Results (absolute event rates, *P* values; OR or RR; 95% CI)	Other relevant findings or adverse events	Limitations; Other comments; Conclusions
Codrenau et al. Electroanatomic characterization of post-infarct scars comparison with 3-dimensional myocardial scar reconstruction based on magnetic resonance imaging.<br>• Year published: 2008<br>• PMID: 18755347	Aim: To compare EAM with DE-MRI<br>Endpoint: Correlation of voltage mapping and delayed enhancement<br>Observational, case series	10 patients with ICM and VAs<br>Inclusion: only ICM<br>Exclusion: other forms of cardiomyopathy	Reduced voltage (unipolar and bipolar) as well as electrogram duration correlated with the presence of DE-MRI and scar depth.<br><br>In 1 of 3 patients there was a mismatch between DE-MRI-defined vs EAM-defined scar.	Bipolar voltage cut-off <1.5 mV best correlated with DE-MRI-defined scar area (R=0.82).	EAM correlates with MRI-defined scar<br><br>Limitation: small sample size
Desjardins et al. Infarct architecture and characteristics on delayed enhanced magnetic resonance imaging and electroanatomic mapping in patients with postinfarction ventricular arrhythmia.<br>• Year published: 2009<br>• PMID: 19389653	Aim: To compare EAM with DE-MRI<br>Endpoint: Correlation of voltage mapping and delayed enhancement<br>Observational, case series	10 patients with ICM and VAs<br>Inclusion: only ICM<br>Exclusion: other forms of cardiomyopathy	Good correlation of EAM-defined scar and DE-MRI-defined scar.<br>Best voltage cut-off 1.0 mV bipolar, 5.8 mV unipolar (AUC 0.89 vs 0.88).<br>All critical areas at sites with DE.<br>Transmural scars can be differentiated from subendocardial scars using unipolar but not bipolar voltage.		Critical sites for post-MI VAs are confined to areas of DE; good correlation between EAM and DE-MRI<br><br>Limitation: small sample size

Study name or acronym; Author; Year published; PMID	Aim of study; Endpoints; Study type (RCT, observational — multicenter or single center; case series or other [if RCT include intervention and comparator]); Study size (N)	Patient population with inclusion and exclusion criteria	Results (absolute event rates, *P* values; OR or RR; 95% CI)	Other relevant findings or adverse events	Limitations; Other comments; Conclusions
Dickfeld et al. MRI-guided ventricular tachycardia ablation: integration of late gadolinium-enhanced 3D scar in patients with implantable cardioverter-defibrillators.<br>• Year published: 2011<br>• PMID: 21270103	Aim: Assessment of imaging-guided VT ablation in patients with ICD<br>Endpoint: VT elimination and correlation with MRI findings<br>Observational, case series	22 post-MI patients with ICDs<br>Inclusion: ICM and NICM with ICD<br>Exclusion: epicardial ICD systems, abandoned leads, CRI	No complications occurred while MRIs were obtained in the patients with ICDs.<br>Some 14 patients underwent VT ablation.<br>Good correlation between EAM and DE-MRI; bipolar cutoff 1.49 mV, unipolar cut-off 4.46 mV (AUC: 0.86 vs 0.78).<br>Some 78% of points with low voltage at sites without scar demonstrated inadequate catheter contact.	All effective VT target sites showed DE	EAM correlates well with DE-MRI findings.<br>MRI guided VT ablation is feasible and safe in patients with CMP and ICDs.<br>Limitations: artifacts of CIEDs limit the ability to fully assess the myocardium for scar.
Fernandez-Armenta et al. Three-dimensional architecture of scar and conducting channels based on high resolution ce-CMR: insights for ventricular tachycardia ablation.<br>• Year published: 2013<br>• PMID: 23685537	Aim: Identification of conducting channels on DE-MRI<br>Endpoint: VT elimination and correlation with MRI findings<br>Observational, case series	21 patients with ICM and VT<br>Inclusion: only ICM<br>Exclusion: other forms of cardiomyopathy	BZ channels containing conductive channels for VT were identified in most patients.<br>BZ: 40%-60% SI, core scar >60% SI<br>MRI-defined BZ channels identified 74% of critical VT sites, and 50% of the conductive channels were identified by MRI.<br>Moderate correlation between EAM-defined scar and MRI defined scar.<br>BZ channels in the epicardium were identified in patients with transmural infarcts only.		High-resolution MRI identifies BZ channels that often correlate with conductive channels critical for VT.<br><br>Limitations: Patients with ICDs were excluded.

Study name or acronym; Author; Year published; PMID	Aim of study; Endpoints; Study type (RCT, observational — multicenter or single center; case series or other [if RCT include intervention and comparator]); Study size (N)	Patient population with inclusion and exclusion criteria	Results (absolute event rates, *P* values; OR or RR; 95% CI)	Other relevant findings or adverse events	Limitations; Other comments; Conclusions
Andreu et al. Usefulness of contrast-enhanced cardiac magnetic resonance in identifying the ventricular arrhythmia substrate and the approach needed for ablation.<br>• Year published: 2014<br>• PMID: 24394378	Aim: Assessment of MRI-guided mapping and ablation<br>Endpoint: Elimination of VA<br>Observational, case series	80 patients with SHD and VAs<br>Inclusion: ICM and NICM<br>Exclusion: Idiopathic VAs, contraindications for MRI	VAs were successfully ablated in 96% of patients, all had DE.<br><br>Of the successful ablation sites, DE was absent in 4%, subendocardial DE was 25%, transmural DE was 47%, intramural DE was 10%, and subepicardial DE was 14%; epicardial DE had an 85% sensitivity and 100% specificity predicting an epicardial VA origin.		DE MRI predicts location of arrhythmogenic tissue and helps to plan the ablation procedure.
Gupta et al. Delayed-enhanced MR scar imaging and intraprocedural registration into an electroanatomical mapping system in post-infarction patients.<br>• Year published: 2012<br>• PMID: 22340829	Aim: To assess the feasibility of preprocedural scar identification and intraprocedural real-time image registration with an EAM<br>Size: 23 patients	23 patients with previous infarction and ventricular arrhythmias	Registration accuracy and cardiac MRI/EAM correlations were assessed, and critical areas for VA were correlated with the presence of scar. With a positional registration error of 3.8 ± 0.8 mm, 86% of LVAs of the EAM projected onto the registered scar. The DE-CMR-defined scar correlated with the area of low voltage (R = 0.82, *P*<.001).	All sites critical to VAs projected on the registered scar.	Selective identification and extraction of DE-CMR-defined scar followed by registration into a real-time mapping system are feasible and help to identify and display the arrhythmogenic substrate in postinfarction patients with VAs.

Study name or acronym; Author; Year published; PMID	Aim of study; Endpoints; Study type (RCT, observational — multicenter or single center; case series or other [if RCT include intervention and comparator]); Study size (N)	Patient population with inclusion and exclusion criteria	Results (absolute event rates, *P* values; OR or RR; 95% CI)	Other relevant findings or adverse events	Limitations; Other comments; Conclusions
Marra et al. Imaging study of ventricular scar in arrhythmogenic right ventricular cardiomyopathy: comparison of 3D standard electroanatomical voltage mapping and contrast-enhanced cardiac magnetic resonance.<br>• Year published: 2012<br>• PMID: 22139887	Aim: To compare endocardial voltage mapping and CE-CMR for imaging scar lesions in ARVC patients<br>Size: 23 patients	23 consecutive ARVC patients (16 males; mean age, 38 ± 12 years) who underwent RV EVM and CE-CMR and 37 controls	In 21 (91%) of 23 ARVC patients, RV EVM was abnormal, with a total of 45 EASs: 17 (38%) in the inferobasal region, 12 (26.6%) in the anterolateral region, 8 (17.7%) in the RVOT, and 8 (17.7%) in the apex. RV DCE was found in 9 (39%) of 23 patients, with a total of 23 RV DCE scars: 4 (17.4%) in the inferobasal region, 9 (39.1%) in the anterolateral region, 4 (17.4%) in the RVOT, and 6 (26.1%) in the apex. There was a mismatch in 24 RV scars, with 22 EAS not confirmed by DCE and 2 DCE scars (both in the RVOT) undetected by EVM. In 9 (75%) of 12 patients with abnormal RV EVM/normal RV DCE, ≥1 DCE was identified in the left ventricle.	Overall, ventricular DCE was detected in 78% of patients. No controls showed either EAS or DCE.	EVM and CE-CMR allow identification of RV scar lesions in most ARVC patients. CE-CMR is less sensitive than EVM in identifying RV scar lesions. The high prevalence of LV DCE confirms the frequent biventricular involvement and indicates the diagnostic relevance of LV scar detection by CE-CMR.
Soto-Iglesias et al. Image-based criteria to identify the presence of epicardial arrhythmogenic substrate in patients with transmural myocardial infarction.<br>• Year published: 2018<br>• PMID: 29427821	Aim: To assess whether infarct transmurality predicts epicardial arrhythmogenic substrate<br>Endpoint: Elimination of VA<br>Observational, case series	38 patients with ICM and VT: 10 patients with subendocardial scar underwent endocardial mapping; 28 patients with transmural scar underwent endocardial/epicardial mapping	Of 28 patients undergoing epicardial mapping, 18 had epicardial VTs and 10 did not. Epicardial scarring >14 cm^2 predicted patients with epicardial VT substrates (sensitivity 100%, specificity 100%).<br>Mean WT in patients with epicardial VTs in the epicardial scar was thinner (3.1 vs 5.5 mm) than in patients without epicardial VT.	WT cutoff ≤3.59 mm predicted presence of epicardial VT.	DE MRI identifies patients with ICM having epicardial VT origins.<br><br>Limitations: data from 3T MRI scanners
**CT for Preprocedural Imaging**					

Study name or acronym; Author; Year published; PMID	Aim of study; Endpoints; Study type (RCT, observational — multicenter or single center; case series or other [if RCT include intervention and comparator]); Study size (N)	Patient population with inclusion and exclusion criteria	Results (absolute event rates, *P* values; OR or RR; 95% CI)	Other relevant findings or adverse events	Limitations; Other comments; Conclusions
Yamashita et al. Image integration to guide catheter ablation in scar-related ventricular tachycardia.<br>• Year published: 2016<br>• PMID: 26918883	Aim: To describe feasibility and characteristics of CT/MRI image registration<br>Endpoint: Impact of image registration<br>Observational, case series	116 patients<br>Inclusion: ICM (n=67), ARVC (n=19), NICM (n=30)<br><br>Exclusion: Contraindication to CT or MRI: MRI not performed in 79 patients due to ICD implantation, CT not performed in 9 patients due to renal failure	CT less effective to demonstrate scar substrate compared with MRI in NICM.<br>In ICM, CT and MRI demonstrate scar in most patients (MRI > CT); image registration possible in all patients; imaging impacted on procedural planning in 33% (ie, epicardial mapping).	Agreement between LAVA and imaging data was better in ICM and ARVC compared with NICM (90%, 90%, and 73%, respectively).<br><br>Limitation: No comparison group to compare outcomes	Image integration feasible to guide ablations.<br>Ability to identify substrate by imaging is lower in NICM (especially if CT is used).<br>Imaging impacts on procedural planning
Ghannham et al. Correlation between computer tomography-derived scar topography and critical ablation sites in postinfarction ventricular tachycardia.<br>• Year published: 2018<br>• PMID: 29380921	Aim: To assess use of CT to localize arrhythmogenic structure in post-MI VT<br>Endpoint: VT elimination, correlation of mapping data with CT data<br>Observational, case series	15 patients<br>Inclusion: Patients with ICM	CT identifies ridges that correspond to critical VT sites; VT target sites were located on ridges for 84% of VTs; 70 CT-defined ridges were identified in the patients, 50% of them containing VT target sites.		CT is useful to identify VT target sites.

Study name or acronym; Author; Year published; PMID	Aim of study; Endpoints; Study type (RCT, observational — multicenter or single center; case series or other [if RCT include intervention and comparator]); Study size (N)	Patient population with inclusion and exclusion criteria	Results (absolute event rates, *P* values; OR or RR; 95% CI)	Other relevant findings or adverse events	Limitations; Other comments; Conclusions
Esposito et al. Cardiac CT with delayed enhancement in the characterization of ventricular tachycardia structural substrate: relationship between CT-segmented scar and electro-anatomic mapping.<br>• Year published: 2016<br>• PMID: 26897692	Aim: Comparison of CT-defined scaring with EAM<br>Endpoint: Registration of CT defined scar to EAM.<br>Observational, case series	42 patients with SHD: NICM: 45%	Scar with delayed enhancement in 36 of 40 patients, and in 27 of 36 patients WT present. WT was present in 3 of 42 patients. There was 76% sensitivity and 86% specificity of CT to identify LVAs on EAM.	RFCA targets in CT-defined scar in 79% of patients. Low voltage correlated with CT-defined scar.	Double phase CT helps to identify scar in NICM and ICM.
Tian et al. Three-dimensional contrast-enhanced multidetector CT for anatomic, dynamic, and perfusion characterization of abnormal myocardium to guide ventricular tachycardia ablations.<br>• Year published: 2010<br>• PMID: 20657032	Aim: Comparison of CT-defined abnormalities with EAM low voltage<br>Endpoint: Registration of CT-defined scar to EAM<br>Observational, case series	11 patients with ICM	CT hypoperfusion best correlated with low voltage in EAM. WT correlates with abnormal voltage.	Limitation: small series	Hypoperfusion on CT correlates with low voltage on EAM.

Study name or acronym; Author; Year published; PMID	Aim of study; Endpoints; Study type (RCT, observational — multicenter or single center; case series or other [if RCT include intervention and comparator]); Study size (N)	Patient population with inclusion and exclusion criteria	Results (absolute event rates, *P* values; OR or RR; 95% CI)	Other relevant findings or adverse events	Limitations; Other comments; Conclusions
Komatsu et al. Regional myocardial wall thinning at multidetector computed tomography correlates to arrhythmogenic substrate in postinfarction ventricular tachycardia: assessment of structural and electrical substrate.<br>• Year published: 2013<br>• PMID: 23476043	Aim: Comparison of WT and arrhythmogenic substrate on CT<br>Endpoint: Registration of CT-defined scar to EAM<br>Observational, case series	13 patients with ICM	WT of <5 mm correlated with low voltage on EAM.<br>Some 87% of sites with LAVA were within the area of <5 mm WT.		In ICM, CT-defined WT of <5 mm indicates low voltage in EAM.
**PET CT**					
Dickfeld et al. Integration of three-dimensional scar maps for ventricular tachycardia ablation with positron emission tomography-computed tomography.<br>• Year published: 2008<br>• PMID: 19356409	Aim: Correlation of PET-defined scar with EAM<br>Endpoint: Comparison of PET/CT-defined scar with EAM-defined scar<br>Observational, case series	14 patients with ICM	Scar location, size, and extent of PET-derived scar correlated well with EAM-defined scar.<br>Metabolically active channels were identified in 2 patients that were not identified in EAM.	Large positional error with registration of 8–11 mm in case of large scars.	Adequate correlation of PET/CT- defined scar with EAM-defined scar.

Study name or acronym; Author; Year published; PMID	Aim of study; Endpoints; Study type (RCT, observational — multicenter or single center; case series or other [if RCT include intervention and comparator]); Study size (N)	Patient population with inclusion and exclusion criteria	Results (absolute event rates, *P* values; OR or RR; 95% CI)	Other relevant findings or adverse events	Limitations; Other comments; Conclusions
Tian et al. Clinical application of PET/CT fusion imaging for three-dimensional myocardial scar and left ventricular anatomy during ventricular tachycardia ablation.<br>• Year published: 2009<br>• PMID: 19207761	Aim: Integration of PET/CT into EAM<br>Endpoint: Integration of PET/CT-defined scar to EAM<br>Observational, case series	10 patients with ICM, FDG, and Rubidium PET	Good correlation of EAM voltage map and FDG metabolism-defined scar. Metabolic characterization of scar and BZ; positional error: 4–6 mm.	Limitations: Segmentation of metabolic PET data requires special expertise and equipment; low spatial resolution of metabolically derived scar data.	Adequate correlation of PET/CT- defined scar with EAM-defined scar.
**Imaging and LV Thrombi**					
Visser et al. Two-dimensional echocardiography in the diagnosis of left ventricular thrombus: a prospective study of 67 patients with anatomic validation.<br>• Year published: 1983<br>• PMID: 6822107	Aim: Correlation of echocardiography with anatomy<br>Endpoint: Assessment for presence of LV thrombi<br><br>Observational, case series	72 patients with prior echocardiography undergoing aneuresmectomy or dying from MI.	Some 24 of 26 thrombi were correctly identified; absence of thrombi was correct in 36 of 41 patients (sensitivity 92%, specificity 88%).	Limitation: Adequate imaging in 67 of 72 patients.	2D echo can identify and exclude thrombi with good sensitivity and specificity.
Ezekowitz et al. Comparison of Indium-111 platelet scintigraphy and two-dimensional echocardiography in the diagnosis of left ventricular thrombi.<br>• Year published: 1982<br>• PMID: 7078607	Aim: Correlation of echocardiography and 111 indium-labeled platelet imaging with anatomy<br>Endpoint: Assessment for presence of LV thrombi<br><br>Observational, case series	53 patients who underwent surgery and had prior imaging for thrombi	Sensitivity and specificity of indium imaging and echocardiography to detect thrombi was 71% and 100% vs 77% and 92%, respectively.	Limitation: 25% of echocardiographies showed poor images.	2D echocardiography and indium imaging are useful to assess for LV thrombi.

Study name or acronym; Author; Year published; PMID	Aim of study; Endpoints; Study type (RCT, observational — multicenter or single center; case series or other [if RCT include intervention and comparator]); Study size (N)	Patient population with inclusion and exclusion criteria	Results (absolute event rates, *P* values; OR or RR; 95% CI)	Other relevant findings or adverse events	Limitations; Other comments; Conclusions
Stratton et al. Detection of left ventricular thrombus by two-dimensional echocardiography: sensitivity, specificity, and causes of uncertainty.<br>• Year published: 1982<br>• PMID: 7083502	Aim: Correlation of echocardiography with anatomy and indium imaging.<br>Endpoint: Assessment for presence of LV thrombi<br><br>Observational, case series	78 patients undergoing cardiac surgery/autopsy/ indium imaging with prior echocardiograms	The sensitivity, specificity, PPV, and NPV of echocardiography was 95%, 86%, 72%, and 98%, respectively.	Limitation: high false positive rate	High sensitivity to identify cardiac thrombi by 2D echocardiography.
Thanigaraj et al. Improved echocardiographic delineation of left ventricular thrombus with the use of intravenous second-generation contrast image enhancement.<br>• Year published: 1999<br>• PMID: 10588776	Aim: To determine value of contrast in patients with nondiagnostic echocardiographic studies.<br>Endpoint: Assessment for presence of LV thrombi<br><br>Observational, case series	409 patients in whom 48 patients had a contrast study because the echocardiogram study was nondiagnostic for assessment of LV thrombi	Some 43 of 48 (90%) studies were diagnostic after contrast administration.		Intravenous contrast enhancement increases yield of 2D echocardiography to assess for LV thrombi.

Study name or acronym; Author; Year published; PMID	Aim of study; Endpoints; Study type (RCT, observational — multicenter or single center; case series or other [if RCT include intervention and comparator]); Study size (N)	Patient population with inclusion and exclusion criteria	Results (absolute event rates, *P* values; OR or RR; 95% CI)	Other relevant findings or adverse events	Limitations; Other comments; Conclusions
Weinsaft et al. Detection of left ventricular thrombus by delayed-enhancement cardiovascular magnetic resonance prevalence and markers in patients with systolic dysfunction.<br>• Year published: 2008<br>• PMID: 18598895	Aim: To compare Cine CMR with delayed enhanced CMR for detection of cardiac thrombi<br>Endpoint: Assessment for presence of LV thrombi<br><br>Observational, case series	784 patients with LV dysfunction underwent Cine CMR vs delayed enhanced CMR for thrombus detection.	Prevalence of LV thrombus was 7% with DE-CMR vs 4.7% with Cine-CMR. DE-CMR more accurate for thrombus detection than Cine CMR (100% vs 40% for cases with pathologic validation). Cine CMR missed small intracavitary and large mural thrombi.<br>Presence of thrombi was associated with degree of LV dysfunction and scar volume (independent).		DE-MRI superior to Cine MRI to detect LV thrombi, especially small thrombi and mural thrombi.
Srichai et al. Clinical, imaging, and pathological characteristics of left ventricular thrombus: a comparison of contrast-enhanced magnetic resonance imaging, transthoracic echocardiography, and transesophageal echocardiography with surgical or pathological validation.<br>• Year published: 2006<br>• PMID: 16824834	Aim: To compare TTE, TEE, and CMR for thrombus detection in high-risk patient population<br>Endpoint: Assessment for presence of LV thrombus with pathologic or surgical validation<br><br>Observational, case series	361 patients with ICM undergoing evaluation for surgical ventricular reconstruction	LV thrombus present in 106 (29%) patients; 160 patients had all 3 imaging modalities; contrast-enhanced MRI had highest sensitivity and specificity compared with TTE and TEE: 88% and 99% vs 23% and 96% vs 40% and 96%, respectively.		Contrast-enhanced CMR has a higher sensitivity and specificity for thrombus detection than TTE and TEE in high-risk patients with ICM.

Study name or acronym; Author; Year published; PMID	Aim of study; Endpoints; Study type (RCT, observational — multicenter or single center; case series or other [if RCT include intervention and comparator]); Study size (N)	Patient population with inclusion and exclusion criteria	Results (absolute event rates, *P* values; OR or RR; 95% CI)	Other relevant findings or adverse events	Limitations; Other comments; Conclusions
Weinsaft et al. Contrast-enhanced anatomic imaging as compared to contrast-enhanced tissue characterization for detection of left ventricular thrombus.<br>• Year published: 2009<br>• PMID: 19679285	Aim: To compare echo with and without contrast using DE-CMR for thrombus detection<br>Endpoint: Assessment for presence of LV thrombus<br>Observational, case series	121 patients with LV dysfunction	Thrombus identified in 24 patients. Contrast echocardiography improved sensitivity from 33% to 61% and specificity from 82% to 92% compared with noncontrast echocardiography. Prevalence of thrombi was higher in DE-CMR compared with contrast echocardiography.	Limitations: Contrast echocardiography misses small thrombi or mural thrombi.	Contrast echocardiography in patients with high risk for thrombus improves detection of LV thrombi.

Anesthesia

Study name or acronym; Author; Year published; PMID	Aim of study; Endpoints; Study type (RCT, observational — multicenter or single center; case series or other [if RCT include intervention and comparator]); Study size (N)	Patient population with inclusion and exclusion criteria	Results (absolute event rates, *P* values; OR or RR; 95% CI)	Other relevant findings or adverse events	Limitations; Other comments; Conclusions
Servatius et al. Propofol sedation administered by cardiologists for patients undergoing catheter ablation for ventricular tachycardia.<br>• Year published: 2016<br>• PMID: 27247017	Aim: To assess the safety and efficacy of continuous propofol sedation for VT ablation administered, monitored, and controlled entirely by the operating cardiologist and his team, without intubation or assisted ventilation<br>Main endpoints: Primary endpoint change of sedation and/or discontinuation of propofol sedation due to adverse effects and/or persistent hemodynamic instability (systolic BP <70 mm Hg)<br>Secondary endpoints: temporary adverse effects of propofol sedation, full recovery within 30 minutes, procedural completion, and absence of any other complications.<br>Study type: Observational single center<br>Size: 205 consecutive procedures in 157 patients	Inclusion criteria: All patients eligible for VT ablation with an endocardial and epicardial substrate and VT episode duration of at least 30 seconds were screened for inclusion.<br><br>Exclusion criteria: Procedures primarily planned to be performed under general anesthesia, or if mechanical or inotropic support by norepinephrine, epinephrine, or dobutamine could be expected.	1. Propofol had to be discontinued (switched to midazolam) in 11.7% of the procedures, predominantly due to hypotension.<br>2. Data analysis revealed a significant though weak correlation between age and mean drop in SBP causing propofol discontinuation (R=0.22; R2=0.047; *P*=.002).<br>3. Procedure duration was longer in patients not tolerating propofol sedation (210 [180–260] vs 180 [125–220] minutes, *P*=.005).<br>4. Three (1.5%) patients experienced respiratory depression resulting in sustained oxygen saturation of <90%, requiring reduction of propofol and transitory mechanical maneuvers to assist ventilation.<br>5. Serious procedural complications occurred in 2.4% of procedures and were not related to the sedation regimen.	1. The average age of patients was 65.6 (54.8–71.3) years.<br>2. At screening, the median ASA score was 3 (3–4) and the LVEF was 36% (25%–55%).<br>3. The VTs were scar related in 135 cases.<br>4. Fentanyl was used for analgesia in 75% of patients and was administered in repeated boluses as required.	Limitations:<br>1. Data from a single center with the limitations expected from an observational study.<br>2. The exclusion criteria were not detailed but probably selected the patients with less severe disease.<br>3. VA induction was not included for analysis.<br><br>Conclusions:<br>Sedation using propofol can be safely performed for VT ablation under supervision of a cardiologist. Close hemodynamic monitoring is required, especially in elderly patients and during lengthy procedures carrying higher risk for SBP decline and an associated higher incidence of propofol interruption.

Study name or acronym; Author; Year published; PMID	Aim of study; Endpoints; Study type (RCT, observational — multicenter or single center; case series or other [if RCT include intervention and comparator]); Study size (N)	Patient population with inclusion and exclusion criteria	Results (absolute event rates, *P* values; OR or RR; 95% CI)	Other relevant findings or adverse events	Limitations; Other comments; Conclusions
Wutzler et al. Minimal and deep sedation during ablation of ventricular tachycardia.<br>• Year published: 2014<br>• PMID: 24447761	Aim: To evaluate the feasibility of minimal and deep sedation during VT ablation<br>Main endpoints: Changes of oxygenation and hemodynamic parameters, sedation-related adverse events, and incidence of complications were compared between the two sedation strategies<br>Study type: Observational single center<br>Size: 120 consecutive patients	Inclusion criteria: Consecutive patients with idiopathic or ischemic VT or VPC, in whom procedures were performed under minimal or deep sedation. Exclusion criteria: Patients who underwent procedures under GA (severe hemodynamic compromise, ES, severe life-threatening comorbidities, or acute illness).<br>Sedation strategies: Minimal sedation (42 patients): midazolam and opioid analgesic with repeated doses as needed.<br>Deep sedation (78 patients): continuous propofol infusion following an initial midazolam dose and opioid analgesic with repeated doses as needed.<br>Minimal or deep sedation were selected at the discretion of the operating physician. In general, idiopathic PVC/VT and stable patients with scar-related VT were performed under minimal sedation. Procedures in stable patients that are expected to have a longer duration or that require more painful and challenging techniques, such as epicardial access, were performed under deep sedation.	1. Significantly fewer patients in the deep sedation group underwent ablation for idiopathic VT (62.8% vs 88.1%; *P*=.011).<br>2. Mean LVEF was significantly lower in the deep sedation group (47% ± 14.4% vs 53.1% ± 11.7%; *P*=.014).<br>3. The procedure duration was significantly longer in the deep sedation group (201.9 ± 85.9 vs 137.9 ± 98.7 minutes; *P*=.001).<br>4. None of the patients received continuous catecholamine infusion to maintain hemodynamic function or required advanced airway management in both sedation strategies.<br>5. Serious procedural complications were not related to the sedation strategy.	1. Inducibility of the target arrhythmia was not significantly different between the sedation strategies (*P*=.67).<br>2. Epicardial ablation was required in 12.8% of patients and was performed under deep sedation.<br>3. Sedation level was changed from minimal to deep sedation in <10% of patients.	Limitations:<br>Data from a single center with the limitations expected from an observational study.<br><br>Conclusions:<br>Minimal sedation and deep sedation are both feasible and considerably safe during VPC/VT ablation procedures. Deep sedation can be performed during longer procedures with expected patient discomfort (eg, epicardial ablation). Minimal sedation can be sufficient for shorter procedures (eg, idiopathic VPC/VT). Adequate monitoring and trained personnel should be present to maintain patient safety.

Study name or acronym; Author; Year published; PMID	Aim of study; Endpoints; Study type (RCT, observational — multicenter or single center; case series or other [if RCT include intervention and comparator]); Study size (N)	Patient population with inclusion and exclusion criteria	Results (absolute event rates, *P* values; OR or RR; 95% CI)	Other relevant findings or adverse events	Limitations; Other comments; Conclusions
Ramoul et al. Conscious sedation with sufentanil and midazolam for epicardial VT ablation.<br>• Year published: 2012	Aim: To report the experience with epicardial VT ablation under conscious sedation (sufentanil + midazolam)<br>Study type: Observational single center<br>Size: 76 consecutive procedures in 72 patients	Inclusion criteria: All patients with epicardial VT ablation performed at the center in a period of time.<br><br>Exclusion criteria: Patients with respiratory failure, chronic obstructive pulmonary disease, allergy to morphine and coronary artery bypass graft surgery or intubated before the procedure for arrhythmia storm or cardiogenic shock were excluded from the study.<br><br>Sedation/analgesia strategy: During the preparation phase of the procedure an intravenous bolus of midazolam and paracetamol was given. Sufentanil (intravenous bolus) was administered just before the epicardial puncture with additional doses as needed to maintain satisfactory analgesia. Intravenous boluses of midazolam were repeated depending on the level of consciousness and anxiety.	1. No major analgesia-related complications (such as respiratory failure necessitating endotracheal intubation) were observed.<br>2. Because of pericardial bleeding, two patients were transferred to the operating room. One patient had developed metabolic acidosis (a patient with diabetes).	1. The average age was 56 ± 12 years and the LVEF was 38% ± 15%.<br>2. The mean duration of the procedure was 248 ± 90 minutes.<br>3. Epicardial ablation was performed in 60 cases with mean RF duration of 9 ± 11 minutes.	Limitations:<br>1. Data from a single center with the limitations expected from an observational study.<br><br>Conclusions:<br>Epicardial VT ablation under conscious sedation with sufentanil and midazolam is feasible and safe. This strategy prevents the need for general anesthesia and muscle relaxants (facilitating the identification of the phrenic nerve during epicardial ablation).

Study name or acronym; Author; Year published; PMID	Aim of study; Endpoints; Study type (RCT, observational — multicenter or single center; case series or other [if RCT include intervention and comparator]); Study size (N)	Patient population with inclusion and exclusion criteria	Results (absolute event rates, *P* values; OR or RR; 95% CI)	Other relevant findings or adverse events	Limitations; Other comments; Conclusions
Nazer et al. Importance of ventricular tachycardia induction and mapping for patients referred for epicardial ablation.<br>• Year published: 2015<br>• PMID: 26228002	Size: 25 patients	Of 68 NICM patients referred for VT ablation, 25 were referred specifically for epicardial ablation. All patients underwent PES under conscious sedation, with conversion to GA and epicardial access only if VT morphology and/or endocardial mapping suggested an epicardial substrate.	VT was induced with PES in 24 of 25 patients (mean age 52 years; 76% male; EF 38% ± 18%). VT was hemodynamically tolerated in 63% and unstable in 38% of patients. The patients with noninducible or unstable VT underwent substrate modification based on voltage and pace mapping. Of the patients with stable VT, 73% were mapped and ablated endocardially (6 right ventricle, 3 left ventricle, 1 LCC, 1 middle cardiac vein), and 33% were successfully ablated in areas of normal endocardial voltage. After ablation, the clinical VT was noninducible in all patients. After mean follow-up of 10 months, 80% were free of ICD shocks or SVT.	An initial approach of PES and entrainment mapping under conscious sedation is critically important for patients with NICM referred for epicardial ablation. Empiric ablation of endocardial or epicardial scar would have missed the clinical VT in 20% of patients.	

Study name or acronym; Author; Year published; PMID	Aim of study; Endpoints; Study type (RCT, observational — multicenter or single center; case series or other [if RCT include intervention and comparator]); Study size (N)	Patient population with inclusion and exclusion criteria	Results (absolute event rates, *P* values; OR or RR; 95% CI)	Other relevant findings or adverse events	Limitations; Other comments; Conclusions
Nof et al. Impact of general anesthesia on initiation and stability of VT during catheter ablation.<br>• Year published: 2015<br>• PMID: 26072026	Aim: To assess the SMVT inducibility by using a predefined PES protocol under GA vs IVCS<br>Main endpoints: Induction of SMVT lasting >30 seconds or requiring termination because of hemodynamic intolerance by using the predefined PES protocol<br>Study type: Observational single center<br>Size: 226 retrospective and 73 prospective patients	Retrospective data: Included a retrospective series of patients with SMVT and SHD with LVEF ≦40% who underwent VT RFCA. The predefined PES protocol was invasively performed from an RVA catheter before RFCA in 155 and 71 patients under IVCS and GA, respectively.<br>Prospective data: Included a prospective series of 73 patients with scar-related SMVT and ICD implanted who underwent VT RFCA. The predefined PES protocol was performed from the ICD (NIPS) prior to inserting catheters and under moderate sedation. Subsequently, GA was implemented, and the same PES protocol was invasively performed from an RVA catheter prior to RFCA<br>Sedation strategy: GA was inhaled anesthetic or continuous infusion of propofol at discretion of the anesthesiologist.	Retrospective comparison:<br>1. IVCS vs. GA patients did not show differences in clinical characteristics, VT inducibility, complications, or abolition of clinical VT.<br>2. Intravenous hemodynamic support was used more often in the GA group (83% vs 12%; *P*<.001).<br><br>Prospective comparison:<br>1. From the 73 included patients, VT was inducible with NIPS in 61 (84%), and just 5 (8%) of these became noninducible under GA.<br>2. Compared with VT induction during NIPS, during GA, VT induction required more aggressive stimulation in 41%.<br>3. After GA, 60 of 73 (82%) patients received pharmacologic hemodynamic support, with the vast majority (96%) receiving intravenous infusion of phenylephrine.	1. The retrospective comparison also showed that VTs were more often hemodynamically unstable with GA than with IVCS (70% vs. 51%; *P*=.007).<br>2. The prospective comparison showed that stable VTs induced by NIPS remained stable after GA in 87%, although all but one were receiving phenylephrine during GA.	Limitations:<br>1. Data from a single reference center with the limitations expected from an observational study.<br>2. The anesthetic agent was not identical due to practitioner variability. Different results could be expected when using different anesthetics.<br><br>Conclusions:<br>1. GA does not prevent inducible VT in the majority of patients.<br>2. GA is associated with more use of hemodynamic support (phenylephrine), but this did not adversely affect VT stability or procedure outcomes.

Vascular Access

Study name or acronym; Author; Year published; PMID	Aim of study; Endpoints; Study type (RCT, observational — multicenter or single center; case series or other [if RCT include intervention and comparator]); Study size (N)	Patient population with inclusion and exclusion criteria	Results (absolute event rates, *P* values; OR or RR; 95% CI)	Other relevant findings or adverse events	Limitations; Other comments; Conclusions
**Vascular Access: Use of Ultrasound**					
Sharma et al. Vascular complications during catheter ablation of cardiac arrhythmias: a comparison between vascular ultrasound guided access and conventional vascular access.<br>• Year published: 2016<br>• PMID: 27433795	Occurrence of vascular complications including bleeding or hematoma; arterio-venous fistula and/or pseudoaneurysm within a 30-day postprocedure period<br><br>Secondary endpoints: All-cause mortality during follow-up<br><br>Cohort study<br>N=689	Consecutive patients who underwent RFCA of AF/VT/PVC at the Hospital of the Virginia Commonwealth University between October 2014 and May 2015 (without US) and between June 2015 and January 2016 (with US).	After a mean 21 ± 7 months of follow up, 30 (16%) patients died.<br>Incidence of all vascular access-related complications was significantly higher in the non-US group compared with the US group (19 [5.3%] vs. 4 [1.1%]; *P*=.002).<br>Major vascular access complications were also significantly higher in the non-US group (9 [2.5%] vs. 2 [0.6%]; *P*=.03).<br>Lower incidence of minor bleeding/hematomas (BARC 1) (2 vs. 10; *P*=.02) and lower incidence of major bleeding (BARC 2+) (0 vs. 4 [1.1%]; *P*=.04) in the US group compared with the non-US group.<br>ARR of 2.2% with an RRR of 79%.	Age 58 ± 16 years, 47% female; average BMI was 30 ± 7 (15–62) kg/m^2.<br><br>Ablation for VT/PVC accounted for 89 (12%) procedures.<br><br>The overall incidence of all vascular access-related complications was 4.5% among patients undergoing VT/PVC ablations.<br><br>The rate of major femoral access-related complications was 2.2% among patients undergoing VT/PVC ablations.	The main limitation of this study was inherent to its observational nature.<br><br>Institution is a tertiary referral center for catheter ablation; as such, the study cohort is affected by an unavoidable degree of referral bias, and the results might not be generalized to other institutions.<br><br>Routine use of US-guided vascular access during EP procedures was associated with a significant reduction in all 30-day risk of overall complications and major vascular complication.

Study name or acronym; Author; Year published; PMID	Aim of study; Endpoints; Study type (RCT, observational — multicenter or single center; case series or other [if RCT include intervention and comparator]); Study size (N)	Patient population with inclusion and exclusion criteria	Results (absolute event rates, *P* values; OR or RR; 95% CI)	Other relevant findings or adverse events	Limitations; Other comments; Conclusions
Tanaka-Esposito et al. Real-time ultrasound guidance reduces total and major vascular complications in patients undergoing pulmonary vein antral isolation on therapeutic warfarin.<br>• Year published: 2013<br>• PMID: 23585239	1. Observational, single center<br>2. Use of ultrasound during femoral venous cannulation reduces vascular complications in patients undergoing AF ablation<br>3. Study compared the vascular complication rates of patients undergoing PVAI with routine use of US to visually guide venipuncture	1. Consecutive patients who underwent catheter-based pulmonary vein isolation for AF ablation at the Cleveland Clinic<br>2. Patients before 2007 who underwent anatomic-based venipuncture (non-US group, n=1909) were the control group. 3. Patients between July 2008 and May 2010 who underwent ultrasound-guided venipuncture (US group, n=1511) were the intervention group.	1. Use of ultrasound guidance resulted in significant reductions in total and major vascular access-related bleeding complications. 2. Despite the more prevalent use of warfarin in the US group compared with the non-US group, total vascular complications were reduced, reflected in the higher mean INR (2.3 ± 0.02 vs 1.3 ± 0.02; *P*<.01) and greater rate of patients with an INR ≥1.2 (91% vs. 14%; *P*<.01)<br>3. Overall, 8 (0.5%) patients in the US group and 32 (1.7%) patients in the non-US group experienced a vascular complication; *P*≤.01.	In the non-US group, warfarin use was associated with a 3.5-fold increase in vascular complications (*P*<.01).	1. Single center, nonrandomized design and the use of historical controls<br>2. Conclusion: Real-time ultrasound guidance for central venous access significantly improves the safety profile of PVAI by reducing the risk of overall and major vascular complications.

Study name or acronym; Author; Year published; PMID	Aim of study; Endpoints; Study type (RCT, observational — multicenter or single center; case series or other [if RCT include intervention and comparator]); Study size (N)	Patient population with inclusion and exclusion criteria	Results (absolute event rates, *P* values; OR or RR; 95% CI)	Other relevant findings or adverse events	Limitations; Other comments; Conclusions
Yamagata et al. Ultrasound-guided versus conventional femoral venipuncture for catheter ablation of atrial fibrillation: a multicentre randomized efficacy and safety trial (ULTRA-FAST trial).<br>• Year published: 2018<br>• PMID: 28575490	1. Multicenter randomized controlled trial.<br>2. Evaluate the efficacy and safety of US-guided venipuncture of femoral veins in patients undergoing catheter ablation for AF without cessation of oral anticoagulants.<br>Endpoints: The primary endpoint was the rate of major vascular access complications defined as (i) hematoma, (ii) arteriovenous fistula, or (iii) pseudoaneurysm that required interventions such as transfusion or surgical repair, and/or that resulted in prolongation of the hospital stay or hospital readmission.	Between March 2016 and November 2016, 323 patients were enrolled in the study. Inclusion criteria: older than 18 years, scheduled for catheter ablation for AF pulmonary vein isolation while using uninterrupted oral anticoagulation therapy. For new oral anticoagulants, the patient skipped the drug on the day of the procedure. The target prothrombin time (INR) for those who were taking warfarin was 2.0—3.0. Inclusion in the study was offered to all consecutive patients who were eligible for the study. Exclusion criteria: patients with history of problematic vascular groin access.	1. No significant difference in the complication rates between the US-guided and conventional group was observed (0.6% vs 1.9%, respectively; *P*=.62<br>2. For secondary objectives, the US-guided group showed significantly shorter puncture times and fewer cases of unsuccessful cannulation, extra puncture attempts, inadvertent arterial puncture, and/or use of X-ray.	1. Trend toward fewer patients with pain scale >3 in the US-guided group (2% vs 6%; *P*=.08).<br>2. The US-guided group presented with significantly higher first pass success.<br>3. All intraprocedural measures were significantly in favor of US-guided venipuncture in the subgroup of trainees.	Limitations<br>1. Unexpectedly low vascular complication rate in the trial despite the substantial proportion of venipunctures performed by trainees.<br>2. Cross-over was allowed per study protocol, which might also diminish the differences between the study arms that were analyzed by intention-to-treat principle.<br>Conclusion: Ultrasound-guided puncture of femoral veins was associated with preferable intraprocedural outcomes, though the major complication rates were not reduced. Both trainees and expert operators benefited from the US-guided strategy.

Study name or acronym; Author; Year published; PMID	Aim of study; Endpoints; Study type (RCT, observational — multicenter or single center; case series or other [if RCT include intervention and comparator]); Study size (N)	Patient population with inclusion and exclusion criteria	Results (absolute event rates, *P* values; OR or RR; 95% CI)	Other relevant findings or adverse events	Limitations; Other comments; Conclusions
Sobolev et al. Ultrasound-guided cannulation of the femoral vein in electrophysiological procedures: a systematic review and meta-analysis.<br>• Year published: 2017<br>• PMID: 27207813	Meta-analysis to determine the utility of real-time US guidance for femoral vein access in EP procedures.	A comprehensive literature search of Medline, Embase, Google Scholar, and the Cochrane Central Register of Controlled Trials	1. Total of 4065 patients were included in the review, with 1848 subjects in the US group and 2217 patients in the standard of care group.<br>2. Compared with standard of care methods, US guidance for femoral vein cannulation was associated with a 60% reduction in major vascular bleeding.	There was a 66% reduction in minor vascular complications (relative risk 0.34; 95% CI 0.15–0.78).	The use of real-time 2D US guidance for femoral vein cannulation during EP procedures decreases life-threatening vascular complications and access-related bleeding rates.

Study name or acronym; Author; Year published; PMID	Aim of study; Endpoints; Study type (RCT, observational — multicenter or single center; case series or other [if RCT include intervention and comparator]); Study size (N)	Patient population with inclusion and exclusion criteria	Results (absolute event rates, *P* values; OR or RR; 95% CI)	Other relevant findings or adverse events	Limitations; Other comments; Conclusions
Seto et al. Real-time ultrasound guidance facilitates femoral arterial access and reduces vascular complications: FAUST (Femoral Arterial Access With Ultrasound Trial).<br>• Year published: 2010<br>• PMID: 20650437	Multicenter, prospective, single- blinded, randomized controlled trial. Utility of US guidance in femoral arterial access. Primary end point of the study was successful cannulation of the CFA, defined as above the femoral bifurcation and below the origin of the inferior epigastric artery.	Inclusion: Patients older than 18 years of age scheduled to undergo a diagnostic or interventional coronary or peripheral procedure from the retrograde femoral arterial approach were eligible for enrolment in the trial. Patient enrolment required the availability of a dual-trained primary operator, US machine, and research coordinator, and thus was non-consecutive.<br><br>Exclusion: (1) had nonpalpable femoral pulses; (2) had a creatinine of ≥3.0 mg/dL, unless already receiving dialysis; (3) had an ST-segment elevation MI or unstable non-ST-segment elevation MI; (4) were pregnant; or (5) were incarcerated.	1. Between April 2008 and February 2009, 1015 patients were enrolled and randomized.<br>2. After exclusion, 1004 patients were assigned to either fluoroscopic (n=501) or US (n=503) guidance and included in the analysis of intraprocedural and clinical outcomes.<br>3. Clinical access complications occurred in 17 of 501 (3.4%) patients in the fluoroscopy group, compared with 7 of 503 (1.4%) in the US-guided group (*P*=.041).	No patients suffered from access site infection, arteriovenous fistula formation, or retroperitoneal hemorrhage at 30 days. No patient had a significant decrease in hemoglobin in the absence of transfusion.	Limitations<br>1. Blinding of the operator and catheterization lab personnel to the study intervention was not possible.<br>2. Could not completely exclude a bias in the performance or measurement of the number of attempts, venipunctures, or time to access.<br><br>Conclusions: US guidance facilitated successful arterial access and reduced clinical complications. The rate of sheath insertion into the CFA was increased in patients with a high CFA bifurcation but not in the overall population. US guidance should be considered for any patient at high risk for a difficult access or complications.
**Vascular Access Type — Retrograde vs Transseptal Complications**					

Study name or acronym; Author; Year published; PMID	Aim of study; Endpoints; Study type (RCT, observational — multicenter or single center; case series or other [if RCT include intervention and comparator]); Study size (N)	Patient population with inclusion and exclusion criteria	Results (absolute event rates, *P* values; OR or RR; 95% CI)	Other relevant findings or adverse events	Limitations; Other comments; Conclusions
Stevenson et al. Irrigated radiofrequency catheter ablation guided by electroanatomic mapping for recurrent ventricular tachycardia after myocardial infarction: the multicenter thermocool ventricular tachycardia ablation trial.<br>• Year published: 2008<br>• PMID: 19064682	Primary endpoint: At 6 months of follow-up as no recurrence of sustained monomorphic VT.<br><br>RCT, N=231	Study conducted between February 1999 and December 2003.<br>SMVT requiring termination by cardioversion or AAD administration with >4 episodes in the previous 6 months despite an ICD or AAD therapy.<br>Patients without ICDs were eligible after 2 episodes of sustained VT.<br>Exclusion criteria: serum creatinine >2.5 mg/dL; LVEF <0.10; mobile LV thrombus on echocardiography; absence of vascular access to the left ventricle; disease process likely to limit survival to <12 months; NYHA class IV HF; cardiac surgery within the past 2 months (unless VT was incessant); unstable angina; severe aortic stenosis or mitral regurgitation with a flail leaflet; pregnancy; and age <18 years.	Median age 68 years; 89% men; 40 patients died. The 1-year actuarial mortality rate was 18%. Seven (3%) patients died within 7 days of the procedure. In 6 patients, death was preceded by uncontrollable VT with progressive hypotension or cardiac arrest. Complications related to vascular access (femoral hematomas or pseudoaneurysms) occurred in 4.7% of patients.	VT was reduced from a median of 11.5 episodes to a median of 0 episodes per 6 months (lower quartile, 0; upper quartile, 7) (*P*=.0001).<br><br>The frequency of VT was reduced by >75% in 67% of patients.<br><br>An increase in the number of VT episodes was observed in 20% of patients.<br><br>At last follow-up, 28% of patients were no longer receiving AAD therapy.	The patient population was selected by referral for ablation, resulting in a group of patients with advanced heart disease.<br>The follow-up period was short: only 6 months for assessment of recurrent arrhythmias and 12 months for death. Although continued antiarrhythmic drug therapy was recommended, drugs were often reduced after a successful ablation, as allowed for adverse effects.<br>In some cases, removal of a proarrhythmic drug effect could have contributed to a beneficial outcome; in other cases, emergence of a VT that was suppressed by the drug at the time of ablation could have contributed to procedure failure.<br>Catheter ablation is a reasonable option to reduce episodes of recurrent VT in patients with prior MI. Multiple and unmappable VTs can be targeted with ablation combined with electroanatomic substrate mapping with acceptable risks and outcomes.

Study name or acronym; Author; Year published; PMID	Aim of study; Endpoints; Study type (RCT, observational — multicenter or single center; case series or other [if RCT include intervention and comparator]); Study size (N)	Patient population with inclusion and exclusion criteria	Results (absolute event rates, *P* values; OR or RR; 95% CI)	Other relevant findings or adverse events	Limitations; Other comments; Conclusions
Sapp et al. Ventricular tachycardia ablation versus escalation of antiarrhythmic drugs.<br>• Year published: 2016<br>• PMID: 27149033	Primary outcome: Death occurring at any time after randomization or VT storm or appropriate ICD shock after a 30-day treatment period.<br><br>Secondary outcome: each of the components of the primary outcome and adverse effects<br><br>RCT, N=259	Catheter ablation for recurrent, drug refractory VA.<br><br>A total of 132 patients were assigned to the ablation group and 127 to the escalated therapy group.	Major bleeding (3 patients vs 1 patient; *P*=.62), vascular injury (3 patients vs 0 patients; *P*=.25), cardiac perforation (2 patients vs 1 patient; *P*=1.00), and heart block (1 patient vs 0 patients; *P*=.49).	Mean (±SD) 27.9 ± 17.1 months of follow-up.<br><br>Rate of the primary outcome was significantly lower in the ablation group than in the escalated therapy group.<br><br>Some 36 (27.3%) patients in the ablation group and 35 (27.6%) in the escalated therapy group died.<br><br>VT storm occurred in 32 (24.2%) patients in the ablation group and 42 (33.1%) patients in the escalated therapy group.<br><br>Appropriate ICD shocks occurred in 50 (37.9%) patients and 54 (42.5%) patients in the ablation and escalated therapy groups, respectively (HR 0.77; 95% CI 0.53–1.14; *P*=.19).	Not powered to assess the effect of the two treatments on mortality.<br><br>Vascular complications: 2%

Study name or acronym; Author; Year published; PMID	Aim of study; Endpoints; Study type (RCT, observational — multicenter or single center; case series or other [if RCT include intervention and comparator]); Study size (N)	Patient population with inclusion and exclusion criteria	Results (absolute event rates, *P* values; OR or RR; 95% CI)	Other relevant findings or adverse events	Limitations; Other comments; Conclusions
**Better Contact With Different Approach**					
Tilz et al. In vivo left-ventricular contact force analysis: comparison of antegrade transseptal with retrograde transaortic mapping strategies and correlation of impedance and electrical amplitude with contact force.<br>• Year published: 2014<br>• PMID: 24493339	Cohort study, N=10	Catheter ablation for recurrent, drug refractory VA.<br><br>Five patients underwent ablation for ischemic VT: 4 patients for idiopathic premature ventricular contractions and VT, and 1 patient for VT following myocarditis.<br><br>The mean EF was 45% ± 14%, and the mean LVEDD was 59 ± 10 mm.<br><br>Exclusion criteria included prior LV ablation procedures and paced ventricular rhythm.	Mean CF was significantly higher when using the antegrade approach in the midanteroseptum, midlateral, and apex, and significantly higher when using the retrograde approach in the basal anteroseptum, basal inferoseptum, basal inferior, and basal lateral segments.	Median antegrade and retrograde CF were 20.2 ± 16.0 and 19.1 ± 14.0 g, respectively (*P*=.048).<br><br>There was no statistically significant difference between the antegrade and retrograde LV maps for the parameters of LV volume, LV surface area, and LV LVA. Mapping duration also showed no significant difference.	This study evaluated CF during mapping rather than ablation, when it might be more important. Stability and consistency of contact, which are also important during ablation, were not assessed.

Intraprocedural Hemodynamic Support

Study name or acronym; Author; Year published; PMID	Aim of study; Endpoints; Study type (RCT, observational — multicenter or single center; case series or other [if RCT include intervention and comparator]); Study size (N)	Patient population with inclusion and exclusion criteria	Results (absolute event rates, *P* values; OR or RR; 95% CI)	Other relevant findings or adverse events	Limitations; Other comments; Conclusions
Miller et al. Activation and entrainment mapping of hemodynamically unstable ventricular tachycardia using a percutaneous left ventricular assist device.<br>• Year published: 2011<br>• PMID: 21920266	To investigate the effects of PLVAD support during catheter ablation of unstable VT. Procedural monitoring included vital signs, left atrial pressure, arterial BP, cerebral perfusion/oximetry, VT characteristics, and ablation outcomes.<br><br>Nonrandomized, retrospective study compared PLVAD (Impella 2.5) with no PLVAD (IABP/no mechanical support); 23 patients: PLVAD (N=10); no PLVAD (N=13):<br>IABP (6), no mechanical support (7).	SHD, at least 1 hemodynamically unstable VT (map < 45 mm Hg) sustained VT and/or recurrent ICD shocks.	1. At least 1 VT was terminable by RFCA during ongoing VT in 9 of 10 (90%) PLVAD patients and 5 of 13 (39%) non-PLVAD patients (*P*=.03).<br>2. The PLVAD group was in VT 2.5 times longer than the non-PLVAD group and required fewer premature terminations of ongoing VT.<br>3. Baseline mean LAP was increased for the entire cohort (14.2 ± 4.8 mm Hg).<br>4. No difference in total duration of RFCA, postprocedure inducibility, and end-organ perfusion surrogates and VT recurrences within 3 months.	Two cases of cardiac tamponade (1 PLVAD, 1 IABP), both recovering uneventfully.	• Electromagnetic interference<br>• Retrospective<br>• Nonrandomized<br>• Small number of patients<br>• Limited follow up<br>• Costs<br><br>PLVAD is superior to no PLVAD to support nontolerated VTs.

Study name or acronym; Author; Year published; PMID	Aim of study; Endpoints; Study type (RCT, observational — multicenter or single center; case series or other [if RCT include intervention and comparator]); Study size (N)	Patient population with inclusion and exclusion criteria	Results (absolute event rates, *P* values; OR or RR; 95% CI)	Other relevant findings or adverse events	Limitations; Other comments; Conclusions
Reddy et al. Percutaneous left ventricular assist devices in ventricular tachycardia ablation: multicenter experience.<br>• Year published: 2014<br>• PMID: 24532564	To evaluate the relative safety and efficacy of using IABP versus non-IABP (Impella or TandemHeart) for unstable VT ablation in a multicenter study.<br><br>To allow activation mapping of unstable VTs and to achieve VT termination by ablation.<br><br>Multicenter, observational study from a prospective registry<br>IABP vs non-IABP (Impella 2.5/Tandem Heart)<br>66 patients: IABP (N=22) vs non-IABP (N=44, Impella [25]/ TandemHeart [19]).	All consecutive patients who underwent VT ablation with a PLVAD in 6 participating centers across the United States between March 2006 and December 2011.	In the non-IABP group compared with the IABP group:<br>1. More patients could undergo entrainment/activation mapping.<br>2. More unstable VTs could be mapped and ablated per patient.<br>3. More VTs could be terminated by ablation.<br>4. Fewer VTs were terminated with rescue shocks.<br>5. Mortality and VT recurrence during 12 ± 5-month follow-up were not different between both groups.	Some 17 (26%) patients had ≥1 major complication during the hospitalization;<br>1 pericardial tamponade/effusion requiring drainage (non-IABP),<br>1 vascular complication requiring intervention (non-IABP),<br>1 MI (non-IABP),<br>1 hematoma (2 IABP, 6 non-IABP),<br>11 (17%) in-hospital deaths; no significant difference.	• Small patient population and observational<br>• The selection PLVAD/ablation techniques operator dependent<br>• LVEF could be a reflection of acute HD<br>• Cerebral oximetry/transcranial Doppler were not used for determining the hemodynamic instability<br>• Access to the LV was driven by the type of PLVAD<br><br>PLVAD is superior to no PLVAD to support nontolerated VTs.

Study name or acronym; Author; Year published; PMID	Aim of study; Endpoints; Study type (RCT, observational — multicenter or single center; case series or other [if RCT include intervention and comparator]); Study size (N)	Patient population with inclusion and exclusion criteria	Results (absolute event rates, *P* values; OR or RR; 95% CI)	Other relevant findings or adverse events	Limitations; Other comments; Conclusions
Baratto et al. Extracorporeal membrane oxygenation for hemodynamic support of ventricular tachycardia ablation.<br>• Year published: 2016<br>• PMID: 27932426	Safety, efficacy of ECMO-supported VT ablation.<br><br>Activation mapping of unstable VTs and ablation success.<br><br>Incidence of periprocedural heart decompensation and acute death.<br><br>Long-term outcome of ECMO-supported VT catheter ablation.<br><br>Single-center, prospective, observational<br>64 patients (74 unstable VT).	1. Bailout (cardiogenic shock)<br>2. Preemptive<br>• Incessant VT/ES leading to cardiogenic shock<br>• High-risk and nontolerated VTs<br>• Previous ineffective substrate-based procedure in patients with nontolerated VTs	Preemptive strategy in 59 of 64 patients (92%); bailout strategy in 5 of 64 patients (8%). MAP >70 mm Hg during VT with ECMO in 58 (78%) procedures; MAP 60–70 mm Hg in 14 (19%) procedures; MAP <60 mm Hg in 2 (3%) procedures with adjunctive Impella 2.5.<br>• The 5 patient with bailout ECMO had faster VTs (215 ± 30 vs 293 ±61 milliseconds; *P*=.005), a lower MAP (67 ± 4 vs 76 ± 12 mm Hg; *P*=.005), despite higher adrenaline (0.19 ± 0.06 vs 0.07 ± 0.06 µg/kg/min; *P*=.012), and a lower intraprocedural minimum pH<br>value (7.31 ± 0.04 vs 7.39 ± 0.06; *P*=.006).<br>• VT mapping and at least one VT termination during RF in 48 of 61 patients (79%).<br>• Prevention of inducibility of any VT in 51 (69%) procedures.<br>• After follow-up of 23 ± 13 months (6–72 months), 21 (33%) patients had VT recurrence; overall mortality was 8 (12%), related to HF in 3 (5%) and to VT in 1 (1.5%) after 4 months; 3 (5%) patients had heart transplant; 5 (8%) had LVAD implantation at 3 ± 2 months.<br>• In the multivariable analyses, LVEF (HR 0.916; *P*=.008) and postablation noninducibility (HR 0.198; *P*=.001) correlated with all-cause death, heart transplantation, and LVAD.	• Transient postprocedural increase of median AST<br>• 4 (6%) patients with transient renal failure (3 AKIN stage 1 and 1 stage 2)<br>• 2 Acute peripheral artery ischemia; both recovered uneventfully<br>• HF in 5 (8%) patients, leading to death in 1 (1.5%)<br>• 3 (5%) heart transplants<br>• 1 (1.5%) permanent LVAD	• Lack of comparative data<br>• Single-center experience in a small cohort of consecutive patients.<br><br>In select patients, ECMO as a support for VT ablation in high-risk patients is a safe strategy and allows successful VT ablation. It allows extended periprocedural circulatory support and has low incidence of AHD and acute death.

Study name or acronym; Author; Year published; PMID	Aim of study; Endpoints; Study type (RCT, observational — multicenter or single center; case series or other [if RCT include intervention and comparator]); Study size (N)	Patient population with inclusion and exclusion criteria	Results (absolute event rates, *P* values; OR or RR; 95% CI)	Other relevant findings or adverse events	Limitations; Other comments; Conclusions
Aryana et al. Outcomes of catheter ablation of ventricular tachycardia with mechanical hemodynamic support: an analysis of the Medicare database.<br>• Year published: 2017<br>• PMID: 28800178	Investigate several in-hospital and posthospitalization benchmark measures of quality associated with mechanical support with and without PVAD: Incidence of heart failure and renal failure; length of hospital stay; hospitalization cost; all-cause, HF-related renal failure related to 30-days hospital readmission; overall mortality index; mortality in patients with cardiogenic shock; repeat ablation at 1 year. Retrospective evaluation of data from a multicenter registry.<br>VT ablation performed with PVAD vs IABP.<br>230 PVAD and 115 IABP.	All patients who underwent catheter ablation of VT associated with the use of either PVAD or IABP from 2010 to 2013 using the International Classification of Diseases, Ninth Revision, Clinical Modification (ICD-9-CM) as captured by the Medicare IPSAF database.	1. PVAD was associated with an increased incidence of preprocedural HF; a higher incidence of post-catheter ablation HF (84.3% vs 73.0%; *P*=.01), but a similar incidence of renal disease (33.0% vs 37.4%; *P*=.42), with a lower incidence of in-hospital cardiogenic shock (9.1% vs 23.5%; *P*<.001) and renal failure (11.7% vs 21.7%; *P*=.01) and a shorter hospitalization time (8.4 ± 7.9 vs 10.6 ± 7.5; *P*<.001), but with similar mean ($66,058 ± $12,522 vs $74,587 ± $13,027; *P*=0.37) and median ($59,601 vs $53,717) associated total hospitalization costs.<br>2. The overall mortality index (6.5% vs 19.1%; *P*=.001) and mortality in those with cardiogenic shock (18.2% vs 41.2%; *P*=.03) were both considerably lower in the PVAD group.<br>3. PVAD was associated with lower all cause and heart failure-related 30-day hospital readmissions. There was no significant difference with regard to 30-day renal failure-related readmissions, and to the rate of VT repeat ablation at 1 year.	No differences in complications: vascular (4 in PVAD, 2 in IABP); pericardial effusion (8 PVAD, 8 IABP)	•Retrospective analysis<br>•No unadjusted analysis<br>•Limited data on patient characteristics and no details regarding the type of PVAD or the ablation approach<br>•Nonrandomized treatment allocation<br>•Nonstandardized treatment among centers<br><br>PVAD-supported catheter ablation yielded reduced in-hospital cardiogenic shock and renal failure, a shorter hospital LOS, and a higher rate of hospital discharges to home or self-care, but similar total hospitalization cost compared with IABP.

Study name or acronym; Author; Year published; PMID	Aim of study; Endpoints; Study type (RCT, observational — multicenter or single center; case series or other [if RCT include intervention and comparator]); Study size (N)	Patient population with inclusion and exclusion criteria	Results (absolute event rates, *P* values; OR or RR; 95% CI)	Other relevant findings or adverse events	Limitations; Other comments; Conclusions
Enriquez et al. Outcomes of rescue cardiopulmonary support for periprocedural acute hemodynamic decompensation in patients undergoing catheter ablation of electrical storm.<br>• Year published: 2018<br>• PMID: 28917560	Outcomes of emergent ECMO for rescue AHD in patients undergoing catheter ablation of ES. Acute (30 days) and long-term mortality after the procedure. Single center, retrospective<br>21 patients with rescue ECMO	Between January 2010 and June 2016, 21 patients (19 men; mean age 62.6 ± 11.3 years) with drug-refractory ES undergoing catheter ablation had periprocedural AHD requiring emergent ECMO support.	• 14 patients with AHD before catheter ablation<br>• 7 patients with AHD during the procedure<br>• In 11 patients hemodynamic stability was achieved<br>• In 4 patients ECMO was a bridge for LVAD<br>• 2 patients died before weaning<br>• 4 withdrew from care<br>• Ablation was performed in 18 patients (9 with VT and 9 with PVC-triggered VF); in 3 patients the procedure was aborted<br>• Overall, acute success was achieved in 15 of 18 cases (83%).<br>• PVC elimination was achieved in 8 of 9 patients; PVS showed no VT inducibility.<br>• After a median follow-up of 10 days (1 day–27 months), 16 (76%) patients died; 13 (81%) died during the index admission within 4.9 ± 4.6 days.<br>• Death was due to refractory VT/VF in 4 cases, HF in 11 cases.<br>• 7 patients survived 6 months postablation; 5 remained free of VT/VF, and 3 received destination therapy.<br>• Patients who died had chronic kidney disease (62.5% vs 0%, respectively).	Three patients had significant bleeding from the ECMO vascular access sites (axillary in 1 and femoral in 2) requiring transfusion (2–5 units of red blood cells). One patient presented thrombosis and occlusion of the ECMO cannula with compromise of oxygenation, and another patient developed an LV thrombus with embolism and transient ST-segment elevation.	• Single-center observational study<br>• The number of patients was relatively small<br>• A detailed assessment of the possible benefits of rescue ECMO support in different subsets of patients was not possible.<br>• Only the Rotaflow ECMO system was used in this study.<br><br>In patients with VT/VF ES undergoing catheter ablation, the outcomes of rescue ECMO support for periprocedural AHD are poor.

Study name or acronym; Author; Year published; PMID	Aim of study; Endpoints; Study type (RCT, observational — multicenter or single center; case series or other [if RCT include intervention and comparator]); Study size (N)	Patient population with inclusion and exclusion criteria	Results (absolute event rates, *P* values; OR or RR; 95% CI)	Other relevant findings or adverse events	Limitations; Other comments; Conclusions
Kusa et al. Outcomes of ventricular tachycardia ablation using percutaneous left ventricular assist devices.<br>• Year published: 2017<br>• PMID: 28576780	To compare the outcomes of patients undergoing scar-related VT ablation with and without PLVAD support.<br>Primary endpoint (death, heart transplant, and recurrent VT)<br>Single-center, retrospective<br>194 patients (109 PLVAD and 85 non-PLVAD).	Some 205 consecutive patients who underwent catheter ablation for hemodynamically unstable scar-related VT at the Mount Sinai Medical Center were included.	• DCM was more common in the PLVAD group (33% vs 13%; *P*=.001); ARVD was less common (2% vs 11%; *P*=.01). PVAD patients had significantly lower LVEF (26% ± 10% vs 39% ±16%; *P*<.001), higher prevalence of NYHA class ≥III (51% vs 25%; P<.001), and more frequent ES (49% vs 34%; *P*=.04).<br>• PLVAD was associated with longer procedures (422 ± 112 vs 330 ±92 minutes; *P*<.001).<br>• No difference in activation mapping or number of terminated VTs<br>• More overall VTs (3.3±2.1 vs 2.4±2.1; *P*=.004) and percentage of patients with at least 1 mappable VT (80% vs 68%; *P*=.06) in the PLVAD patients<br>• VT inducibility was greater in the PLVAD group (20% vs 7%; *P*=.02);<br>• PLVAD was associated with a longer postprocedure hospitalization (median 6 vs 4 days; *P*=.001);<br>• During a median follow-up of 215 days, no difference in the primary endpoint 36% (39 of 109) vs 26% (22 of 85) (*P*=.14).<br>• Death occurred in 9 (8%) and 5 (6%) patients (*P*=.53), heart transplant in 4 (4%) and 0 (0%) patients (*P*=.10), and recurrent VT in 35 (32%) PLVAD and 18 (21%) non-PLVAD patients (*P*=.09).	Procedure-related complications were similar between the 2 groups (17% vs 9%; *P*=.15): pericardial effusion (7% vs 4%; *P*=.26), vascular complications (7% vs 5%; *P*=.45), and worsening HF (3% vs 1%; *P*=.41). Acute kidney injury occurred in 24% of the PLVAD and 6% of non-PLVAD groups (*P*=.001). It resolved in all individuals except in 2 patients who died because of ES after ablation.	•Single-center, nonrandomized, retrospective analysis<br>•Only Impella PLVADs were used.<br><br>There were no advantages demonstrated with PLVAD use with respect to acute procedural outcomes; use of PLVAD HS with the Impella device was associated with better than expected outcomes.

Study name or acronym; Author; Year published; PMID	Aim of study; Endpoints; Study type (RCT, observational — multicenter or single center; case series or other [if RCT include intervention and comparator]); Study size (N)	Patient population with inclusion and exclusion criteria	Results (absolute event rates, *P* values; OR or RR; 95% CI)	Other relevant findings or adverse events	Limitations; Other comments; Conclusions
Mathuria et al. Outcomes of pre-emptive and rescue use of percutaneous left ventricular assist device in patients with structural heart disease undergoing catheter ablation of ventricular tachycardia.<br>• Year published: 2017<br>• PMID: 27497847	To assess the outcomes of preemptive and rescue use of PLVAD during VT ablation in patients with ICM and NICM. VT inducibility at the end of the procedure.<br><br>Freedom from VT at 3-month follow-up, 30-day mortality<br><br>Single center, retrospective study; 93 patients<br>Rescue PLVAD: 12<br>Preemptive PLVAD: 24<br>Non-PLVAD: 57	Between January 2009 and October 2011, 93 patients underwent VT ablation.	1. Comparison of rescue PLVAD and non-PLVAD<br>• More rescue PLVAD patients were on multiple AADs; had a higher risk score (17.8 vs 13.4, *P*=.01); longer procedure times (226 ± 88 vs 182 ± 77 minutes, *P*=.01); an increased 30-day all-cause mortality (7 of 12 or 58.3%) (2 of 57 or 3.5%; *P*=.001); and a higher % of MAP <50 mm Hg with RV pacing (16 of 57 vs 9 of 12; *P*=.006).<br>• The non-PLVAD group spent less time in VT (9.3 ± 8.5 vs 23.0 ± 28.9 minutes, *P*=.03).<br>• There was no difference in inducibility of VT (4 of 8 in the rescue group vs 29 of 44 in the non-PLVAD group; *P*=.43) and in freedom from VT at 3-month follow-up (31 of 55 [56%] patients in the non-PLVAD group and 3 of 5 [60%] in the rescue group; *P*=.34).<br>2. Comparison of preemptive and rescue PLVAD<br>• No difference in the PAAINESD score, in procedure times, or time in VT; in hemodynamic parameters and inducibility of VT in freedom from VT at 3-month follow-up (17 of 23 vs 31 of 55; *P*=.15), and in 30-day mortality (1 of 24 vs 2 of 57; *P*=.89).<br>• More VTs terminated during ablation in the preemptive PLVAD; an increased 30-day all-cause mortality in the rescue PLVAD group (58.3%) compared with the preemptive PLVAD group (4.2%; *P*=.003).<br>3. Comparison of non-PLVAD and preemptive PLVAD<br>No differences, but a higher PAAINESD score (16.5 ± 5.4 vs 13.4 ± 5.4; *P*=.02) and more patients with a drop in MAP with RV pacing (16 of 57 vs 13 of 24; *P*=.03) in the preemptive group.	Rescue PLVAD<br>• 9 of 12 patients had cardiogenic shock<br>• 2 patients had cardiac arrest during the procedure (1 pulseless electrical activity, 1 refractory VF).<br>• 1 pericardial effusion treated with pericardiocentesis, and PLVAD insertion due to refractory hypotension.<br>• The 30-day all-cause mortality was 58.3% (5 of 7 refractory HFs postprocedure despite the use of PLVAD, 1 intracerebral hemorrhage, and 1 from sepsis).<br>Preemptive PLVAD<br>• 1 pulseless electrical activity during the procedure, which was upgraded to ECMO support (survived); 1 died within 30 days due to refractory HF 20 days after ablation.	• No randomization of the groups<br>• No comparison of outcomes of patients with hemodynamic collapse who did not undergo PLVAD<br>• No end-organ perfusion data<br>• No RV function in outcome assessment<br><br>The role of rescue PLVAD insertion for acute hemodynamic collapse during VT ablation in patients with cardiomyopathy is limited, given it continues to be associated with a high 30-day mortality. Preemptive PLVAD insertion might benefit certain high-risk patients.

Study name or acronym; Author; Year published; PMID	Aim of study; Endpoints; Study type (RCT, observational — multicenter or single center; case series or other [if RCT include intervention and comparator]); Study size (N)	Patient population with inclusion and exclusion criteria	Results (absolute event rates, *P* values; OR or RR; 95% CI)	Other relevant findings or adverse events	Limitations; Other comments; Conclusions
Muser et al. Outcomes with prophylactic use of percutaneous left ventricular assist devices in high-risk patients undergoing catheter ablation of scar-related ventricular tachycardia: a propensity-score matched analysis.<br>• Year published: 2018<br>• PMID: 29753944	To evaluate the outcomes of prophylactic use of PLVAD in high-risk patients undergoing CA of scar-related VT.<br><br>Occurrence of periprocedural AHD.<br><br>Occurrence of any VT after the index procedure.<br><br>Occurrence of the composite end point of death or cardiac transplantation.<br><br>Single-center, retrospective case-control study of 75 high-risk patients undergoing VT catheter ablation; VT with prophylactic PLVAD.<br><br>Control:<br>75 propensity-matched	Selected among 674 consecutive patients screened with SVT and SHD who were referred to the Hospital of the University of Pennsylvania for VT ablation from January 2009 to December 2015.	• Periprocedural AHD occurred in 22 (15%) patients: 5 (7%) in the propensity PLVAD group vs 17 (23%) (*P*=.01)<br>• VT noninducibility was more frequent in the PLVAD group (81% vs 62%; *P*=.02).<br>• No difference in noninducibility at NIPS (79% of patients in the PLVAD group vs 75% of patients in the control group (*P*=.75).<br>• 3 patients had a persistent low output state (2 died, 1 had emergency cardiac surgery due to cardiac vein perforation).<br>• Median follow-up of 9 months (25th–75th percentile, 15–27 months), 30 patients died or underwent heart transplant (40%) in the propensity PLVAD group vs 45 (60%) (*P*=.01).<br>• No difference in death (60 [40%] patients died during follow-up: 25 [33%] in the propensity PLVAD vs 35 [47%]; *P*=.09), nor in heart transplantation: 15 (10%) patients; 5 (7%) in the propensity PLVAD group vs 10 (13%) (*P*=.17).<br>• Cumulative incidence of death/transplant was lower in the propensity PLVAD group (18% and 33% at 30-day and 12-month follow-up, respectively) vs (30% and 66% at 30-day and 12-month follow-up, respectively) (*P*<.01).<br>• Substantial mortality benefit with propensity PLVAD in high-risk patients (PAAINESD score >15 HR 0.43; 95% CI 0.21–0.87; *P*=.02).<br>• No significant difference of VT recurrence in the propensity PLVAD group (17% and 40% at 30-day and	Prophylactic PLVAD group<br>• 1 pericardial tamponade requiring open chest surgery with subsequent left lower limb ischemia and surgical PLVAD removal and repair of the femoral artery<br>• 3 pericardial effusions, successfully drained percutaneously<br>• 1 subtotal occlusion of the femoral artery requiring surgical endarterectomy, which was complicated by retroperitoneal hematoma.<br>• 1 fracture of the distal end of the device occurred at the time of percutaneous removal and required surgical retrieval.<br>• 1 embolic stroke	• Lack of randomization<br>• The use of a PLVAD was discretional, and it was occasionally implanted in patients at low to moderate risk.<br>• The Impella ventricular assist device was the only device used<br><br>In high-risk patients with SHD undergoing catheter ablation of scar-related VT, prophylactic implantation of a PLVAD is associated with a significant reduction of the risk of AHD and postprocedural mortality or need for heart transplant.

Study name or acronym; Author; Year published; PMID	Aim of study; Endpoints; Study type (RCT, observational — multicenter or single center; case series or other [if RCT include intervention and comparator]); Study size (N)	Patient population with inclusion and exclusion criteria	Results (absolute event rates, *P* values; OR or RR; 95% CI)	Other relevant findings or adverse events	Limitations; Other comments; Conclusions
	patients treated without prophylactic PLVAD		12-month follow-up, respectively) vs control group (20% and 41% at 30-day and 12-month follow-up, respectively) (*P*=.97).<br>• In multivariable analysis, baseline LVEF (HR 0.97; 95% CI 0.95–0.99; *P*=.03), CKD (HR 2.24; 95% CI 1.35–3.72; *P*<.01), VT recurrence (HR 2.33; 95% CI 1.31–4.14; *P*<.01), and propensity PLVAD (HR 0.28; 95% CI 0.16–0.49; *P*<.01) all correlated with death/transplant.<br>• LVEF (HR 0.97; 95% CI 0.95–0.99; *P*=.02) and presentation with VT storm (HR 4.39; 95% CI 1.26–15.38; *P*=.02) were the only independent predictors of VT recurrence at follow-up.	• 1 deep venous thrombosis<br><br>Control group<br>• 1 phrenic nerve injury during epicardial ablation, along with transient emidiaphragmatic paralysis<br>• 4 pericardial effusions, successfully drained percutaneously	

Study name or acronym; Author; Year published; PMID	Aim of study; Endpoints; Study type (RCT, observational — multicenter or single center; case series or other [if RCT include intervention and comparator]); Study size (N)	Patient population with inclusion and exclusion criteria	Results (absolute event rates, *P* values; OR or RR; 95% CI)	Other relevant findings or adverse events	Limitations; Other comments; Conclusions
Turagam et al. Hemodynamic support in ventricular tachycardia ablation: an international VT ablation center collaborative group study.<br>• Year published: 2017<br>• PMID: 29759835	To evaluate the clinical outcomes of patients receiving HS during VT ablation.<br><br>To examine clinical predictors of long-term outcomes of patients undergoing VT ablation receiving HS and its impact on mortality and VT recurrence compared with those not receiving HS.<br><br>Retrospective, multicenter from the International VT Ablation Center Collaborative (IVTCC) group database.<br><br>105 patients receiving HS (TandemHeart, Impella 2.5, or ECMO)	VTCA supported procedures from the shared database of the IVTCC group.	Acute procedural success (71.8% vs 73.7%; *P*=.04) was significantly lower and complications (12.5% vs 6.5%; *P*=.03) and 1-year mortality (34.7% vs 9.3%; *P*<.001) were significantly higher in the HS group.<br><br>HS has been shown to be an independent predictor of mortality (HR 5.01; 95% CI 3.44–7.20; *P*<.001).<br><br>No significant difference in VT recurrence between groups at 12 months of follow-up (29.6% vs 25.4%; *P*=.4).<br><br>In a subgroup analysis including LVEF ≤20% and NYHA functional class III to IV patients, acute procedural success (74.0% vs 70.5%; *P*=.8), complications (15.6% vs 7.8%; *P*=.2), VT recurrence (30.2% vs 38.1%; *P*=.44), and 1-year mortality (40.0% vs 28.8%; *P*=.2) were no different between the HS and no-HS groups.	Included pericardial tamponade or effusion requiring drainage, and vascular complications such as access site hematoma, arteriovenous fistula requiring intervention, systemic thromboembolism, and in-hospital mortality.<br><br>HS group: 13 events (12.5%)<br><br>No-HS group: 98 (6.5%)	• Retrospective nonrandomized cohort study<br>• Type of HS at discretion of the operator<br>• No data about HS strategy (preemptive vs rescue)<br>• Data only from high-volume experienced centers performing VT ablation<br>• No data about the extent of HS provided<br>• Variable complication rate according to HS type.<br><br>Indications for and risks of HS for VT catheter ablation in patients at high-risk for mortality should be carefully weighed.

Anticoagulation

Study name or acronym; Author; Year published; PMID	Aim of study; Endpoints; Study type (RCT, observational — multicenter or single center; case series or other [if RCT include intervention and comparator]); Study size (N)	Patient population with inclusion and exclusion criteria	Results (absolute event rates, *P* values; OR or RR; 95% CI)	Other relevant findings or adverse events	Limitations; Other comments; Conclusions
Kuck et al. Catheter ablation of stable ventricular tachycardia before defibrillator implantation in patients with coronary heart disease (VTACH): a multicentre randomised controlled trial.<br>• Year published: 2010<br>• PMID: 20109864	Aim: To assess the potential benefit of catheter ablation before implantation of an ICD.<br><br>Endpoints:<br>- The primary endpoint was the time to first recurrence of VT or VF.<br>- Secondary endpoints were survival free from severe clinical events (death, syncope, hospital admission for a cardiac reason, and VT storm, defined as more than 3 VT episodes in 24 hours), number of appropriate ICD interventions (ATP or shock), and quality of life.<br><br>Study type: prospective, open, randomized controlled trial<br><br>Study size (N): 110 patients	Inclusion criteria: Patients aged 18–80 years were eligible for enrollment if they had stable VT, previous MI, and reduced LVEF (≤50%).<br><br>Patients were excluded if they had one or more of the following: An acute MI within the preceding month, cardiac surgery within the preceding 2 months, a protruding LV thrombus on echocardiogram before ablation, valvular heart disease or a mechanical heart valve that precluded LV access, unstable angina, incessant VT, BBRT, contraindication to heparin treatment, impaired renal function (serum creatinine >220 µmol/L), HF class IV, or other medical conditions likely to limit survival to less than 12 months.	Mean age 66 years<br><br>Male sex, 100 (93%)<br><br>LVEF (%) 34<br><br>Mean follow-up was 22.5 months (SD 9.0)	Time to recurrence of VT or VF was longer in the ablation group (median 18.6 months [lower quartile 2.4, upper quartile not determinable]) than in the control group (5.9 months [IQR 0.8–26.7]).<br><br>At 2 years, estimates for survival free from VT or VF were 47% in the ablation group and 29% in the control group (HR 0.61; 95% CI 0.37–0.99; *P*=.045).<br><br>Complications related to the ablation procedure occurred in 2 patients; no deaths occurred within 30 days after<br>ablation. Some 15 device-related complications requiring surgical intervention occurred in 13 patients (ablation group, 4; control group, 9). Nine patients died during the study (ablation group, 5; control group, 4).	Limitations:<br>- Small number of patients in centers with experienced clinicians.<br>- Whether the additional costs of catheter ablation would be compensated by the reduced number of hospital admissions in patients with ICDs is unclear.<br>- Furthermore, patients underwent catheter ablation before any ICD intervention. Therefore, the question of the best possible time of catheter ablation in patients with an indication for an ICD cannot be answered.<br>- The quality-of-life analysis was limited by the fairly small number of patients with assessed data.<br>- This study did not compare ablation with treatment with AADs, and therefore we cannot comment on the relative efficacy of these two therapeutic strategies in this setting.<br>Other comments and conclusions:<br>- Prophylactic VT ablation before defibrillator implantation seemed to prolong time to recurrence of VT in patients with stable VT, previous MI, and reduced LVEF.<br>- Prophylactic catheter ablation should therefore be considered before implantation of an ICD in such patients.

Study name or acronym; Author; Year published; PMID	Aim of study; Endpoints; Study type (RCT, observational — multicenter or single center; case series or other [if RCT include intervention and comparator]); Study size (N)	Patient population with inclusion and exclusion criteria	Results (absolute event rates, *P* values; OR or RR; 95% CI)	Other relevant findings or adverse events	Limitations; Other comments; Conclusions
Kuck et al. Impact of substrate modification by catheter ablation on implantable cardioverter-defibrillator interventions in patients with unstable ventricular arrhythmias and coronary artery disease: results from the multicenter randomized controlled SMS (substrate modification study).<br>• Year published: 2017<br>• PMID: 28292751	Aim: To assess whether prophylactic ablation of the arrhythmogenic substrate reduces or prevents the recurrence of VT/VF in such patients.<br><br>Endpoints:<br>- The primary study endpoint was the time to first recurrence of VT/VF.<br>- Secondary endpoints were appropriate ICD therapies, quality of life according to the Medical Outcome Study Short Form-36 score, number of hospital readmissions because of a cardiac indication, and severe clinical events (death, number of syncopes, and number of ES episodes, defined as >3 VT episodes within 24 hours).<br><br>Study type: Randomized clinical trial<br><br>Study size (N): 111 patients	Inclusion criteria: Patients between 18 and 80 years of age with CAD, LVEF ≤40%, and clinically unstable spontaneous VT, or cardiac arrest or syncope with unstable VT inducible at the baseline EPS.<br><br>Patients were excluded if they had an LV thrombus, NYHA functional class IV, an acute MI within the preceding 2 months, valvular heart disease or a mechanical heart valve, unstable angina, cardiac surgery within the past 2 months, serum creatinine >220 mEq/L (>2.5 mg/dL), thrombocytopenia or coagulopathy, a contraindication to heparin, pregnancy, or participation in another investigational study.	Age 67.1 ± 8.1 years<br><br>Male sex, 93 (84%)	Patients were followed up for 2.3 ± 1.1 years. The primary endpoint was reached by 25 ablation patients and 26 ICD-only patients.<br><br>Two-year event-free survival was estimated at 49.0% (95% CI 33.3%–62.9%) in the former and 52.4% (36.7%–65.9%) in the latter groups. Comparison of episode incidence revealed no significant difference in the primary endpoint (*P*=.84).<br><br>In an Andersen–Gill regression model with multiple end point recurrences, the difference between the study arms significantly favored catheter ablation for both the primary endpoint and all but one of the predefined subgroups of detected arrhythmia events.	Limitations:<br>- Small number of patients randomized.<br>- Patients had multiple types of VA, with the common denominator of hemodynamic instability.<br>- The ablation procedure was not prespecified in detail, which would have led to a more homogeneous approach among individual investigators and participating centers.<br>- Proper randomization was affected by 6 (10%) patients from the ablation arm excluded from the analysis because of missing data.<br>Other comments and conclusions:<br>- Of 111 patients included in an intention-to-treat analysis, 54 were randomly assigned catheter ablation plus ICD implantation (ablation group: 68 ±8 years; 47 men), whereas 57 were assigned ICD implantation without catheter ablation (ICD-only group: 66 ± 8 years; 46 men).<br>- SMS is the third randomized trial after SMASH-VT and VTACH to investigate the impact of prophylactic catheter ablation followed by ICD implantation on VT recurrences in patients with sustained VAs. However, it is the first of these 3 trials that did not show a benefit of prophylactic catheter ablation with respect to the primary endpoint of time to recurrence of any VT.

Study name or acronym; Author; Year published; PMID	Aim of study; Endpoints; Study type (RCT, observational — multicenter or single center; case series or other [if RCT include intervention and comparator]); Study size (N)	Patient population with inclusion and exclusion criteria	Results (absolute event rates, *P* values; OR or RR; 95% CI)	Other relevant findings or adverse events	Limitations; Other comments; Conclusions
Sapp et al. Ventricular tachycardia ablation versus escalation of antiarrhythmic drugs.<br>• Year published: 2016<br>• PMID: 27149033	Aim: To compare catheter ablation with escalated AAD therapy in patients with ICM and an ICD who had VT despite first-line AAD therapy.<br><br>Endpoints:<br>- Primary endpoint: composite of death and appropriate ICD shock.<br>Secondary Endpoints: all-cause mortality; appropriate ICD ATP; appropriate ICD shocks; documented sustained; VT below detection rate of the ICD; number of ICD shocks; hospital admission for cardiac causes; procedural complications, amiodarone toxicity or adverse events; effects on EF; quality of life/anxiety; cost-effectiveness.<br><br>Study type: Randomized clinical trial<br>Study size (N): 259 patients	Patients were eligible for inclusion if they had had an MI, had undergone placement of an ICD, and had had an episode of VT during treatment with amiodarone or another class I or class III AAD within the previous 6 months.	Of the 259 patients who were enrolled, 132 were assigned to the ablation group and 127 to the escalated-therapy group.<br><br>Mean follow-up (±SD) 27.9 ± 17.1 months	The primary outcome occurred in 59.1% of patients in the ablation group and 68.5% of those in the escalated-therapy group (HR in the ablation group, 0.72; 95% CI 0.53–0.98; *P*=.04).<br><br>There was no significant between-group difference in mortality. There were 2 cardiac perforations and 3 cases of major bleeding in the ablation group, and 2 deaths from pulmonary toxic effects and 1 from hepatic dysfunction in the escalated-therapy group.	Limitations:<br>- It was not powered to assess the effect of the two treatments on mortality.<br>- Although the practitioners who performed catheter ablation in our trial were experienced in the procedure, it is possible that specialized referral centers for ablation of VT could have achieved better procedural outcomes.<br>- Patients had a high disease burden relatively late in the course of advanced cardiac disease and evaluated second-line therapy for VT. Thus, further study is required to show whether catheter ablation or AAD therapy is the most effective first-line therapy for scar-related VT.<br>Other comments and conclusions:<br>- Intravenous heparin will be administered to maintain an activated clotting time >300 seconds (changed to >250 seconds)<br>- At the end of the procedure, intravenous heparin will be administered (without bolus) 6 hours after sheath removal and oral anticoagulation with warfarin will be started if a substrate-mapping approach was used, or if extensive ablation was performed (≥10 minutes of RF time).

Study name or acronym; Author; Year published; PMID	Aim of study; Endpoints; Study type (RCT, observational — multicenter or single center; case series or other [if RCT include intervention and comparator]); Study size (N)	Patient population with inclusion and exclusion criteria	Results (absolute event rates, *P* values; OR or RR; 95% CI)	Other relevant findings or adverse events	Limitations; Other comments; Conclusions
Di Biase et al. Ablation of stable VTs versus substrate ablation in ischemic cardiomyopathy: the VISTA randomized multicenter trial.<br>• Year published: 2015<br>• PMID: 26718674	Aim: To determine rates of VT recurrence in patients undergoing ablation limited to clinical VT along with mappable VTs ("clinical ablation") versus substrate-based ablation.<br><br>Endpoints:<br>- Primary endpoints: Recurrence of any VT during the 12-month postablation period, as demonstrated by device interrogation and clinical evaluation.<br>- Secondary endpoints: Periprocedural complications, 12-month postprocedure mortality, rehospitalization, and combined incidence of rehospitalization and mortality.<br><br>Study type: Randomized clinical trial<br><br>Study size (N): 118 patients	Inclusion criteria: patients with ICM who had received an ICD before the ablation and had recurrent stable MMVTs that were symptomatic or required ICD therapies despite AADs. Between April 2009 and July 2013, patients from 7 centers were randomized to substrate-based ablation (n=58) or to ablation of clinical and mappable VTs only (n=60).<br><br>Exclusion criteria: VT that presented with syncope, loss of consciousness, or cardiac arrest; age <18 years; severe renal (glomerular filtration rate 15 to 29 ml/minute/1.73 m^2 for ≥3 months), LV thrombus, unstable angina, severe aortic stenosis, end-stage HF with limited life expectancy, and prior failed VT ablation.	Baseline clinical characteristics and electrophysiologic features were not significantly different between the substrate ablation and clinical ablation groups; mean LVEF was 32.0% ± 9.9% and 32.6% ± 14.1% (*P*=.8), and patients failed an average of 1.4 ± 0.7 and 1.5 ± 0.7 AADs, respectively (*P*=.86). After ablation, both groups underwent the same preablation induction protocol. In the clinical ablation group, the average number of postablation unstable nonclinical VTs induced was lower than that observed at the beginning (2.0 ± 1.4 vs 2.7 ± 1.2; *P*=.011).<br><br>Age (5-year	At 12-month follow-up, 9 (15.5%) and 29 (48.3%) patients had VT recurrence in substrate-based and clinical VT ablation groups, respectively (log-rank *P*<.001).<br>More patients undergoing clinical VT ablation (58%) were on AADs after ablation vs substrate-based ablation (12%; *P*<.001).<br>Seven (12%) patients with substrate ablation and 19 (32%) with clinical ablation required rehospitalization (*P*=.014).<br>Overall 12-month mortality was 11.9%; 8.6% in substrate ablation and 15.0% in clinical ablation groups, respectively (log-rank *P*=.21). Combined incidence of rehospitalization and mortality was significantly lower with substrate ablation (*P*=.003).<br>Periprocedural complications were similar in both groups (*P*=.61).<br>After adjusting for	Limitations:<br>- Complete elimination of abnormal potentials within the scar tissue in the substrate ablation group was not possible in 16% of the population.<br>- Determination of whether an induced VT is "clinical" is sometimes difficult because similarity in CLs and intracardiac ICD morphologies are imperfect predictors.<br><br>Other comments and conclusions:<br>- Combined reduction of rehospitalization and mortality was observed in the substrate ablation group.<br>- This is the first randomized multicenter study showing that substrate-based ablation reduces recurrence of any VT at follow-up when compared with ablation limited to clinical and mappable VTs in patients with ICM, suggesting that a larger area of scar tissue must be ablated to reduce recurrence from any VTs in patients with ICM and stable clinical VTs.<br>- More patients were able to discontinue AADs after substrate ablation, an important finding because AADs can have significant long-term adverse effects.

Study name or acronym; Author; Year published; PMID	Aim of study; Endpoints; Study type (RCT, observational — multicenter or single center; case series or other [if RCT include intervention and comparator]); Study size (N)	Patient population with inclusion and exclusion criteria	Results (absolute event rates, *P* values; OR or RR; 95% CI)	Other relevant findings or adverse events	Limitations; Other comments; Conclusions
			increments), male sex, ES, LVEF, diabetes, and substrate-based ablation showed significant unadjusted association with VT recurrence.	covariates in a Cox multivariate model, substrate-based ablation was associated with 67% lower risk of recurrence compared with clinical ablation (HR 0.33; 95% CI 0.13–0.81; *P*=.014).	- Systemic anticoagulation was achieved with intravenous heparin targeted to a minimum activation clotting time of 300 seconds.

Study name or acronym; Author; Year published; PMID	Aim of study; Endpoints; Study type (RCT, observational — multicenter or single center; case series or other [if RCT include intervention and comparator]); Study size (N)	Patient population with inclusion and exclusion criteria	Results (absolute event rates, *P* values; OR or RR; 95% CI)	Other relevant findings or adverse events	Limitations; Other comments; Conclusions
Reddy et al. Prophylactic catheter ablation for the prevention of defibrillator therapy.<br>• Year published: 2007<br>• PMID: 18160685	Aim: To examine whether prophylactic RFCA of arrhythmogenic ventricular tissue would reduce the incidence of ICD therapy<br><br>Over the course of 46 months, 128 patients were enrolled and randomly assigned to the ablation group or the control group (64 in each group)<br><br>Endpoints:<br>- Primary endpoint: Survival free from any appropriate ICD therapy (shock or ATP)<br>- Secondary endpoint: Freedom from any appropriate ICD shock, death, or ICD storm<br><br>Study type: Prospective, unblinded, randomized, controlled multicenter trial<br><br>Study size (N): 128 patients	Inclusion criteria: Men and women who were at least 18 years of age with a history of MI more than 1 month before enrollment and if they had undergone a planned or a recent (within 6 months) implantation of a defibrillator for VF, hemodynamically unstable VT, or syncope with inducible VT during invasive electrophysiological testing.<br><br>Exclusion criteria: Patients being treated with a class I or class III AAD, if the substrate for the VA was thought not to be due to the MI, if active and ongoing cardiac ischemia was thought to be the cause of the VA, or if they were having incessant or multiple episodes of VT necessitating immediate treatment (with drugs or ablation). Additional exclusion criteria included	Age 67 years<br><br>Male sex, 87%<br><br>The qualifying index arrhythmia was VF in 18% of patients, VT in 49%, syncope with inducible VT in 21%, and recent VF or VT treated by a previously implanted ICD in 12%.<br><br>LVEF was 30.7% ± 9.5% in the ablation group and 32.9% ± 8.5% in the control group (*P*=.16).<br><br>LVEF was <20% in 16 patients in the ablation group and in 7 patients in the control group (*P*=.06).<br><br>The two groups were well balanced with respect to baseline characteristics, and a high percentage	The mortality rate 30 days after ablation was 0%, and there were no significant changes in ventricular function or functional class during the mean (±SD) follow-up period of 22.5 ± 5.5 months.<br><br>Some 21 patients assigned to defibrillator implantation alone (33%) and 8 patients assigned to defibrillator implantation plus ablation (12%) received appropriate ICD therapy (ATP or shocks) (HR in the ablation group, 0.35; 95% CI 0.15–0.78; *P*=.007). Among these patients, 20 in the control group (31%) and 6 in the ablation group (9%) received shocks (*P*=.003). Multivariate analysis adjusted for patient characteristics and medication use at baseline did not reduce the magnitude of the effect observed (HR 0.31; 95% CI 0.13–0.76; *P*=.01).	Limitations:<br>- Small number of participants<br>- Absence of quality of life information<br>- Long recruitment period (4 years)<br>- No standardized ATP algorithm<br>- Selection of only high-volume, highly experienced ablation centers.<br><br>Other comments and conclusions:<br>- Ablation was performed with the use of a substrate-based approach in which the myocardial scar is mapped and ablated while the heart remains predominantly in SR.<br>- During follow-up (22.5 ± 5.5 months; range, 0–26), no patient received an AAD (other than BBs) before the primary endpoint was reached.<br>- There was no significant change in ventricular function or functional status during follow-up. This remained true even for those patients with the most depressed cardiac function. Overall mortality was not greater in the patients assigned to ablation; indeed, there was even a trend toward decreased mortality in the ablation group, although it was not statistically significant.

Study name or acronym; Author; Year published; PMID	Aim of study; Endpoints; Study type (RCT, observational — multicenter or single center; case series or other [if RCT include intervention and comparator]); Study size (N)	Patient population with inclusion and exclusion criteria	Results (absolute event rates, *P* values; OR or RR; 95% CI)	Other relevant findings or adverse events	Limitations; Other comments; Conclusions
		the inability to give informed consent, stroke within 30 days before screening, contraindication to anticoagulation therapy, or any medical or nonmedical condition likely to prevent completion of the trial.	received BBs, antagonists of the renin–angiotensin–aldosterone system, and statin drugs.	Compared with the control population, patients in the ablation group had a 65% reduction in the risk of receiving ICD therapy during the subsequent 2 years. When ATP therapy was excluded from this analysis, there remained a 73% reduction in the risk of receiving subsequent ICD shocks.<br><br>There was a trend toward decreased mortality in the ablation group compared with the control group (9% vs 17%, *P*=.29). ICD storms occurred in 4 patients assigned to ablation (6%) and 12 control patients (19%) (*P*=.06).<br><br>Mortality was not increased in the ablation group compared with the control group (9% vs 17%; *P*=.29).	- In 3 (5%) patients, no appreciable endocardial scar was visualized; therefore, no ablation lesions were placed.<br>- Intravenous heparin was administered to achieve an activated clotting time >220 seconds.

Study name or acronym; Author; Year published; PMID	Aim of study; Endpoints; Study type (RCT, observational — multicenter or single center; case series or other [if RCT include intervention and comparator]); Study size (N)	Patient population with inclusion and exclusion criteria	Results (absolute event rates, *P* values; OR or RR; 95% CI)	Other relevant findings or adverse events	Limitations; Other comments; Conclusions
Calkins et al. Catheter ablation of ventricular tachycardia in patients with structural heart disease using cooled radiofrequency energy: results of a prospective multicenter study. Cooled RF multicenter investigators group.<br>• Year published: 2000<br>• PMID: 10841242	Aim: To evaluate the safety and efficacy of an RFCA system with internal saline irrigation.<br><br>Endpoints:<br>- Acute success: absence of inducible, mappable VT at the end of the ablation procedure.<br>- Long-term: absence of any spontaneous sustained VT during follow-up.<br>- Safety: procedure-related adverse event.<br><br>Study type: Prospective multicenter<br><br>Study size (N): 146 patients	Patients were initially recruited for the study if they fulfilled the following criteria: (1) documented SMVT with 2 or more episodes in the 2 months before enrollment; (2) spontaneous VT that was hemodynamically stable; (3) VT due to IHD; (4) an ICD device with electrogram storage; and (5) failure of at least two AADs. At the onset of the study, patients were randomized 1:1 between ablation and continued antiarrhythmic therapy. As the study progressed, the enrollment criteria were altered to facilitate enrollment, including elimination of randomization and the requirement for an ICD.	Mean age 65 years<br><br>LVEF (%) 31 ± 13<br><br>IC 82%<br><br>The duration of follow-up was 243 ± 153 days.	Catheter ablation was acutely successful, as defined by elimination of all mappable VTs, in 106 (75%) patients. In 59 (41%) patients, no VT of any type was inducible after ablation. Twelve (8%) patients experienced a major complication.	Limitations:<br>- No randomization<br>- The number of patients enrolled at participating institutions ranged from 3 to 32. Therefore, it is possible that the relatively low efficacy and high incidence of complications could in part be attributable to an unequal distribution of mapping/ablation skills at participating centers.<br>- The type of structural heart disease present in the study patients was classified by the investigator as to whether they did or did not have IHD. The types of NICMs were not broken down further.<br><br>Other comments and conclusions:<br>- Before the ablation catheter was positioned in the left ventricle, heparin was administered to achieve an activated clotting time >250 seconds.<br>- Four of the major complications led to a patient death (2.7%). The most significant complications were 4 strokes or TIAs, 4 episodes of pericardial tamponade, inadvertent complete heart block in 2 patients, and 1 MI and aortic valve injury in 1 patient.

Study name or acronym; Author; Year published; PMID	Aim of study; Endpoints; Study type (RCT, observational — multicenter or single center; case series or other [if RCT include intervention and comparator]); Study size (N)	Patient population with inclusion and exclusion criteria	Results (absolute event rates, *P* values; OR or RR; 95% CI)	Other relevant findings or adverse events	Limitations; Other comments; Conclusions
Whitman et al. Brain emboli after left ventricular endocardial ablation.<br>• Year published: 2017<br>• PMID: 28119381	Aim: To determine whether cerebral emboli identified by comparing pre- and postprocedural DWI-MRI of the brain occur after LV endocardial ablation.<br><br>Study type: Cohort<br><br>Study size (N): 18 patients	Inclusion criteria: All consenting consecutive patients scheduled for PVC or VT ablations.<br><br>Exclusion criteria: Age <18 years, contraindication to MRI, presence of ICD or permanent pacemaker, or inability to provide informed consent.	Mean age 58 years.<br><br>No statistically significant relationships between ACT measurements and brain emboli; the only relationship that neared statistical significance (*P*=.05) revealed a trend toward more subtherapeutic time in those without the development of emboli.	LV ablation was performed in 12 patients (VT, n=2; PVC, n=10) and exclusively RV ablation in 6 patients (VT, n=1; PVC, n=5).<br><br>Seven (58%) patients undergoing LV ablation experienced a total of 16 cerebral emboli, compared with 0 patients undergoing RV ablation (*P*=.04). Seven of 11 (63%) patients undergoing a retrograde approach to the LV developed at least one new brain lesion.	Limitations:<br>- Relatively small sample size<br>- Patients in the study did not routinely undergo transesophageal echocardiography or computerized axial tomography to assess for aortic atherosclerosis.<br><br>Other comments and conclusions:<br>- RF energy was used for ablation in all cases and heparin was administered with goal-activated clotting times of 300–400 seconds for all LV procedures.<br>- More than half of patients undergoing routine LV ablation procedures (predominately PVC ablations) experienced new brain emboli after the procedure.<br>- None of the patients was symptomatic and none of the pre or post physical examinations revealed any new neurologic deficits.

Study name or acronym; Author; Year published; PMID	Aim of study; Endpoints; Study type (RCT, observational — multicenter or single center; case series or other [if RCT include intervention and comparator]); Study size (N)	Patient population with inclusion and exclusion criteria	Results (absolute event rates, *P* values; OR or RR; 95% CI)	Other relevant findings or adverse events	Limitations; Other comments; Conclusions
Sawhney et al. Epicardial catheter ablation for ventricular tachycardia on uninterrupted warfarin: a safe approach for those with a strong indication for peri-procedural anticoagulation?<br>• Year published: 2016<br>• PMID: 27454616	Aim: To investigate the safety of obtaining epicardial access on uninterrupted warfarin.<br><br>Endpoints:<br>- The primary endpoint was a composite of major and minor procedural complications.<br>- Secondary endpoints included acute procedural success and freedom from arrhythmia at the 3-month follow-up.<br>- Acute ablation success was defined as an identifiable epicardial target, and noninducibility was not sought in all patients.<br><br>Study type: Retrospective, case-control<br><br>Study size (N): 46 patients	Consecutive patients in whom epicardial access was attempted.	Some 46 patients were included, of which 13 were taking warfarin.<br><br>There was no significant difference in clinical and procedural characteristics (except INR and AF) between the 2 groups. Epicardial access was achieved in all patients.	There were no deaths, and no patients required surgery.<br><br>A higher proportion of patients in the warfarin group had a drop-in hemoglobin of >2 g/dL compared with the no-warfarin group (38.5% vs 27.3%, *P*=.74) and delayed pericardial drain removal (7.8% vs 3.03%, *P*=.47).<br><br>There was no difference in the overall procedural complication rate. No patients required warfarin reversal or blood transfusion.	Limitations:<br>- Small cohort of patients in a single center.<br>- The 2 groups were approximately matched, albeit with a trend toward greater age and comorbidity in the anticoagulated group.<br>- All oral anticoagulation was performed with warfarin; the safety of newer anticoagulants in this setting remains unknown.<br><br>Other comments and conclusions:<br>- All patients were heparinized prior to epicardial access with a target ACT of 300–350 seconds. Patients who had procedures performed on uninterrupted warfarin (in addition to heparin) were compared with those not taking an oral anticoagulant.<br>- No patient underwent epicardial ablation on a NOAC during the study period.

Study name or acronym; Author; Year published; PMID	Aim of study; Endpoints; Study type (RCT, observational — multicenter or single center; case series or other [if RCT include intervention and comparator]); Study size (N)	Patient population with inclusion and exclusion criteria	Results (absolute event rates, *P* values; OR or RR; 95% CI)	Other relevant findings or adverse events	Limitations; Other comments; Conclusions
Tanner et al. Catheter ablation of recurrent scar-related ventricular tachycardia using electroanatomical mapping and irrigated ablation technology: results of the prospective multicenter Euro-VT-study.<br>• Year published: 2010<br>• PMID: 19656251	Aim: To assess the efficacy and safety of EAM in combination with open saline irrigated ablation technology for ablation of chronic recurrent mappable and unmappable VT in remote MI.<br><br>Endpoints:<br>- The primary endpoint of the study was procedural effectiveness of catheter ablation.<br>- Acute success was defined as termination and noninducibility of all clinically relevant VTs on hospital discharge.<br>- Chronic success was defined as no recurrence of VT at 6 and 12 months of follow-up. Recurrences were documented by ICD telemetry for those patients or by conventional surface ECG recordings in the others.<br>- The secondary endpoint of the study was procedural safety as	Patients with recurrent drug and/or device refractory SVT due to prior MI were consecutively included from November 1999 to January 2003. They had to present with 4 or more episodes of symptomatic VT occurring within the preceding 6 months or with incessant VT refractory to medication and cardioversion. For patients without an ICD, 2 documented spontaneous SVT episodes (>1 minute) had to have occurred in the previous 2 months.<br><br>The main exclusion criteria were (1) age <18 years; (2) definite protruding LV thrombus on preablation echocardiography; (3) MI within the preceding 2 months, or within the preceding 3 weeks in case of incessant VT; (4) unstable angina; (5) severe aortic stenosis or	Age 64 ± 9 years<br><br>Male sex, 56 (89%)<br><br>LVEF 30% ± 13%<br><br>All patients had remote MI and presented with a median number of 17 (range 1–380) VTs in the preceding 6 months.<br><br>The mean follow-up period was 12 ± 3 months.	Ablation was acutely successful in 51 (81%) patients. One (1.5%) patient experienced a major complication with degeneration of VT into VF, necessitating cardiopulmonary resuscitation maneuvers. However, no death occurred acutely or within the first 30 days after catheter ablation.<br><br>During the follow-up, 19 (37%) of the initially successful ablated patients and 31 (49%) of all ablated patients developed some type of VT recurrence.	Limitations:<br>- No randomization<br>- Small number of patients<br><br>Other comments and conclusions: Anticoagulation with heparin targeted an activated clotting time of >250 seconds.

Study name or acronym; Author; Year published; PMID	Aim of study; Endpoints; Study type (RCT, observational — multicenter or single center; case series or other [if RCT include intervention and comparator]); Study size (N)	Patient population with inclusion and exclusion criteria	Results (absolute event rates, *P* values; OR or RR; 95% CI)	Other relevant findings or adverse events	Limitations; Other comments; Conclusions
	defined by the number of acute or subacute (within 1 week) complications associated with catheter ablation.<br><br>Study type: Prospective multicenter<br><br>Study size (N): 63 patients	mitral regurgitation; and (6) unwillingness to participate in the study or unavailable for follow-up visits.			

Study name or acronym; Author; Year published; PMID	Aim of study; Endpoints; Study type (RCT, observational — multicenter or single center; case series or other [if RCT include intervention and comparator]); Study size (N)	Patient population with inclusion and exclusion criteria	Results (absolute event rates, *P* values; OR or RR; 95% CI)	Other relevant findings or adverse events	Limitations; Other comments; Conclusions
Stevenson et al. Irrigated radiofrequency catheter ablation guided by electroanatomic mapping for recurrent ventricular tachycardia after myocardial infarction: the multicenter thermocool ventricular tachycardia ablation trial.<br>• Year published: 2008<br>• PMID: 19064682	Aim: To assess the outcome of VT ablation with a saline-irrigated catheter combined with an EAM system.<br><br>Endpoints:<br>- The prespecified primary endpoint was defined at 6 months of follow-up as no recurrence of SMVT.<br>- For patients with incessant VT, success was defined as no recurrence of incessant VT.<br><br>Study type: Observational multicenter<br><br>Study size (N): 231 patients	Entry criteria were SMVT requiring termination by cardioversion or AAD administration with ≥4 episodes in the previous 6 months despite an ICD or AAD therapy. Patients without ICDs were eligible after 2 episodes of SVT.<br><br>Exclusion criteria included serum creatinine >2.5 mg/dL, LVEF ≤0.10, mobile LV thrombus on echocardiography, absence of vascular access to the LV, disease process likely to limit survival to <12 months, NYHA class IV HF, cardiac surgery within the past 2 months (unless VT was incessant), unstable angina, severe aortic stenosis or mitral regurgitation with a flail leaflet, pregnancy, and age <18 years.	Median age, 68 years<br><br>Male sex, 89%<br><br>Median LVEF, 0.25%<br><br>HF in 62% (previous inferior MI in 63% of patients)<br><br>Patients had a median of 11 episodes of VT in the preceding 6 months.<br><br>A prior ablation procedure had been performed in 37% of patients.	Ablation abolished all inducible VTs in 49% of patients.<br><br>The primary endpoint of freedom from recurrent incessant VT or intermittent VT after 6 months of follow-up was achieved for 123 patients (53%).<br><br>In 142 patients with ICDs before and after ablation for intermittent VT who survived 6 months, VT episodes were reduced from a median of 11.5 to 0 (*P*<.0001).<br><br>The 1-year mortality rate was 18%, with 72.5% of deaths attributed to VAs or HF. The procedure mortality rate was 3%, with no strokes.	Limitations:<br>- Group of patients with advanced heart disease.<br>- The follow-up period was short (only 6 months for assessment of recurrent arrhythmias and 12 months for death).<br>- Although continued AAD therapy was recommended, drugs were often reduced after a successful ablation, as allowed for adverse effects. In some cases, removal of a proarrhythmic drug effect could have contributed to a beneficial outcome; in other cases, emergence of a VT that was suppressed by the drug at the time of ablation could have contributed to procedure failure.<br>Other comments and conclusions:<br>- This study was prospective, was analyzed according to intention to treat (including patients who did not receive ablation), and had external monitoring that could have improved the rigor for detecting endpoints and complications compared with some prior reports.<br>- A 3-month course of warfarin was used post-procedurally if ablation had been performed over an area with >3 cm distance between ablation sites; otherwise, full-dose aspirin was used.

Study name or acronym; Author; Year published; PMID	Aim of study; Endpoints; Study type (RCT, observational — multicenter or single center; case series or other [if RCT include intervention and comparator]); Study size (N)	Patient population with inclusion and exclusion criteria	Results (absolute event rates, *P* values; OR or RR; 95% CI)	Other relevant findings or adverse events	Limitations; Other comments; Conclusions
Siontis et al. Thromboembolic prophylaxis protocol with warfarin after radiofrequency catheter ablation of infarct-related ventricular tachycardia.<br>• Year published: 2018<br>• PMID: 29315941	Aim: To assess the feasibility, safety, and effectiveness of a thromboembolic prophylaxis protocol in patients undergoing catheter ablation for infarct-related VT.<br><br>Endpoints:<br>- Procedural success was classified as complete when no SMVT was inducible, partial when only nonclinical VTs were inducible, and failed when clinical VTs were inducible at the end of the procedure.<br>- The first incident thromboembolic (stroke, TIA, peripheral arterial embolism) and bleeding events in the 3 months postablation were documented (in-hospital and postdischarge)<br><br>Study type: Cohort<br><br>Study size (N): 217 patients	Consecutive patients undergoing ablation for infarct-related VT with open irrigated-tip catheters between 2008 and 2015. Patients either had a clinical history of MI or evidence of prior infarction on cardiac imaging.	Age 67.8 ± 9.1 years<br><br>Male sex, 199 (92%)<br><br>LVEF (%) 27 (20–40)	Some 11 (6%) patients experienced bleeding events; 1 required endovascular intervention and 1 (0.6%) experienced lower extremity arterial embolism requiring vascular surgery.<br><br>Systemic anticoagulation was prescribed in 190 (89%) of 214 patients discharged from the hospital (warfarin in 98%), whereas the rest received single- or dual-antiplatelet therapy alone.<br><br>Patients treated with an anticoagulant had significantly longer RF time compared with patients treated with antiplatelet agents only. One (0.5%) of the patients treated with oral anticoagulation experienced major bleeding 2 weeks postablation. No thromboembolic events were documented in either the anticoagulation or the “antiplatelet only” group post discharge.	Limitations:<br>- Retrospective study<br>- The majority of patients underwent extensive LV ablation; thus, the number of patients treated only with antiplatelet agents was small.<br>- Small study population<br>- Absence of control group<br><br>Other comments and conclusions: patients with large LV endocardial ablation area (>3 cm between ablation lesions) were started on low-dose, slowly escalating UFH infusion 8 hours after access hemostasis, followed by 3 months of anticoagulation. Patients with less extensive ablation were treated only with antiplatelet agents postablation. Postablation bridging anticoagulation was used in 181 (83%) patients.

Study name or acronym; Author; Year published; PMID	Aim of study; Endpoints; Study type (RCT, observational — multicenter or single center; case series or other [if RCT include intervention and comparator]); Study size (N)	Patient population with inclusion and exclusion criteria	Results (absolute event rates, *P* values; OR or RR; 95% CI)	Other relevant findings or adverse events	Limitations; Other comments; Conclusions
Al-Khatib et al. Catheter ablation for ventricular tachycardia in patients with an implantable cardioverter defibrillator (CALYPSO) pilot trial.<br>• Year published: 2015<br>• PMID: 25332150	Aim: To determine the feasibility of a large, multicenter clinical trial aimed at testing whether early use of percutaneous catheter ablation of VT is superior to AADs at reducing all-cause mortality in patients with an ICD who receive ICD therapy (shock and/or ATP) for monomorphic VT in the absence of a reversible cause.<br><br>The primary endpoint: Feasibility<br>Other endpoints:<br>- Death<br>- Time to first recurrent therapy for VT<br>- Recurrent VT<br>- Adverse events related to the procedure or to the use of antiarrhythmic medications<br>- Hospitalizations for VT<br><br>A pilot multicenter, randomized clinical trial at 4 sites; N=27: 13 were randomized to the	Inclusion criteria:<br>- Over 18 years of age<br>- Have an ICD with or without cardiac resynchronization therapy that was implanted for a primary or a secondary prevention indication.<br>- IHD defined as the presence of wall motion abnormalities and documented CAD (1 >70% stenosis in at least 1 major coronary artery)<br>- At least 1 documented ICD shock or >2 ATP therapies within 6 months before randomization for MMVT (rate <260 beats/minute) in the absence of a reversible cause that, in the opinion of the treating physician, required further therapy<br>- Eligible for catheter ablation<br>- Have no history of intolerance or contraindication to at least 1 of the following antiarrhythmic medications: amiodarone,	Of 243 patients screened, 27 met all eligibility criteria.<br>The common reasons for failing screening:<br>- The patient had already been on an AAD for VT<br>- VT was due to a reversible cause<br>- Incessant VT<br>13 patients were randomized to the catheter ablation arm, and 14 to the antiarrhythmic medication arm.<br>Eleven (85%) of the 13 patients randomized to the catheter ablation underwent the procedure. Of these 11 patients, 5 were also started on an AAD. One of the 14 patients randomized to the AAD crossed over to the catheter ablation arm.<br>Of the deaths that occurred during the	The median age was 64 years. The vast majority of patients were male and white. The median number of sustained and/or treated VT episodes in the preceding 6 months was 3.<br><br>Patients in this trial had relatively severe cardiovascular disease: a history of HF in 85%; a median LVEF of 25%; biventricular pacing in 22%; a history of AF in 33%.<br><br>A total of 7 (26%) patients had used amiodarone prior to enrollment. At enrollment, 25 (93%) were on a BB, and 25 (93%) patients were on an angiotensin-converting enzyme-inhibitor or an angiotensin-receptor blocker.<br><br>Of 27 patients, 23 (92%) completed 3 months of follow-up and 17 (71%) completed 6 months of follow-up; 4 patients (2 in	Conclusion:<br>Most patients in clinical practice have already failed AAD therapy before catheter ablation is considered, and the VT recurrence rates and death in these patients are high. For a large clinical trial to be feasible, factors limiting early consideration of catheter ablation need to be identified and addressed. One important observation in this study is that only 18 (8%) patients of the 216 who failed screening did so because of refusal to participate in the trial.<br><br>Limitations:<br>The short follow-up; the small sample size and the lack of statistical power to show a difference rather than differences in patient populations or study design.<br><br>The event rate in this study was relatively high, with 4 of 27 (15%) patients dying within 6 months of follow-up and 14 of 27 (52%) patients developing recurrent VT within 6 months of follow-up. Some 5 of 11 patients who underwent ablation required an AAD during follow-up.

Study name or acronym; Author; Year published; PMID	Aim of study; Endpoints; Study type (RCT, observational — multicenter or single center; case series or other [if RCT include intervention and comparator]); Study size (N)	Patient population with inclusion and exclusion criteria	Results (absolute event rates, *P* values; OR or RR; 95% CI)	Other relevant findings or adverse events	Limitations; Other comments; Conclusions
	catheter ablation arm and 14 to the AAD arm.	sotalol, mexiletine, ranolazine, and dofetilide.<br><br>Exclusion criteria: Incessant VT; contraindication to catheter ablation of VT; nonischemic; hypertrophic obstructive, restrictive, or infiltrative cardiomyopathy; acute myocarditis; CHD; valvular disease likely to require surgery in the following year; inoperable obstructive valvular disease; heart transplant or imminent cardiac transplantation within 12 months; an LV assist device; heritable arrhythmias or increased risk for TdP with class III drugs; end-stage renal disease; estimated life expectancy of <1 year from a noncardiac cause; women who are pregnant or who have childbearing potential and are not using a reliable method of contraception.	6-month follow-up period, 3 were deemed to be cardiac; 1 was due to progressive heart failure with no VT or VF; 1 patient died of a noncardiac cause after the 6-month visit.<br>A total of 14 patients had recurrent VT: 8 (62%) in the ablation arm, 6 (43%) in the antiarrhythmic medication arm. The median time to recurrent VT: 75 days in the ablation arm, 57 days in the antiarrhythmic arm.<br>Three patients developed heart failure: 2 (15%) in the ablation arm, 1 (7%) in the AAD arm.<br>A total of 12 patients were hospitalized for VT: 5(46%) in the ablation arm, 7 (50%) in the AAD arm.	each arm) did not complete follow-up due to death; 4 patients (2 in each arm) had not reached the follow-up mark when the study was terminated. No patient was lost to follow-up.<br><br>Eight patients developed a serious adverse event: 3 (23%) in the ablation arm (in 1 patient an access site hematoma and renal failure); 5 (36%) in the AAD arm.	

Sinus Rhythm Substrate Mapping

Study name or acronym; Author; Year published; PMID	Aim of study; Endpoints; Study type (RCT, observational — multicenter or single center; case series or other [if RCT include intervention and comparator]); Study size (N)	Patient population with inclusion and exclusion criteria	Results (absolute event rates, *P* values; OR or RR; 95% CI)	Other relevant findings or adverse events	Limitations; Other comments; Conclusions
Di Biase et al. Ablation of stable VTs versus substrate ablation in ischemic cardiomyopathy: the VISTA randomized multicenter trial.<br>• Year published: 2015<br>• PMID: 26718674	Aim: To determine rates of VT recurrence in patients undergoing ablation limited to clinical VT along with mappable VTs ("clinical ablation") vs substrate-based ablation.<br><br>Endpoints:<br>- Primary endpoints: Recurrence of any VT during the 12-month postablation period, as demonstrated by device interrogation and clinical evaluation.<br>- Secondary endpoints: Periprocedural complications, 12-month postprocedure mortality, rehospitalization, and combined incidence of rehospitalization and mortality.<br><br>Study type: Randomized multicenter<br><br>Study size (N): 118 patients	Inclusion criteria: patients with ICM who had received an ICD before the ablation and had recurrent stable MMVTs that were symptomatic or required ICD therapies despite AADs. Between April 2009 and July 2013, patients from 7 centers were randomized to substrate-based ablation (n=58) or to ablation of clinical and mappable VTs only (n=60).<br><br>Exclusion criteria: VT that presented with syncope, loss of consciousness, or cardiac arrest; age <18 years; severe renal (glomerular filtration rate 15 to 29 ml/min/1.73 m^2 for ≥3 months), LV thrombus, unstable angina, severe aortic stenosis, end-stage HF with limited life expectancy, and prior failed VT ablation.	Baseline clinical characteristics and electrophysiologic features were not significantly different between the substrate ablation and clinical ablation groups; mean LVEF was 32.0% ± 9.9% and 32.6% ± 14.1% (*P*=.8), and patients failed an average of 1.4 ± 0.7 and 1.5 ± 0.7 AADs, respectively (*P*=.86).<br><br>After ablation, both groups underwent the same preablation induction protocol. In the clinical ablation group, the average number of postablation unstable nonclinical VTs induced was lower than that observed at the beginning (2.0 ± 1.4 vs 2.7 ± 1.2; *P*=.011).<br><br>Age (5-year increments),	At 12-month follow-up, 9 (15.5%) and 29 (48.3%) patients had VT recurrence in the substrate-based and clinical VT ablation groups, respectively (log-rank *P*<.001).<br><br>More patients undergoing clinical VT ablation (58%) were on AADs after ablation versus substrate-based ablation (12%; *P*<.001).<br><br>Seven (12%) patients with substrate ablation and 19 (32%) with clinical ablation required rehospitalization (*P*=.014).<br><br>Overall 12-month mortality was 11.9%; 8.6% in the substrate ablation and 15.0% in the clinical ablation groups, respectively (log-rank *P*=.21). Combined incidence of rehospitalization and mortality was significantly lower with substrate	Limitations:<br>- Complete elimination of abnormal potentials within the scar tissue in the substrate ablation group was not possible in 16% of the population.<br>- Determination of whether an induced VT is "clinical" is sometimes difficult because similarity in cycle lengths and intracardiac ICD morphologies are imperfect predictors.<br><br>Other comments and conclusions:<br>- Combined reduction of rehospitalization and mortality was observed in the substrate ablation group.<br>- This is the first randomized multicenter study showing that substrate-based ablation reduces recurrence of any VT at follow-up when compared with ablation limited to clinical and mappable VTs in patients with ICM, suggesting that a larger area of scar

Study name or acronym; Author; Year published; PMID	Aim of study; Endpoints; Study type (RCT, observational — multicenter or single center; case series or other [if RCT include intervention and comparator]); Study size (N)	Patient population with inclusion and exclusion criteria	Results (absolute event rates, *P* values; OR or RR; 95% CI)	Other relevant findings or adverse events	Limitations; Other comments; Conclusions
			male sex, electrical storm, LVEF, diabetes, and substrate-based ablation showed significant unadjusted association with VT recurrence.	ablation (*P*=.003).<br><br>Periprocedural complications were similar in both groups (*P*=.61).<br><br>After adjusting for covariates in a Cox multivariate model, substrate-based ablation was associated with 67% lower risk of recurrence compared with clinical ablation (HR 0.33; 95% CI 0.13–0.81; *P*=.014).	tissue must be ablated to reduce recurrence of any VTs in patients with ICM and stable clinical VTs.<br>- A larger percentage of patients was able to discontinue AADs after substrate ablation, an important finding because AADs can have significant long-term adverse effects.

Study name or acronym; Author; Year published; PMID	Aim of study; Endpoints; Study type (RCT, observational — multicenter or single center; case series or other [if RCT include intervention and comparator]); Study size (N)	Patient population with inclusion and exclusion criteria	Results (absolute event rates, *P* values; OR or RR; 95% CI)	Other relevant findings or adverse events	Limitations; Other comments; Conclusions
Reddy et al. Prophylactic catheter ablation for the prevention of defibrillator therapy.<br>• Year published: 2007<br>• PMID: 18160685	Aim: To examine whether prophylactic RFCA of arrhythmogenic ventricular tissue would reduce the incidence of ICD therapy.<br><br>Over the course of 46 months, 128 patients were enrolled and randomly assigned to the ablation group or the control group (64 in each group).<br><br>Endpoints:<br>- Primary endpoint: Survival free from any appropriate ICD therapy (shock or ATP).<br>- Secondary endpoint: Freedom from any appropriate ICD shock, death, or ICD storm.<br><br>Study type: Prospective, unblinded, randomized, controlled multicenter trial<br><br>Study size (N): 128 patients	Inclusion criteria: Men and women who were at least 18 years of age with a history of MI more than 1 month before enrollment and if they had undergone a planned or a recent (within 6 months) implantation of a defibrillator for VF, hemodynamically unstable VT, or syncope with inducible VT during invasive electrophysiological testing.<br><br>Exclusion criteria: Patients being treated with a class I or class III AAD, if the substrate for the VA was thought not to be due to the MI, if active and ongoing cardiac ischemia was thought to be the cause of the VA, or if they were having incessant or multiple episodes of VT necessitating immediate treatment (with drugs or ablation). Additional exclusion criteria included	Age 67 years<br><br>Male sex, 87%<br><br>The qualifying index arrhythmia was VF in 18% of patients, VT in 49%, syncope with inducible VT in 21%, and recent VF or VT treated by a previously implanted ICD in 12%.<br><br>LVEF was 30.7% ± 9.5% in the ablation group and 32.9% ± 8.5% in the control group (*P*=.16).<br><br>LVEF was <20% in 16 patients in the ablation group and in 7 patients in the control group (*P*=.06).<br><br>The two groups were well balanced with respect to baseline characteristics, and a high percentage received BBs, antagonists of the renin–angiotensin–aldosterone system, and statin drugs.	The mortality rate 30 days after ablation was 0%, and there were no significant changes in ventricular function or functional class during the mean (±SD) follow-up period of 22.5 ± 5.5 months.<br><br>Some 21 patients assigned to defibrillator implantation alone (33%) and 8 patients assigned to defibrillator implantation plus ablation (12%) received appropriate ICD therapy (ATP or shocks) (HR in the ablation group, 0.35; 95% CI 0.15–0.78; *P*=.007). Among these patients, 20 in the control group (31%) and 6 in the ablation group (9%) received shocks (*P*=.003). Multivariate analysis adjusted for patient characteristics and medication use at baseline did not reduce the magnitude of the effect observed (HR 0.31; 95% CI 0.13–0.76; *P*=.01).<br><br>Compared with the control	Limitations:<br>- Small number of participants<br>- Absence of quality of life information<br>- Long recruitment period (4 years)<br>- No standardized ATP algorithm<br>- Selection of only high-volume, highly experienced ablation centers.<br><br>Other comments and conclusions:<br>- Ablation was performed with the use of a substrate-based approach in which the myocardial scar is mapped and ablated while the heart remains predominantly in SR.<br>- During follow-up (22.5 ± 5.5 months; range, 0–26), no patient received an AAD (other than BBs) before the primary endpoint was reached.<br>- There was no significant change in ventricular function or functional status during follow-up. This remained true even for those patients with

Study name or acronym; Author; Year published; PMID	Aim of study; Endpoints; Study type (RCT, observational — multicenter or single center; case series or other [if RCT include intervention and comparator]); Study size (N)	Patient population with inclusion and exclusion criteria	Results (absolute event rates, *P* values; OR or RR; 95% CI)	Other relevant findings or adverse events	Limitations; Other comments; Conclusions
		the inability to give informed consent, stroke within 30 days before screening, contraindication to anticoagulation therapy, or any medical or nonmedical condition likely to prevent completion of the trial.		population, patients in the ablation group had a 65% reduction in the risk of receiving ICD therapy during the subsequent 2 years. When ATP therapy was excluded from this analysis, there remained a 73% reduction in the risk of receiving subsequent ICD shocks.<br><br>There was a trend toward decreased mortality in the ablation group compared with the control group (9% vs 17%, *P*=.29). ICD storms occurred in 4 patients assigned to ablation (6%) and 12 control patients (19%) (*P*=.06).<br><br>Mortality was not increased in the ablation group compared with the control group (9% vs 17%; *P*=.29).	the most depressed cardiac function. Overall mortality was not greater in the patients assigned to ablation; indeed, there was even a trend toward decreased mortality in the ablation group, although it was not statistically significant.<br>- In 3 (5%) patients, no appreciable endocardial scar was visualized; therefore, no ablation lesions were placed.<br>- Intravenous heparin was administered to achieve an activated clotting time >220 seconds.

Study name or acronym; Author; Year published; PMID	Aim of study; Endpoints; Study type (RCT, observational — multicenter or single center; case series or other [if RCT include intervention and comparator]); Study size (N)	Patient population with inclusion and exclusion criteria	Results (absolute event rates, *P* values; OR or RR; 95% CI)	Other relevant findings or adverse events	Limitations; Other comments; Conclusions
Acosta et al. Multielectrode vs point-by-point mapping for ventricular tachycardia substrate ablation: a randomized study.<br>• Year published: 2018<br>• PMID: 28069835	Aim: To analyze whether high-density MEM is superior to conventional PPM in guiding VT substrate ablation procedures.<br><br>Endpoints: Clinical and nonclinical VTs induced after scar dechanneling were targeted for ablation.<br><br>The acute results of the ablation procedure (VT inducibility after ablation) and midterm outcomes (VT recurrence-free survival at 12 months) were compared between groups.<br><br>Study type: Randomized controlled trial<br><br>Study size (N): 20 patients	Consecutive patients with IHD undergoing VT substrate ablation were included from September 2013 to December 2015.<br><br>Inclusion criteria were age >18 years, the presence of prior MI, and a symptomatic episode of SMVT. Patients were randomized (1:1) to group A (n= 10; substrate mapping performed first by PPM [Navistar] and second by MEM [PentaRay], ablation guided by PPM) or group B (n= 10; substrate mapping performed first by MEM and second by PPM, ablation guided by MEM).	Age 67.3 ± 10.3 years<br><br>Male sex, 95%<br><br>LVEF (%) 34 ± 9 (PPM-guided ablation) and 36 ± 8 (MEM-guided ablation); *P*=.563.<br><br>All patients had ICM.<br><br>In 11 patients (55%), a preprocedural CE-CMR was obtained and integrated into the navigation system.	Larger bipolar scar areas were obtained with MEM (55.7 ± 31.7 vs 50.5 ± 26.6 cm^2; *P*=.017).<br><br>Substrate mapping time was similar with MEM (19.7 ± 7.9 minutes) and PPM (25 ± 9.2 minutes); *P*=.222. No differences were observed in the number of LPs identified within the scar by MEM vs PPM (73 ± 50 vs 76 ± 52 LPs per patient, respectively; *P*=.965). Despite the concordance between MEM and PPM in the identification of CCs, the proportion identified by voltage scanning was higher when using MEM (71.7% vs 56.3%, respectively; *P*=.024).<br><br>A total of 1104 LP pairs were analyzed. Using PentaRay, the far-field/LP ratio was significantly lower (0.58 ± 0.4 vs 1.64 ± 1.1; *P*=.01) and RF time was shorter (median IQR 12 (7–20) vs 22 (17–33) minutes; *P*=.023).	Limitations:<br>- Single-center, randomized controlled pilot study<br>- Small study cohort<br>- Only ischemic patients were included<br>- EGM-DC paired analysis was performed manually by an experienced electrophysiologist. Automatic algorithms are required to improve the reproducibility and standardization of this analysis.<br><br>Other comments and conclusions:<br>- The scar dechanneling technique was used in all ablation procedures. Conducting channels were identified as either voltage channels or LP channels.<br>- This is the first randomized clinical study assessing the potential benefits of MEM vs conventional PPM for VT substrate ablation in ischemic patients.<br>- In 2 (10%) patients, MEM had to be interrupted due to

Study name or acronym; Author; Year published; PMID	Aim of study; Endpoints; Study type (RCT, observational — multicenter or single center; case series or other [if RCT include intervention and comparator]); Study size (N)	Patient population with inclusion and exclusion criteria	Results (absolute event rates, *P* values; OR or RR; 95% CI)	Other relevant findings or adverse events	Limitations; Other comments; Conclusions
				No differences were observed in VT inducibility after the procedure. Complete scar dechanneling (complete elimination of CC-EGMs) was achieved in 17 patients (85%). At 12 months postprocedure, 2 (20%) patients showed VT recurrence.	VF induced by multielectrode catheter manipulation.<br>- The main findings are as follows:<br>(i) MEM with PentaRay catheter provided a lower sensitivity for far-field signals;<br>(ii) scar dechanneling guided by MEM was associated with a shorter RF time.

Study name or acronym; Author; Year published; PMID	Aim of study; Endpoints; Study type (RCT, observational — multicenter or single center; case series or other [if RCT include intervention and comparator]); Study size (N)	Patient population with inclusion and exclusion criteria	Results (absolute event rates, *P* values; OR or RR; 95% CI)	Other relevant findings or adverse events	Limitations; Other comments; Conclusions
Carbucicchio et al. High-density substrate-guided ventricular tachycardia ablation: role of activation mapping in an attempt to improve procedural effectiveness.<br>• Year published: 2013<br>• PMID: 24055940	Aim: To investigate whether a conventional activation map further contributes to the identification of critical sites of VT reentry and whether this translates into a more effective ablation outcome in a cohort of patients undergoing VT ablation.<br><br>Study type: Cohort<br><br>Study size (N): 126 patients	From 296 consecutive patients undergoing ablation for VAs at the Centro Cardiologico Monzino (Milan, Italy) and at the Texas Cardiac Arrhythmia Institute at St David's Medical Center (Austin, TX) from June 2010 to September 2011, 126 patients with ICM or IDCM and recurrent SVT (≥2 episodes) were enrolled in whom the CARTO system (Biosense Webster, Diamond Bar, CA) was used to guide ablation.	Mean age 65.3 ± 10.5 years<br><br>ICM (86 of 126 [70.6%]) or IDCM (37 of 126 [29.%])<br><br>LVEF 33.3% ± 7.2%<br><br>Undergoing endocardial (n=105) or endo-epicardial (n=21) EAM and ablation.<br><br>In 62 of 104 (59.6%) patients, the activation map was acutely effective.<br><br>Among clinical variables, IDCM was the only statistically significant predictor of VT recurrence (*P*<.001).	The activation map successfully guided ablation in 62 of 104 (59.6%) patients with inducible VT(s). At 1 year, 6 of 126 (4.8%) patients died; VT recurred in 28 of 126 (22.2%) patients.<br><br>No significant difference in VT recurrence rate was observed between patients in whom the activation map proved effective vs those in whom substrate-guided ablation was not corroborated by the activation map (16 of 62 [25.8%] vs 12 of 64 [18.8%]; log-rank test, *P*=.3).<br><br>In the acute setting, complete success was achieved in 89 of 104 (85.6%), partial success was achieved in 7 of 104 (6.7%), and failure in 2 of 104 (1.9%) patients.	Limitations:<br>- Not randomized.<br>- The use of amiodarone could have played a role with regard to the overall high procedural success rate. In this series, however, amiodarone discontinuation was not routinely attempted.<br><br>Other comments and conclusions:<br>- The 1-year recurrence-free survival was 80.9% after a complete success (72 of 89), 42.9% after a partial success (3 of 7), and 0% after a failure (0 of 2).<br>- Patients who underwent failed activation map attempts did not present a higher VT recurrence rate, indicating that the accomplishment of an ablation strategy guided by high-density substrate mapping reliably achieves control of long-term recurrences.

Study name or acronym; Author; Year published; PMID	Aim of study; Endpoints; Study type (RCT, observational — multicenter or single center; case series or other [if RCT include intervention and comparator]); Study size (N)	Patient population with inclusion and exclusion criteria	Results (absolute event rates, *P* values; OR or RR; 95% CI)	Other relevant findings or adverse events	Limitations; Other comments; Conclusions
Cano et al. Utility of high density multielectrode mapping during ablation of scar-related ventricular tachycardia.<br>• Year published: 2017<br>• PMID: 28744991	Aim: To evaluate the utility of multielectrode mapping catheters (MEMCs) during scar-related VT ablation procedures.<br><br>Endpoints:<br>- Primary endpoints: To evaluate the utility of the MEMC during VT ablation in terms of total procedural time and endocardial/epicardial mapping times.<br>- Secondary endpoints: To evaluate the mapping density obtained with the MEMC.<br>- Other secondary objectives included the comparison of radiation exposure parameters, the incidence of procedure-related adverse events, and the need for additional mapping with the linear catheter as an indicator of possible limitations in the mapping capabilities of the MEMC.	Consecutive patients undergoing scar-related ablation at the Hospital Universitari i Politècnic La Fe.<br><br>In order to establish comparisons, consecutive patients who underwent scar-related VT ablation with a standard linear catheter were retrospectively reviewed in the database of the Universitari i Politècnic La Fe (control group).	The baseline characteristics of the patients were comparable between the two groups<br><br>A total of 28 patients underwent epicardial access and mapping (12 patients in the control group and 16 patients in the study group; *P*=.45).<br><br>An endocardial RV map was more frequently performed in the MEMC group (49.1% vs 19.2%; *P*=.015).<br><br>Activation mapping during the induced VT was performed in 26.9% of patients treated with a linear catheter and in 39% of patients treated with the MEMC (*P*=.33).	The use of the MEMC resulted in a significant shortening of the endocardial and epicardial mapping times (38 ± 15 vs 56 ± 24 minutes for endocardial LV mapping in the study and control group, respectively; *P*=.001; and 28 ± 9 vs 41 ± 16 minutes for epicardial mapping; *P*=.011) as well as the total procedural time (177 ± 53 vs 206 ± 50 minutes, respectively, *P*=.02).<br><br>The mapping density was also significantly increased in the study group (mean endocardial LV points: 2143 ± 1419 vs 485 ± 174 for the study and control group, respectively; *P*<.0001), as well as mean epicardial points (3327 ± 1825 vs 877 ± 258 points, respectively; *P*<.0001), especially within the scar area (49.6 ± 34 vs 8.4 ± 4.6 points/cm^2; *P*<0.001).<br><br>Radiation exposure was	Limitations:<br>- Single-center nonrandomized observational study.<br>- The same patient was not sequentially mapped with the linear catheter and with the MEMC so the comparison of the performance of the two catheters could not be directly evaluated.<br><br>Other comments and conclusions:<br>This is the first detailed description of the clinical utility of multielectrode mapping during scar-related VT ablation in a large series (including both ICM and NICM) specifically designed with this purpose.

Study name or acronym; Author; Year published; PMID	Aim of study; Endpoints; Study type (RCT, observational — multicenter or single center; case series or other [if RCT include intervention and comparator]); Study size (N)	Patient population with inclusion and exclusion criteria	Results (absolute event rates, *P* values; OR or RR; 95% CI)	Other relevant findings or adverse events	Limitations; Other comments; Conclusions
	Study type: Observational prospective<br><br>Study size (N): 81 patients (85 scar-related VT ablations)			significantly reduced in the MEMC group. Overall, the mean fluoroscopy time and total dose area product were 6.4 ± 5.8 minutes in the study group vs 11.4 ± 8.9 minutes in the control group, and 201 ± 304 cGy cm^2 vs 445 ± 529 cGy cm^2, *P*=.014 and *P*=.036, respectively.<br><br>Although there were no significant differences in the acute and long-term outcomes between the 2 groups, there was a trend toward a lower rate of VT recurrence during follow-up in the study group (log-rank *P*=.082) (acute setting: complete success 70.2% for the study group and 73.1% for the control group, *P*=.98). By the end of follow-up, 93.2% of the patients in the study group were free of VT recurrence compared with 73.1% in the control group (log-rank *P*=.082).	

Study name or acronym; Author; Year published; PMID	Aim of study; Endpoints; Study type (RCT, observational — multicenter or single center; case series or other [if RCT include intervention and comparator]); Study size (N)	Patient population with inclusion and exclusion criteria	Results (absolute event rates, *P* values; OR or RR; 95% CI)	Other relevant findings or adverse events	Limitations; Other comments; Conclusions
Volkmer et al. Substrate mapping vs tachycardia mapping using CARTO in patients with coronary artery disease and ventricular tachycardia: impact on outcome of catheter ablation.<br>• Year published: 2006<br>• PMID: 17043071	Aim: To evaluate success rate of VT ablation using CARTO, with a subgroup analysis comparing VT mapping with the results of mapping that had to be performed during SR or pacing (substrate mapping).<br><br>Endpoints:<br>The endpoint of focal applications in the VT-mapping group was VT termination by RF and in the substrate-mapping group, the elimination of isolated potentials. The endpoint for linear ablation (lines or cross-lines) was the completion of the designed lines. Acute success was defined as noninducibility of the clinical VT and any VT slower than the clinical VT, either at the end of the ablation procedure (VT-mapping group) or in a repeat EP stimulation study before hospital discharge (substrate-	Consecutive patients with clinically sustained, hemodynamically tolerated MMVT remote from MI (>3 months), who underwent endocardial catheter mapping and RFCA by use of the EAM system CARTO.	Age (mean+SD) 65 ± 7 (VT group), 65 ± 9 (substrate group); *P*=.979.<br><br>Male/female sex 21/1(VT group), 22/3 (substrate group); *P*=.611.<br><br>EF (%), 30 ± 7 (VT group), 30 ± 8 (substrate group); *P*=.921.<br><br>At the time of study inclusion, 43 of 47 patients were on AAD therapy.<br><br>The mean follow-up period of the entire cohort was 25 ± 13 months (range 4–48 months).	Acute ablation success in all patients with regard to noninducibility of the clinical VT or any slower VT was 79% after a single ablation procedure but increased to 95% after a mean of 1.2 ablation procedures. However, chronic success was 75% when it was defined as freedom from any ventricular tachyarrhythmia (VT or VF) during a follow-up of 25 ± 13 months.<br><br>In the subgroup analysis, patients in the VT-mapping group were not significantly different from patients in the substrate-mapping group with regard to age (65 ± 7 vs 65 ± 9 years), EF (30% ± 7% vs 30% ± 8%), VT cycle length (448 ± 81 vs 429 ± 82 milliseconds), number of RF applications (17 ± 9 vs 14 ± 6 applications), use of an irrigated tip catheter (23% vs 32%), and ablation results.	Limitations:<br>- Not a randomized investigation prospectively comparing VT-mapping and substrate mapping.<br>- In most patients in both groups, prophylactic linear RF ablations were applied for connection of electrical barriers, or ablation targeted diastolic potentials during SR or pacing, suggesting areas of slow conduction. The identical outcome of both groups could be related to these additional prophylactic lines.<br>- Irrigated tip ablation was used in only 28% of procedures; however, to the same extent in both groups.<br>- Comparing VT mapping and substrate mapping can be criticized because there is some heterogeneity from nonsystematic variation in lesion strategy (focal/linear), ablation delivery (conventional/cooled-tip), using limited VT induction and entrainment criteria in the substrate-mapping group, and not targeting VTs faster than

Study name or acronym; Author; Year published; PMID	Aim of study; Endpoints; Study type (RCT, observational — multicenter or single center; case series or other [if RCT include intervention and comparator]); Study size (N)	Patient population with inclusion and exclusion criteria	Results (absolute event rates, *P* values; OR or RR; 95% CI)	Other relevant findings or adverse events	Limitations; Other comments; Conclusions
	mapping group), but which allowed induction of fast VT or VF.<br>In both groups, chronic success during follow-up was divided into freedom from targeted VTs (defined as no recurrence of the clinical VT or any VT slower than the clinical VT) and freedom from any VT/VF (defined as no occurrence of sustained VA, including fast VT and VF).<br><br>Study type: Cohort<br><br>Study size (N): 47 patients				the clinical VT.<br>- Only clinical or slower VT but not faster VT were ablated. This could have led to a lower success rate compared with centers that target all induced VT morphologies. The rationale for not targeting the faster VT was that they must have a shorter zone of slow conduction than the clinical VT and that the ICD could be easily programmed for their effective termination.<br>Other comments and conclusions: The main finding of this study was that the outcome of the patients was the same, no matter whether complete VT mapping could be performed or only substrate mapping. This indicates that careful mapping of the infarct area during SR or pacing is as effective to identify critical channels as it is during VT mapping, the latter often being associated with a higher risk of jeopardizing the patient’s health due to abnormal hemodynamics.

Study name or acronym; Author; Year published; PMID	Aim of study; Endpoints; Study type (RCT, observational — multicenter or single center; case series or other [if RCT include intervention and comparator]); Study size (N)	Patient population with inclusion and exclusion criteria	Results (absolute event rates, *P* values; OR or RR; 95% CI)	Other relevant findings or adverse events	Limitations; Other comments; Conclusions
Vergara et al. Late potentials abolition as an additional technique for reduction of arrhythmia recurrence in scar related ventricular tachycardia ablation.<br>• Year published: 2012<br>• PMID: 22486970	Aim: To evaluate the efficacy of RF VT ablation targeting complete LP activity<br><br>Endpoints: Primary endpoint is complete abolition of all LP areas<br><br>Study type: Cohort<br><br>Study size (N): 64 patients	Consecutive patients with CAD or IDCM undergoing RFCA for recurrent VTs were evaluated for this study.	Age 63.6 ± 13.6 years<br><br>Male sex, 60 (64%)<br><br>A total of 41 (64.1%) patients had CAD and 23 patients (35.9%) had IDCM<br><br>Mean LVEF 32.2% ± 9.4% in patients with CAD and 35.6% ± 10.0% in patients with IDCM.<br><br>Some 21 (58.0%) patients underwent endocardial mapping only, 3 patients epicardial mapping only; in 18 cases (36.0%) endoepicardial maps were obtained in the same procedure (7 patients with CAD [accounting for 20.6% of CAD patients], 11 patients with IDCM [52.4% of IDCM patients]).<br><br>Clinical characteristics of patients without LPs did not differ from those of patients with LPs, except for LVEF that was 33.3% ±	Some 50 patients had LPs at EAM; 35 patients had at least 1 VT inducible at basal programmed stimulation.<br><br>After substrate mapping, RFCA was performed with the endpoint of all LP abolition. Complete abolition of LPs was feasible in 42 of 50 (84.0%) patients. LPs could not be abolished in 5 patients despite extensive ablation.<br><br>At the end of procedure, prevention of VT inducibility was achieved in 25 of 35 (71.4%) patients with previously inducible VT; VT was still inducible in 5 of 8 patients with incomplete LP abolition; and in 5 of 42 (16.1%) patients with complete LP abolition (*P*<.01).<br><br>After a follow-up of 13.4 ± 4.0 months, 10 patients (20.0%) had VT recurrences, and one of them died after surgical VT ablation; VT	Limitations:<br>- LPs in this study were considered present only when a delayed electrical activity was recorded after the surface QRS end; further studies are needed evaluate the role of the small and fragmented electrical components occurring within the main QRS deflection.<br>- A combined endoepicardial approach was performed in 39.6% of patients; thus, it cannot be excluded that a complete endocardial and epicardial substrate mapping and modification might have improved the detection rate of LPs and led to different results in the remaining patients.<br>- Various catheters were used in this and previously published studies. Catheter tip size could affect the morphology of the recorded potentials, and the use of smaller electrodes could allow a better definition of the arrhythmogenic substrate.

Study name or acronym; Author; Year published; PMID	Aim of study; Endpoints; Study type (RCT, observational — multicenter or single center; case series or other [if RCT include intervention and comparator]); Study size (N)	Patient population with inclusion and exclusion criteria	Results (absolute event rates, *P* values; OR or RR; 95% CI)	Other relevant findings or adverse events	Limitations; Other comments; Conclusions
			9.6% in patients with LPs and 47.8% ± 9.3% in patients without LPs (*P*<.001).<br><br>A scar tissue area was present in all patients with LPs, with a mean surface extension of 33.1 ± 25.1 cm^2.<br><br>The average extension of the area containing LPs was 22.2 ± 17.0 cm^2.	recurrence was 9.5% in patients with LP abolition (4 of 42 patients) and 75.0% (6 of 8 patients) in those with incomplete abolition (PPV 75%, NPV 90.4%, sensitivity 60.0%, and specificity 95.0%; *P*<.0001); although it was 12.5% (5 of 40 patients) in patients without VT inducibility after the ablation, and 50% (5 of 10 patients) in those with inducible VT (PPV 50%, NPV 87.5%, sensitivity 50.0%, and specificity 87.5%; *P*=.008).	Other comments and conclusions:<br>- This is the first study to compare the predictive values of LP abolition with programmed stimulation to assess the result of VT ablation, showing a relationship between abolition of late activity and prevention of VT inducibility after the ablation procedure.<br>- These data suggest that complete elimination of late activity might constitute an additional endpoint to the VT ablation procedure, offering the advantage of an objective measure of substrate modification that, in contrast to VT inducibility, is not affected by reproducibility issues.

Study name or acronym; Author; Year published; PMID	Aim of study; Endpoints; Study type (RCT, observational — multicenter or single center; case series or other [if RCT include intervention and comparator]); Study size (N)	Patient population with inclusion and exclusion criteria	Results (absolute event rates, *P* values; OR or RR; 95% CI)	Other relevant findings or adverse events	Limitations; Other comments; Conclusions
Di Biase et al. Endo-epicardial homogenization of the scar versus limited substrate ablation for the treatment of electrical storms in patients with ischemic cardiomyopathy.<br>• Year published: 2012<br>• PMID: 22766340	Aim: To investigate the impact on recurrences of 2 different substrate approaches for the treatment of electrical storms (endo-epicardial homogenization of the scar versus limited substrate ablation) in patients with ICM.<br><br>Procedural endpoints: The acute procedural endpoint was the noninducibility of any MMVTs before and after the administration of isoproterenol.<br><br>Study type: Cohort<br><br>Study size (N): 92 patients	Consecutive patients with ICM and ES (defined as ≥3 ICD interventions in 24 hours) undergoing ablation at 5 different institutions were enrolled in this prospective study. All patients had an ICD before the ablation and had ES despite AADs such as BBs, dofetilide, amiodarone, mexiletine, sotalol, and ICD therapies.<br><br>Exclusion criteria included severe renal insufficiency, mobile LV thrombus, unstable angina, severe aortic stenosis, end-stage HF (NYHA functional class IV with a limited survival expectation at 12 months), previous coronary artery bypass graft, age <18 years, and prior failed VA ablations.	Age 62 ± 13 years<br>Male sex, 80%<br>LVEF 26% ± 9%<br>All patients had previously experienced treatment failure with 2 ± 0.9 AADs. Among the groups (1 and 2), no significant difference in baseline clinical and electrophysiologic characteristics was observed. No statistical differences were observed in terms of VT cycle lengths, percentage of hemodynamically stable VTs, and scar size (130 ± 54 vs 137 ± 66 cm^2, *P*=.58). Patients were treated either by confining the RF lesions to the endocardial surface with limited substrate ablation (Group 1, n=49) or underwent endocardial and epicardial ablation of abnormal potentials within the scar (homogenization of the scar, Group 2, n=43).<br><br>As observed from the univariable analysis,	When compared with Group 1, in Group 2, a higher procedure time (3.6 ± 1.3 vs 4.8 ± 1.5 hours, *P*<.001), fluoroscopy time (32 ± 14 vs 38 ±11 minutes, *P*=.017), and RF time (39 ± 17 vs 74 ± 21, *P*<.001) were reported.<br><br>The acute procedural endpoint was achieved in 100% of cases. Induction of very fast VT (CL ≤200 milliseconds) or VF, ventricular flutter, or fast PMVT after ablation occurred in 9 patients (18%) in Group 1 and in 5 patients (12%) in Group 2 (*P*=.37).<br><br>During a mean follow-up of 25 ± 10 months, the VA recurrence rate of any VT was 47% (23 of 49 patients) in Group 1 and 19% (8 of 43 patients) in Group 2 (log-rank *P*=.006). One patient in Group 1 and 1 patient in Group 2 died at follow-up for noncardiac reasons.<br><br>The probability of	Limitations:<br>- Prospective but absence of a formal randomization.<br>- All the procedures have been performed by highly experienced operators.<br>- An endo-epicardial homogenization approach is not suitable for patients with previous coronary artery bypass grafting who, however, represent the minority of patients with MI undergoing coronary revascularization in the current era.<br>- It cannot be excluded that a more dense pace mapping would have resulted in a better outcome in Group 1 patients. However, because a large area around the presumed VT exit site was empirically targeted, it is unlikely that this might explain the lower success rate in Group 1.<br>Other comments and conclusions:<br>- This is the first study showing that extensive ablation within the scar area in patients with IHD and ES increases the freedom from VAs at long-term

Study name or acronym; Author; Year published; PMID	Aim of study; Endpoints; Study type (RCT, observational — multicenter or single center; case series or other [if RCT include intervention and comparator]); Study size (N)	Patient population with inclusion and exclusion criteria	Results (absolute event rates, *P* values; OR or RR; 95% CI)	Other relevant findings or adverse events	Limitations; Other comments; Conclusions
			ablation with homogenization of scar was a determinant of VA-free survival (HR 0.44; 95% CI 0.14–0.86; *P*=.014). No significant association was observed for other covariates tested in the univariable model.<br>Under multivariable analysis, after controlling for important covariates (age, sex, LVEF, VT CL, scar size), ablation with endo-epicardial homogenization of the scar was found to be associated with significantly lower risk for VT recurrence (HR: 0.46; 95% CI 0.21 to 0.87; *P*=.025). Ablation with endo-epicardial homogenization had 62% decreased risk for VT recurrence compared with endocardial ablation alone. Other baseline risk factors such as sex, age, LVEF, and VT CL did not show any significant influence on long-term outcome.	recurrence-free survival was significantly lower in Group 1 (26 [53%] vs 35 [81%], log-rank *P*=.006).	follow-up.<br>- Moreover, in the multivariate analysis, the technique used for the ablation (homogenization of the scar) was the only predictor of a better outcome.

Study name or acronym; Author; Year published; PMID	Aim of study; Endpoints; Study type (RCT, observational — multicenter or single center; case series or other [if RCT include intervention and comparator]); Study size (N)	Patient population with inclusion and exclusion criteria	Results (absolute event rates, *P* values; OR or RR; 95% CI)	Other relevant findings or adverse events	Limitations; Other comments; Conclusions
Arenal et al. Safety, long-term results, and predictors of recurrence after complete endocardial ventricular tachycardia substrate ablation in patients with previous myocardial infarction.<br>• Year published: 2013<br>• PMID: 23228925	Aim: To assess the safety, efficacy, and predictors of VT recurrence after ablation of all conduction channels, isolated components, and LPs (CEVTSA).<br><br>The local end points were disappearance of isolated components, LPs, CC electrograms, or absence of local capture at 10 mA.<br><br>Study type: Cohort<br><br>Study size (N): 59 patients	Consecutive patients referred for ablation of SMVT from January 2004 to December 2008. The patients fulfilled the following inclusion criteria: (1) chronic MI; (2) documented SMVT by ICD stored electrograms or ECGs; (3) complete endocardial LV mapping during SR or RV pacing; and (4) intention to target all isolated components, LPs, and CCs.	Age, 66 ± 10 years<br>Male sex, 54 (91%)<br>Mean LVEF, 29% ± 10%<br>The mean areas of scar (≤1.5 mV) and dense scar (≤0.5 mV) were 76 ± 42 and 34 ± 24 cm^2, respectively. Isolated components/LPs, and conduction channels were identified and ablated in 97% and 83% of patients, respectively (mean ablation area 14 ± 10 cm^2). No life-threatening complications occurred during the procedure.<br>The LVEF did not decrease after ablation (29% ± 10% vs 31% ± 11%, *P*=NS). Only 34% of patients were discharged with AADs. Univariate analysis identified the LVEF, VT CL, infarct location (inferior vs anterior), and dense scar area as predictors of VT recurrence, and Cox analysis identified VT CL (HR 0.42; *P*<.001) and dense scar area (HR 2.65, *P*<.0006) as independent predictors.	After 1 year, 81% and 86% of the patients were free from VT recurrences and ICD shocks, respectively; at the end of follow-up, these percentages had decreased to 58% and 64%, respectively (39 ± 21 months).	Limitations of this study include that patients were not randomized to different ablation strategies; thus, it was not possible to establish the superiority of scar homogenization over a standard procedure. In addition, all isolated component/LP sites were not ablated in 20% of patients.<br><br>Other comments and conclusions:<br>- This single-center study showed that (1) CEVTSA is safe and effective, even in patients without clinical VT references; (2) scar extension and VT CL are the main predictors of VT recurrence; and (3) patients with dense scar ≤25 cm^2 and VT CLs >350 ms have a good prognosis.<br>- In contrast to previous studies, in which most patients were receiving AADs, only 34% of our patients were discharged with AADs. Long-term mortality (22%) was similar to the mortality in previous ablation studies.

Study name or acronym; Author; Year published; PMID	Aim of study; Endpoints; Study type (RCT, observational — multicenter or single center; case series or other [if RCT include intervention and comparator]); Study size (N)	Patient population with inclusion and exclusion criteria	Results (absolute event rates, *P* values; OR or RR; 95% CI)	Other relevant findings or adverse events	Limitations; Other comments; Conclusions
Arenal et al. Ablation of electrograms with an isolated, delayed component as treatment of unmappable monomorphic ventricular tachycardias in patients with structural heart disease.<br>• Year published: 2003<br>• PMID: 12570949	Aim: The purpose of this study was to assess (1) the incidence, location, and extension of E-IDCs in patients with clinical SMVTs and SHD; (2) the feasibility of relating E-IDC to documented clinical SMVT; and (3) the efficacy of E-IDC ablation as treatment for noninducible and/or nontolerated VT.<br><br>The endpoints of the procedure were (1) disappearance of all IDCs of a clinical VT-related area in those patients with unmappable VT; and (2) inducibility suppression of clinical VT.<br><br>Study type: Cohort<br><br>Study size (N): 24 patients	Some 24 of 38 consecutive patients with SHD and SMVT documented by a 12-lead ECG who were referred for RFCA.	Some 21 of 24 patients with ICM, 2 of 24 with NICM, and 1 with ToF.<br><br>Twelve patients had an ICD.<br><br>Conventional activation mapping was not possible in 18 patients: at least 1 of the clinical VTs or the clinical VT was not inducible in 12 patients, and VT was not tolerated in 6 patients. This group had experienced between 1 and 106 VT episodes in the month before the ablation procedure.<br><br>The E-IDC was detected in 12 of 22 patients during SR and in 23 of 24 patients during RVA pacing *P*<.01). One area of E-IDC was recorded in 20 patients, and 2 or more were recorded in 4 patients.<br><br>Extension of the E-IDC area (3.5 ± 2.6 cm^2 [range	Ablation guided by E-IDC suppressed all but one clinical VT whose inducibility suppression was tested.<br><br>During a follow-up period of 9 ± 4 months, 3 patients had recurrences of the ablated VT and 2 of a different VT.	Limitations:<br>- In patients with inducible and nontolerated VT, it was not possible to differentiate between the E-IDC recorded in the central common pathway of the circuit from those recorded in areas probably connected but not related to the VT circuit, as an adjacent bystander. Nevertheless, isolated potentials are usually recorded near the central common pathway. In a few patients with more than one clinical VT, not all VTs could be related to the E-IDC areas; a nonendocardial circuit and the complexity of some circuits in which several exit sites coexist might preclude the reproduction of certain morphologies.<br>- This treatment strategy has not proven its efficacy in patients with nontolerated and undocumented VTs, mainly when these patients have more than one E-IDC area. In patients with a low frequency of episodes, it is not possible to determine the long-term efficacy of the procedure.

Study name or acronym; Author; Year published; PMID	Aim of study; Endpoints; Study type (RCT, observational — multicenter or single center; case series or other [if RCT include intervention and comparator]); Study size (N)	Patient population with inclusion and exclusion criteria	Results (absolute event rates, *P* values; OR or RR; 95% CI)	Other relevant findings or adverse events	Limitations; Other comments; Conclusions
			1–12]) was smaller than the area with a voltage ≤0.5 mV, including complete and dense scar (22 ± 11 cm^2 [range 11–50]; *P*<.01). The E-IDCs were recorded close to the border but inside the dense scar.<br><br>From a total of 29 E-IDC areas, 24 were related to documented clinical VTs.		- A reduction in ventricular function was not systematically evaluated; nevertheless, no clinical deterioration related to the procedure was observed.<br>Other comments and conclusions:<br>- It is important to note that RVA pacing permitted the identification of the VT substrate in several patients in whom the SR map was not sensitive enough, supporting our hypothesis that a change in the direction of the activation front might unmask some areas of block and slow conduction.<br>- The E-IDC areas are relatively small compared with scar areas; therefore, this method permits a focus on the diagnostic techniques and ablation in defined areas.<br>- This procedure allows the ablation of some nontolerated VTs not approachable by conventional entrainment mapping and could also eliminate the substrate for different VTs otherwise not ablated by limited conventional strategies.

Study name or acronym; Author; Year published; PMID	Aim of study; Endpoints; Study type (RCT, observational — multicenter or single center; case series or other [if RCT include intervention and comparator]); Study size (N)	Patient population with inclusion and exclusion criteria	Results (absolute event rates, *P* values; OR or RR; 95% CI)	Other relevant findings or adverse events	Limitations; Other comments; Conclusions
Berruezo et al. Combined endocardial and epicardial catheter ablation in arrhythmogenic right ventricular dysplasia incorporating scar dechanneling technique.<br>• Year published: 2012<br>• PMID: 22205683	Aim: The objective of this single-center prospective study was to analyze the short- and long-term results of a combined ENDO and EPI catheter ablation incorporating the scar dechanneling technique as a first-line therapy in patients with ARVD/C.<br><br>The end point of the ablation procedure was the elimination of all identified CCs (scar dechanneling) and the abolition of all inducible VTs.<br><br>Study type: Cohort<br><br>Study size (N): 11 patients	All patients with ARVD/C diagnosed on the basis of 2010 task force criteria and presenting with a clinical SVT episode were included in the study.	Mean age 42 ± 13 years<br>Male sex, 82%<br>Mean LVEF, 55% ±7%<br>A high-density 3D ENDO (321 ± 93 sites mapped) and EPI (302 ± 158 sites mapped) electroanatomical voltage map was obtained during SR to define scar areas (<1.5 mV) and CCs inside the scars, between scars, or between the TA and a scar. The mean surface scar area was 3 times lower at the endocardium than at the epicardium (26 ± 18 vs 95 ± 45 cm^2; *P*<.01). Individually, ENDO surface scar area was never larger than EPI scar area. The CCs were identified more frequently by annotating E-IDCs (32 LP channels) than with voltage scanning (10 voltage channels). Intrascar CCs represented 66% of all CCs, followed by 24% being interscar CCs and 10% related to the TA. The mean number of CCs per patient was 3 ± 2, with 78% being EPI.	The combined ENDO and EPI VT ablation eliminated all clinical and induced VTs, and the addition of scar dechanneling resulted in noninducibility in all cases.<br><br>During a median follow-up of 11 months (6–24 months), only 1 (9%) patient had a VT recurrence. There was a single major bleeding event that did not preclude a successful procedure.	Limitations:<br>- The short-term efficacy of scar dechanneling cannot be calculated individually because a complete stimulation protocol was not repeated after clinical or induced VT ablation.<br>- The possibility exists that some CCs do not participate in VTs during the stimulation protocol but do activate during follow-up. Thus, the only possibility to determine the proportionate advantage of scar dechanneling over a combined ENDO and EPI strategy for clinical and inducible VT ablation would be to perform a larger, randomized, follow-up study.<br>Other comments and conclusions:<br>combined ENDO and EPI mapping reveals a wider EPI VT substrate in patients with ARVD/C with clinical VTs. As a first-line therapy, combined ENDO and EPI VT ablation incorporating scar dechanneling achieves a very good short- and midterm success rate.

Study name or acronym; Author; Year published; PMID	Aim of study; Endpoints; Study type (RCT, observational — multicenter or single center; case series or other [if RCT include intervention and comparator]); Study size (N)	Patient population with inclusion and exclusion criteria	Results (absolute event rates, *P* values; OR or RR; 95% CI)	Other relevant findings or adverse events	Limitations; Other comments; Conclusions
Berruezo et al. Scar dechanneling: new method for scar-related left ventricular tachycardia substrate ablation.<br>• Year published: 2015<br>• PMID: 25583983	Aim: To assess whether scar dechanneling is a feasible first step (before VT induction and ablation attempt) for patients with LV scar-related VT, and whether it can result in less extensive ablation and no VA during the procedure.<br><br>Procedure workflow:<br>1. Substrate mapping - CC entrance identification<br>2. CC elimination<br>3. Remapping residual CC ablation<br>4. Residual VT ablation inducibility<br><br>The procedural endpoint was the elimination of all identified CCs by ablation at the CC entrance followed by abolition of residual inducible VTs.<br><br>Study type: Cohort<br><br>Study size (N): 101 patients	Inclusion criteria: Presence of SHD (previous MI, LV dilatation/systolic dysfunction, or normal LV diameters/systolic function with evidence of ventricular scar on CE-CMR or EAM) and SMVT documented by 12-lead ECG or ICD electrograms.<br><br>Exclusion criteria: Patients with VAs caused by reversible causes.	Age, 65 ± 12 years<br><br>Male sex, 92 (91.1%)<br><br>LVEF %, 36 ± 13<br><br>AADs: BBs, 81 (80.2%); Class I, 8 (7.9%); Class III, 55 (54.5%).<br><br>Complete scar dechanneling could be achieved in 85 (84.2%) patients.<br><br>Patients needing only scar dechanneling had a shorter procedure (213 ± 64 vs 244 ± 71 minutes; *P*=.027), fewer RF applications (19% ± 11% versus 27% ± 18%; *P*=.01), and external cardioversion/defibrillation shocks (20% vs 65.2%; *P*<.001).<br><br>Univariate analysis showed 4 predictors of the primary end point: VT inducibility after scar dechanneling (HR 2.58,	Complete scar dechanneling rendered noninducibility in 54.5% of patients; ablation of residual inducible VT increased noninducibility to 78.2%.<br><br>Despite having no significant differences in LVEF and myocardial scar area, patients needing only scar dechanneling had fewer endocardial electrograms with delayed components (36 ± 29 vs 50 ± 32; *P*=.04), required fewer RF applications (19 ± 11 vs 27 ± 18; *P*=.01), and less frequently had incomplete CC-electrogram elimination (7.3% vs 26.1%; *P*=.01).<br><br>Fewer complications occurred in patients rendered noninducible by scar dechanneling alone than in patients requiring residual VT ablation (1.8% vs 13%, respectively; *P*=.027).<br><br>After a median (IQR) follow-up of 21 (11–29) months,	Limitations:<br>- This study could not determine whether some of the benefits observed—lower procedure requirements, fewer complications, and better outcomes for patients needing only the scar dechanneling technique—are exclusively because of substrate ablation as the first step during the procedure or involve other conditions that permit the use of this strategy (ie, the absence of incessant VT) or uncontrolled determinant factors not taken into account. Improved outcomes observed in patients for whom scar dechanneling alone achieved noninducibility suggest that this subgroup had a more accessible substrate. A small proportion of patients with clinical VTs could be noninducible before ablation; the lack of a basal induction protocol limits the evaluation of the acute effect of substrate ablation.<br>- Because substrate mapping

Study name or acronym; Author; Year published; PMID	Aim of study; Endpoints; Study type (RCT, observational — multicenter or single center; case series or other [if RCT include intervention and comparator]); Study size (N)	Patient population with inclusion and exclusion criteria	Results (absolute event rates, *P* values; OR or RR; 95% CI)	Other relevant findings or adverse events	Limitations; Other comments; Conclusions
			1.18–5.67; *P*=.018), VT inducibility after residual VT ablation (HR 2.59, 1.18–5.68; *P*=.018), epicardial mapping (HR 0.16, 0.05–0.57; *P*=.004), and incomplete CC-electrogram elimination (HR 3.50, 1.55–7.87; *P*=.003).<br>Under multivariable analysis, the only independent predictor of any sustained ventricular arrhythmia episode or SCD was incomplete CC-electrogram elimination (HR 2.54, 1.06–6.10; *P*=.037). Higher endpoint-free survival rates were observed in patients noninducible after scar dechanneling (log-rank *P*=.013) and those with complete CC-electrogram elimination (log-rank *P*=.013). The complications rate was 6.9%, with no deaths.	recurrences of sustained VA episode or SCD were observed in 27 (26.7%) patients.<br><br>At 2 years, patients needing scar dechanneling alone had better event-free survival (80% vs 62%) and lower mortality (5% vs 11%). Survival curve analysis showed higher endpoint-free survival for patients with complete CC-electrogram elimination (log-rank test *P*=.001).	was performed during intrinsic rhythm in the vast majority of patients, the effect was not analyzed of different activation wavefronts (ie, intrinsic rhythm versus paced rhythm) on CC entrance location and characteristics.<br><br>Other comments and conclusions:<br>- This study shows that substrate ablation with the scar dechanneling technique as the first step of the ablation protocol (before any VT induction/ablation attempt) renders more than half of the patients with LV scar-related VTs noninducible. These patients can benefit from starting and finishing the procedure in SR, with lower procedural requirements (procedure duration, RF delivery), fewer complications, no hemodynamic instability, and a much lower chance of direct current cardioversion or defibrillation.

Study name or acronym; Author; Year published; PMID	Aim of study; Endpoints; Study type (RCT, observational — multicenter or single center; case series or other [if RCT include intervention and comparator]); Study size (N)	Patient population with inclusion and exclusion criteria	Results (absolute event rates, *P* values; OR or RR; 95% CI)	Other relevant findings or adverse events	Limitations; Other comments; Conclusions
					- In the long term, a patient subgroup (ie, noninducible after scar dechanneling) is identified that, despite a similar clinical profile (age, VT CL, LVEF, scar size), have a lower probability of VT recurrences or sudden death (16.4% vs 37%, *P*=.018) and lower mortality (3.6% vs 15.2%, *P*=.042), compared with patients needing residual inducible VT ablation. The differences in procedure requirements and better outcomes likely reflect that patients needing scar dechanneling alone have a more accessible substrate.<br>- 82% of patients undergoing a redo procedure required epicardial ablation.

Study name or acronym; Author; Year published; PMID	Aim of study; Endpoints; Study type (RCT, observational — multicenter or single center; case series or other [if RCT include intervention and comparator]); Study size (N)	Patient population with inclusion and exclusion criteria	Results (absolute event rates, *P* values; OR or RR; 95% CI)	Other relevant findings or adverse events	Limitations; Other comments; Conclusions
Jaïs et al. Elimination of local abnormal ventricular activities: a new end point for substrate modification in patients with scar-related ventricular tachycardia.<br>• Year published: 2012<br>• PMID: 22492578	Aim: To verify whether the elimination of LAVAs during sinus rhythm or ventricular pacing would be a useful and effective endpoint for substrate-based VT ablation.<br><br>Endpoints: Elimination of LAVAs during SR or ventricular pacing substrate-based VT ablation.<br><br>Study type: Cohort<br><br>Study size (N): 70 patients	Inclusion criteria: Documented episodes of repetitive SVT resistant to AAD therapy and requiring external cardioversion or ICD antitachycardia pacing or shocks, and SHD with ischemic or nonischemic dilated cardiomyopathy.<br><br>Patients were excluded if VAs were attributable to an acute or reversible cause. Patients with repetitive PVCs or NSVT in the absence of SVT were also excluded.	Age, 67 ± 11 years<br><br>Male sex, 63 (90%)<br><br>Conventional mapping was performed in SR for all.<br><br>LAVAs were recorded in 67 patients (95.7%; 95% CI 89.2–98.9). Endocardial and epicardial LAVAs were present in 63 of 70 (90%) patients and in 17 of 21 (81%) patients, respectively.<br><br>No baseline clinical characteristic, including age, EF, and type of cardiomyopathy, was predictive of LAVA elimination.	LAVAs were successfully abolished or dissociated in 47 of 67 patients (70.1%; 95% CI 58.7– 80.1).<br><br>Patients were followed up for a median of 22 months (IQR, 14–27 months) from the initial ablation procedure. The combined endpoint of VT recurrence or death occurred in 39 patients (55.7%; 95% CI 44.0–66.8), with recurrent VT in 32 (46%) and death in 13 (19%). The combined endpoint occurred in 21 of 47 patients (45%) with LAVA elimination, with recurrent VT in 15 (32%) and death in 9 (19%). In contrast, the combined endpoint occurred in 16 of 20 (80%) patients without LAVA elimination, with recurrent VT in 15 (75%) and death in 4 (20%).<br><br>A redo procedure was performed in 14 patients, 6 of whom had LAVAs successfully eliminated during the first intervention	Limitations:<br>- Complete elimination of LAVAs was confirmed in 70.1% of patients in whom they had been documented. Interestingly, procedures associated with LAVA elimination had significantly longer RF ablation times.<br>- In a minority of patients, entrainment and EAM were also performed.<br>- The study was not designed to determine the optimal density of maps nor the role of adjunctive mapping techniques in targeting and eliminating LAVA. A potential disadvantage of this strategy could be greater tissue destruction than required to achieve clinical success. However, the postablation LVEF remained unchanged, suggesting that the ablation of surviving cells in scar areas was not deleterious.<br><br>Other comments and conclusions:<br>- The main findings are the following:

Study name or acronym; Author; Year published; PMID	Aim of study; Endpoints; Study type (RCT, observational — multicenter or single center; case series or other [if RCT include intervention and comparator]); Study size (N)	Patient population with inclusion and exclusion criteria	Results (absolute event rates, *P* values; OR or RR; 95% CI)	Other relevant findings or adverse events	Limitations; Other comments; Conclusions
				and 8 of whom had persistent LAVAs. At last follow-up, 62.7% of patients remained free of VT recurrence after the last ablation procedure.<br><br>In the multivariate analysis, LAVA elimination was independently associated with a reduction in recurrent VT or death (HR 0.49; 95% CI 0.26–0.95; *P*=.035) during long-term follow-up (median, 22 months). Noninducibility (*P*=.11) and ischemic vs nonischemic heart disease (*P*=.29) were not predictive of VT-free survival.	(1) LAVAs are observed in the majority of patients with ischemic or dilated cardiomyopathy;<br>(2) LAVAs can be eliminated or dissociated by catheter ablation in SR;<br>(3) Ablation of LAVAs appears reasonably safe;<br>(4) LAVA elimination is a clear procedural endpoint;<br>(5) Complete elimination of LAVAs is associated with a superior clinical outcome.<br>- Complete elimination of LAVAs was confirmed in 70.1% of patients in whom they had been documented. Interestingly, procedures associated with LAVA elimination had significantly longer RF ablation times.

Study name or acronym; Author; Year published; PMID	Aim of study; Endpoints; Study type (RCT, observational — multicenter or single center; case series or other [if RCT include intervention and comparator]); Study size (N)	Patient population with inclusion and exclusion criteria	Results (absolute event rates, *P* values; OR or RR; 95% CI)	Other relevant findings or adverse events	Limitations; Other comments; Conclusions
Marchlinski et al. Linear ablation lesions for control of unmappable ventricular tachycardia in patients with ischemic and nonischemic cardiomyopathy.<br>• Year published: 2000<br>• PMID: 10725289	Aim: To demonstrate that in patients with unmappable, unimorphic VT that (1) the abnormal endocardium can be defined using detailed SR voltage mapping; and (2) linear ablation lesions that repeatedly and/or selectively interrupt the border zone of abnormal endocardium could control VT.<br><br>Study type: Cohort<br><br>Study size (N): 16 patients	Patients who had drug refractory, unimorphic, unmappable VT.<br><br>The 16 patients came from a pool of 48 patients who were referred to our hospital for catheter ablation.	A total of 12 men and 4 women; 9 patients had ICM, and 7 patients NICM. All patients had implantable defibrillators.<br>A total of 15 patients were also treated with AADs during the study.<br>The amount of endocardium demonstrating an abnormal electrogram amplitude ranged from 25 to 127 cm^2.<br>A total of 8 to 87 RF lesions (mean, 55) produced a median of 4 linear lesions that had an average length of 3.9 cm (range, 1.4–9.4 cm).<br>The total procedure and fluoroscopy time ranged from 6.0–13.5 hours (mean, 8.8 ± 1.9 hours) and from 60–196 minutes (mean, 121 ± 38 minutes), respectively, and were greater for patients with VT who had NICM rather than ICM (10.8 ± 2.1 vs 8.1 ± 2.1 hours; *P*<.05) and total fluoroscopy time (156 ± 32 vs 90 ± 27 minutes; *P*=.01), respectively.	Twelve (75%) patients have been free of VT during 3 to 36 months of follow-up (median, 8 months); 4 patients had VT episodes at 1, 3, 9, and 13 months, respectively. Only one of these patients had frequent VT.	Limitations:<br>- Because of the variability in the frequency of VT, it is difficult to ascertain whether a good clinical response is causally related to any ablation procedure.<br>- Average high duration of the procedure and the duration of fluoroscopic exposure.<br><br>Other comments and conclusions:<br>The study documents that (1) the extent and voltage characteristics of the abnormal endocardium can be defined using SR, bipolar electrogram, and voltage mapping; and (2) that catheter-based ablative therapy that creates linear lesions targeting the border zone can control recurrent VT.

Study name or acronym; Author; Year published; PMID	Aim of study; Endpoints; Study type (RCT, observational — multicenter or single center; case series or other [if RCT include intervention and comparator]); Study size (N)	Patient population with inclusion and exclusion criteria	Results (absolute event rates, *P* values; OR or RR; 95% CI)	Other relevant findings or adverse events	Limitations; Other comments; Conclusions
Nogami et al. Changes in the isolated delayed component as an endpoint of catheter ablation in arrhythmogenic right ventricular cardiomyopathy: predictor for long-term success.<br>• Year published: 2008<br>• PMID: 18284499	Aim: To assess the usefulness of a change in the IDC as an endpoint of the catheter ablation in ARVC.<br><br>Endpoints: Disappearance of all the IDCs of the VT-related area and noninducibility of any VT.<br><br>Study type: Cohort<br><br>Study size (N): 18 patients	Consecutive patients with ARVC who were admitted to our hospital between 1995 and 2005. All patients had SMVT and were diagnosed with ARVC according to the criteria proposed by the international study group on ARVC.	Mean age 48 ± 11 years; 5 women and 13 men.<br><br>IDCs were recorded in 16 patients and the latest IDCs were related to the VT circuit. Catheter ablation was performed in the areas with the IDCs. At the end of the session, the IDC was electrically dissociated in 1, disappeared in 5, exhibited second-degree block in 1, was significantly delayed (≥50 milliseconds) in 3, and remained unchanged in 6. The change in the IDC was correlated with the change in the type II/III LPs in the signal-averaged ECG and in the inducibility of the clinical VT after the ablation.	During a follow-up of 61 ± 38 months, VT recurred in 6. The patients with a changed IDC had a significantly lower VT recurrence than those with no IDC or an unchanged IDC (*P*<.02).	Limitations: Results are not based on a controlled comparison with a different ablation technique, and the number of patients is small. In patients with nontolerated VT, it is not possible to confirm the relationship between the IDC and VT circuit by entrainment. Even if pace mapping reproduced the QRS morphology of the VT, there was a possibility of a bystander. Because epicardial mapping was not performed, an IDC at the critical isthmus might have been recorded from the epicardium in the unsuccessful cases.<br>Other comments and conclusions: Detailed endocardial mapping of the right ventricle was performed during SR. All AADs except amiodarone were discontinued for at least 5 half-lives. In patients with ARVC, (1) the IDCs during SR are related to the clinical VT and can be a target for the ablation; (2) a change in the IDC can be used as an endpoint; and (3) qualitative analyses of the serial signal-averaged ECGs might be useful for the long-term follow-up.

Study name or acronym; Author; Year published; PMID	Aim of study; Endpoints; Study type (RCT, observational — multicenter or single center; case series or other [if RCT include intervention and comparator]); Study size (N)	Patient population with inclusion and exclusion criteria	Results (absolute event rates, *P* values; OR or RR; 95% CI)	Other relevant findings or adverse events	Limitations; Other comments; Conclusions
Soejima et al. Catheter ablation in patients with multiple and unstable ventricular tachycardias after myocardial infarction: short ablation lines guided by reentry circuit isthmuses and sinus rhythm mapping.<br>• Year published: 2001<br>• PMID: 11489772	Aim: The purpose of this study is to (1) assess the feasibility of guiding RF line placement by limited mapping to attempt to identify reentry circuit isthmuses; (2) relate the length of RF ablation lines to the effect on inducible VTs; and (3) determine whether the presence of multiple morphologies of inducible VT and unstable VTs reduces the efficacy of these approaches.<br><br>The procedural endpoint was reached when no monomorphic VT was inducible.<br><br>Study type: Cohort<br><br>Study size (N): 40 patients	Between October 1997 and June 2000, a total of 60 patients were referred for RF ablation of MMVT associated with MI. Patients with multiple morphologies of VT or unstable VT were not excluded.	Age 66.6 ± 10.9 years<br><br>Male sex, 38 (40%)<br><br>MI location: inferior, 28; anterior, 7; and both, 5<br><br>A total of 143 VTs (42 stable, 101 unstable) were induced.<br><br>An isthmus was identified in 25 patients (63%; 5 with only stable VTs, 5 with only unstable VTs, and 15 with both VTs).	Inducible VTs were abolished or modified in 100% of patients when the RF line included an isthmus compared with 53% when RF had to be guided by pace mapping (*P*=.0002); those with an isthmus identified received shorter ablation lines (4.9 ± 2.4 vs 7.4 ± 4.3 cm total length, *P*=.02).<br><br>During follow-up, spontaneous VT decreased markedly regardless of whether an isthmus was identified. VT stability and number of morphologies did not influence outcome.	Limitations:<br>- This study was not a randomized comparison of RF line length or RF line placement.<br>- The minimum line length required was not determined.<br><br>Other comments and conclusions:<br>a 4- to 5-cm line of RF lesions abolishes all inducible VTs in more than 50% of patients. Less ablation is required if a reentry circuit isthmus is identified even when multiple and unstable VTs are present.

Study name or acronym; Author; Year published; PMID	Aim of study; Endpoints; Study type (RCT, observational — multicenter or single center; case series or other [if RCT include intervention and comparator]); Study size (N)	Patient population with inclusion and exclusion criteria	Results (absolute event rates, *P* values; OR or RR; 95% CI)	Other relevant findings or adverse events	Limitations; Other comments; Conclusions
Tung et al. Impact of local ablation on interconnected channels within ventricular scar: mechanistic implications for substrate modification.<br>• Year published: 2013<br>• PMID: 24162832	Aim: To evaluate the impact of local ablation of LPs on adjacent and remote areas of slow conduction with simultaneous multipolar mapping.<br><br>Acute procedural success was defined as noninducibility after ablation. Partial success was defined as inducibility of only faster nonclinical VTs.<br><br>Study type: Cohort<br><br>Study size (N): 21 patients	Consecutive patients referred for ablation of scar-mediated VT with double ventricular access using a multipolar catheter and ablation catheter.	21 patients (ICM=15, NICM=2, ARVD=1, sarcoid=1, Chagas=1, noncompaction=1) underwent double ventricular access for VT ablation using a multipolar catheter with the intention of monitoring RF application effects within scar. The median age was 63 (52–70) years and all patients were male. The median ejection fraction was 25% (25–30%). Approximately half (48%) of patients referred had prior ablation and 90% were on antiarrhythmic medications.	In 21 patients, a multipolar catheter placed within scar visualized spatially distinct LPs. Among 39 RF applications, ablation at earlier LPs had an effect on neighboring and remote LPs in 31 (80%), with delay in 8 (21%), partial elimination in 9 (23%), and complete elimination in 14 (36%). The mean distance where an ablation impact was detected was 17.6 ± 14.7mm (range 2 mm–50 mm). Among all patients, 9.7 ± 7.8 RF applications were delivered to homogenize the targeted scar region with a mean number of 23 ± 12 LPs targeted.	Limitations:<br>- The observed interconnectedness of channels within scar might not be generalizable to all scar substrates because only patients in whom multiple potentials are recorded at a single catheter position were included. Multiple causes of scar were included in this cohort, and the small sample size is inadequate to draw conclusions across all substrates.<br>- Only electrograms with a gradient of late activation were chosen, which biases the ablation lesions toward orientation along a channel. However, the absence of remote impact detected during local ablation does not exclude effects on regions that might not be contacted by the electrodes on the multipolar catheter.<br>- Different multipolar catheters were used within this cohort, and the use of smaller interelectrode spacing might increase the

Study name or acronym; Author; Year published; PMID	Aim of study; Endpoints; Study type (RCT, observational — multicenter or single center; case series or other [if RCT include intervention and comparator]); Study size (N)	Patient population with inclusion and exclusion criteria	Results (absolute event rates, *P* values; OR or RR; 95% CI)	Other relevant findings or adverse events	Limitations; Other comments; Conclusions
					ability to detect ablation impact within scar.<br><br>Other comments and conclusions:<br>- Ablation can eliminate neighboring and remote areas of slow conduction, suggesting that channels within scar are frequently interconnected.<br>- This is the first mechanistic demonstration to show that ablation can modify electrical activity in regions of scar outside of the known radius of a RF lesion. The targeting of relatively earlier LPs can expedite scar homogenization without the need for extensive ablation of all LPs.

Study name or acronym; Author; Year published; PMID	Aim of study; Endpoints; Study type (RCT, observational — multicenter or single center; case series or other [if RCT include intervention and comparator]); Study size (N)	Patient population with inclusion and exclusion criteria	Results (absolute event rates, *P* values; OR or RR; 95% CI)	Other relevant findings or adverse events	Limitations; Other comments; Conclusions
Tzou et al. Core isolation of critical arrhythmia elements for treatment of multiple scar-based ventricular tachycardias.<br>• Year published: 2015<br>• PMID: 25681389	Aim: To investigate a unique ablation endpoint among patients with VT in the setting of SHD by (1) identifying critical or core VT circuit elements based on careful electrophysiological characterization; and (2) ablating these areas circumferentially with the goal of achieving electric isolation. CI thus refers to circumferential ablation around all critical VT circuit elements.<br><br>Endpoints:<br>- Identifying critical or core VT circuit elements based on careful electrophysiological characterization.<br>- Ablating these areas circumferentially with the goal of achieving electric isolation.<br><br>Study type: Cohort<br><br>Study size (N): 44 patients	Patients referred for VT ablation to the University of Pennsylvania and the University of Colorado Health Systems between January 2011 and November 2013 were evaluated. All included patients had SHD and VT that was refractory to AAD therapy.<br><br>Exclusion criteria included <18 years of age, lack of LVAs as determined by bipolar voltage mapping, inability or refusal to provide informed consent, or individual operator decision before starting the case not to attempt CI as the ablation strategy.	Age, 63 ± 14 years<br><br>Male sex, 42 (95%)<br><br>Type of SHD: ICM 32 (73%), dilated/idiopathic cardiomyopathy 4 (9%), other 8 (18%).<br><br>LVEF %, 31 ± 13<br><br>Some 68% had multiple unstable VTs (mean, 3 ± 2)<br><br>More than half had undergone prior VT ablation.<br><br>The only characteristic that was significantly associated with long-term VT recurrence in univariate analysis was achievement of CI (HR 0.17; *P*=.03).	The CI area was 11 ± 12 vs 55 ± 40 cm^2 total scar area.<br><br>Additional substrate modification was performed in 27 (61%), and epicardial RF ablation was performed in 4 (9%) patients.<br><br>CI was achieved in 37 (84%). Notably, CI could be achieved in 11 of the 12 (92%) patients with a nonischemic VT substrate.<br><br>Kaplan–Meier survival analysis demonstrated that those in whom CI was successfully achieved had significantly better long-term VT-free survival compared with those in whom CI was not successful (log-rank *P*=.013).	Limitations:<br>- Observational study and lacked a control group. Sample size was relatively small (limiting interpretation of multivariable analysis).<br>- Patient selection for CI was based on operator preference (possible selection bias).<br>- Only patients with arrhythmogenic substrate identified by bipolar voltage mapping were included; this strategy thus cannot be extended to those with purely intramural myocardial substrate in which bipolar voltage might appear normal.<br>- Because of patient safety or length of procedure, reassessment of durable CI after a waiting period was not performed.<br>- Additional substrate modification, occasionally including epicardial ablation, was performed in many patients because of either continued VT inducibility or operator preference to reinforce lesion sets. There is inevitable overlap in regions

Study name or acronym; Author; Year published; PMID	Aim of study; Endpoints; Study type (RCT, observational — multicenter or single center; case series or other [if RCT include intervention and comparator]); Study size (N)	Patient population with inclusion and exclusion criteria	Results (absolute event rates, *P* values; OR or RR; 95% CI)	Other relevant findings or adverse events	Limitations; Other comments; Conclusions
					that would be targeted using other recently reported approaches for VT ablation, although the approach investigated could have been more limited in proportionate area of ablation.<br><br>Other comments and conclusions:<br>- CI is a novel strategy with a discrete and measurable endpoint beyond VT inducibility to treat patients with multiple or unmappable VTs.<br>- The CI region can be selected based on standard characterization of suspected VT isthmus surrogates, thus limiting ablation target size. Exit block within the isolated area is achievable in most and may further improve long-term success.

Study name or acronym; Author; Year published; PMID	Aim of study; Endpoints; Study type (RCT, observational — multicenter or single center; case series or other [if RCT include intervention and comparator]); Study size (N)	Patient population with inclusion and exclusion criteria	Results (absolute event rates, *P* values; OR or RR; 95% CI)	Other relevant findings or adverse events	Limitations; Other comments; Conclusions
Calkins et al. Catheter ablation of ventricular tachycardia in patients with structural heart disease using cooled radiofrequency energy: results of a prospective multicenter study. Cooled RF Multi Center Investigators Group.<br>• Year published: 2000<br>• PMID: 10841242	Aim: To evaluate the safety and efficacy of an RFCA system with internal saline irrigation.<br>Endpoints: Elimination of all mappable VTs.<br>Prospective multicenter study<br>- At the onset of the study, patients were randomized 1:1 between ablation and continued antiarrhythmic therapy. As the study progressed, the enrollment criteria were altered to facilitate enrollment, including elimination of randomization and the requirement for an ICD.<br>N= 146: 63 patients who were randomized to catheter ablation; 17 patients who were randomized to medical therapy and underwent catheter ablation after failure of medical therapy; 13 patients who received ablation under compassionate use; and 53 patients who were enrolled in the study after the randomization eliminated.	Inclusion criteria:<br>(1) Documented SMVT with 2 or more episodes in the 2 months before enrollment;<br>(2) Spontaneous VT that was hemodynamically stable;<br>(3) VT due to IHD;<br>(4) An ICD device with electrogram storage;<br>(5) Failure of at least 2 AADs.	Catheter ablation was acutely successful in 106 patients (75%) as defined by elimination of all mappable VTs; in 59 patients (41%), no VT of any type was inducible after ablation.<br><br>The duration of follow-up was 243 ± 153 days.<br><br>After catheter ablation, 66 (46%) patients developed 1 or more episodes of a sustained VA; the median time to VT recurrence was 24 days; the 1-year recurrence rate was 56%.	Twelve (8%) patients experienced a major complication: 4 (2.7%) strokes, 4 pericardial tamponades (2.7%), 2 complete AV block, 1 valve injury, and 1 MI. Four of the major complications led to a patient death (2.7%).<br>- LMT embolus in the subtherapeutical ACT<br>- ICM patient after tamponade from left atrial appendage<br>- Cerebrovascular accident<br>- Aortic valve injury in ICM patient	Conclusions:<br>This study defines the efficacy and risks of catheter ablation for patients with recurrent episodes of VT. Prevention of further spontaneous episodes of VT was achieved in 54% of patients. This efficacy must be balanced against the risk of procedure complications.<br><br>Limitations:<br>High incidence of complications could in part be attributable to an unequal distribution of skills.<br>This study was not randomized.

Stevenson et al. Irrigated radiofrequency catheter ablation guided by electroanatomic mapping for recurrent ventricular tachycardia after myocardial infarction: the multicenter thermocool ventricular tachycardia ablation trial.<br>• Year published: 2008<br>• PMID: 19064682	Aim: To evaluate the safety and efficacy of an RF ablation catheter with external irrigation combined with an EAM system for ablation of recurrent VT caused by prior MI.<br><br>Endpoints: No recurrence of sustained MMVT or incessant VT at 6 months of follow-up.<br><br>Observational multicenter study<br><br>18 centers; N=231.	Inclusion criteria:<br>- Sustained monomorphic VT requiring termination by cardioversion or AAD administration with more than 3 episodes in the previous 6 months despite an ICD or AAD therapy.<br>- Patients without ICDs were eligible after 2 episodes of sustained VT.<br><br>Exclusion criteria: Serum creatinine 2.5 mg/dL; LVEF <0.10; mobile LV thrombus on echocardiography; absence of vascular access to the LV; disease process likely to limit survival to <12 months; NYHA class IV HF; cardiac surgery within the past 2 months (unless VT was incessant); unstable angina; severe aortic stenosis or mitral regurgitation with a flail leaflet; pregnancy; age <18 years.<br><br>In contrast to many prior reports, patients with multiple VTs, unmappable VT, and a history of prior failed VT ablation were not excluded.	The freedom from VT or incessant VT during 6 months of follow-up: 123 patients(53%).<br><br>Predictors of primary outcome: older; more HF; more AF; multiple MI locations; more inducible VTs; received more RF lesions; more often inducible VTs after ablation.<br><br>In the multivariable analysis, incessant VT was associated with better outcomes (OR .33; *P*=.024), increasing number of inducible VTs (OR 1.28, *P*=.003), a history of HF (OR 2.40, *P*=.015), and a history of AF (OR 2.13, *P*=.05).	Of the 108 ablation failures, 7 (3%) patients died within 7 days of the procedure.<br><br>Of the 24 patients who died within 6 months, 19 had recurrent VT, and 5 died without recurrent VT.<br><br>An increase in the number of VT episodes was observed in 20% of patients.<br><br>At the 1 year follow-up: 40 (18%) patients died; 17 (37.5%) were attributed to VA; 14 (35%) were attributed to HF.<br><br>In 24 (7.3%) patients, significant complications were related to the procedure; HF occurred in 6 patients, who received a median of 1500 mL saline through the irrigated ablation catheter.	Conclusions:<br>Catheter ablation is a reasonable option to reduce episodes of recurrent VT in patients with prior MI. Multiple and unmappable VTs can be targeted with ablation combined with electroanatomic substrate mapping with acceptable risks and outcomes.<br><br>Limitations:<br>- Short follow-up<br>- AADs were often reduced after a successful ablation<br>- Unable to define whether a recurrent VT was related to a targeted VT for ablation

Study name or acronym; Author; Year published; PMID	Aim of study; Endpoints; Study type (RCT, observational — multicenter or single center; case series or other [if RCT include intervention and comparator]); Study size (N)	Patient population with inclusion and exclusion criteria	Results (absolute event rates, *P* values; OR or RR; 95% CI)	Other relevant findings or adverse events	Limitations; Other comments; Conclusions
Sapp et al. Ventricular tachycardia ablation versus escalation of antiarrhythmic drugs.<br>• Year published: 2016<br>• PMID: 27149033	Aim: To compare catheter ablation with escalated AAD therapy in patients with ICM and an ICD who had VT despite first-line AAD therapy.<br><br>Endpoints: The primary outcome was a composite of death occurring at any time after randomization; VT storm (3 or more documented episodes of VT within 24 hours); appropriate ICD shock after a 30-day treatment period. The 30-day treatment period was imposed to exclude nonfatal outcomes that might occur before adequate drug loading or actual performance of catheter ablation.<br><br>A multicenter, randomized, controlled trial; 22 centers.<br><br>N=259 (ablation group, 132 and escalated therapy group, 127; 1:1)	Inclusion criteria: MI, ICD placement; an episode of VT during treatment with amiodarone or another class I or class III AAD within the previous 6 months.<br><br>Episodes of VT were defined as any one of the following: 3 or more episodes of VT treated with antitachycardia pacing, of which at least 1 episode was symptomatic; 1 or more appropriate ICD shocks; 3 or more episodes of VT within 24 hours; SVT at a rate below the programmed detection rate of the ICD. Qualifying episodes of VT were required to be monomorphic and to have rates of less than 250 beats per minute.	The primary outcome occurred in 78 of 132 (59.1%) patients in the ablation group and in 87 of 127 (68.5%) patients in the escalated-therapy group. It was significantly lower in the ablation group than in the escalated therapy group; the HR in the ablation group was 0.72; 95% CI 0.53–0.98; *P*=.04.<br><br>Some 36 (27.3%) patients died in the ablation group and 35 (27.6%) died in the escalated therapy group (HR 0.96; 95% CI 0.60–1.53; *P*=.86).<br><br>VT storm occurred in 32 (24.2%) patients in the ablation group and in 42 (33.1%) patients in the escalated therapy group (HR 0.66; 95% CI 0.42–1.05; *P*=.08).<br><br>Appropriate ICD shocks occurred in 50 (37.9%) patients in	Among the patients in the escalated therapy group, 3 deaths were attributed to AAD therapy (2 from pulmonary toxicity and 1 from hepatic dysfunction).<br><br>Nonfatal hepatic dysfunction was more frequent in the escalated therapy group than in the ablation group (6 patients vs 0 patients, *P*=.001), as were tremor or ataxia (6 patients vs 0 patients, *P*=.01) and drug adverse effects leading to therapy changes (6 patients vs 0 patients, *P*=.01).<br><br>In the escalated therapy group, treatment-related adverse events were more frequent (51 vs 22, *P*=.002) and occurred in more patients (39 vs 20, *P*=.003). There was a higher incidence of SVT at a rate below the detection limit of the ICD at any time during the trial in the escalated therapy group than in the ablation group;	Conclusions:<br>Among patients with ICM who had recurrent VT and an ICD despite first-line AAD therapy, the rate of the composite outcome of death at any time or VT storm or appropriate ICD shock after 30 days was lower than that among patients who received escalated AAD therapy. In addition, treatment-attributed adverse events were more frequent in the escalated therapy group than in the ablation group.<br><br>A significant benefit of catheter ablation with respect to the primary outcome in this trial was observed only among patients in whom the index arrhythmia had occurred despite amiodarone therapy at baseline.<br><br>Limitations:<br>First, it was not powered to assess the effect of the two treatments on mortality. Second, although the

Study name or acronym; Author; Year published; PMID	Aim of study; Endpoints; Study type (RCT, observational — multicenter or single center; case series or other [if RCT include intervention and comparator]); Study size (N)	Patient population with inclusion and exclusion criteria	Results (absolute event rates, *P* values; OR or RR; 95% CI)	Other relevant findings or adverse events	Limitations; Other comments; Conclusions
			the ablation group and in 54 (42.5%) patients in the escalated therapy group (HR 0.77; 95% CI 0.53–1.14; *P*=.19).	there was also a greater total number of episodes of such events (*P*=.02).<br><br>The rate of the primary outcome did not differ significantly between the two groups among the subgroup of patients who were not being treated with amiodarone at baseline (*P*=.64). The rate of the primary outcome was significantly lower in the ablation group than in the escalated therapy group among patients in whom the index arrhythmia occurred despite the receipt of amiodarone (*P*=.001).	practitioners who performed catheter ablation in our trial were experienced in the procedure, it is possible that specialized referral centers for ablation of VT could have achieved better procedural outcomes. Third, most of the deaths were attributed to CHF or noncardiac causes, with few deaths from arrhythmia.

Study name or acronym; Author; Year published; PMID	Aim of study; Endpoints; Study type (RCT, observational — multicenter or single center; case series or other [if RCT include intervention and comparator]); Study size (N)	Patient population with inclusion and exclusion criteria	Results (absolute event rates, *P* values; OR or RR; 95% CI)	Other relevant findings or adverse events	Limitations; Other comments; Conclusions
Kuck et al. Impact of substrate modification by catheter ablation on implantable cardioverter-defibrillator interventions in patients with unstable ventricular arrhythmias and coronary artery disease: results from the multicenter randomized controlled SMS (Substrate Modification Study).<br>• Year published: 2017<br>• PMID: 28292751	Aim: To assess whether prophylactic ablation of arrhythmogenic substrate reduces or prevents the recurrence of VT or VF in such patients.<br><br>Endpoints: The primary study endpoint was the time to first recurrence of VT/VF. Secondary endpoints were appropriate ICD therapies; quality of life according to the Medical Outcome Study Short Form-36 score; number of hospital readmissions because of a cardiac indication; severe clinical events (death, number of syncopes, and number of ES episodes, defined as >3 VT episodes within 24 hours).<br><br>A long-term, prospective, multinational randomized trial;<br>10 centers.	Inclusion criteria: Patients between 18 and 80 years of age with CAD; LVEF ≤40%; and clinically unstable spontaneous VT, or cardiac arrest or syncope with unstable VT inducible at the baseline EPS. Unstable VT was defined as VF or VT with a systolic BP <90 mm Hg.<br><br>Exclusion criteria: LV thrombus, NYHA functional class IV; an acute MI within the preceding 2 months; valvular heart disease or a mechanical heart valve; unstable angina; cardiac surgery within the past 2 months; serum creatinine >220 mEq/L (>2.5 mg/dL); thrombocytopenia or coagulopathy; a contraindication to heparin; pregnancy; or participation in another investigational study.	Acute ablation success was achieved in 45 of the 48 (94%) patients who underwent the procedure. Of the 31 patients with VT inducible at baseline, 28 became noninducible, 2 were still inducible, and it was undefined in 1 patient. In the remaining 17 patients, substrate modification was successfully performed as intended.<br><br>A total of 1449 episodes were detected by the ICDs. Of the 912 documented episodes, 612 (152 [25%] in the ablation group) were classified as spontaneous VT/VF episodes in a total of 51 patients (25 ablation-group patients and 26 ICD-only patients). Kaplan–Meier analysis of time to first verified VT/VF episode in 51 patients showed no significant difference.	A total of 117 patients were enrolled: 60 patients to VT ablation plus ICD implantation (ablation group) and 57 patients to ICD-only therapy.<br><br>Six patients randomized into the ablation group did not undergo ablation and were excluded from further analysis because of missing ICD data crucial for the main analysis: 5 of them did not receive an ICD; 1 had an ICD implanted, but important implantation data were missing, and the patient was not followed up at all.<br><br>A total of 111 patients remained for modified intention-to-treat analysis: 54 in the ablation group and 57 in the ICD-only group.<br><br>Of the 54 modified intention-to-treat patients randomized to catheter ablation, 48 actually underwent the procedure. The reasons for not	Conclusions:<br>SMS is the third randomized trial after SMASH-VT9 and VTACH10 to investigate the impact of prophylactic catheter ablation followed by ICD implantation on VT recurrences in patients with sustained VAs. However, it is the first of these 3 trials that did not show a benefit of prophylactic catheter ablation with respect to the primary endpoint of time to recurrence of any VT. Furthermore, none of the secondary endpoints showed significant differences between groups when comparing time to first event. However, catheter ablation did achieve a >50% reduction in the total number of ICD interventions during the duration of follow-up.<br><br>Limitations:<br>Differences in patient and treatment center selection and in ablation strategies might have underestimated in SMS the potential benefit

Study name or acronym; Author; Year published; PMID	Aim of study; Endpoints; Study type (RCT, observational — multicenter or single center; case series or other [if RCT include intervention and comparator]); Study size (N)	Patient population with inclusion and exclusion criteria	Results (absolute event rates, *P* values; OR or RR; 95% CI)	Other relevant findings or adverse events	Limitations; Other comments; Conclusions
	N=111: 54 (ablation group) and 57 (ICD-only group).			performing ablation in 6 patients were the inability to locate a VT substrate (n=3); no vascular access (n=1); pericardial tamponade (n=1); and hemodynamic instability during the procedure (n=1).	of VT ablation with respect to time to first VT/VF event. However, despite all limitations, the benefit of prophylactic ablation could be demonstrated, with a >50% reduction in the number of almost all types of arrhythmia episodes, when the times to multiple VT/VF events were analyzed.

Study name or acronym; Author; Year published; PMID	Aim of study; Endpoints; Study type (RCT, observational — multicenter or single center; case series or other [if RCT include intervention and comparator]); Study size (N)	Patient population with inclusion and exclusion criteria	Results (absolute event rates, *P* values; OR or RR; 95% CI)	Other relevant findings or adverse events	Limitations; Other comments; Conclusions
Makimoto et al. Clinical impact of mapping strategies for treatment of ventricular tachycardias in patients with structural heart disease.<br>• Year published: 2015<br>• PMID: 25639823	Aim: To compare the efficacy between substrate-based and activation/entrainment-based ablation.<br><br>Endpoints: VT recurrences, ESs, HF, and cardiac deaths.<br><br>A single-center observational study.<br><br>N=85; the primary strategy was activation/entrainment mapping (Group-AE, N=35); otherwise, substrate-based strategy was adopted (Group-S, N=50) because of noninducibility of VT or hemodynamic instability.	Eighty-five consecutive patients (62 male, 53 ± 16 years) with SHD who underwent catheter ablation targeting VT between 1999 and 2009 were included in this study. There was at least one episode of monomorphic SVT.<br>The underlying SHDs were ARVC in 34 patients, ICM in 16, DCM in 14, HCM in 1, DHCM in 2, cardiac sarcoidosis in 11, CHD (ToF) in 6, and mitral valve insufficiency in 1.<br>An ICD was implanted in 43 patients.<br>Before the index ablation, 30 patients experienced a VT storm, which was defined as 3 or more sustained episodes of VT over 24 hours, including events treated by antitachycardia pacing or shocks; the number of documented VT episodes was 1.4 ± 0.7.	There were no significant differences between the 2 strategies in the incidence of VT recurrences, ESs, HF, cardiac deaths, or the number of combined AADs at the time of the VT recurrence.	The LVEF was 51.7% ± 16.4%.<br><br>The endpoint of the procedure differed between the 2 groups. However, there was no significant difference in the recurrence rate between them regardless of the procedure results during 5 years of long-term follow-up. This suggested that the endpoint definitions were reasonable.	Conclusion:<br>The substrate-based strategy adopted as an alternative option when the activation/entrainment-based strategy was unable to be performed resulted in a comparable occurrence rate of VT, hospitalization because of heart failure, and cardiac death during 5 years of follow-up. If a VT is inducible, the ultimate goal of the procedure should be the extinction of the inducible VT regardless of the mapping strategy.<br><br>Limitations:<br>First, the study population was small, and it was a single center experience. Second, they did not perform epicardial mapping or ablation. Third, they included various SHDs in this study. Fourth, they divided the patients into two groups based on the mapping strategy for the clinical VTs. Finally, this was not a randomized study.

Study name or acronym; Author; Year published; PMID	Aim of study; Endpoints; Study type (RCT, observational — multicenter or single center; case series or other [if RCT include intervention and comparator]); Study size (N)	Patient population with inclusion and exclusion criteria	Results (absolute event rates, *P* values; OR or RR; 95% CI)	Other relevant findings or adverse events	Limitations; Other comments; Conclusions
Briceño et al. Long-term outcomes of different ablation strategies for ventricular tachycardia in patients with structural heart disease: systematic review and meta-analysis.<br>• Year published: 2018<br>• PMID: 28575378	Aim: To compare the long-term outcomes of standard ablation of stable VT vs substrate modification, and of complete vs incomplete substrate modification in patients with SHD presenting with VT.<br><br>Primary endpoints: (1) A composite of long-term VA recurrence and all-cause mortality of standard ablation of stable VT vs substrate modification; (2) long-term VA recurrence of standard ablation of stable VT vs substrate modification; (3) long-term all-cause mortality of standard ablation of stable VT vs substrate modification; and (4) long-term VA recurrence in complete vs incomplete substrate modification.<br><br>Meta-analysis	Using PubMed, Embase, and Cochrane Central Register of Clinical Trials (Cochrane Library, Issue 09, 2016).<br><br>Inclusion criteria:<br>Human studies in peer-reviewed journals up to September 2016. No language restriction was applied. The reference lists of identified articles were also reviewed. This search was conducted using the terms (VT OR ventricular tachyarrhythmia OR VT ablation) AND (substrate ablation OR standard ablation OR activation mapping OR entrainment mapping OR activation and entrainment mapping OR VT recurrence OR mortality OR SHD OR late potentials OR scar dechanneling OR local abnormal ventricular activities OR core isolation OR homogenization of the scar).	A total of 11,280 articles were identified, 10,756 of which did not meet inclusion criteria. After evaluation of the 524 remaining abstracts, 13 studies fulfilled the inclusion criteria.<br><br>ICM (>70%).<br><br>Substrate modification was associated with decreased composite VA recurrence and all-cause mortality (RR 0.57; 95% CI 0.40–0.81) compared with standard ablation of stable VTs.<br><br>Complete substrate modification was associated with decreased VA recurrence (RR 0.39; 95% CI 0.27–0.58) compared with incomplete substrate modification.		Conclusions:<br>In patients with SHD who had VT related mainly to ischemic substrates, there was a significantly lower risk of the composite primary outcome of long-term VA recurrence and all-cause mortality among those undergoing substrate modification compared with standard ablation. Long-term success is improved when performing complete substrate modification.<br><br>Limitations:<br>First, there is only one randomized study in our analysis comparing both ablation strategies, which likely provides accurate assessment and comparison of both ablation approaches, in contrast to the other studies included. Second, the number of patients included in each of the studies is small in view of the specific population studied, hence the need to do a composite outcome. However, the use

Study name or acronym; Author; Year published; PMID	Aim of study; Endpoints; Study type (RCT, observational — multicenter or single center; case series or other [if RCT include intervention and comparator]); Study size (N)	Patient population with inclusion and exclusion criteria	Results (absolute event rates, *P* values; OR or RR; 95% CI)	Other relevant findings or adverse events	Limitations; Other comments; Conclusions
	Six studies for the comparison of standard ablation of stable VT vs substrate modification, with a total of 396 patients, and 7 studies to assess the impact of extensive substrate modification, with a total of 391 patients.	Selection criteria: The PRISMA statement for reporting systematic reviews and meta-analyses was applied to the methods for this study. The studies had to fulfill the following criteria to be included in the analysis of standard ablation of stable VT vs substrate modification: patients with SHD presenting for VT ablation; studies that strictly compared standard ablation of stable VT with substrate modification without the use of hybrid strategies (ie, combining substrate with standard ablation); studies including VT or VA recurrence and/or total mortality as their endpoints; and studies that were not designed to use hemodynamic support for standard VT ablation as a primary strategy. The studies had to fulfil the following			of the composite endpoint improves the resolving ability of the meta-analysis, strengthening its capacity to pick out weaker signals of effect from the background noise of sampling error. Third, the substrate modification strategies are all different between studies; hence, it is difficult to generalize that all these are better than standard-ablation. Fourth, our results apply mostly to ICM and should not be generalized to NICM substrates. Fifth, as any meta-analysis including interventions, there is a potential for significant bias related to the operators' experience from each study center.

Study name or acronym; Author; Year published; PMID	Aim of study; Endpoints; Study type (RCT, observational — multicenter or single center; case series or other [if RCT include intervention and comparator]); Study size (N)	Patient population with inclusion and exclusion criteria	Results (absolute event rates, *P* values; OR or RR; 95% CI)	Other relevant findings or adverse events	Limitations; Other comments; Conclusions
		criteria to be included in the analysis of complete vs incomplete substrate modification: patients with SHD presenting for VT ablation, studies that reported complete and incomplete substrate modification, and studies including VT or VA recurrence and/or total mortality as their endpoints.			

Study name or acronym; Author; Year published; PMID	Aim of study; Endpoints; Study type (RCT, observational — multicenter or single center; case series or other [if RCT include intervention and comparator]); Study size (N)	Patient population with inclusion and exclusion criteria	Results (absolute event rates, *P* values; OR or RR; 95% CI)	Other relevant findings or adverse events	Limitations; Other comments; Conclusions
Kumar et al. Substrate-based ablation versus ablation guided by activation and entrainment mapping for ventricular tachycardia: a systematic review and meta-analysis.<br>• Year published: 2016<br>• PMID: 27574120	Aim: To compare the acute procedural efficacy and outcomes of predominantly substrate-based ablation versus ablation guided predominantly by activation and entrainment mapping.<br><br>Endpoints: The primary endpoint was the relative risk of VT recurrence at follow-up; secondary endpoints were acute success based on noninducibility of VT; procedural complications; and mortality.<br><br>A systematic review and meta-analysis. Database searches through April 2016 identified 6 eligible studies (enrolling 403 patients, with 1 randomized study) comparing the 2 strategies.	Used PubMed, Embase, Web of Science, Scopus, and Cochrane Database for published studies describing the outcomes of VT ablation, using the broad search term of "ventricular" and "ablation" in either the title or the abstract. No limits of start date were applied. The search was conducted up through 24 April 2016. Reference lists from review articles were also used.<br>Guidelines and meta-analyses were manually searched for references otherwise not listed in the initial search. Abstracts from conference meetings were not included in the search strategy. All randomized controlled trials, case-control studies, and cohort studies were reviewed.<br><br>Studies were included if they enrolled patients with SHD and VT referred	The initial search yielded 9551 references. After exclusions, the full text of 290 studies reporting outcomes of catheter ablation of VT were reviewed. Six studies directly compared a predominantly substrate-based ablation with a predominantly activation/entrainment-guided ablation strategy. The majority of the studies were excluded because they used a combination of activation/entrainment and substrate mapping and lacked a direct comparison between the 2 strategies.<br><br>There was no significant publication bias for the primary outcome as assessed by Funnel plots.<br><br>At a median follow-up of 18 months, substrate-based vs activation/entrainment	A number of studies described different substrate-based ablation strategies in the absence of a control group undergoing ablation predominantly guided by activation/entrainment mapping.<br><br>A number of studies reported the use of hemodynamic support devices to facilitate detailed activation/entrainment but lacked a control group of patients undergoing ablation using a predominantly substrate-based ablation.	Conclusions: This meta-analysis demonstrates similar acute procedural efficacy, complications, VT recurrence, and mortality rates when comparing a predominantly substrate-based ablation strategy to a strategy guided predominantly by activation and entrainment mapping of inducible and hemodynamically tolerated VTs.<br><br>Limitation:<br>The current meta-analysis comprises only 6 studies. These 6 studies comprise a relatively small cohort with heterogeneity in both clinical phenotype (ie, ICM vs NICM) and procedural methodology. The majority of the studies included in this meta-analysis were retrospective studies. Furthermore, it is important to note that the experience, skill, and commitment to a comprehensive approach to either technique also might influence outcomes.

Study name or acronym; Author; Year published; PMID	Aim of study; Endpoints; Study type (RCT, observational — multicenter or single center; case series or other [if RCT include intervention and comparator]); Study size (N)	Patient population with inclusion and exclusion criteria	Results (absolute event rates, *P* values; OR or RR; 95% CI)	Other relevant findings or adverse events	Limitations; Other comments; Conclusions
		for catheter ablation; had reported data on VT recurrence; compared the predominantly substrate-based ablation strategy in one arm with an ablation strategy predominantly guided by activation/entrainment mapping in the other study arm.<br><br>Case reports, reviews, meta-analyses, and studies published in languages other than English were excluded.	guided VT ablation showed no significant difference in VT recurrence (RR 0.72; 95% CI 0.44–1.18; *P*=.2); acute success (RR 1.02; 95% CI 0.95–1.1; *P*=.6); procedural complications (RR 0.8; 95% CI 0.35–1.82; *P*=.5); cardiovascular mortality (RR 0.83; 95% CI 0.38–1.79; *P*=.6); and total mortality (RR 0.76; 95% CI 0.36–1.59; *P*=.5).		

Study name or acronym; Author; Year published; PMID	Aim of study; Endpoints; Study type (RCT, observational — multicenter or single center; case series or other [if RCT include intervention and comparator]); Study size (N)	Patient population with inclusion and exclusion criteria	Results (absolute event rates, *P* values; OR or RR; 95% CI)	Other relevant findings or adverse events	Limitations; Other comments; Conclusions
Berte et al. Impact of electrode type on mapping of scar-related VT.<br>• Year published: 2015<br>• PMID: 26198475	Aim: To define the efficacy of multipolar mapping catheters by comparing maps obtained with quadripolar ablation catheters to those obtained in the same patients with multipolar small electrode mapping catheters.<br><br>A single-center prospective study.<br><br>N=13; 4 sheep (infarction model) and 9 patients.	Consecutive patients referred for VT ablation were included. Endocardial and/or epicardial substrate maps were made in random order using a standard bipolar 3.5 mm catheter (NAV) and a multipolar catheter (PR).	Using the multipolar catheter, 8702 (1088 ± 839) endocardial and 11,282 (1612 ± 789) epicardial mapping points were recorded. With the NAV catheter, 1578 (197 ± 86) endocardial and 1699 (243 ± 48) epicardial mapping points were recorded.<br><br>The density inside the scar was significantly higher using PR (PR vs NAV, 3.16 vs 0.70 points/cm^2; *P*=.001). The overall low-voltage area was significantly larger using PR (PR vs NAV, 68 ± 55 vs 58 ± 48 cm^2; *P*=.001). The epicardial low-voltage scar area in humans was larger using PR (PR vs NAV, 95 ± 72 vs 78 ± 66 cm^2; *P*=.018). There was a trend toward a larger endocardial low-voltage area in both animals and patients (PR vs NAV, animals: 37 ± 3.8 vs 33 ± 7.5 cm^2, *P*=.144; patients:	In total, there were 818 endocardial and epicardial point pairs: 285 point pairs in 4 animals and 533 point pairs in 9 humans were found with a distance 3 mm. Overall, automatic far-field voltage measurements were significantly lower using PR in both bipolar and unipolar mode.<br>When all 818 point pairs were analyzed separately, there was no bipolar voltage difference (PR vs NAV bipolar, 3.29 ± 2.07 vs 3.11 ± 2.87 mV, respectively, *P*=.294); unipolar voltage was lower with PR versus NAV mapping in normal tissue (PR vs NAV, 6.49 ± 3.51 vs 7.25 ± 4.84 mV, respectively; *P*=.003; n=241). In the border zone of the low-voltage area, a significantly lower bipolar and a trend toward lower unipolar voltage was observed with PR (PR vs NAV, bipolar: 0.92 ± 0.29 vs 1.46 ± 1.33 mV, respectively, *P*< 0.001; unipolar: 4.25 ±	Conclusion:<br>PentaRay mapping increases map accuracy for EGM characteristics, map density, channel detection, and LAVA detection. Based on these findings, multipolar mapping would be preferable for accurate substrate assessment. The impact on clinical outcome should be investigated in future studies.<br><br>Limitations:<br>First, this was a single-center study. Second, there is currently no gold standard to detect LAVA activity. Third, LAVA analysis was performed manually. In addition, CF is known to impact the recorded signals, but no multipolar mapping catheters are capable of CF measurement. Fourth, a HANA signal was considered as a far-field signal to facilitate analysis. Finally, follow-up is relatively short.

Study name or acronym; Author; Year published; PMID	Aim of study; Endpoints; Study type (RCT, observational — multicenter or single center; case series or other [if RCT include intervention and comparator]); Study size (N)	Patient population with inclusion and exclusion criteria	Results (absolute event rates, *P* values; OR or RR; 95% CI)	Other relevant findings or adverse events	Limitations; Other comments; Conclusions
			50 ± 16 vs 47 ± 16 cm^2, *P*=.068). More voltage-based channels were detected using PR (PR vs NAV, 2.0 ± 1.8 vs 0.5 ± 0.8; *P*=.017). In the animals, 8 channels were found in 2 animals (50% of animals) using PR; all were missed by NAV mapping. In the humans, 18 channels were identified using PR in 7 patients (78%); 12 of the 18 (67%) were missed by NAV. NAV detected 6 channels in 4 patients, also detected by PR.	2.46 vs 4.42 ± 2.54 mV, respectively, *P*=.156; n=263). Inside dense scar a significantly lower bipolar and a trend toward lower unipolar voltage was observed (PR vs NAV, bipolar: 0.25 ± 0.13 vs 0.76 ± 0.86 mV, respectively, *P*< 0.001 and unipolar: 2.05 ± 0.89 vs 2.49 ± 0.99 mV, respectively, *P*=.630; n=298). In 54 point pairs (<3 mm), agreement on LAVA was analyzed between PR and NAV mapping. Manual bipolar LAVA voltage measurement was significantly higher for PR versus NAV mapping (0.48 ± 0.33 vs 0.31 ± 0.21 mV, respectively; *P*=.0001). There was an overall agreement of 72% between PR and NAV in terms of EGM analysis. The agreement on LAVA was only 40%, and on normal EGM was 82%. When using PR as the comparator, NAV had a sensitivity of 49%, specificity of 77%, PPV of 33%, and NPV of 87%.	

Study name or acronym; Author; Year published; PMID	Aim of study; Endpoints; Study type (RCT, observational — multicenter or single center; case series or other [if RCT include intervention and comparator]); Study size (N)	Patient population with inclusion and exclusion criteria	Results (absolute event rates, *P* values; OR or RR; 95% CI)	Other relevant findings or adverse events	Limitations; Other comments; Conclusions
Yamashita et al. Impact of new technologies and approaches for post-myocardial infarction ventricular tachycardia ablation during long-term follow-up.<br>• Year published: 2016<br>• PMID: 27406604	Aim: To identify the impact of some innovations on ablation outcome for post-MI VT.<br><br>Endpoints: Complete LAVA elimination during sinus rhythm and noninducibility of any VT<br><br>A single-center observational study.<br><br>N=125	From January 2008 to November 2013, consecutive patients undergoing catheter ablation for post-MI VT were enrolled.<br><br>Inclusion criteria: History of MI, drug-resistant sustained VT.<br><br>Exclusion criteria: Presence of intracardiac thrombus, NYHA class IV HF, cardiac surgery within the past 2 months (unless VT was incessant).<br><br>VT storm was defined as ≥3 VT episodes in 24 hours.<br><br>A total of 140 patients met the inclusion criteria; 15 patients were excluded: 8 for intracardiac thrombus, 2 for insufficient delay post-MI (VT spontaneously resolved in both), and 5 declined consent.	VT was inducible at baseline in 106 cases (75%). A total of 216 VTs were induced, 55 (25%) of which were mapped and terminated by RF application, 161 (75%) of which were unmappable.<br><br>After a total RF time of 32 minutes (IQR 20–50), complete LAVA elimination was achieved in 79 cases (60% of patients with LAVA).<br>At the end of the procedure, VT inducibility was not tested in 30 of 142 patients (21%). Of the remaining 112 patients who underwent programmed stimulation, there was an endpoint of VT noninducibility in 93 of 112 patients (83%). After a median follow-up of 850 days, VT recurred in 53 of 146 patients (36%).<br><br>The ability to achieve complete LAVA elimination or VT	Transseptal, retrograde aortic, and epicardial approaches were performed in 124 (87%), 48 (34%), and 52 cases (37%), respectively. Criteria for epicardial access was met in 64 cases (45%), in whom pericardial access was not possible in 12 cases (3 cases because of pericardial adhesion; 7 cases because of previous cardiac surgery; 1 case because of a risk of bleeding by dual-antiplatelet therapy; and 1 case because of pericardial bleeding). A multipolar mapping catheter was used in 73 cases (51%).<br><br>Eight patients had pericardial bleeding (5 related to epicardial approach). One patient required surgery, whereas the others resolved spontaneously.<br><br>Permanent AV block occurred in 1 patient.<br><br>There were no strokes,	Conclusion:<br>This study demonstrated that the ability to achieve complete LAVA elimination, the integration of scar data from preprocedural imaging, and the use of multipolar catheters to perform high-density mapping enhances VT-free survival after post-MI VT ablation. Our results further confirm that complete LAVA elimination is a procedural endpoint of high predictive value.<br><br>Limitations:<br>The impact of image integration and multipolar catheters on patient outcome after post-MI VT ablation should be confirmed in a randomized-controlled fashion.<br><br>The small sample size, which particularly prevented us from analyzing predictors of mortality.<br><br>A potential confounding effect of accumulating

Study name or acronym; Author; Year published; PMID	Aim of study; Endpoints; Study type (RCT, observational — multicenter or single center; case series or other [if RCT include intervention and comparator]); Study size (N)	Patient population with inclusion and exclusion criteria	Results (absolute event rates, *P* values; OR or RR; 95% CI)	Other relevant findings or adverse events	Limitations; Other comments; Conclusions
		The study population consisted of 125 patients (age, 64 ± 11 years; 7 women; 100 [80%] ICD) who underwent 142 VT ablation procedures.	noninducibility was not related to any baseline characteristics. Among procedural characteristics, low-voltage zone and dense scar zone tended to be associated with failure to achieve LAVA elimination. Baseline inducibility was associated with a lower rate of noninducibility at the end of the procedure (*P*=.002). Total RF and procedure time were also associated with lower rates of noninducibility at the end of the procedure (*P*=.0003 and *P*=.002, respectively).<br><br>On multivariable analysis, 3 characteristics were associated with the outcome: (1) the ability to achieve complete LAVA elimination (R2=.29; *P*<.0001; RR 0.52, 0.38–0.70), (2) the use of real-time image integration (R2=.21; *P*=.0006; RR 0.49, 0.33–0.74), (3) the use of multipolar catheters	phrenic palsies, coronary injuries, or procedure-related deaths.<br><br>One hundred of 125 (80%) patients had an ICD implanted before VT ablation. A further 3 patients had an ICD implanted after VT ablation.	experience for one technique influencing outcome of subsequently introduced techniques.<br><br>Another potential bias would be an evolution in the indication to perform VT ablation during the course of the study.

Study name or acronym; Author; Year published; PMID	Aim of study; Endpoints; Study type (RCT, observational — multicenter or single center; case series or other [if RCT include intervention and comparator]); Study size (N)	Patient population with inclusion and exclusion criteria	Results (absolute event rates, *P* values; OR or RR; 95% CI)	Other relevant findings or adverse events	Limitations; Other comments; Conclusions
			(R^2=.08; *P*=.05; RR 0.75, 0.56–1.00).<br><br>Follow-up at 1 year was available for 23 patients in whom both multielectrode mapping and image integration had been used. LAVA elimination had been achieved in 18 of 23 patients (78%); 20 of 23 patients (87%) were free from recurrence.		

Study name or acronym; Author; Year published; PMID	Aim of study; Endpoints; Study type (RCT, observational — multicenter or single center; case series or other [if RCT include intervention and comparator]); Study size (N)	Patient population with inclusion and exclusion criteria	Results (absolute event rates, *P* values; OR or RR; 95% CI)	Other relevant findings or adverse events	Limitations; Other comments; Conclusions
Hutchinson et al. Endocardial unipolar voltage mapping to detect epicardial ventricular tachycardia substrate in patients with nonischemic left ventricular cardiomyopathy.<br>• Year published: 2011<br>• PMID: 21131557	Aim: To determine whether ENDO unipolar mapping with a larger electric field of view could identify EPI low bipolar voltage regions in patients with LVCM undergoing VT ablation.<br><br>Endpoints: ENDO UNI voltage and area of low voltage; EPI bipolar voltage and area of low voltage.<br><br>A single-center observational study.<br><br>N=16	They examined consecutive patients undergoing VT ablation from June 2002 to June 2010.<br><br>Inclusion Criteria: Patients undergoing detailed(>100 points) ENDO and EPI EAM with complete sampling of all LV segments.<br><br>The decision for an epicardial approach was made based on either (1) the characteristics of the VT on the surface 12-lead ECG; (2) the presence of epicardial substrate on imaging studies (CT, MRI, ICE); and/or (3) the failure of prior endocardial ablation procedure.<br><br>From the total cohort, they examined patients with normal LV ENDO bipolar voltage from the initial cohort: (1) patients with structurally normal hearts and normal EPI	Of the 1517 patients undergoing ablation for VT between June 2002 and June 2010, 168 had a combined ENDO-EPI procedure; 16 had detailed mapping of the ENDO and EPI with normal ENDO bipolar voltage. From this group of 16 patients, 5 had structurally normal hearts and normal bipolar EPI voltage (EPI-) and underwent mapping and ablation of idiopathic VT/ventricular premature depolarization. Eleven additional patients had LVCM and VT with confirmed epicardial origin with a confluent region (>2 cm^2) of EPI bipolar low voltage present (EPI+).<br><br>In the EPI+ (versus EPI- groups), the mean ENDO unipolar voltage was significantly lower (10.5 ± 3.2 vs 14.7 ± 2.6 mV, *P*=.04). There was no	The reference population consisted of 6 patients (5 males, 1 female), with a mean age of 36 ± 18 years. A total of 683 LV electrograms were analyzed (range, 100–168 points per patient). Ninety-five percent of LV ENDO unipolar signals had an amplitude >8.27 mV (mean, 19.6 ± 6.9 mV), defined as the value of normal LV ENDO unipolar signal amplitude.<br><br>The mean number of electroanatomic points on the ENDO and EPI maps was similar between EPI+ and EPI- patients (ENDO maps: 188 ± 57 vs 166 ± 67, *P*=.3; EPI maps: 491 ± 178 vs 361 ± 206, *P*=.1).<br><br>There was no difference in the ENDO-EPI distance in the EPI+ versus EPI- patients (16.0 ± 3.0 vs 15.0 ± 5.4 mm, *P*=.5).	Conclusions:<br>These results suggest that unipolar ENDO voltage can provide an indication of epicardial VT substrate in patients with LVCM with normal bipolar endocardial voltage.<br><br>Limitations:<br>This study includes a limited number of patients.

Study name or acronym; Author; Year published; PMID	Aim of study; Endpoints; Study type (RCT, observational — multicenter or single center; case series or other [if RCT include intervention and comparator]); Study size (N)	Patient population with inclusion and exclusion criteria	Results (absolute event rates, *P* values; OR or RR; 95% CI)	Other relevant findings or adverse events	Limitations; Other comments; Conclusions
		bipolar voltage (EPI-, group 1); (2) patients with LVCM and LV EPI bipolar low-voltage regions present (EPI+, group 2).<br><br>SHD was excluded in the EPI- patients with transthoracic echocardiography and stress testing (if >30 years old).<br><br>Exclusion criteria: Arrhythmogenic RV dysplasia or cardiomyopathy, cardiac sarcoidosis, and alcoholic cardiomyopathy.	difference in ENDO bipolar voltage.<br>For the confluent ENDO unipolar low-voltage regions (>2 cm^2), in 9 of 11 (82%) of the EPI+, and in none of 5 of the EPI-, the mean EPI bipolar low-voltage area was significantly larger than the corresponding ENDO unipolar area (49.1 ± 38.4 vs 19.2 ± 28.2 cm^2, *P*=.02). Of the total ENDO unipolar low-voltage area, 61% directly overlapped EPI bipolar low-voltage regions. The mean LV ENDO unipolar voltage within the low-voltage areas in the EPI+ patient cohort was 5.5 ± 1.7 mV.		

Study name or acronym; Author; Year published; PMID	Aim of study; Endpoints; Study type (RCT, observational — multicenter or single center; case series or other [if RCT include intervention and comparator]); Study size (N)	Patient population with inclusion and exclusion criteria	Results (absolute event rates, *P* values; OR or RR; 95% CI)	Other relevant findings or adverse events	Limitations; Other comments; Conclusions
Polin et al. Endocardial unipolar voltage mapping to identify epicardial substrate in arrhythmogenic right ventricular cardiomyopathy/ dysplasia.<br>• Year published: 2011<br>• PMID: 20933099	Aim: To test the hypothesis that endocardial unipolar voltage mapping in patients with RV VT and preserved endocardial bipolar voltage abnormalities might identify the extent of epicardial bipolar voltage abnormality.<br><br>Endpoints: Predictability of endocardial unipolar mapping for epicardial bipolar voltage abnormalities.<br><br>A single-center observational study.<br><br>Group 1 patients: Retrospective evaluation N=10<br><br>Group 2 patients: Prospective evaluation N=13	Group 1 patients: Retrospective evaluation. Thirteen consecutive patients presenting with ARVC/D and documented VT who underwent simultaneous endocardial and epicardial catheter mapping of VT between June 2005 and December 2008 were included in the initial phase of the analysis. All patients fulfilled task force criteria for ARVC/D with evidence of RV dilation, segmental wall motion abnormalities, ECG abnormalities, and multiple LBBB VT morphologies. Ultimately, 13 patients were excluded from the analysis if the RV endocardial bipolar free-wall scar burden was >50% of the total RV free wall surface area. This was done to optimize the chance that we could demonstrate a more extensive area of epicardial voltage	Some 10 patients had endocardial bipolar voltage abnormalities that incorporated 0%–42% of the total RV endocardial free-wall surface area. The area of endocardial and epicardial bipolar low voltage generally involved the perivalvular region of the tricuspid valve and/or pulmonary valve and extended for a variable distance across the RV free wall.<br><br>The area of low-voltage bipolar electrogram abnormality was always greater on the epicardial voltage map than on the endocardial bipolar voltage map (82 ± 22 cm^2, range 43–127 vs 22 ± 17 cm^2, range 0–52, *P*<.0001); the area of electrogram abnormalities on endocardial bipolar voltage maps did not correlate significantly with the	To establish a reference value for normal unipolar electrograms, detailed RV endocardial sinus rhythm mapping was performed in 8 patients (6 men and 2 women, mean age 36 ± 18 years) without SHD undergoing electrophysiologic evaluation. From 105 to 164 endocardial RV electrograms were recorded per patient in the 8 reference patients; 95% of unipolar signals had an amplitude >5.5 mV and defined a normal unipolar electrogram amplitude. Dense scar for display purposes was arbitrarily defined as having a unipolar signal amplitude >3.5 mV.	Conclusion:<br>This study describes a new technique for identifying the presence and anatomic extent of anticipated epicardial bipolar voltage abnormalities consistent with "scar" in patients with ARVC/D. The study defined a normal unipolar RV endocardial signal as having an amplitude >5.5 mV and validated the use of endocardial unipolar voltage mapping to identify confluent areas of signals with an amplitude <5.5 mV as a strategy for approximating the degree and location of epicardial bipolar voltage abnormality in patients with arrhythmogenic RV cardiomyopathy/dysplasia with only limited endocardial bipolar voltage changes. The value of endocardial unipolar voltage mapping was demonstrated convincingly in a retrospective analysis and an online prospective cohort. Importantly, using the 5.5-mV cutoff, no false-positive recordings in the 7 patients

Study name or acronym; Author; Year published; PMID	Aim of study; Endpoints; Study type (RCT, observational — multicenter or single center; case series or other [if RCT include intervention and comparator]); Study size (N)	Patient population with inclusion and exclusion criteria	Results (absolute event rates, *P* values; OR or RR; 95% CI)	Other relevant findings or adverse events	Limitations; Other comments; Conclusions
		abnormality that would be identified with the endocardial unipolar signal analysis and not have the unipolar changes just reflect the extensive endocardial substrate. Three patients were excluded on this basis.<br><br>Three other patients with structurally normal hearts who underwent both endocardial and epicardial catheter mapping of idiopathic VT demonstrated to ultimately originate from the LVOT served as additional controls to validate accuracy of unipolar reference values.<br><br>Group 2 patients: Prospective evaluation in 13 additional patients with RV VT (Group 2). Eight of the 13 patients had task force criteria consistent with ARVC; 1 had biventricular dilated cardiomyopathy; the	epicardial bipolar voltage maps (r =0.49, *P*=.15).<br><br>The percentage of electrogram abnormalities on the RV free-wall surface area was not significantly different between endocardial unipolar voltage mapping (61% ± 19%, range 33%–96%) and epicardial bipolar voltage mapping (*P*=.23).<br><br>There was also a significant correlation between the percent abnormal voltage areas (r=0.63, *P*=.05) for endocardial unipolar and epicardial bipolar maps in terms of both overall size and matching anatomic location.<br><br>An online assessment was performed for 13 consecutive patients who presented with suspected RV epicardial VT who underwent detailed		with idiopathic RV or LV VT were identified. These unipolar voltage recording techniques appear to accurately predict the location and extent of epicardial involvement and might help in decision making related to proceeding to epicardial substrate mapping and ablation.<br>Study limitations:<br>The areas of electrogram abnormalities from both the endocardium and epicardium were not corroborated by histopathologic analysis. Furthermore, because the majority of patients in this cohort had an ICD, no attempt was made to correlate unipolar and bipolar voltage map findings with cardiac DE-MRI findings. In addition, this study did not analyze areas of the RV near the septum to avoid an LV-related contamination of the unipolar RV signal amplitude. Given that unipolar signal amplitude is generated by a tissue volume surrounding the tip

Study name or acronym; Author; Year published; PMID	Aim of study; Endpoints; Study type (RCT, observational — multicenter or single center; case series or other [if RCT include intervention and comparator]); Study size (N)	Patient population with inclusion and exclusion criteria	Results (absolute event rates, *P* values; OR or RR; 95% CI)	Other relevant findings or adverse events	Limitations; Other comments; Conclusions
		remaining 4 patients had RV VT without diagnostic criteria for ARVC/D or DCM. These 4 patients were included to help define the specificity of the unipolar voltage findings.<br><br>AADs were discontinued 5 half-lives before all mapping procedures.	endocardial and epicardial mapping.<br><br>The area of endocardial and epicardial bipolar low voltage generally involved the perivalvular region of the tricuspid valve and extended for a variable distance across the RV free wall when present. The epicardial abnormality typically was greater than the endocardial abnormality.<br><br>Notably, the endocardial unipolar low-voltage area correlated closely with the epicardial bipolar area with respect to both size (68 ± 41 cm^2, 56% ± 28% total area, r =0.81, *P*=.008) and location. In contrast, no significant correlation was found between endocardial bipolar and epicardial bipolar electrogram abnormalities.		electrode, it is possible that unipolar voltage might be reduced due to adjacent pathology rather than epicardial fibrosis specifically. The patient population was limited to ARVC/D patients with only modest endocardial bipolar voltage abnormalities because no reports in the literature have described ARVC/D pathologic specimens in which fibrofatty replacement is limited to the endocardial layer with sparing of the midmural and/or epicardial layers. On the other hand, limited endocardial bipolar voltage changes in the presence of more impressive unipolar changes might be a helpful gauge to support a combined endocardial/epicardial ablation approach. Most of the patients in this study had undergone prior ablation attempts and had extensive epicardial substrate. It is possible that our findings would not apply to patients with milder disease.

Study name or acronym; Author; Year published; PMID	Aim of study; Endpoints; Study type (RCT, observational — multicenter or single center; case series or other [if RCT include intervention and comparator]); Study size (N)	Patient population with inclusion and exclusion criteria	Results (absolute event rates, *P* values; OR or RR; 95% CI)	Other relevant findings or adverse events	Limitations; Other comments; Conclusions
Chopra et al. Relation of the unipolar low-voltage penumbra surrounding the endocardial low-voltage scar to ventricular tachycardia circuit sites and ablation outcomes in ischemic cardiomyopathy.<br>• Year published: 2014<br>• PMID: 24576211	Aim: To investigate the relationship of combined endocardial unipolar and bipolar voltage maps and location of presumptive VT isthmus sites to VT ablation outcomes in ischemic cardiomyopathy.<br><br>Endpoints: Area and size of endocardial unipolar low voltage,<br>VT isthmus or exits localization.<br><br>A retrospective pilot study.<br><br>N=20 (8 with recurrent, 12 without)	Twenty consecutive patients with ICM who underwent endocardial catheter mapping and ablation for SMVT, and who met the criteria below were retrospectively reviewed: (1) SMVT was inducible; (2) endocardial electroanatomic voltage maps were available for analysis of bipolar and unipolar voltages.<br><br>ICM was defined if the patient had any 2 or more features from the following: history of prior myocardial infarction; epicardial coronary artery stenosis of >70%; prior coronary artery revascularization; evidence of infarction on the ECG; evidence of regional wall motion abnormality or scar, and depressed LVEF on echocardiography, MRI, or nuclear imaging.	The total mean LV endocardial area mapped per patient was 230 ± 66 cm^2. The mean area of LVA (<1.5 mV) was 70 ± 20 cm^2 (30% of the mapped LVA).<br><br>In all patients, the endocardial unipolar LVA incorporated the endocardial bipolar LVA and extended beyond it creating a unipolar LVA “penumbra” on the EAM. The mean size of the total endocardial unipolar LVA per patient was 147 ± 47 cm^2. The unipolar LVA penumbra around the bipolar LVA had an area of 77 ± 40 cm^2 , not different between the anterior and inferior/posterior scars (66.6 ± 37 vs 87 ± 42; *P*=.2, t-test).<br><br>The size of the unipolar LVA penumbra did not differ between the groups who did (88 ± 47 cm^2) and did not (69 ± 35 cm^2) have	There was no significant difference between the 2 groups in terms of age; LVEF; AAD usage; prior ablations; total LV area; infarct LVA location; size of bipolar (<1.5 mV) and unipolar (<8.3 mV) scar; the number of VTs induced; total ablation time and procedural details; transseptal vs retrograde aortic access; general anesthesia use; and hemodynamic support with intravenous vasopressor use.<br><br>The number of VTs per patient mapped and ablated in the group that recurred was significantly higher than in the group that did not recur.	Conclusion:<br>In ICM, a unipolar LVA surrounds the endocardial bipolar LVA, consistent with inhomogeneous scar with varying intramural extensions as observed on MRI. In this pilot study, the size of this area did not predict early recurrence after endocardial ablation. However, ablation of VT isthmuses and exits in this region was associated with a greater rate of early recurrence.<br><br>Limitations:<br>This is a retrospective pilot study of patients referred to a tertiary care center for VT ablation. The results might not be generalizable to a patient population undergoing VT ablation for the first time even though only 25% of patients in this cohort had prior ablations. They did not have cardiac MRI available to compare the size of the bipolar and unipolar LVA on the EAM, neither did they have

Study name or acronym; Author; Year published; PMID	Aim of study; Endpoints; Study type (RCT, observational — multicenter or single center; case series or other [if RCT include intervention and comparator]); Study size (N)	Patient population with inclusion and exclusion criteria	Results (absolute event rates, *P* values; OR or RR; 95% CI)	Other relevant findings or adverse events	Limitations; Other comments; Conclusions
			recurrent VT within 3 months (*P*=.3, Mann–Whitney test).<br><br>At VT isthmus/exit sites, 62 different VTs (a mean of 3.1 ± 1.4 VTs per patient) were induced during the VT ablation procedures.<br>Only 58% of the VTs (36 of 62) could be sufficiently mapped to elucidate their presumptive isthmus/exit sites. All ablations were confined to bipolar LVAs and its borders incorporating unipolar LVAs.<br><br>Of the 36 exit/isthmus sites identified, 19 (53%) were well inside the endocardial bipolar LVA (>1 cm from bipolar LVA border). The remaining presumptive VT isthmus/exits (17 of 36; 47%) were in the borders of the endocardial bipolar LVA (<1 cm from the bipolar LVA border). All 8		epicardial mapping largely due to frequent prior CABG and use of epicardial mapping only when endocardial mapping and ablation failed. They do not have the ECG morphologies of recurrent VTs to assess whether the recurrent VT was related to a border zone exit/isthmus, given these were terminated by ICDs. VT ablation targeted both isthmus and exit regions. Once one such region was identified, it was ablated. They cannot exclude the possibility that the reason for poorer outcomes when ablating in the periphery as opposed to the central LVA is not related to more challenging substrate, but rather to a failure to identify isthmus sites, which are typically located within the dense scar. By targeting exit sites in the periphery instead of isthmus sites in the core, recurrence rates would be expected to increase.

Study name or acronym; Author; Year published; PMID	Aim of study; Endpoints; Study type (RCT, observational — multicenter or single center; case series or other [if RCT include intervention and comparator]); Study size (N)	Patient population with inclusion and exclusion criteria	Results (absolute event rates, *P* values; OR or RR; 95% CI)	Other relevant findings or adverse events	Limitations; Other comments; Conclusions
			patients who had recurrent VT within 3 months had VT isthmus/exits in the bipolar LVA border area compared with only 3 of 12 of the group that did not recur (100% vs 25%; *P*<.05, Fisher exact test). Isthmus/exits were identified within the bipolar LVA area (<1.5 mV) in 11 of 12 patients without recurrent VT vs 4 of 8 of those with recurrent VT (*P*=ns).		

Study name or acronym; Author; Year published; PMID	Aim of study; Endpoints; Study type (RCT, observational — multicenter or single center; case series or other [if RCT include intervention and comparator]); Study size (N)	Patient population with inclusion and exclusion criteria	Results (absolute event rates, *P* values; OR or RR; 95% CI)	Other relevant findings or adverse events	Limitations; Other comments; Conclusions
Soto-Becerra et al. Ventricular tachycardia in the setting of Chagasic cardiomyopathy: use of voltage mapping to characterize endoepicardial nonischemic scar distribution.<br>• Year published: 2017<br>• PMID: 29133379	Aim: To characterize the LV scar distribution pattern specific to the CC and to evaluate the usefulness of endocardial unipolar voltage mapping to identify epicardial scar in a prospective series of patients with CC undergoing epicardial/endocardial LV tachycardia mapping and ablation.<br><br>A prospective observational study<br><br>N=19	Fifty-seven consecutive patients with CC, no atherosclerotic obstruction in the coronary arteries, no other cause of cardiomyopathy with recurrent VT undergoing EAM/ablation in both the epicardial and endocardial surfaces between July 2007 and February 2015.<br><br>The coronary angiography was performed before ablation in all patients.<br><br>On an anticipated prominent epicardial LV substrate and a poor long-term arrhythmia control with sole endocardial ablation in CC, the mapping/ablation protocol incorporated single procedural sequential epicardial and endocardial mapping in all patients.<br><br>Dense sampling of all epicardial/endocardial LV	The ablation procedure was considered successful: all induced VTs eliminated; no residual induction of any sustained VT in 11 (58%) patients. In the remaining 8,<br>no index VT had been induced. VT reinducibility after ablation was not assessed because of the patient's hemodynamic compromise at high risk for complication at operator discretion and based on published risk criteria.<br><br>After a follow-up of 13 (7–21) months, 3 (16%) patients experienced VT recurrence, of whom one underwent a second ablation procedure and 2 were maintained on amiodarone with isolated VT episodes. The matching process produced 1285 pairs of epicardial/endocardial points within the LV free wall. The subsequent	the median LV ejection fraction was 30% (25%–45%), 89% of patients had an intracardiac defibrillator, in 7, the indication for ablation was VT/ventricular fibrillation storm.<br>In 15 patients (79%), amiodarone could not be discontinued before the preliminary established 1-month period before ablation.<br>A total number of 34 VTs were induced,<br>- 6 (37%) patients having no inducible VT,<br>- 3 having (16%) 1 induced VT,<br>- the remaining 10 (53%) having ≥2 induced VTs.<br>- Epicardial ablation was needed to eliminate VT in 15 patients (79%),<br>- 13 of them undergoing combined epicardial/endocardial ablation (sole epicardial ablation in 2).<br>- The remaining 4 patients underwent ablation	Conclusions:<br>CC sets for a unique myocardial VT substrate. Predominance to the basal lateral LV, a more extensive epicardial than endocardial scarring, and a particularly dense and transmural scar are characteristic of this disease process. In this setting, unipolar endocardial EAM is useful to set for the suspicion of epicardial scar and to depict the area of epicardial bipolar abnormalities. Their results suggest that the unipolar endocardial cutoffs best predicting epicardial scar can be influenced by the nature of underlying cardiomyopathy and its scar transmural distribution.<br><br>Study limitations:<br>Aggregation of data of a paired nature into a single level of analysis potentially disregarded the 2 levels of dependency. Because manual revision of epicardial/endocardial pairs

Study name or acronym; Author; Year published; PMID	Aim of study; Endpoints; Study type (RCT, observational — multicenter or single center; case series or other [if RCT include intervention and comparator]); Study size (N)	Patient population with inclusion and exclusion criteria	Results (absolute event rates, *P* values; OR or RR; 95% CI)	Other relevant findings or adverse events	Limitations; Other comments; Conclusions
		regions (>100 points per surface) was considered compelling for inclusion.<br><br>Exclusion criteria: patients with a prior VT ablation, during ventricular pacing map.	random stratified sampling process produced 314 epicardial/endocardial pairs to be analyzed in a point-by-point fashion: 155 (49%) within epicardial bipolar scar and 159 (51%) within epicardial normal myocardium. The distance between pairs was 10 ± 3 mm, with no differences between pairs obtained from normal vs abnormal epicardium. Overall, epicardial electrograms showed lower bipolar voltages than endocardial electrograms:<br>(1.0 [0.3–2.8] mV versus 1.8 [0.6–3.5] mV; *P*<.001). The endocardial electrograms abutting epicardial scar had lower unipolar and bipolar voltages, longer electrogram duration, and were more fractionated than those abutting normal epicardium (*P*<.001).	exclusively from the endocardium.	was compelling for the comparison of epicardial versus endocardial electrogram characteristics and to assess for the usefulness of unipolar endocardial mapping, aggregation of data into a single level was considered the most operative strategy. The provided *P* values, therefore, are not adjusted for the intrasubject correlation. The randomly stratified sampling and a statistical test that takes into account the distinct variance of the measurements and the paired nature of the data aimed to compensate for this limitation. The limited number of patients included is a consequence of strict inclusion criteria. The specificity values of unipolar endocardial voltage mapping might be limited by the underrepresentation of myocardial areas with abnormal endocardium and normal epicardium because the vast majority of epicardial

Study name or acronym; Author; Year published; PMID	Aim of study; Endpoints; Study type (RCT, observational — multicenter or single center; case series or other [if RCT include intervention and comparator]); Study size (N)	Patient population with inclusion and exclusion criteria	Results (absolute event rates, *P* values; OR or RR; 95% CI)	Other relevant findings or adverse events	Limitations; Other comments; Conclusions
			Confluent epicardial/endocardial scar was suggested by a high correspondence ratio between epicardial and endocardial low-voltage signals: the majority of abnormal endocardial bipolar electrograms (108 of 133; 79%) were opposite to abnormal bipolar epicardial; most abnormal epicardial electrograms (110 of 155; 71%) were opposite to abnormal bipolar endocardium. The Spearman coefficient analysis confirmed moderate but positive correlation between the endocardial unipolar voltages and the epicardial bipolar voltages (ρ=.6173; *P*<.001). A logistic regression model indicated that the endocardial unipolar voltage was an independent predictor of epicardial scar (P<0.001). The endocardial bipolar		normal bipolar voltages had normal bipolar voltages at the endocardial counterpart. Additionally, use of the conventional <1-mV cutoff to define abnormal epicardium underestimates the extent of CC epicardial scar. The criterion they used to define dense and transmural scarring (confluent epicardial/endocardial areas with median bipolar voltages of ≤0.5 mV), although based on comparative MRI/pathological/electroanat omic data, can be considered an assumption: intramural layers of preserved myocardium might still be present. The proposed unipolar voltage cutoff was developed in patients with CC with high prevalence of LV basal inferolateral scars; therefore, it cannot be extrapolated either to other CC substrate locations or to ischemic or other NICM pathogeneses. A majority of patients (79%) were on amiodarone at the time of

Study name or acronym; Author; Year published; PMID	Aim of study; Endpoints; Study type (RCT, observational — multicenter or single center; case series or other [if RCT include intervention and comparator]); Study size (N)	Patient population with inclusion and exclusion criteria	Results (absolute event rates, *P* values; OR or RR; 95% CI)	Other relevant findings or adverse events	Limitations; Other comments; Conclusions
			voltage was also predictive of epicardial scar (*P*=.010).<br><br>The ROC curve analysis of endocardial unipolar voltage demonstrated an AUC of 0.80 (95% CI, 0.76–0.85) to detect epicardial bipolar scar, using a ≤4.0-mV cutoff for the endocardial unipolar voltage, sensibility of 71%, and specificity of 75%, with a 55% (range, 26–57%) overlap between endocardial unipolar abnormal and epicardial bipolar abnormal areas.		the procedure. The use of additional electrogram criteria to define epicardial bipolar scar (≤1 mV plus split/wide/late potentials) produced a selection bias in the analysis of epicardial versus endocardial voltage characteristics favoring the predominance of epicardial LPs, with no influence in the bipolar scar area measurements.

Study name or acronym; Author; Year published; PMID	Aim of study; Endpoints; Study type (RCT, observational — multicenter or single center; case series or other [if RCT include intervention and comparator]); Study size (N)	Patient population with inclusion and exclusion criteria	Results (absolute event rates, *P* values; OR or RR; 95% CI)	Other relevant findings or adverse events	Limitations; Other comments; Conclusions
Desjardins et al. Characteristics of intramural scar in patients with nonischemic cardiomyopathy and relation to intramural ventricular arrhythmias.<br>• Year published: 2013<br>• PMID: 23985383	Aim: To assess the value of voltage mapping to detect MRI-defined intramural scar and to correlate the scar with ventricular arrhythmias.<br><br>A single-center observational study.<br><br>N=15	The patients were from among a pool of 28 consecutive patients with NICM referred for ablation of VAs and who underwent MRI and had DE on MRI. Only patients with scar that did not reach the endocardium were selected for this analysis.<br><br>The subjects of this study were 15 consecutive patients (3 women, age: 55 ± 16 years, EF 49% ± 13%) with NICM referred for RFCA of symptomatic VAs:<br>VT (n=7); PVCs (n=6); both (n=2), in whom a predominant intramural scar was detected on DE-MRI.<br><br>Four patients had cardiac sarcoidosis and 11 patients had IDCM.<br><br>The patients failed 1 ± 1 AADs.<br>None of the patients had	Intramural scar had a volume of 11.7 ± 8 cm^3 and an area projecting on the endocardium of 19.3 ± 13.4 cm^2. the distance of the intramural scar from the endocardial surface was 3.3 ± 1.6 mm. The scar was located in the septum in 8 of 15, in the free wall in 8 of 15, in a basal left ventricle in 8 patients, and was multifocal in 3 patients.<br><br>The mean bipolar voltage overlying the intramural scar was 1.62 ± 1.73 mV and the unipolar endocardial voltage overlying the intramural scar was 4.49 ± 3.25 mV. An area of 1 cm^2 surrounding the intramural scar showed a mean bipolar voltage of 2.12 ± 2.15 mV. Unipolar voltage in the scar-free area was 6.59 ± 3.81 mV. Both bi- and unipolar voltage overlying the scar	During follow-up, the PVC burden in patients with frequent PVCs was reduced from 28% ± 13% to 0.9% ± 0.3% in patients with effective procedures (*P*<.01). Three patients with VT and 2 patients with PVCs had recurrences of their arrhythmias. One of 3 VTs was intramural and 1 of 2 patients with PVCs had an intramural site of origin.	Conclusion:<br>This report shows that intramural scar harbors VAs. Intramural scar can be precisely located by DE-MRI. Endocardial mapping with the use of bipolar and unipolar voltage can be used to identify intramural scar. However, voltage mapping cannot precisely define the borders of intramural scar. A cardiac MRI is more accurate than a voltage map for characterization of intramural scar. Registration of intramural scar helps to identify a region of interest on which the mapping procedure can focus. The incremental value of scar registration for ablating intramural VAs remains to be determined.<br><br>Limitations:<br>The study is limited by its small sample size. Only patients with IDCM and cardiac sarcoidosis were included, and the findings might not apply to all patients

Study name or acronym; Author; Year published; PMID	Aim of study; Endpoints; Study type (RCT, observational — multicenter or single center; case series or other [if RCT include intervention and comparator]); Study size (N)	Patient population with inclusion and exclusion criteria	Results (absolute event rates, *P* values; OR or RR; 95% CI)	Other relevant findings or adverse events	Limitations; Other comments; Conclusions
		an ICD at the time of the MRI. All but 2 patients with VT subsequently had an ICD implanted. One patient refused ICD implantation,<br>the other patient VT was hemodynamically tolerated and became noninducible postablation.<br><br>Eight patients had VT and 7 patients had frequent PVCs, with a PVC burden of 21% ± 12 %. When distinguishing different VT or PVC morphologies, the following aspects of the QRS complex were assessed: BBB morphology, axis, amplitude, and QRS notching.	was significantly lower than the bi- and unipolar voltage at a distance of 1 cm from the scar (*P*<.0001). More remote areas had significantly higher bi- and unipolar voltage (2.83 ± 2.34 and 8.32 ± 3.39 mV respectively, both *P*<.0001). By ROC curves, the bipolar cutoff voltage that best separated endocardial sites overlying intramural scar compared with sites without intramural scar was 1.55 mV (AUC 0.69, sensitivity 61%, specificity 66%). The unipolar cutoff was 6.78 mV (AUC: 0.78, sensitivity 76%, specificity 69%). The bootstrapped 95% CI for the bipolar voltage cutoff was 1.33 and 1.71; for unipolar voltage 6.28 and 7.26.		with NICM. A distinction between intramural and epicardial scar was not attempted in this patient population. the scar was predominantly intramural by MRI in all patients. The intramural scar extended to the epicardium in one patient, and VTs were ablated from the epicardium in this patient. Critical sites for VAs were not identified in all patients; therefore, it is unclear whether failure of ablation was due to an intramural location of the arrhythmia or due to another site of origin.<br>It is possible that longer applications of RF energy delivery than used in this study might have resulted in improved outcomes of ablation of intramural VAs.<br>Systematic mapping of both aspects of the ventricular septum containing the intramural scar was not performed in this study.<br>A major limitation of the unipolar voltage data is that

Study name or acronym; Author; Year published; PMID	Aim of study; Endpoints; Study type (RCT, observational — multicenter or single center; case series or other [if RCT include intervention and comparator]); Study size (N)	Patient population with inclusion and exclusion criteria	Results (absolute event rates, *P* values; OR or RR; 95% CI)	Other relevant findings or adverse events	Limitations; Other comments; Conclusions
					there is a substantial overlap of unipolar low voltage between scar zones and regions of no scar. This makes it difficult to precisely define the border of the intramural scar. A cardiac MRI with delayed enhancement remains the gold standard for exact demarcation of scars and therefore is preferable over voltage mapping for defining intramural scar.

Intraprocedural Imaging: Intracardiac Echocardiography, Fluoroscopy, Cardiac Magnetic Resonance Imaging

Study name or acronym; Author; Year published; PMID	Aim of study; Endpoints; Study type (RCT, observational — multicenter or single center; case series or other [if RCT include intervention and comparator]); Study size (N)	Patient population with inclusion and exclusion criteria	Results (absolute event rates, *P* values; OR or RR; 95% CI)	Other relevant findings or adverse events	Limitations; Other comments; Conclusions
**ICE and PAP VAs**					
Good et al. Ventricular arrhythmias originating from a papillary muscle in patients without prior infarction: a comparison with fascicular arrhythmias.<br>• Year published: 2008<br>• PMID: 18984528	Aim: To report on ablation on targeting of PAP arrhythmias<br>Endpoint: VA elimination<br>Study type: Observational, case series	9 patients<br><br>Inclusion criteria: PAP VA from ALPAP or PMPAP	All VAs ablated. Compared with fascicular VAs; PAP VAs can be distinguished electrocardiographically. ICE helpful in targeting PAP VAs.	All VAs ablated. Compared with fascicular VAs, PAP VAs can be distinguished electrocardiographically. ICE helpful in targeting PAP VAs.	ICE is useful to identify the SOO of PAP VAs and might be beneficial for mapping them.
Yamada et al. Idiopathic focal ventricular arrhythmias originating from the anterior papillary muscle in the left ventricle.<br>• Year published: 2009<br>• PMID: 19298560	Aim: To report on ablation of PAP VAs<br>Endpoint: VA elimination, noninducibility<br>Study type: Observational, case series	6 patients<br><br>Inclusion criteria: PAP VA from anterolateral PAP	VAs in all 6 patients ablated. ICE used in 4 patients; recurrence of VAs from same location in 4 of 6 patients in <3 weeks.	Conventional RF failed acutely in 2 of 6 patients requiring irrigated tip or 8 mm tip catheters.	ICE not used to target PAPs, but to confirm that VAs originate from PAPs.
Crawford et al. Ventricular arrhythmias originating from papillary muscles in the right ventricle.<br>• Year published: 2010<br>• PMID: 20206325	Aim: To report on mapping and ablation of RV PAP VAs<br>Endpoint: VA elimination<br>Study type: Observational, case series	8 patients<br><br>Inclusion criteria: RV PAP VAs	ICE used to target VAs on PAP in all patients; SOO on PAP: anterior, 4; posterior, 3; septal, 8.	MRI normal in all patients; PPs at SOO in 2 of 8 patients.	ICE useful to target RV PAP VAs.

Study name or acronym; Author; Year published; PMID	Aim of study; Endpoints; Study type (RCT, observational — multicenter or single center; case series or other [if RCT include intervention and comparator]); Study size (N)	Patient population with inclusion and exclusion criteria	Results (absolute event rates, *P* values; OR or RR; 95% CI)	Other relevant findings or adverse events	Limitations; Other comments; Conclusions
Yokokawa et al. Predictors of successful catheter ablation of ventricular arrhythmias arising from the papillary muscles.<br>• Year published: 2010<br>• PMID: 20637311	Aim: To describe predictors for successful ablation of PAP VAs<br>Endpoint: Comparison of effective vs failed ablations.<br>Study type: Observational, case series	40 patients<br><br>Inclusion criteria: PAP VAs	Some 31 of 40 patients had successful ablation. PP at SOO and matching pace maps predicted successful ablation. Smaller PAP mass (on MRI) in patients with successful ablation.	ICE was used to identify PAPs and confirm catheter stability during RF.	ICE useful in confirming catheter stability during RF delivery.
Yamada et al. Idiopathic ventricular arrhythmias originating from the papillary muscles in the left ventricle: prevalence, electrocardiographic and electrophysiological characteristics, and results of the radiofrequency catheter ablation.<br>• Year published: 2010<br>• PMID: 19793147	Aim: To distinguish PAP VAs from other VAs<br>Endpoint: VA elimination<br>Study type: Observational, case series	19 patients with PAP VAs<br><br>Inclusion criteria: PAP, MA, fascicular VAs (total 71 patients).	PAP VAs can be distinguished by ECG from fascicular and MA VAs.	ICE used in 13 patients; ICE demonstrated catheter stability during ablation.	ICE useful in confirming catheter stability during RF delivery.

Study name or acronym; Author; Year published; PMID	Aim of study; Endpoints; Study type (RCT, observational — multicenter or single center; case series or other [if RCT include intervention and comparator]); Study size (N)	Patient population with inclusion and exclusion criteria	Results (absolute event rates, *P* values; OR or RR; 95% CI)	Other relevant findings or adverse events	Limitations; Other comments; Conclusions
Yamada et al. Electrocardiographic and electrophysiological characteristics in idiopathic ventricular arrhythmias originating from the papillary muscles in the left ventricle: relevance for catheter ablation.<br>• Year published: 2010<br>• PMID: 20558848	Aim: To describe mapping and ablation in PAP VAs<br>Endpoint: VA elimination	19 patients with PAP VAs	Spontaneous pleomorphic PVCs in 9 of 19 patients;<br>in 7 of 9 patients, RF from both sides of PAP required.	ICE used in 13 patients; in patients with failed RF, epicardial ablation also failed; changing morphologies during RF in 17/19 patients; 9 patients required a second procedure due to recurrence of targeted PVC.	ICE useful in assessing catheter location relative to PAPs.
Bassil et al. Comparison of robotic magnetic navigation-guided and manual catheter ablation of ventricular arrhythmias arising from the papillary muscles.<br>• Year published: 2018<br>• PMID: 29722854	Aim: To compare manual vs magnetic guided navigation of catheter ablation for PAP VAs<br>Endpoint: VA elimination<br>Study type: Observational, case series	35 patients with PAP VAs<br><br>Inclusion criteria: PAP VAs	Success rate is similar in manual vs magnetic navigation (73% vs 74%); lower fluoroscopy in magnetic navigation.	ICE was used to determine catheter position relative to PAPs.	No comment on whether IC was useful or not to map and ablate VAs.

Study name or acronym; Author; Year published; PMID	Aim of study; Endpoints; Study type (RCT, observational — multicenter or single center; case series or other [if RCT include intervention and comparator]); Study size (N)	Patient population with inclusion and exclusion criteria	Results (absolute event rates, *P* values; OR or RR; 95% CI)	Other relevant findings or adverse events	Limitations; Other comments; Conclusions
Ban et al. Electrophysiological characteristics related to outcome after catheter ablation of idiopathic ventricular arrhythmia originating from the papillary muscle in the left ventricle.<br>• Year published: 2013<br>• PMID: 24385992	Aim: To assess reason for PAP VA recurrence after ablation<br>Endpoint: VA elimination<br>Study type: Observational, case series	12 patients with PAP VAs<br><br>Inclusion criteria: PAP VAs	There were 8 patients without and 4 patients with recurrence. Patients with a successful outcome had high amplitude potentials at SOO; patients with recurrence had low-amplitude signals at presumed SOO. Echocardiography was used: TTE, TEE and ICE, not specified how often; ICE was not integrated in the mapping system.	ICE not considered beneficial in identifying location of catheter tip and was not integrated into the mapping system.	
Rivera et al. Results of cryoenergy and radiofrequency-based catheter ablation for treating ventricular arrhythmias arising from the papillary muscles of the left ventricle, guided by intracardiac echocardiography and image integration.<br>• Year published: 2016<br>• PMID: 27069089	Aim: To compare cryoablation vs RF ablation for PAP VAs<br>Endpoint: VA elimination<br>Study type: Observational, case series	21 patients with PAP VAs<br><br>Inclusion criteria: PAP VAs<br><br>Sequential design: 12 patients treated with cryoablation and 9 patients with RF ablation by different operator. ICE and CT data were used for image integration.	Patients with cryoablation therapy had higher acute success (*P*=.08), better catheter stability (*P*=.001), less change in QRS morphologies postablation (*P*=.01), and a lower recurrence rate (*P*=.03).	Cryoablation for PAP VAs showed better catheter stability, a lower recurrence rate, and requires transseptal access with a 15 Fr sheath.	Limitation: only monomorphic PVCs mapped.<br><br>ICE helpful in directing cryoablation catheter.

Study name or acronym; Author; Year published; PMID	Aim of study; Endpoints; Study type (RCT, observational — multicenter or single center; case series or other [if RCT include intervention and comparator]); Study size (N)	Patient population with inclusion and exclusion criteria	Results (absolute event rates, *P* values; OR or RR; 95% CI)	Other relevant findings or adverse events	Limitations; Other comments; Conclusions
Proietti et al. Intracardiac echo-facilitated 3D electroanatomical mapping of ventricular arrhythmias from the papillary muscles: assessing the 'fourth dimension' during ablation.<br>• Year published: 2017<br>• PMID: 27485578	Aim: To assess use of ICE for targeting PAP VAs<br>Endpoint: VA elimination<br>Study type: Observational, case series	16 patients with PAP VAs<br><br>Inclusion criteria: PAP VAs with and without SHD<br><br>A total of 24 procedures; 3D ICE was used in 14 of 24 procedures	A higher success rate was attained if 3D ICE was used during the first procedure.	ICE beneficial in identifying PAPs and mapping of PAPs.	ICE beneficial for real-time imaging and mapping of PAP VAs.
Peichl et al. The tip of the muscle is a dominant location of ventricular ectopy originating from papillary muscles in the left ventricle.<br>• Year published: 2018<br>• PMID: 28884872	Aim: To correlate PAP VA origin with outcomes postablation	34 patients<br><br>Inclusion criteria: PAP VAs	PAP VAs were eliminated in 86%; long-term success was achieved in 67%; PAP VAs originated from distal, mid, and basal PAPs in 67%, 19%, and 14%, respectively; monomorphic PVCs had better outcome.	ICE crucial for catheter manipulation and to confirm catheter stability; chordal rupture in 1 patient resulted in worsening mitral regurgitation.	ICE crucial for catheter manipulation and to confirm catheter stability.
Lee et al. Catheter ablation of papillary muscle arrhythmias: Implications of mitral valve prolapse and systolic dysfunction.<br>• Year published: 2018<br>• PMID: 29732567	Aim: To determine the association of PAP VAs with MVP<br>Endpoint: VA elimination<br>Study type: Observational, case series	23 patients<br><br>Inclusion criteria: patients with PAP VAs; all 3D ICE with image integration into EAM	MVP more frequent in PAP VAs compared with the control group (9 of 23 patients vs 0 of 129 patients, *P*<.01).	One patient died after 2 ablations, who had MVP (bileaflet); association of PAP VAs and MVP.	Presence of MVP does not affect ablation outcomes; ICE useful to target PAP VAs.

Study name or acronym; Author; Year published; PMID	Aim of study; Endpoints; Study type (RCT, observational — multicenter or single center; case series or other [if RCT include intervention and comparator]); Study size (N)	Patient population with inclusion and exclusion criteria	Results (absolute event rates, *P* values; OR or RR; 95% CI)	Other relevant findings or adverse events	Limitations; Other comments; Conclusions
**ICE and Aortic Cusp Ablation**					
Hachiya et al. How to diagnose, locate, and ablate coronary cusp ventricular tachycardia.<br>• Year published: 2002<br>• PMID: 12108495	Aim: To describe ablation of VAs originating from the aortic cusps<br>Endpoint: VA elimination<br>Study type: Observational, case series	15 patients<br><br>Inclusion criteria: patients with aortic cusp VAs; coronary angiogram in all patients	Some 14 of 15 VAs were successfully ablated with the catheter >1 cm from the coronary ostia; 1 patient was not ablated because catheter was <1 cm from the coronary ostium.	Successful ablation in all patients without complications when SOO >1 cm from the coronary ostium.	Coronary angiogram recommended; ablation safe if SOO >1 cm from the coronary ostium.
Ouyang et al. Repetitive monomorphic ventricular tachycardia originating from the aortic sinus cusp: electrocardiographic characterization for guiding catheter ablation.<br>• Year published: 2002<br>• PMID: 11823089	Aim: To describe characteristics and ablation of VAs originating from aortic cusps<br>Endpoint: Elimination of VA<br>Study type: Observational, case series	15 patients<br><br>Inclusion criteria: Patients with repetitive MMVT or frequent PVCs; coronary angiogram prior to ablation in aortic cusps.	RF in aortic cusps eliminated cusp VAs; distance from ablation site to coronary ostium (left main): 7.3–16.1 mm.	ECG (R wave width and R/S amplitude in V1, V2) can distinguish cusp origin from RVOT origin.	Ablation in cusps safe if SOO is ≥7.3 mm from coronary ostium.

Study name or acronym; Author; Year published; PMID	Aim of study; Endpoints; Study type (RCT, observational — multicenter or single center; case series or other [if RCT include intervention and comparator]); Study size (N)	Patient population with inclusion and exclusion criteria	Results (absolute event rates, *P* values; OR or RR; 95% CI)	Other relevant findings or adverse events	Limitations; Other comments; Conclusions
Yamada et al. Idiopathic ventricular arrhythmias originating from the aortic root prevalence, electrocardiographic and electrophysiologic characteristics, and results of radiofrequency catheter ablation.<br>• Year published: 2008<br>• PMID: 18598894	Aim: To describe characteristics of aortic root VAs<br>Endpoint: Elimination of VAs in relation to coronary ostia<br>Study type: Observational, case series	44 patients<br><br>Inclusion criteria: Patients with aortic cusp VAs; cath performed in all patients prior to RF in cusps; RF not performed within 5 mm of coronary ostia.	All 44 VAs were eliminated; there was a temporary complete AV block in 1 patient when RF was performed from the RCC; no other complications.	Cusp VAs originate from the LCC>RCC>NCC; ratio of leads II/II can distinguish L vs R cusp; distance of ablation site to coronary ostia ranged from 7.5–17.2 mm in the LCC and 8.3–18.1 mm in the RCC.	Aortic cusp VAs can safely be ablated when the distance between the ablation site and the coronary ostia is ≥7.5 mm.
Hoffmayer et al. Safety of radiofrequency catheter ablation without coronary angiography in aortic cusp ventricular arrhythmias.<br>• Year published: 2014<br>• PMID: 24732373	Aim: To assess whether RF ablation of cusp VAs can be safely performed with ICE but no coronary angiography<br>Endpoint: Elimination of VA if SOO ≥1 cm from coronary ostia<br>Study type: Observational, case series	35 patients<br><br>Inclusion criteria: Patients with aortic cusp VAs; ICE performed in all patients; RF performed if SOO >1.0 cm from coronary ostia.	Some 29 of 35 VAs were successfully ablated, with no complications. Coronary angiography was performed in 3 cases where the SOO was <1 cm but >5 mm from the coronary ostia when ablation was performed.	RF of cusp VAs is safe if the catheter by ICE assessment is >1 cm from the coronary ostia; coronary angiogram recommended if distance of SOO to coronary ostia is <1 cm.	Distance of SOO to coronary ostia can be determined by ICE, and if distance is >1 cm coronary angiogram can be avoided.
**Angiography for VA Epicardial Ablation**					

Study name or acronym; Author; Year published; PMID	Aim of study; Endpoints; Study type (RCT, observational — multicenter or single center; case series or other [if RCT include intervention and comparator]); Study size (N)	Patient population with inclusion and exclusion criteria	Results (absolute event rates, *P* values; OR or RR; 95% CI)	Other relevant findings or adverse events	Limitations; Other comments; Conclusions
Baman et al. Mapping and ablation of epicardial idiopathic ventricular arrhythmias from within the coronary venous system.<br>• Year published: 2010<br>• PMID: 20400776	Aim: To describe ablation of epicardial PVCs from the CVS<br>Endpoint: Eliminate VAs<br>Study type: Observational, case series	27 patients<br>Inclusion criteria: Patients with epicardial PVCs; coronary angiogram performed in all patients pre- and postablation; RF delivered if SOO ≥4 mm from major coronary artery.	PVCs were eliminated in 74% of patients, with no complications; in 4 patients, SOO was not reachable with the catheter; in 1 patient, the SOO was too close to a coronary artery.		RF ablation of epicardial VAs is safe and moderately effective; coronary angiography beneficial to assess distance between ablation catheter and coronary arteries.
Yokokawa et al. Ablation of epicardial ventricular arrhythmias from nonepicardial sites.<br>• Year published: 2011<br>• PMID: 21703218	Aim: To systematically analyze the contribution of ablation at sites other than the epicardium to eliminate an arrhythmia originating in the epicardium	In a consecutive patient series of 33 patients (14 women, age 51 ± 14 years, EF 51% ± 9%) with epicardial VAs, mapping and ablation was performed via the CVS/pericardial space, the aortic sinus cusp, and the LV endocardium. An arrhythmia was defined as epicardial if the earliest onset of activation and a matching pace map (≥10 of 12 leads) were identified in the epicardium.	In 12 of 33 patients (36%), either an endocardial approach alone (n=3) or a combined endocardial/epicardial (n=6), cusp/endocardial (n=1), or cusp/epicardial (n=2) approach was required to eliminate the VAs. In 10 of 33 (30%) patients, epicardial ablation alone was effective in eliminating epicardial VAs. Ablation was ineffective due to failure to reach the site of origin with the ablation catheter in 5 of 33 (15%) patients, the SOO was too close to an epicardial artery or the phrenic nerve in 3 (6%) patients, and power delivery was insufficient in 3 (9%) patients.	About one-third of epicardial arrhythmias require ablation from sites other than the epicardium to eliminate the arrhythmia focus.	

Study name or acronym; Author; Year published; PMID	Aim of study; Endpoints; Study type (RCT, observational — multicenter or single center; case series or other [if RCT include intervention and comparator]); Study size (N)	Patient population with inclusion and exclusion criteria	Results (absolute event rates, *P* values; OR or RR; 95% CI)	Other relevant findings or adverse events	Limitations; Other comments; Conclusions
Sacher et al. Epicardial ventricular tachycardia ablation: a multicenter safety study.<br>• Year published: 2010<br>• PMID: 20488308	Aim: To assess safety of epicardial VT ablation<br>Endpoint: VT elimination<br>Study type: Multicenter, observational case series	156 epicardial VT ablation procedures in 134 patients; coronary angiogram prior to ablation in most but not all patients.	A surgical approach was used in 14 patients; 8 major complications: 7 epicardial bleeding, 1 coronary stenosis (after cryoablation); 3 delayed complications: 1 inflammatory reaction, 1 delayed tamponade (10 days post-RF), 1 delayed occlusion (ARVC-RF RV free wall, no prior catheter ablation, unusual coronaries).	Epicardial bleeding occurred in 17% of patients (≥80 ccs = major bleeding).	Coronary angiography important to minimize coronary artery-related risks; cryoablation in close proximity to coronaries, not safe.
**ICE and Substrate/Thrombus/Identification/Effusion/Steam Pop**					
Bala et al. Assessing epicardial substrate using intracardiac echocardiography during VT ablation.<br>• Year published: 2011<br>• PMID: 21880675	Aim: Can ICE assess epicardial scar in patients with NICM?<br>Endpoint: Compare EAM and/or MRI data with ICE data; coronary ostia<br>Study type: Observational, case series	18 patients<br><br>Inclusion criteria: only NICM; ICE performed in all patients; MRI performed in 8 patients.	Increased echogeneity found in all 18 patients, which corresponds to low voltage on EAM and scar on MRI. Epicardial EAM showed smaller LVA compared with scar on MRI or area of increased echogeneity on ICE.		MRI only in 8 patients, and there was significant artifact in 2 of the 8 patients; ICE helps to identify epicardial and intramural scarring.
Peichl et al. Catheter ablation of ventricular tachycardia in the presence of an old endocavitary thrombus guided by intracardiac echocardiography.<br>• Year published: 2016<br>• PMID: 26969894	Aim: To analyze outcomes in patients with thrombi undergoing VT ablation<br>Endpoint: Assess outcomes<br>Study type: Observational, case series	8 patients (TTE preprocedural: n=4), ICE intraprocedural: n=4) of 344 patients.<br><br>Inclusion criteria: Presence of SHD; uninterrupted anticoagulation with transseptal approach.	Endocardial mapping was performed in all patients; epicardial mapping was performed in in 3 of 8 patients; VT had recurred in 2 of 8 patients at 14 months' follow-up.	No thromboembolic complications; waiting time from thrombus detection procedure unclear.	ICE was more accurate than TTE for thrombus detection; mapping/ ablation in presence of thrombus without mobile components was possible; waiting time from thrombus detection-procedure unclear.

Study name or acronym; Author; Year published; PMID	Aim of study; Endpoints; Study type (RCT, observational — multicenter or single center; case series or other [if RCT include intervention and comparator]); Study size (N)	Patient population with inclusion and exclusion criteria	Results (absolute event rates, *P* values; OR or RR; 95% CI)	Other relevant findings or adverse events	Limitations; Other comments; Conclusions
Feigenbaum et al. Ultrasound diagnosis of pericardial effusion.<br>• Year published: 1965<br>• PMID: 14245510	Aim: To analyze echocardiographic appearance of fluid in dogs and humans<br>Study type: animal study, case studies	5 dogs, 2 normal probands, 1 patient with cardiac tamponade	Detection of pericardial fluid by ultrasound in dogs/humans; normalization of findings after fluid removal.		Ultrasound can detect pericardial fluid accumulation.
Nguyen et al. Use of tissue electric and ultrasound characteristics to predict and prevent steam-generated cavitation during high-power radiofrequency ablation.<br>• Year published: 2018<br>• PMID: 30067489	Aim: Assessment of predictors of steam pops<br>Endpoint: description of electric and ultrasonographic characteristics preceding steam pops.<br>Study type: ex vivo bovine model, in vivo porcine model, case series	Bovine thigh preparation covered with circulating blood; bovine ex vivo heart preparation; retrospective analysis of case series in which ablation was performed for SVT and VT.	Increase in tissue echogeneity, especially 5 seconds prior to steam pop.	Other factors predicting steam pops include total impedance reduction and rate of impedance reduction.	Echocardiography can be helpful to detect tissue changes prior to occurrence of steam pops.
Filgueiras-Rama et al. Utility of intracardiac echocardiography for catheter ablation of complex cardiac arrhythmias in a medium-volume training center.<br>• Year published: 2015<br>• PMID: 25109241	Aim: To assess utility and safety of ICE during catheter ablation<br>Endpoint: Assessment for complications<br>Study type: Observational	110 ablation procedures for atrial arrhythmias and VT	Detection of pericardial effusion: 13 (11.8%); tamponade: 4 (36%). Puncture, drainage, and protamine administration required in all severe effusions (>1 cm).	Thrombus formation observed in 1 patient with LV dysfunction despite anticoagulation, which resulted in a stroke. RF delivery resulted in increased echodensity at the ablation sites, and edema formation was observed in 2 VT ablation procedures. Steam pops were observed in 2 patients.	ICE helps in early detection of potential complications.

**Study name or acronym; Author; Year published; PMID**	**Aim of study; Endpoints; Study type (RCT, observational — multicenter or single center; case series or other [if RCT include intervention and comparator]); Study size (N)**	**Patient population with inclusion and exclusion criteria**	**Results (absolute event rates, *P* values; OR or RR; 95% CI)**	**Other relevant findings or adverse events**	**Limitations; Other comments; Conclusions**
Weintraub et al. Intracardiac two-dimensional echocardiography in patients with pericardial effusion and cardiac tamponade.<br>• Year published: 1991<br>• PMID: 1760178	Aim: Utility of 2D ICE to detect pericardial effusion and tamponade.	ICE used in 5 patients with effusion	ICE identified pericardial effusion in all 5 patients; RA collapse was identified in 2 patients; reduction in pericardial fluid was observed in all patients after pericardiocentesis as well as increase in RA size.	The entire right atrium could not be assessed with the 20 MHz probe; easy manipulation of the echoprobe in the right atrium.	2D ICE helps to detect pericardial effusion and tamponade.

Electroanatomical Mapping Systems and Robotic Navigation

Study name or acronym; Author; Year published; PMID	Aim of study; Endpoints; Study type (RCT, observational — multicenter or single center; case series or other [if RCT include intervention and comparator]); Study size (N)	Patient population with inclusion and exclusion criteria	Results (absolute event rates, *P* values; OR or RR; 95% CI)	Other relevant findings or adverse events	Limitations; Other comments; Conclusions
Zhang et al. Magnetic vs manual catheter navigation for mapping and ablation of right ventricular outflow tract ventricular arrhythmias: a randomized controlled study.<br>• Year published: 2013<br>• PMID: 23692891	Procedure time<br>Fluoroscopy time<br>Ablation success<br>Complications<br>RCT<br>RMN vs manual<br>N=30<br>15 of 15	RVOT PVC<br>RVOT VT	Procedure times similar; fluoroscopy times 68% lower; success in 14 of 15 manual, 10 of 15 RMN.	No major complications	Small size; used noncontact mapping system
Skoda et al. Catheter ablation of ischemic ventricular tachycardia with remote magnetic navigation: STOP-VT multicenter trial.<br>• Year published: 2016<br>• PMID: 26969220	Noninducibility<br>VT free at 12 months<br>Multicenter (4), prospective, single-arm RMN<br><br>67 years, 93% male, 32% LVEF, 60% amiodarone<br>N=53	Ischemic VT	94% target VT noninducible; 73% all VT noninducible; 62% VT-free at 12 months.	No complications; 10% death rate at 12 months	

Study name or acronym; Author; Year published; PMID	Aim of study; Endpoints; Study type (RCT, observational — multicenter or single center; case series or other [if RCT include intervention and comparator]); Study size (N)	Patient population with inclusion and exclusion criteria	Results (absolute event rates, *P* values; OR or RR; 95% CI)	Other relevant findings or adverse events	Limitations; Other comments; Conclusions
Shauer et al. Clinical research: remote magnetic navigation vs manually controlled catheter ablation of right ventricular outflow tract arrhythmias: a retrospective study.<br>• Year published: 2018<br>• PMID: 29722855	Procedural time<br>Fluoroscopy time<br>Procedural success<br>Complications<br>Multicenter (2), Retrospective comparison<br>N=75<br>42 RMN vs 47 MCC	RVOT PVCs and VT	Procedural time was 113 ± 53 minutes in the RMN vs 115 ± 69 minutes in MCC (*P*=.90). Total fluoroscopic time was 10.9 ± 5.8 vs 20.5 ± 13.8 (*P*<.05). Acute procedural success rate was 80% in RMN vs 74% in MCC group (*P*=.46).<br>After a median follow-up of 25 months (IQR 13–34), the success rate remained 55% in the RMN group and 53% in MCC (*P*=.96).	Two complications in RMN group and 5 in MCC (*P*=.43).	Balanced Carto vs NavX use.
Kawamura et al. Comparison of remote magnetic navigation ablation and manual ablation of idiopathic ventricular arrhythmia after failed manual ablation.<br>• Year published: 2017<br>• PMID: 27314679	Procedural success<br>Fluoroscopy time<br>Complications	Idiopathic VA, repeat procedure	Higher success (91% RMN vs 69% MCC; *P*=.02). Fluoroscopy was 17 ± 12 minutes RMN vs 43 ± 18 minutes MCC (*P*=.009).	No major complications	Success in posterior RVOT plus posterior TA was higher with RMN than MCC (92% vs 50%, respectively; *P*=.03).

Study name or acronym; Author; Year published; PMID	Aim of study; Endpoints; Study type (RCT, observational — multicenter or single center; case series or other [if RCT include intervention and comparator]); Study size (N)	Patient population with inclusion and exclusion criteria	Results (absolute event rates, *P* values; OR or RR; 95% CI)	Other relevant findings or adverse events	Limitations; Other comments; Conclusions
Jin et al. Acute and long-term outcomes of catheter ablation using remote magnetic navigation for the treatment of electrical storm in patients with severe ischemic heart failure.<br>• Year published: 2015<br>• PMID: 25662047	Acute success<br>Procedure time<br>Fluoroscopy time<br>Complications<br>Retrospective, single-arm<br>N=40	ES	Acute success 80%; procedure time and fluoroscopy time were 105 ± 27 minutes and 7.5 ± 4.8 minutes, respectively. During a mean follow-up of 17.4 months, 19 (47.5%) patients remained free of VT recurrence, with reduced ICD shock burden (4.3 vs 1.9 per year; *P*<.05).	No major complications occurred during procedures	
Dinov et al. Long-term efficacy of single procedure remote magnetic catheter navigation for ablation of ischemic ventricular tachycardia: a retrospective study.<br>• Year published: 2012<br>• PMID: 22313170	Acute success<br>Procedure time<br>Fluoroscopy time<br>Complications<br>Retrospective comparison<br>N=102<br>RMN 50 vs 52	Ischemic VT	Acute success RMN 82% vs 71% MCC, *P*=.246. RMN had shorter fluoroscopy time than MCC (13 ± 12 minutes vs 32 ± 17 minutes, respectively; *P*=.0001). Total procedure time was similar (157 ± 40 minutes vs 148 ± 50 minutes, respectively; *P*=.42). At median of 13 (range 1–34) months' follow-up, 63% in the RMN and 53% in the MCC group were free from VT recurrence (*P*=.206).	3 in-hospital deaths	

Study name or acronym; Author; Year published; PMID	Aim of study; Endpoints; Study type (RCT, observational — multicenter or single center; case series or other [if RCT include intervention and comparator]); Study size (N)	Patient population with inclusion and exclusion criteria	Results (absolute event rates, *P* values; OR or RR; 95% CI)	Other relevant findings or adverse events	Limitations; Other comments; Conclusions
Arya et al. Catheter ablation of scar-related ventricular tachycardia in patients with electrical storm using remote magnetic catheter navigation.<br>• Year published: 2010<br>• PMID: 20723092	Acute success<br>Procedure time<br>Fluoroscopy time<br>Complication<br>Retrospective, single arm<br>N=30	ES	Acute success was achieved in 80% of cases; procedure and fluoroscopy times of 158 ± 47 minutes and 9.8 ± 5.3 minutes; during follow-up of 7.8 months, 21 (70%) patients had no recurrence of VT.	No complications	
Haghjoo et al. Initial clinical experience with the new irrigated tip magnetic catheter for ablation of scar-related sustained ventricular tachycardia: a small case series.<br>• Year published: 2009<br>• PMID: 19175449	Acute success<br>Procedure time<br>Fluoroscopy time<br>Retrospective, single arm<br>N=5	ES, scar VT	Acute success was achieved in 3 patients; median total procedure and fluoroscopy times of 135 (100–150) minutes and 6.5 (5–9) minutes, respectively.		

Study name or acronym; Author; Year published; PMID	Aim of study; Endpoints; Study type (RCT, observational — multicenter or single center; case series or other [if RCT include intervention and comparator]); Study size (N)	Patient population with inclusion and exclusion criteria	Results (absolute event rates, *P* values; OR or RR; 95% CI)	Other relevant findings or adverse events	Limitations; Other comments; Conclusions
Di Biase et al. Endo-epicardial ablation of ventricular arrhythmias in the left ventricle with the Remote Magnetic Navigation System and the 3.5-mm open irrigated magnetic catheter: results from a large single-center case-control series.<br>• Year published: 2010<br>• PMID: 20434589	Procedural time<br>RF time<br>Fluoroscopy time<br>Acute success<br>Long-term success	Left VA<br>30% ICM<br>13% NICM<br>57% idiopathic<br>33% epicardial	RMN was associated with a longer procedural time (2.9 ± 1.2 hours vs 3.3 ± 1.1 hours, *P*=.004) and RF time (24 ± 12 minutes vs 33 ± 18 minutes, *P*=.005). Fluoroscopic time was shorter (35 ± 22 minutes vs 26 ± 14 minutes, *P*=.033). At 11.7 ± 2.1 months of follow-up in the study group and 18.7 ± 3.7 months in the manual ablation group, 85% and 86% (*P*=.817) of patients, respectively, were free of VA.		During the procedures, crossover to manual ablation was required in 15 (14%) patients.
Aryana et al. Remote magnetic navigation to guide endocardial and epicardial catheter mapping of scar-related ventricular tachycardia.<br>• Year published: 2007<br>• PMID: 17296855	VT termination<br>Fluoroscopy time<br>Retrospective, single arm<br>N=27	VT (all)<br>24 LV<br>10 RV<br>12 epicardial	There was 81% termination, with 4 manual crossovers (for irrigated tip RF). The mean fluoroscopy times for endocardial and epicardial mapping were 27 ± 23 seconds (range, 0–105 seconds) and 18 ± 18 seconds (range, 0–49 seconds), respectively.		RMN RF catheter without irrigation

Study name or acronym; Author; Year published; PMID	Aim of study; Endpoints; Study type (RCT, observational — multicenter or single center; case series or other [if RCT include intervention and comparator]); Study size (N)	Patient population with inclusion and exclusion criteria	Results (absolute event rates, *P* values; OR or RR; 95% CI)	Other relevant findings or adverse events	Limitations; Other comments; Conclusions
Qian et al. Early and long-term outcomes after manual and remote magnetic navigation-guided catheter ablation for ventricular tachycardia.<br>• Year published: 2018<br>• PMID: 29722861	Procedure success<br>Complications<br>Long-term success<br><br>Retrospective comparison<br><br>N=139<br>69 RMN vs 70 MCC<br><br>N=79 for long-term follow-up	VT	RMN had higher acute procedural success than MCC (80% vs 60%, respectively; *P*=.01). In ischemic VT, RMN was associated with longer survival from composite endpoint of VT recurrence, rehospitalization or repeat catheter ablation, and all-cause mortality.		
Hendriks et al. Safety and clinical outcome of catheter ablation of ventricular arrhythmias using contact force sensing: consecutive case series.<br>• Year published: 2015<br>• PMID: 26200478	Acute success<br>Major complications<br>Retrospective comparison<br>N=239<br>RMN vs manual CF vs MCC (no CF)	All VT	Acute success in MCC, manual CF ablation and RMN ablation was 71%, 71%, and 86%, respectively (*P*=.03).	Major complications occurred in 3.3%, and there were fewer major complications (*P*=.04) in the RMN group.	

Study name or acronym; Author; Year published; PMID	Aim of study; Endpoints; Study type (RCT, observational — multicenter or single center; case series or other [if RCT include intervention and comparator]); Study size (N)	Patient population with inclusion and exclusion criteria	Results (absolute event rates, *P* values; OR or RR; 95% CI)	Other relevant findings or adverse events	Limitations; Other comments; Conclusions
Szili-Torok et al. Catheter ablation of ventricular tachycardias using remote magnetic navigation: a consecutive case-control study.<br>• Year published: 2012<br>• PMID: 22554147	Acute success<br>Complications<br>Fluoroscopy time<br>Ablation time<br>Retrospective comparison<br>N=113<br>72 RMN vs 41 MCC	SHD and idiopathic VA<br>43 SHD<br>70 idiopathic	Acute success RMN (82%) vs 27 (66%) MCC (*P*=.046).<br>Overall procedural time (177 ± 79 vs 232 ± 99 minutes; *P*<.01) and mean patient fluoroscopy time (27 ± 19 vs 56 ± 32 minutes; *P*<.001) were all significantly lower using RMN.<br>In idiopathic VT, higher acute success was achieved with RMN (83.7% vs 61.9%; *P*=.049), with shorter procedure times (151 ± 57 vs 210 ± 96, *P*=.011), whereas in SHD-VT these were not significantly different. At follow-up (20 ± 11 vs 20 ± 10 months), VT had recurred in 14 (23.7%) patients with RMN vs 12 (44.4%) patients with MCC (*P*=.047).	No major complications in the RMN group (0%) vs 2 in the MCC group (4.9%, NS).	
Turagam et al. Hemodynamic support in ventricular tachycardia ablation: an international VT ablation center collaborative group study.<br>• Year published: 2017<br>• PMID: 29759835	Long-term VT recurrence<br>Acute success<br>Fluoroscopy<br>Complications<br>Meta-analysis of 7 studies: 1 RCT; 3 prospective; 3 retrospective.<br>N=779<br>433 RMN vs 339 MCC	SHD and idiopathic VA	VT recurrence was lower with RMN (OR 0.61; 95% CI 0.44–0.85; *P*=.003) compared with MCC. RMN had higher acute procedural success (OR 2.13; 95% CI 1.40–3.23; *P*=.0004).<br>Fluoroscopy (mean difference -10.42; 95% CI -12.7 to -8.1; *P*<.0001), procedural time (mean difference -9.79; 95% CI -19.27 to -0.3; *P*=.04) and complications (OR 0.35, 95% CI 0.17 to 0.74; *P*=.0006) were also significantly lower in RMN compared with MCC.		In a subgroup analysis of SHD, there was no significant difference in VT recurrence or acute procedural success with RMN vs MCC. In idiopathic VT, RMN significantly increased acute procedural success, with no difference in VT recurrence.

Study name or acronym; Author; Year published; PMID	Aim of study; Endpoints; Study type (RCT, observational — multicenter or single center; case series or other [if RCT include intervention and comparator]); Study size (N)	Patient population with inclusion and exclusion criteria	Results (absolute event rates, *P* values; OR or RR; 95% CI)	Other relevant findings or adverse events	Limitations; Other comments; Conclusions
Akca et al. Outcomes of repeat catheter ablation using magnetic navigation or conventional ablation.<br>• Year published: 2013<br>• PMID: 23493412	Comparison of RMN vs MCC in repeat ablation procedures; single center; retrospective observational; n=28 VA cases	Patients undergoing repeat catheter ablation of arrhythmias; of these, 28 were undergoing repeat VT ablation	No difference in acute success or recurrence between RMN and MCC (87.5% vs 66.7%, *P*=.230 and 16.7% vs 40.0%, *P*=.330, respectively). No difference in the median number of RF applications (13.0 vs 9.0; *P*=.392), total RF time (481 [IQR 214–990] vs 270 [IQR 51–1200) seconds; *P*=.495), or procedure time (181 ± 100 vs 190 ± 62 minutes; *P*=.891). RMN was associated with decreased fluoroscopy time (22.8 ± 14.7 vs 41.2 ± 10.8 minutes; *P*=.011).		No crossovers were reported from MCC to RMN.
Bauerinfeind et al. The magnetic navigation system allows safety and high efficacy for ablation of arrhythmias.<br>• Year published: 2011<br>• PMID: 21508006	To compare the safety and long-term efficacy of the MNS with MCC; single center; observational registry; n=610 total, of which 83 patients underwent VT ablation (54 MNS and 29 MCC); the numbers of SHD VT ablations was similar between groups, but MNS was used more often than MCC for idiopathic VA ablations (37 vs 14).	Patients undergoing ablation in a single center from January 2008 to March 2010.	Acute success was similar between MNS and MCC for VT in SHD (VT-SHD 14 of 17 [82%] vs 10 of 15 [67%]; *P*=.306) but was higher with MNS in idiopathic VA cases (36 of 37 [97%] vs 11 of 14 [79%]; *P*=.026). This was the only subgroup of the entire population in which there was a significant difference between groups. Fluoroscopy time and procedure time were shorter with MNS (27 ± 21 vs 56 ± 31 minutes, *P*=.001; and 166 ± 54 vs 222 ± 97 minutes, *P*=.009, respectively). There was no difference in recurrence in the VA group (14% vs 14%, *P*=NS).	In two patients (both in the VT group, both had abdominal ICD implants), magnet mode (asynchronous pacing) was induced, without long-term device damage. In the overall population, there were fewer major complications in the MNS group, driven by pericardial effusion or tamponade in the MCC group.	No crossovers from MNS to MCC occurred in the VT group.

Study name or acronym; Author; Year published; PMID	Aim of study; Endpoints; Study type (RCT, observational — multicenter or single center; case series or other [if RCT include intervention and comparator]); Study size (N)	Patient population with inclusion and exclusion criteria	Results (absolute event rates, *P* values; OR or RR; 95% CI)	Other relevant findings or adverse events	Limitations; Other comments; Conclusions
**Electroanatomic Mapping Systems**					
Sporton et al. Electroanatomic vs fluoroscopic mapping for catheter ablation procedures: a prospective randomized study.<br>• Year published: 2004<br>• PMID: 15030422	Procedural success<br>Duration<br>Fluoroscopy time<br>Prospective randomized<br>N=102<br>CARTO vs conventional	Referred for catheter ablation	Acute procedural success was similar with either strategy (CARTO vs conventional 43 of 47 vs 51 of 55, respectively; *P*>.5), as was procedure duration (mean [SD] 144 [58] vs 125 [48] minutes, respectively; *P*=.07). CARTO was associated with a substantial reduction in fluoroscopy time (9.3 [7.6] vs 28.8 [19.5] minutes; *P*<.001) and radiation dose (6.2 [6.1] vs 20.8 [32.7] Gray; *P*=.003).		No VT
Khaykin et al. Real-time integration of 2D intracardiac echocardiography and 3D electroanatomical mapping to guide ventricular tachycardia ablation.<br>• Year published: 2008<br>• PMID: 18929326	To evaluate a mapping system integrating ICE with 3D mapping (Cartosound) to guide VT ablation, including annotation of scar based on ICE appearances; single center; observational; N=17	From February 2007 to April 2008, 17 patients with nonidiopathic VT refractory to amiodarone undergoing VT ablation.	Cartosound maps took 26 ± 8 minutes to create. Anatomical structures such as PAPs, aneurysms, and trabeculations were successfully traced. A mean of 23 ± 7 contours were used to create LV maps. Regional wall motion abnormalities were identified and tagged in all patients. These corresponded to areas of low bipolar voltage (<0.5 mV) during substrate mapping in each case. The average scar area defined by ICE was 33 ± 32 cm^2 compared with 36 ± 33 cm^2 for point-by-point substrate mapping using a 3.5-mm catheter (*P*=.4).		Expertise in ICE imaging is required for this system; electrogram analysis must be performed by conventional point-by-point or multielectrode mapping.

Study name or acronym; Author; Year published; PMID	Aim of study; Endpoints; Study type (RCT, observational — multicenter or single center; case series or other [if RCT include intervention and comparator]); Study size (N)	Patient population with inclusion and exclusion criteria	Results (absolute event rates, *P* values; OR or RR; 95% CI)	Other relevant findings or adverse events	Limitations; Other comments; Conclusions
Earley et al. Radiofrequency ablation of arrhythmias guided by non-fluoroscopic catheter location: a prospective randomized trial.<br>• Year published: 2006<br>• PMID: 16613932	Procedural success<br>Complications<br>Long term success<br>Fluoroscopy time<br>Cost<br>Prospective, randomized<br>N=145<br>CARTO vs NavX vs conventional	Referred for catheter ablation	Overall procedure time, immediate and short-term success, complication rate, and freedom from symptoms at follow-up were identical for all groups. NavX led to the least X-ray exposure: Navx vs conventional, median (range) 4 (0–50) vs 13 (2–46) minutes (*P*<.001); NavX vs Carto, median (range) 4 (0–50) vs 6 (1–55) minutes (*P*=.008). Both Carto and NavX increased disposable costs by 50% when compared with conventional (*P*<.001).		No VT
Khongphatthanayothin et al. Nonfluoroscopic three-dimensional mapping for arrhythmia ablation: tool or toy?<br>• Year published: 2000<br>• PMID: 10749346	Fluoroscopy time<br>Retrospective comparison<br>N=188<br>88 EAM vs 100 conventional	AV nodal reentrant tachycardia<br>AT/atrial flutter<br>VT<br>ORT	Fluoroscopy time was shorter using the CARTO technique: 15 ± 12 vs 34 ± 31 minutes for VT; *P*<.05).		Small VT cohort
Marchlinski et al. Linear ablation lesions for control of unmappable ventricular tachycardia in patients with ischemic and nonischemic cardiomyopathy.<br>• Year published: 2000<br>• PMID: 10725289	Retrospective<br>Single arm<br>N=16<br>EAM used in 13	Unmappable VT	The amount of endocardium demonstrating an abnormal electrogram amplitude ranged from 25 to 127 cm^2. A total of 8 to 87 RF lesions (mean, 55) produced a median of 4 linear lesions that had an average length of 3.9 cm (range, 1.4–9.4 cm). Twelve (75%) patients have been free of VT during 3 to 36 months of follow-up (median, 8 months);<br>use of 1.5 mV cutoff for scar.		

Study name or acronym; Author; Year published; PMID	Aim of study; Endpoints; Study type (RCT, observational — multicenter or single center; case series or other [if RCT include intervention and comparator]); Study size (N)	Patient population with inclusion and exclusion criteria	Results (absolute event rates, *P* values; OR or RR; 95% CI)	Other relevant findings or adverse events	Limitations; Other comments; Conclusions
Soejima et al. Catheter ablation in patients with multiple and unstable ventricular tachycardias after myocardial infarction: short ablation lines guided by reentry circuit isthmuses and sinus rhythm mapping.<br>• Year published: 2001<br>• PMID: 11489772	Retrospective<br>Single arm<br>N=40	Ischemic VT	An isthmus was identified in 25 patients (63%; 5 with only stable VTs, 5 with only unstable VTs, and 15 with both VTs). Inducible VTs were abolished or modified in 100% of patients when the RF line included an isthmus compared with 53% when RF had to be guided by pace mapping (*P*=.0002); those with an isthmus identified received shorter ablation lines (4.9 ± 2.4 vs 7.4 ± 4.3 cm total length; *P*=.02). During follow-up, spontaneous VT decreased markedly regardless of whether an isthmus was identified.		
Ouyang et al. Electroanatomic substrate of idiopathic left ventricular tachycardia: unidirectional block and macroreentry within the Purkinje network.<br>• Year published: 2002<br>• PMID: 11815429	Retrospective<br>Single arm<br>N=9<br>6 additional control patients	Idiopathic LV VT	RetroPP was found within the posterior Purkinje fiber network only in patients with ILVT. No ILVT was inducible during control stimulation, and none recurred during follow-up of 9.1 ± 5.1 months.		

Study name or acronym; Author; Year published; PMID	Aim of study; Endpoints; Study type (RCT, observational — multicenter or single center; case series or other [if RCT include intervention and comparator]); Study size (N)	Patient population with inclusion and exclusion criteria	Results (absolute event rates, *P* values; OR or RR; 95% CI)	Other relevant findings or adverse events	Limitations; Other comments; Conclusions
Soejima et al. Electrically unexcitable scar mapping based on pacing threshold for identification of the reentry circuit isthmus: feasibility for guiding ventricular tachycardia ablation.<br>• Year published: 2002<br>• PMID: 12270862	Retrospective<br>Single arm<br>N=14	Ischemic VT	EUS was identified in the infarct in all 14 patients (11.8 ± 13.9 cm^2). All 20 VT circuit isthmuses identified were adjacent to EUS. Many isthmuses had very low-amplitude electrograms, and EUS could not be identified from electrogram amplitude alone. RF ablation lines connecting selected EUS regions abolished all inducible VTs in 10 (71%) patients; spontaneous VT was markedly reduced during follow-up (from 142 ± 360 to 0.9 ± 2.0 episodes per month; *P*=.002).		
Reddy et al. Short-term results of substrate mapping and radiofrequency ablation of ischemic ventricular tachycardia using a saline-irrigated catheter.<br>• Year published: 2003<br>• PMID: 12821253	Prospective<br>Single arm<br>(Mapping method: SR)<br>N=11	Ischemic VT	With SR ablation strategy, target VT was eliminated in 9 of 11 patients (82%). Furthermore, when targeting all inducible monomorphic VTs, complete procedural success was achieved in 7 of 11 patients (64%).		RF ablation lesions were placed in a linear fashion traversing the border zones of infarcted and normal tissue (mean of 3.4 linear lesions per patient).

Study name or acronym; Author; Year published; PMID	Aim of study; Endpoints; Study type (RCT, observational — multicenter or single center; case series or other [if RCT include intervention and comparator]); Study size (N)	Patient population with inclusion and exclusion criteria	Results (absolute event rates, *P* values; OR or RR; 95% CI)	Other relevant findings or adverse events	Limitations; Other comments; Conclusions
Reithmann et al. Electroanatomic mapping of endocardial right ventricular activation as a guide for catheter ablation in patients with arrhythmogenic right ventricular dysplasia.<br>• Year published: 2003<br>• PMID: 12822746	Retrospective<br>Single arm<br>N=5	ARVC	Electroanatomic mapping during ventricular tachycardia facilitates localization of exit sites in relation to aneurysms in diseased right ventricle and can guide catheter ablation in patients with arrhythmogenic RV dysplasia.		
Arenal et al. Ablation of electrograms with an isolated, delayed component as treatment of unmappable monomorphic ventricular tachycardias in patients with structural heart disease.<br>• Year published: 2003<br>• PMID: 12570949	Retrospective<br>Single Arm<br>N=24: 21 ICM, 2 NICM, 1 ToF	Referred for catheter ablation	Endocardial EAMs (CARTO System) during SR and RVA pacing were obtained to define areas for which an electrogram displayed isolated, delayed components (E-IDC). An E-IDC area related to the clinical VT was identified in each patient. Ablation guided by E-IDC suppressed all but one clinical VT whose inducibility suppression was tested.<br>During a follow-up period of 9 ± 4 months, 3 patients had recurrences of the ablated VT and 2 of a different VT.		

Study name or acronym; Author; Year published; PMID	Aim of study; Endpoints; Study type (RCT, observational — multicenter or single center; case series or other [if RCT include intervention and comparator]); Study size (N)	Patient population with inclusion and exclusion criteria	Results (absolute event rates, *P* values; OR or RR; 95% CI)	Other relevant findings or adverse events	Limitations; Other comments; Conclusions
Soejima et al. Endocardial and epicardial radiofrequency ablation of ventricular tachycardia associated with dilated cardiomyopathy: the importance of low-voltage scars.<br>• Year published: 2004<br>• PMID: 15145109	Retrospective<br>Single arm<br>N=28<br>Voltage mapping<br>26 endocardial<br>8 epicardial	NICM	Ventricular tachycardia was due to focal VT in 5, BBR in 2, and myocardial reentry in 22 patients. All patients with myocardial reentry had endocardial (20 of 20 patients) and/or epicardial (7 of 7 patients mapped) scar. Most (63%) endocardial scars were adjacent to a valve annulus. Of the 19 VT circuit isthmuses identified, 12 were associated with an endocardial scar and 7 with an epicardial scar. All myocardial reentrant VTs were abolished in 12 of 22 patients, and inducible VT was modified in 4 patients. During follow-up of 334 ± 280 days, 54% of patients with myocardial reentry were free of VT despite frequent episodes before ablation.		The VTs in DCM are most commonly the result of myocardial reentry associated with scar. Scars are often adjacent to a valve annulus, deep in the endocardium, and can be greater in extent on the epicardium than on the endocardium. The use of epicardial mapping and RF is likely to improve success.
Marchlinski et al. Electroanatomic substrate and outcome of catheter ablative therapy for ventricular tachycardia in setting of right ventricular cardiomyopathy.<br>• Year published: 2004<br>• PMID: 15477406	Retrospective<br>Single Arm<br>N=21<br>Voltage mapping	RV cardiomyopathy	Electrogram abnormalities extended from perivalvular tricuspid valves (5 patients), pulmonic valves (6 patients), or both valves (10 patients). Electrogram abnormalities always involved free wall, spared the apex, and included the septum in 15 patients (71%). The area of abnormality was 55 ± 37 cm^2 (range, 12–130 cm^2) and represented 34% ± 19% of the RV. In 52 of 66 LBBB VTs, the origin was from the RV perivalvular region. LV perivalvular low-voltage areas noted in 5 patients were associated with an RBBB VT origin. No VT recurred after ablation in 17 (89%) patients during 27 ± 22 months.		

Study name or acronym; Author; Year published; PMID	Aim of study; Endpoints; Study type (RCT, observational — multicenter or single center; case series or other [if RCT include intervention and comparator]); Study size (N)	Patient population with inclusion and exclusion criteria	Results (absolute event rates, *P* values; OR or RR; 95% CI)	Other relevant findings or adverse events	Limitations; Other comments; Conclusions
Zeppenfeld et al. Catheter ablation of ventricular tachycardia after repair of congenital heart disease: electroanatomic identification of the critical right ventricular isthmus.<br>• Year published: 2007<br>• PMID: 17967973	Retrospective<br>Single arm<br>N=11	Repaired congenital HD	The reentry circuit isthmuses of all 15 induced VTs, identified by activation, entrainment, and/or pace mapping, were located in an anatomic isthmus (11 of 15 VTs in anatomic isthmus 1). Transecting the anatomic isthmuses by ablation lesions abolished all VTs. During 30.4 ± 29.3 months of follow-up, 91% of patients remained free of VT.		
Stevenson et al. Irrigated radiofrequency catheter ablation guided by electroanatomic mapping for recurrent ventricular tachycardia after myocardial infarction: the multicenter thermocool ventricular tachycardia ablation trial.<br>• Year published: 2008<br>• PMID: 19064682	VT freedom<br>Observational, multicenter<br>N=231	Ischemic MMVT	Ablation abolished all inducible VTs in 49% of patients. The primary endpoint of freedom from recurrent incessant VT or intermittent VT after 6 months of follow-up was achieved for 123 patients (53%). In 142 patients with ICD before and after ablation for intermittent VT who survived 6 months, VT episodes were reduced from a median of 11.5 to 0 (*P*<.0001). The 1-year mortality rate was 18%, with 72.5% of deaths attributed to VAs or heart failure.<br>The procedure mortality rate was 3%, with no strokes.		

Study name or acronym; Author; Year published; PMID	Aim of study; Endpoints; Study type (RCT, observational — multicenter or single center; case series or other [if RCT include intervention and comparator]); Study size (N)	Patient population with inclusion and exclusion criteria	Results (absolute event rates, *P* values; OR or RR; 95% CI)	Other relevant findings or adverse events	Limitations; Other comments; Conclusions
Codreanu et al. Electroanatomic characterization of post-infarct scars comparison with 3-dimensional myocardial scar reconstruction based on magnetic resonance imaging.<br>• Year published: 2008<br>• PMID: 18755347	Scar size<br>EGM quality<br>Retrospective (Imaging)<br>N=10	Infarct scar<br>EAM vs MRI	Endocardial scars had a larger degree of signal reduction than intramural or epicardial scars. A clear mismatch in infarct surface between CARTO and MRI maps was observed in one-third of infarct zones.	None of the parameters was correlated with transmural scar depth.	

Postprocedural Care

Study name or acronym; Author; Year published; PMID	Aim of study; Endpoints; Study type (RCT, observational — multicenter or single center; case series or other [if RCT include intervention and comparator]); Study size (N)	Patient population with inclusion and exclusion criteria	Results (absolute event rates, *P* values; OR or RR; 95% CI)	Other relevant findings or adverse events	Limitations; Other comments; Conclusions
**Arterial Access**					
Schulz-Schüpke et al. Comparison of vascular closure devices vs manual compression after femoral artery puncture: the ISAR-CLOSURE randomized clinical trial.<br>• Year published: 2014<br>• PMID: 25399273	Aim: To compare outcomes with the use of 2 hemostasis strategies after diagnostic coronary angiography performed via transfemoral access: a vascular closure device-based strategy with 2 types of devices, an intravascular device and an extravascular device, vs standard MC<br>Endpoints: Primary endpoint: vascular access site complications, ie, the composite of a hematoma at least 5 cm in size, pseudoaneurysm, arteriovenous fistula, access site-related major bleeding, acute ipsilateral leg ischemia, need for vascular surgical or interventional treatment, or local	Inclusion criteria: Undergoing diagnostic coronary angiography (without subsequent percutaneous coronary intervention) with a 6 Fr sheath through the common femoral artery, which had to have a diameter of greater than 5 mm (proven by angiography).<br><br>Exclusion criteria: Implantation of a VCD within the last 30 days, symptomatic leg ischemia, prior TEA or patch plastic of the common femoral artery, planned invasive diagnostic or interventional procedure in the following 90 days, a heavily calcified	The primary endpoint was observed in 208 (6.9%) patients assigned to receive a VCD and 119 (7.9%) patients assigned to MC (difference, −1.0% [1-sided 97.5% CI, 0.7%]; P for noninferiority <.001). Time to hemostasis was significantly shorter in patients with VCD (1 [IQR 0.5–2.0] minute) vs manual compression (10 [IQR 10–15] minutes; *P*<.001). Time to hemostasis was significantly shorter among patients with intravascular VCD (0.5 [IQR 0.2–1.0] minute), vs extravascular VCD (2.0 [IQR 1.0–2.0] minutes; *P*<.001) and closure device failure was also significantly lower among those with intravascular vs extravascular VCD (80 [5.3%] vs 184 [12.2%] patients; *P*<.001).	In patients undergoing transfemoral coronary angiography, VCDs were noninferior to manual compression in terms of vascular access-site complications and reduced time to hemostasis.	Randomly assigned to arteriotomy closure with 1 of the following techniques: the intravascular FemoSeal VCD, the extravascular Exoseal VCD, or MC at a 1:1:1 ratio after performance of coronary angiography and femoral angiography of the access site via the 6 Fr sheath. Time to hemostasis (0.5 [0.2–1.0] vs 2.0 [1.0–2.0] minutes; *P*<.001) and closure device failure (80 [5.3%] vs 184 patients [12.2%]; *P*<.001) were lower with the intravascular VCD group, compared with the extravascular VCD group.

Study name or acronym; Author; Year published; PMID	Aim of study; Endpoints; Study type (RCT, observational — multicenter or single center; case series or other [if RCT include intervention and comparator]); Study size (N)	Patient population with inclusion and exclusion criteria	Results (absolute event rates, *P* values; OR or RR; 95% CI)	Other relevant findings or adverse events	Limitations; Other comments; Conclusions
	infection at 30 days after randomization.<br>Secondary endpoints: time to hemostasis, repeat MC, and VCD failure. An α-level of .025 was chosen for primary and secondary comparisons.<br>Study type: Randomized, large-scale, multicenter, open-label clinical trial<br>Size: 4524 enrolled, 3015 randomized to hemostasis with an intravascular VCD, extravascular VCD, or MC in a 1:1:1 ratio.	vessel, active bleeding or bleeding diathesis, severe arterial hypertension (>220/110 mm Hg), local infection, autoimmune disease, allergy to resorbable suture, and pregnancy.			

Study name or acronym; Author; Year published; PMID	Aim of study; Endpoints; Study type (RCT, observational — multicenter or single center; case series or other [if RCT include intervention and comparator]); Study size (N)	Patient population with inclusion and exclusion criteria	Results (absolute event rates, *P* values; OR or RR; 95% CI)	Other relevant findings or adverse events	Limitations; Other comments; Conclusions
Holm et al. Randomised comparison of manual compression and FemoSeal™ vascular closure device for closure after femoral artery access coronary angiography: the CLOSure dEvices Used in everyday Practice (CLOSE-UP) study.<br>• Year published: 2014<br>• PMID: 24603054	Aim: To compare in a randomized trial the safety and efficacy of the FemoSeal VCD vs MC after femoral access CAG<br>Primary endpoint: Incidence of large groin hematoma<br>Study type: Randomized<br>Size: 1014; 1001 analyzed		Median (IQR) closure time was 8.0 (6–10) minutes after MC versus 1.0 (1–1) minute (*P*<.0001) for the FemoSeal VCD. The primary endpoint of incidence of large groin hematoma was 6.7% in the MC group vs 2.2% (*P*=.002) in the FemoSeal group. The combined endpoint of 14-day adverse vascular events occurred in 1.0% in the MC group vs 0.6% in the FemoSeal VCD group (*P*=.7).	MC (OR 3.3; 95% CI 1.5–7.2; *P*=.002), female sex (OR 2.1; 95% CI 1.1–3.9; *P*=.018), and multiple punctures (OR 10.5; 95% CI 3.2–34.3; *P*=.001) were identified as independent predictors of adverse events and large hematomas.	Bed rest for 1 hour after the closure procedure was recommended in both groups. Closure of femoral access after coronary angiography by the FemoSeal VCD was safe, faster, and associated with significantly fewer in-hospital large hematomas compared with closure by manual compression.
Robertson et al. Vascular closure devices for femoral arterial puncture site haemostasis.<br>• Year published: 2016<br>• PMID: 26948236	Aim: To determine efficacy and safety of VCDs vs traditional methods of extrinsic compression in achieving hemostasis after retrograde and antegrade percutaneous arterial puncture of the CFA.<br>Endpoints: Efficacy (time to hemostasis, time to	Randomized and quasi-randomized controlled trials in which people undergoing a diagnostic or interventional procedure via percutaneous CFA puncture were randomized to one type of VCD versus	Time to hemostasis: Both metal clip-based (MD -14.81 mins; 95% CI -16.98 to -12.63 mins; 5 studies, 1665 participants) and suture-based VCDs (MD -14.58 mins, 95% CI -16.85 to -12.32 mins; 7 studies; 1664 participants) were associated with reduced time to hemostasis compared with extrinsic compression.<br>Time to mobilization: studies comparing collagen-, metal clip- and suture-based devices with extrinsic compression were too heterogeneous to be combined.		For time to hemostasis, studies comparing collagen-based VCDs and extrinsic compression were too heterogeneous to be combined. However, both metal clip-based and suture-based VCDs were associated with

Study name or acronym; Author; Year published; PMID	Aim of study; Endpoints; Study type (RCT, observational — multicenter or single center; case series or other [if RCT include intervention and comparator]); Study size (N)	Patient population with inclusion and exclusion criteria	Results (absolute event rates, *P* values; OR or RR; 95% CI)	Other relevant findings or adverse events	Limitations; Other comments; Conclusions
	mobilization), safety (major adverse event, such as mortality and vascular injury requiring repair), adverse events<br>Study type: Systematic review<br>Size: 52 studies (19,192 participants) in the review.	extrinsic compression or another type of VCD. Studies comparing VCDs and extrinsic compression (sheath size ≤9 Fr) were found.	Deaths: None reported in studies comparing collagen-based, metal clip-based or suture-based VCDs with extrinsic compression.<br>Vascular injury requiring repair: neither collagen (OR 2.81; 95% CI 0.47–16.79; 6 studies; 5731 participants) nor metal clip-based VCDs (OR 0.49; 95% CI 0.03–7.95; 3 studies; 783 participants) were more effective than extrinsic compression. No cases of vascular injury required repair in the study testing suture-based VCD with extrinsic compression.<br>Infection: No differences in the incidence between collagen-based (OR 2.14; 95% CI 0.88–5.22; 9 studies; 7616 participants) or suture-based VCDs (OR 1.66, 95% CI 0.22–12.71; 3 studies; 750 participants) and extrinsic compression. No cases of infection were observed in studies testing suture-based VCD versus extrinsic compression.<br>Groin hematoma: Lower with collagen-based VCDs than with extrinsic compression (OR 0.46; 95% CI 0.40–0.54; 25 studies; 10,247 participants), but no difference was evident when metal clip-based (OR 0.79; 95% CI 0.46–1.34; 4 studies; 1523 participants) or suture-based VCDs (OR 0.65; 95% CI 0.41–1.02; six studies; 1350 participants) were compared with extrinsic compression.		reduced time to hemostasis compared with extrinsic compression. For time to mobilization, studies comparing VCDs with extrinsic compression were too heterogeneous to be combined. No difference was demonstrated in the incidence of vascular injury or mortality when VCDs were compared with extrinsic compression. No difference was demonstrated in the efficacy or safety of VCDs with different mechanisms of action. Further work is necessary to evaluate the efficacy of devices currently in use and to compare these with one other and extrinsic

Study name or acronym; Author; Year published; PMID	Aim of study; Endpoints; Study type (RCT, observational — multicenter or single center; case series or other [if RCT include intervention and comparator]); Study size (N)	Patient population with inclusion and exclusion criteria	Results (absolute event rates, *P* values; OR or RR; 95% CI)	Other relevant findings or adverse events	Limitations; Other comments; Conclusions
			Pseudoaneurysm: Lower with collagen-based devices than with extrinsic compression (OR 0.74; 95% CI 0.55–0.99; 21 studies; 9342 participants), but no difference was noted when metal clip-based (OR 0.76; 95% CI 0.20–2.89; 6 studies; 1966 participants) or suture-based VCDs (OR 0.79; 95% CI 0.25–2.53; 6 studies; 1527 participants) were compared with extrinsic compression.<br>Other adverse events: No differences between collagen-based, clip-based or suture-based VCDs and extrinsic compression.<br>VCDs compared with each other: metal clip-based VCDs were associated with shorter time to hemostasis (MD -2.24 minutes; 95% CI -2.54 to -1.94 minutes; 469 participants) and shorter time to mobilization (MD -0.30 hours; 95% CI -0.59 to -0.01 hours; 469 participants) than suture-based devices. Few studies measured (major) adverse events, and those that did found no cases or no differences between VCDs.		compression with respect to clearly defined outcome measures.

Study name or acronym; Author; Year published; PMID	Aim of study; Endpoints; Study type (RCT, observational — multicenter or single center; case series or other [if RCT include intervention and comparator]); Study size (N)	Patient population with inclusion and exclusion criteria	Results (absolute event rates, *P* values; OR or RR; 95% CI)	Other relevant findings or adverse events	Limitations; Other comments; Conclusions
Jiang et al. Network meta-analysis of randomized trials on the safety of vascular closure devices for femoral arterial puncture site haemostasis.<br>• Year published: 2015<br>• PMID: 26349075	Aim: To examine the safety of VCDs<br>Endpoints: Rate of CAVEs and hematomas<br>Study size: 42 RCTs in qualitative synthesis; 40 RCTs in meta-analysis quantitative synthesis (total 16,868 patients)	Patients underwent coronary angiography, angioplasty, peripheral intervention procedures	Direct comparison meta-analysis: Risk for CAVE similar between VCDs and MC: (heterogeneity: Chi2 = 108.07, I2 = 68%; test for overall effect: Z = 1.19, *P*=.23).<br>VCDs after 2005 associated with decreased risk of VCD-associated complications.<br>Hematoma risk lower with VCD than MC.<br>Network meta-analysis: AngioSeal associated with lower risk of CAVE compared with MC; other VCDs have similar risk of CAVE compared with MC. No significant differences between VCDs.		No significant difference in the rate of CAVEs between all the VCDs and MC.
**Epicardial Access**					
Della Bella et al. Epicardial ablation for ventricular tachycardia: a European multicenter study.<br>• Year published: 2011<br>• PMID: 21841191	Aim: To describe the epicardial percutaneous ablation experience of 6 European high-volume VT ablation centers<br>Size: 218	Inclusion criteria: Epicardial VT ablations from centers providing at least 8 cases.	Major procedure-related complications in 4.1%, minor in 7.8%.	Precordial pain could be due to the pigtail left in the pericardium for continuous drainage. Tamponade occurred late in 4 of 8 patients; “provides the rationale for leaving the pericardial sheath in place after the procedure”; important to have a soft additional catheter inserted through the introducer to avoid a myocardial lesion by its sharp edges.	Some 46 (21%) experienced postprocedural precordial pain, considered severe in 1 of 2. Oral steroids used by 1 center routinely after 2007; 1 center routinely used intrapericardial infusion of steroids, 4 centers did not use steroids.

Study name or acronym; Author; Year published; PMID	Aim of study; Endpoints; Study type (RCT, observational — multicenter or single center; case series or other [if RCT include intervention and comparator]); Study size (N)	Patient population with inclusion and exclusion criteria	Results (absolute event rates, *P* values; OR or RR; 95% CI)	Other relevant findings or adverse events	Limitations; Other comments; Conclusions
Dyrda et al. Influence of steroid therapy on the incidence of pericarditis and atrial fibrillation after percutaneous epicardial mapping and ablation for ventricular tachycardia.<br>• Year published: 2014<br>• PMID 24970295	Aim: To evaluate the influence of 3 therapeutic approaches on the incidence of pericarditis and AF after percutaneous epicardial mapping and ablation for VT<br>Study type: Retrospective review; therapeutic approach evolved over time from no steroids to systemic steroids to intrapericardial steroids<br>Size: 85	Inclusion criteria: Consecutive procedures in which epicardial mapping or mapping and RF ablation was performed for VT from 2006–2011<br>Exclusion criteria: Of all the patients, 17 received no steroids; 30 received systemic steroids 1 mg/kg/day intravenously or PO for 3 consecutive days; 38 had complete fluid removal and intrapericardial steroid injection with triamcinolone acetate 2 mg/kg, injected via pigtail, left *in situ* by capping the pigtail. The NSAID diclofenac 50 mg was administered every 8 hours, as needed.	Pericarditis in 15.3%. Pericarditic CP reduced in patients with intrapericardial steroids (21.1%) vs no steroids (58.8%; *P*=.006). Systemic steroids did not significantly reduce pericarditic CP (43.4% vs 58.8% intravenous or PO steroids vs no steroids; *P*=.31). No difference in pericarditis ECG with steroid therapy (36.8%, 30.0%, 41.2% for intrapericardial, intravenous/PO or none, respectively). Nonsignificant reduced incidence of CP and ECG changes with steroids (13.2%, 10.0%, 29.4%, respectively).<br>Pigtail catheter left <3 hours in 8%, 44% had it ≥24%. More CP if pigtail in ≥24 hours (51% vs 25%, *P*=.012), but fewer ECG changes (19% vs 48%, *P*=.006). Postprocedure pericardial effusion or tamponade in 13 patients, observed 18 ± 14 hours after the procedure. In the first echo in 8 of 13, the second in 4 patients (19–20 hours postprocedure), and the third echo in 1 (50 hours but <5 mm).<br>AF: 8.3% AF in patients with no prior history of AF. Median time to new AF 36 hours. Patients with pericarditic ECG tended to be at greater risk of AF (16.7 vs 3.6%; *P*=.091). There were 4 cases of severe pericardial bleeding acutely in 2 and delayed in 2; 1 after epicardial puncture, resolved in 20 minutes, the second due to RV puncture. Delayed cases: 13 hours in a patient accidentally given LMWH; the second at 4.75 hours on heparin and DAPT for recent coronary stent. Both drained.	Median LOS postprocedure 5 days (IQR 3–8 days).	Pericarditic CP was lower with intrapericardial steroids but not with intravenous or oral steroids. AF is frequent and tends to occur more commonly in patients with pericarditic ECG changes.

Study name or acronym; Author; Year published; PMID	Aim of study; Endpoints; Study type (RCT, observational — multicenter or single center; case series or other [if RCT include intervention and comparator]); Study size (N)	Patient population with inclusion and exclusion criteria	Results (absolute event rates, *P* values; OR or RR; 95% CI)	Other relevant findings or adverse events	Limitations; Other comments; Conclusions
Mahapatra et al. Incidence, risk factors, and consequences of new-onset atrial fibrillation following epicardial ablation for ventricular tachycardia.<br>• Year published: 2011<br>• PMID: 21296778	Aim: To determine the incidence, predictors, and consequences of new-onset AF following epicardial VT ablation<br>Endpoints: Documented new-onset AF within 7 days<br>Study type: Retrospective review<br>Size: 41	Inclusion criteria: From a review of 91 epicardial VT ablation procedures from August 2007 to January 2010, 41 patients were included without a history of AF or any atrial arrhythmias by clinical history, prior ECGs, or ICD monitoring.<br>Exclusion criteria: Prior cardiac surgery, requirement for a pericardial window for access, or complicated procedure requiring sternotomy.	AF occurred in 8.3% of patients with no prior history of AF. Median time to new AF was 36 hours. Patients with pericarditic ECGs tended to be at greater risk of AF (16.7 vs 3.6%; *P*=.091). Mahapatra reported a new AF incidence of 19.5%, and all had clinical symptoms of pericarditis. New AF was associated with longer epicardial ablation time (424 ± 169 vs 867 ± 450 seconds; *P*<.001), epicardial ablation >600 seconds (3.0% vs 62.5%; *P*<.001), ablation in epicardium 103 ± 28 vs 135 ± 51 minutes; *P*=.02), RV puncture with ICE bubbles (9.1% vs 50.0%; *P*=.02), and pericarditis score at 24 hours (1.58 ± 0.79 vs 2.25 ± 0.16, *P*=.03). Prolonged drainage >24 hours was not associated with AF (*P*=.28).		All patients received 50 mL of 1% lidocaine without epinephrine intrapericardially. At the operator's discretion, 5 patients received intrapericardial steroids (100 mg triamcinolone), 22 patients received systemic steroids (8 mg dexamethasone), and 8 patients received 30 mg of ketorolac. A 7 Fr Jackson–Pratt drain was left in the pericardium for at least 18–36 h (3M, St Paul, MN). A transthoracic echocardiography was performed routinely the following day to assess for the presence of a pericardial effusion. The drain was removed if a trace or smaller effusion was present, and the pericardial drainage was, 100 cm^3 during the previous 8 h.

Study name or acronym; Author; Year published; PMID	Aim of study; Endpoints; Study type (RCT, observational — multicenter or single center; case series or other [if RCT include intervention and comparator]); Study size (N)	Patient population with inclusion and exclusion criteria	Results (absolute event rates, *P* values; OR or RR; 95% CI)	Other relevant findings or adverse events	Limitations; Other comments; Conclusions
Sugrue et al. Significance and clinical characteristics of atrial fibrillation post epicardial access.<br>• Year published: 2017<br>• PMID: 27943113	Aim: To assess the incidence, clinical impact, and impact of steroids of postprocedural AF in patients undergoing epicardial access and ablation<br>Endpoints: Postprocedural AF occurring during the procedure hospitalization or within 7 days of the procedure (if hospitalization was prolonged >7 days). Only episodes >30 seconds considered<br>Study type: Retrospective review<br>Size: 170	Inclusion criteria: Patients who underwent epicardial access as part of a VT or PVC ablation procedure from 1/2004 to 7/2014 at Mayo Clinic Rochester.	A total of 7 (4.1%) patients developed AF within 7 days of the procedure, mean 49 ± 18 hours postprocedure. All episodes were self-limited; no patient required chemical or electrical cardioversion; 36 (21.1%) had documented AF during the total follow-up period, mean 1.6 ± 2.0 years. Impact of steroids: 60 (35%) patients received intrapericardial steroids; 46 patients received intrapericardial methylprednisolone (dose range 40–125 mg); 14 received intrapericardial dexamethasone (dose range 4–12 mg). One patient received systemic steroids (methylprednisolone 32 mg orally). In those who received intrapericardial steroids (60 patients), 1 (2%) had postprocedural AF documented (received intrapericardial methylprednisolone 80 mg), while 6 (5%) of the remaining 110 who did not receive steroids developed AF (*P*=.42).	None of the AF group received colchicine compared with 21.4% in the non-AF group (*P*=.35).<br>Amiodarone use: Trend toward more use in the group that did not develop AF (29% in postprocedural AF group vs 58%; *P*=.12).<br>No significant difference in epicardial ablation time (370 vs 403 minutes; *P*=.54), pericardial drain use, or the number of days the drain was left in situ postprocedure between groups (0.7 vs 0.6; *P*=.36), rates of pericardial effusion, or cardiac tamponade.	AF was infrequent, tends to be short lived, and was associated with minimal complications. Steroids do not seem to have an impact on recurrence of AF.

Study name or acronym; Author; Year published; PMID	Aim of study; Endpoints; Study type (RCT, observational — multicenter or single center; case series or other [if RCT include intervention and comparator]); Study size (N)	Patient population with inclusion and exclusion criteria	Results (absolute event rates, *P* values; OR or RR; 95% CI)	Other relevant findings or adverse events	Limitations; Other comments; Conclusions
**Venous Access**					
Akkaya et al. Safety and feasibility of percutaneous skin closure using purse-string suture compared with compression bandage after pulmonary vein isolation.<br>• Year published: 2017<br>• PMID: 28608980	Aim: To analyze safety and feasibility of using a PSS vs a CBD after PVI<br>Endpoints: Vascular or thromboembolic complications, hospital cost, hospital stay<br>Study type: Observational<br>Size: 407	Inclusion criteria: RF or cryoablation PVI receiving phenprocoumon with LMWH bridging.<br>Exclusion criteria: Patients on NOACs, continuous phenprocoumon, or no pre-PVI anticoagulation.	RF (N=217) CBD N=125 vs PSS N=92; hematoma with compression therapy 8.8% vs 2.2% (*P*=.05); any groin complication in a patient 12.0% vs 3.3% (*P*<.05); any groin complication/# puncture sites 6.0% vs 1.6% (*P*<.05); patient hospital stay, PVI 2.98 ± 1.57 vs 2.34 ± 1.32 days (*P*<.05); hospital costs per patient 5,802 ± 4,006 vs 4,921 ± 3,145 Euro (*P*<.05).<br><br>Cryoballoon (N=190) CBD N=94, PSS N=96; any groin complication in a patient 7.45% vs 1.04% (*P*<.05); any groin complication/# puncture sites 7.45% vs 1.04% (*P*<.05); patient hospital stay, PVI 2.61 ± 1.55 vs 2.14 ± 1.37 days (*P*<.05); hospital costs per patient, 5,661 ± 3,563 vs 4,705 ± 3,091 Euro (*P*<.05).<br><br>PSS use was the only predictor of vascular and thromboembolic complications (OR 0.126; 95% CI 0.029–0.553; *P*<.01).		No difference in time to achieve venous closure. Lower vascular complications, hospital cost, hospital stay with PSS. Severe complications (retroperitoneal bleeding and hematomas needing blood cell transfusions and/or surgical repair) were observed only after CBD.

Study name or acronym; Author; Year published; PMID	Aim of study; Endpoints; Study type (RCT, observational — multicenter or single center; case series or other [if RCT include intervention and comparator]); Study size (N)	Patient population with inclusion and exclusion criteria	Results (absolute event rates, *P* values; OR or RR; 95% CI)	Other relevant findings or adverse events	Limitations; Other comments; Conclusions
Aytemir et al. Usefulness of 'figure-of-eight' suture to achieve haemostasis after removal of 15-French calibre femoral venous sheath in patients undergoing cryoablation.<br>• Year published: 2016<br>• PMID: 26705565	Aim: To assess the efficacy and safety of FoE suture vs MC for immediate closure of 15 Fr venous access after cryoballoon PVI AF ablation<br>Endpoints: Achievement of hemostasis, median time to hemostasis, major vascular access site complications<br>Study type: Sequential allocation<br>Size: 200 (FoE suture, 100; MC, 100)	Inclusion criteria: Undergoing initial PVI using second generation cryoballoon technique<br>Exclusion criteria: Moderate–severe valvular disease, LA thrombus, uncontrolled thyroid dysfunction, preprocedural significant coronary artery stenosis, MI or cardiac surgery in the previous 3 months, contraindication of anticoagulation, pregnancy, and LA diameter of >55 mm.	Acute success was achieved in all patients. FoE suture: hemostasis was achieved immediately after tying the knot (n=95) or within ≤1 minute of light pressure (n=4). One patient had failure of the stitch because the silk suture snapped during knotting, and hemostasis was achieved by MC as per the usual protocol.<br>MC: 4 (4%) patients had hematoma/pseudoaneurysm within 24 hours of sheath removal.<br>Median time to hemostasis: shorter in the suture group (0 vs 14 minutes; *P*<.001). On immediate and short-term (3 months) follow-up no vascular access site complications in the suture group.	Time to mobilization and duration of hospital stay no different (had arterial sheath removal also). No unexpected adverse events or right femoral access complications reported over median follow-up of 3 months.	No ACT measurement.<br>Manual compression: 6 hours bedrest, 12 hours compression dressing.<br>FOE suture: If there was no bleeding, no pressure was applied. If there was slight bleeding, then light pressure was applied for ≤1 minute. Sutures were removed the following morning.<br>The first 40 (20 each group) patients had Doppler within the first 24 hours.<br>Conclusions: FoE suture was simple and faster at achieving hemostasis after removing 15 Fr femoral venous sheaths.

Study name or acronym; Author; Year published; PMID	Aim of study; Endpoints; Study type (RCT, observational — multicenter or single center; case series or other [if RCT include intervention and comparator]); Study size (N)	Patient population with inclusion and exclusion criteria	Results (absolute event rates, *P* values; OR or RR; 95% CI)	Other relevant findings or adverse events	Limitations; Other comments; Conclusions
Lakshmanadoss et al. Figure-of-eight suture for venous hemostasis in fully anticoagulated patients after atrial fibrillation catheter ablation.<br>• Year published: 2017<br>• PMID: 29192589	Aim: To evaluate the FoE closure technique in achieving vascular hemostasis after AF catheter ablation compared to manual pressure<br>Endpoints: Time to ambulation postprocedure<br>Secondary: vascular complications including groin bleeding, hematoma (size >3 cm), retroperitoneal bleeding, pseudoaneurysm, or arteriovenous fistula. Major vascular complication (need for blood transfusion, vascular intervention, or vascular surgical intervention as a result of access site complication)<br>Study type: Nonrandomized retrospective case control cohort; manual pressure historical control group 1/2012-8/2013; case study group using FoE suture 9/2013-8/2014.<br>Size: 284 (manual compression 105; FoE 179)	Inclusion criteria: All patients 18 years or older who underwent AF catheter ablation by two cardiac electrophysiologists from January 2012 to August 2014.<br>Exclusion criteria: Presence of LA thrombus, INR >3.5 on the day of procedure, and severe uncontrolled HF or any contraindications to general anesthesia.	No difference in holding time on table. Longer time on bedrest with manual compression compared with FoE suture (573 ± 80 vs 373 ± 49 minutes, *P*<.0001). More major hematoma in manual compression vs FoE suture (10.5% vs 3.9%, *P*=.041). No difference in groin access complications.		No US guidance use, which could have increased vascular complications (AV fistula, pseudoaneurysm). Temporal changes in practice could have confounded results. FoE associated with shorter time on bedrest.

Study name or acronym; Author; Year published; PMID	Aim of study; Endpoints; Study type (RCT, observational — multicenter or single center; case series or other [if RCT include intervention and comparator]); Study size (N)	Patient population with inclusion and exclusion criteria	Results (absolute event rates, *P* values; OR or RR; 95% CI)	Other relevant findings or adverse events	Limitations; Other comments; Conclusions
Okada et al. Efficacy and safety of figure-of-eight suture for hemostasis after radiofrequency catheter ablation for atrial fibrillation.<br>• Year published: 2018<br>• PMID: 29375108	Aim: To evaluate the safety and efficacy of venous FoE suture to achieve femoral venous hemostasis after RFCA for AF, examining incidence of vascular access site hemostatic failure before and after introduction of the FoE suture technique<br>Endpoints: Rebleeding at the groin requiring additional manual and/or bandage compression after leaving the EP lab. Groin hematoma requiring additional manual and/or bandage compression. Frequency of analgesic and/or antiemetic use during bedrest.<br>Study type: Retrospective case control series<br>Size: N=517 (manual compression with 6-hour bedrest N=247; FoE suture N=270 with 4 hour bedrest).	Patients with AF undergoing RFCA for AF. First 247 consecutive patients had manual compression vs subsequent 270 who had FoE suture technique (changed 5/2016).<br>Inclusion criteria: All patients had symptomatic or asymptomatic AF documented on ECG.<br>Exclusion criteria: Patients with suspected CAD who underwent diagnostic coronary angiography with a 5 Fr femoral artery sheath.	Rebleeding: Lower in FoE vs control (3.7% vs 18.6%, *P*<.001).<br>Hematoma: 0.7% FoE vs 1.2% control (*P*=.67).<br>Analgesic and/or antiemetic: Less in FoE group 19.3% vs 32.0% control (*P*<.001).<br>Major vascular access complications: 2 hematomas in FoE group, 3 hematomas and 1 pseudoaneurysm in controls (*P*=.43).	After discharge, 1 patient had suture abscess due to residual thread, but was cured with its removal.	Limitations: Retrospective, non-randomized, single center.<br>Risk of major bleeding was low.<br>FoE suture was associated with decreased rate of rebleeding, less analgesic and/or antiemetic use; no difference in rate of hematoma. FoE suture technique was an independent negative predictor of rebleeding after CA even after adjustment for the other covariates.

Study name or acronym; Author; Year published; PMID	Aim of study; Endpoints; Study type (RCT, observational — multicenter or single center; case series or other [if RCT include intervention and comparator]); Study size (N)	Patient population with inclusion and exclusion criteria	Results (absolute event rates, *P* values; OR or RR; 95% CI)	Other relevant findings or adverse events	Limitations; Other comments; Conclusions
Jackson et al. Groin haemostasis with a purse string suture for patients following catheter ablation procedures (GITAR Study).<br>• Year published: 2018<br>• PMID: 29685719	Aim: To perform an RCT for femoral venous hemostasis using a PSS technique vs 10 minutes manual pressure, to compare safety, effectiveness, comfort, and determine any other predictors of vascular access site complications.<br>Endpoints: Vascular access site complications. Occurrence of any bruising or bleeding requiring intervention or not. Pain score at the time of PSS/manual pressure.<br>Size: N=200	Patients referred for EP procedures at 2 hospitals randomized to 10 minutes manual pressure or PSS over the femoral vein.	Bleeding requiring additional pressure or a Femostop for complete hemostasis: 17% in PSS vs 19% in manual pressure arm (*P*=.72). No cases of hematoma prolonging hospital stay, arteriovenous fistula, pseudoaneurysm, or retroperitoneal bleeding. Mean duration to achieve hemostasis: 45 seconds PSS arm vs 10 minutes 44 seconds manual pressure arm (*P*<.001). Pain/discomfort associated with hemostasis: 15% PSS arm vs 29% manual pressure (*P*=.03).		No differences in rates of bleeding or vascular complications, complications requiring intervention between PSS vs 10 minutes manual pressure for catheter ablation procedures; no difference in bleeding requiring additional pressure or Femostop. PSS was faster to achieve hemostasis, more comfortable than manual pressure.

**Study name or acronym; Author; Year published; PMID**	**Aim of study; Endpoints; Study type (RCT, observational — multicenter or single center; case series or other [if RCT include intervention and comparator]); Study size (N)**	**Patient population with inclusion and exclusion criteria**	**Results (absolute event rates, *P* values; OR or RR; 95% CI)**	**Other relevant findings or adverse events**	**Limitations; Other comments; Conclusions**
Pracon et al. A randomized comparison of modified subcutaneous "Z"-stitch versus manual compression to achieve hemostasis after large caliber femoral venous sheath removal.<br>• Year published: 2018<br>• PMID: 28303670	Aim: To compare subcutaneous "Z"-stitch versus manual compression in attaining hemostasis after large bore femoral venous access, and to assess its impact on venous patency.<br>Endpoints: time to hemostasis, time to ambulation, and a composite safety endpoint comprising vascular access site complications (access site bleeding, access site hematoma, DVT, AV fistulas, and access site infection).<br>Study type: Single center randomized trial<br>Size: N=86 patients, with 90 access sites	Consecutive patients with femoral venous access sites requiring 10 Fr sheaths to the "Z"-stitch or manual compression for hemostasis in a 2:1 fashion.	Patients randomized to "Z"-stitch achieved hemostasis quicker (<1 vs 12.0 [IQR 10.0–15.0] minutes; *P*<.001) and ambulated sooner (7.0 [IQR 4.0–12.0] vs 16.0 [IQR 11.8–20.3] hours postprocedure; P<.001) compared with manual compression. "Z"-stitch reduced rates of access site complications (OR 0.27; 95% CI 0.09–0.76; *P*=.01). All imaged veins were patent before and after stitch removal.		Limitations: Relatively small sample size, prior to Z stitch, 10-hour bed rest was standard of care. "Z"-stitch is a safe and effective method of achieving hemostasis after large bore femoral venous sheath removal and results in faster hemostasis, early patient ambulation and less access site complications, without compromising vein patency compared with manual compression alone.

Study name or acronym; Author; Year published; PMID	Aim of study; Endpoints; Study type (RCT, observational — multicenter or single center; case series or other [if RCT include intervention and comparator]); Study size (N)	Patient population with inclusion and exclusion criteria	Results (absolute event rates, *P* values; OR or RR; 95% CI)	Other relevant findings or adverse events	Limitations; Other comments; Conclusions
Shaw et al. Use of suture-mediated vascular closure devices for the management of femoral vein access after transcatheter procedures.<br>• Year published: 2004<br>• PMID: 15558775	Aim: To report feasibility and experience with Perclose suture-mediated vascular closure device to achieve hemostasis and early mobility in patients with venous access.<br>Study type: Case series<br>Size: 42	Patients undergoing cardiac catheterization with femoral venous access who received Perclose suture-mediated closure device (6 Fr Closer or 8 Fr Prostar XL) for venous closure.<br>Contraindications: Groin hematoma during the procedure, difficult access due to extensive scar from surgery, or multiple prior vascular accesses.	There were 2 cases with failure requiring manual compression (1 with 14 Fr sheath, closing with 6 Fr Perclose); 3 patients had initial Perclose failure, requiring another Perclose deployment; 3 groin hematomas. Complications at access site in 2 patients (late staph infection requiring rehospitalization for intravenous antibiotics; DVT/PE).	Following the infection, prophylactic antibiotics policy (single dose cephazolin) was begun.	Perclose, Angioseal, or manual compression used to close arterial sites.
Coto. Closure of the femoral vein puncture site after transcatheter procedures using Angio-Seal.<br>• Year published: 2002<br>• PMID: 11793489	N=110	Inclusion criteria: Patients undergoing R/L heart catheterization or coronary intervention.	AngioSeal device deployed successfully in the vein in all patients (8 Fr) with immediate venous hemostasis; no late venous bleeding. Some 8% had bleeding due to ipsilateral arterial access.	A total of 93 (85%) patients received arterial Angio-Seal, 8 received Perclose, and 9 (8%) had manual pressure or a Fem-Stop applied to control arterial bleeding.	All had ipsilateral arterial access.

Study name or acronym; Author; Year published; PMID	Aim of study; Endpoints; Study type (RCT, observational — multicenter or single center; case series or other [if RCT include intervention and comparator]); Study size (N)	Patient population with inclusion and exclusion criteria	Results (absolute event rates, *P* values; OR or RR; 95% CI)	Other relevant findings or adverse events	Limitations; Other comments; Conclusions
Dou et al. Venous access site closures using the VASCADE vascular closure system.<br>• Year published: 2016<br>• PMID: 27886954	Aim: To describe use of the VASCADE vascular closure system to close venous access sites<br>Endpoints: Technical success (hemostasis after 3 minutes of manual compression following device deployment). Secondary: time to discharge and complications.<br>Study design: Single-center retrospective case series<br>Size: 32 venous access sites in 21 patients	Deep venous cases requiring ≥5 Fr sheath size. Used on 5–10 Fr sheaths.	Technical success rate 93.8%, hemostasis in 100% of inpatients 90% of outpatient cases; 2 sites failed: one required longer manual compression and the other manual compression and suturing.	Complications in 18.8% of device deployments: 5 minor (hematomas), including 1 with pain and 1 with increased thrombosis; and 1 major (cancer, chronic DVT with hypotension, inability to extubate after LE lysis and stent; stabilized after 2 days).	VASCADE device delivers a resorbable hemostatic collagen patch onto the extravascular surface of vessels, indicated for 5–7 Fr femoral arterial closures. All patients were anticoagulated with warfarin, enoxaparin, or heparin prior to the procedure; heparin during the procedure in 48%, thrombolytics in 48%. Postprocedure warfarin, heparin, enoxaparin, or apixaban in 92%.
Ben-Dor et al. MynxGrip vascular closure device versus manual compression for hemostasis of percutaneous transfemoral venous access closure: results from a	Aim: To evaluate safety of MynxGrip for common femoral vein closure<br>Endpoints: Safety: deep venous thrombotic and bleeding/vascular injury-related complications prior to discharge; device/procedure failure.	Inclusion criteria: Patients undergoing procedures requiring a 5-, 6-, or 7-Fr sheath in the common femoral vein.<br>Exclusion criteria: History of bleeding disorder; venous thrombosis or thromboembolism; pregnant or lactating;	No events for the primary composite safety endpoint of venous thrombosis; no major or minor vascular complications, access site infection, nerve injury, or access site bleeding requiring transfusion in either group. No device failure in the MynxGrip group. Time to hemostasis shorter in the MynxGrip group compared with manual compression: 0.12 ± 0.89 vs 7.6 ± 5.7 minutes; *P*<.001.		Followed to hospital discharge, with potential to miss late events. Largest sheath tested was 7 Fr; thus, safety and effectiveness of MynxGrip for large bore venous access not assessed.

Study name or acronym; Author; Year published; PMID	Aim of study; Endpoints; Study type (RCT, observational — multicenter or single center; case series or other [if RCT include intervention and comparator]); Study size (N)	Patient population with inclusion and exclusion criteria	Results (absolute event rates, *P* values; OR or RR; 95% CI)	Other relevant findings or adverse events	Limitations; Other comments; Conclusions
prospective multicenter randomized study.<br>• Year published: 2018<br>• PMID: 29656937	Efficacy: Time to hemostasis<br>Study type: Prospective multicenter randomized study<br>Size: N=208 (MynxGrip 104; Manual compression 104)	known severe allergy to contrast medium; allergy to PEG; known to require extended hospitalization or rehospitalization; scheduled to have CABG <30 days postprocedure; multiple (>1) attempts at venous access in the target vein; intraprocedural bleeding around the access site prior to sheath removal; critically ill, requiring IV vasopressors for BP stabilization; ipsilateral femoral artery puncture or sheath; GP IIb/IIIa use; any bleeding or vascular access site complication evident pre venous closure.			

Study name or acronym; Author; Year published; PMID	Aim of study; Endpoints; Study type (RCT, observational — multicenter or single center; case series or other [if RCT include intervention and comparator]); Study size (N)	Patient population with inclusion and exclusion criteria	Results (absolute event rates, *P* values; OR or RR; 95% CI)	Other relevant findings or adverse events	Limitations; Other comments; Conclusions
**Anticoagulation**					
Stevenson et al. Irrigated radiofrequency catheter ablation guided by electroanatomic mapping for recurrent ventricular tachycardia after myocardial infarction: the multicenter thermocool ventricular tachycardia ablation trial.<br>• Year published: 2008<br>• PMID: 19064682	Aim: To evaluate safety and efficacy of VT ablation with a saline-irrigated RF catheter combined with EAM.<br>Endpoints: No recurrence of SMVT at 6 months.<br>Study type: Observational multicenter<br>Size: 231	Inclusion criteria: SMVT requiring termination by cardioversion or AAD administration with 4 episodes in the previous 6 months despite an ICD or AAD therapy. Patients without ICDs were eligible after 2 episodes of SVT.<br>Exclusion criteria: Serum creatinine 2.5 mg/dL; LVEF 0.10; mobile LV thrombus on echocardiography; absence of vascular access to the LV; disease process likely to limit survival to 12 months; NYHA class IV HF; cardiac surgery within the past 2 months (unless VT was incessant; unstable angina; severe aortic stenosis or mitral regurgitation with a flail leaflet; pregnancy; and age <18 years.	No thromboembolic complications or stroke detected by neurological exam.<br>Vascular access complications (hematoma or pseudoaneurysms) 4.7%.		Anticoagulation recommended for 3 months after ablation with either 325 mg/d aspirin or warfarin, which was recommended if ablation had been performed over an area with >3 cm between ablation sites. The use of postprocedure bridging anticoagulation was not specified.

Study name or acronym; Author; Year published; PMID	Aim of study; Endpoints; Study type (RCT, observational — multicenter or single center; case series or other [if RCT include intervention and comparator]); Study size (N)	Patient population with inclusion and exclusion criteria	Results (absolute event rates, *P* values; OR or RR; 95% CI)	Other relevant findings or adverse events	Limitations; Other comments; Conclusions
Siontis et al. Thromboembolic prophylaxis protocol with warfarin after radiofrequency catheter ablation of infarct-related ventricular tachycardia.<br>• Year published: 2018<br>• PMID: 29315941	Aim: To assess the feasibility, safety, and effectiveness of a thromboembolic prophylaxis protocol in patients undergoing catheter ablation for infarct-related VT	Inclusion criteria: Patients undergoing ablation for infarct-related VT with open irrigated-tip catheters.<br>Exclusion criteria: LA thrombus, mobile, pedunculated LV thrombus.	In-hospital bleeding 6%, arterial thromboembolic event 0.6%. No definite or possible thromboembolic events in the first 3 months; 1 major bleeding event; no bleeding events in the antiplatelet group.		Anticoagulation for 3 months after extensive LV endocardial ablation (>3 cm between ablation lesions).

Study name or acronym; Author; Year published; PMID	Aim of study; Endpoints; Study type (RCT, observational — multicenter or single center; case series or other [if RCT include intervention and comparator]); Study size (N)	Patient population with inclusion and exclusion criteria	Results (absolute event rates, *P* values; OR or RR; 95% CI)	Other relevant findings or adverse events	Limitations; Other comments; Conclusions
Sawhney et al. Epicardial catheter ablation for ventricular tachycardia on uninterrupted warfarin: a safe approach for those with a strong indication for peri-procedural anticoagulation?<br>• Year published: 2016<br>• PMID: 27454616	Aim: To assess safety of obtaining epicardial access on uninterrupted warfarin<br>Study type: Prospective registry of epicardial VT ablation patients over 2 years<br>Size: 46	Inclusion criteria: VT catheter ablation; epicardial access attempted after heparinization with or without interrupted warfarin.<br>Exclusion criteria: Patients in whom epicardial access was attempted prior to heparinization.	There were no deaths, and no patients required surgery. A higher proportion of patients in the warfarin group had a drop-in hemoglobin of N2 g/dL compared with the no-warfarin group (38.5% vs 27.3%; *P*=.74) and delayed pericardial drain removal (7.8% vs 3.03%, *P*=.47). There was no difference in the overall procedural complication rate. No patients required warfarin reversal or blood transfusion.		Study suggests that epicardial access can be achieved without significant increased risk in patients on uninterrupted warfarin in addition to heparinization, though risk/benefit remains incompletely defined.

Study name or acronym; Author; Year published; PMID	Aim of study; Endpoints; Study type (RCT, observational — multicenter or single center; case series or other [if RCT include intervention and comparator]); Study size (N)	Patient population with inclusion and exclusion criteria	Results (absolute event rates, *P* values; OR or RR; 95% CI)	Other relevant findings or adverse events	Limitations; Other comments; Conclusions
Li et al. Prevention and treatment of lower limb deep vein thrombosis after radiofrequency catheter ablation: results of a prospective active controlled study.<br>• Year published: 2016<br>• PMID: 27329582	Aim: To determine the formation of LDVT post-RFCA and to evaluate the effect of rivaroxaban on LDVT formation<br>Endpoints: Composite of LDVT occurrence, change in diameter of femoral veins, and safety outcomes that were analyzed based on major or minor bleeding events. Blood flow velocity was also determined.<br>Study type: Prospective, single-center, active controlled study.	Inclusion criteria: Chinese patients with rapid VA who had received RFCA; 18 years or older; had no symptoms of PE; had not received a vitamin-K antagonist or any other anticoagulant; and had received no more than 36 hours of treatment with UFH or LMWH (3 doses 12 hours apart or 2 doses 24 hours apart).<br><br>Exclusion criteria: AF, thrombus, or bleeding diseases; coagulation disorders; hepatic and renal insufficiency; had undergone femoral central venous catheter operation for almost 1 month; had documented evidence of recent LDVT; reported cerebral ischemia, intracerebral	No complete occlusive thrombus or bleeding events were reported in either group. Lower incidence rate of nonoccluded thrombus in the rivaroxaban (5.8%) compared with the aspirin (16.7%) group.		Post 2–3 hours of the RFCA procedure, patients were assigned to the rivaroxaban group (10 mg/d for 14 days, n=86) or to the active control aspirin group (100 mg/d for 3 months, n=90). However, the method of allocation to rivaroxaban or aspirin was not specified. Also, rapid VA was stated as present; however, some ablations were for atrial arrhythmias.

Study name or acronym; Author; Year published; PMID	Aim of study; Endpoints; Study type (RCT, observational — multicenter or single center; case series or other [if RCT include intervention and comparator]); Study size (N)	Patient population with inclusion and exclusion criteria	Results (absolute event rates, *P* values; OR or RR; 95% CI)	Other relevant findings or adverse events	Limitations; Other comments; Conclusions
		bleeding, or gastrointestinal bleeding within the past 6 months; had undergone neurosurgery within the past 4 weeks or other surgery within the past 10 days; reported evidence of brain metastasis; cytotoxic chemotherapy; life expectancy <6 months; body weight <45 kg; severe HF (NYHA class III to IV); uncontrolled severe hypertension (>200/100 mm Hg); child-bearing potential without effective contraception; required thrombolytic therapy or treatment with antiplatelet agents; NSAIDs with a half-life of >17 hours; or potent CYP3A4 inhibitors, such as ketoconazole.			

Hemodynamic Deterioration and Proarrhythmia

Study name or acronym; Author; Year published; PMID	Aim of study; Endpoints; Study type (RCT, observational — multicenter or single center; case series or other [if RCT include intervention and comparator]); Study size (N)	Patient population with inclusion and exclusion criteria	Results (absolute event rates, *P* values; OR or RR; 95% CI)	Other relevant findings or adverse events	Limitations; Other comments; Conclusions
Santangeli et al. Acute hemodynamic decompensation during catheter ablation of scar-related ventricular tachycardia: incidence, predictors, and impact on mortality.<br>• Year published: 2015<br>• PMID: 25491601	Size: 193<br>Retrospective observational study	The study cohort consisted of consecutive patients who underwent RFCA of scar-related VT in the setting of ICM or NICM.	1. Periprocedural AHD occurred in 22 (11%) patients during 24 ablation procedures. In the majority of patients (63%), AHD did not occur during sustained VT activation mapping and was observed during substrate ablation.<br>2. Among procedural data, only use of general anesthesia was significantly associated with AHD (59% vs 29%; *P*=.004).<br>3. After a mean follow-up of 21 ± 7 months, 30 (16%) patients died. The mortality rate over follow-up was significantly higher in the AHD group compared with the rest of the population (50% vs 11%; log-rank *P*<.001).	1. AHD occurs in 11% of patients undergoing RFCA of scar-related VT and is associated with increased risk of mortality over follow-up.<br>2. AHD can be predicted by clinical factors, including advanced age, ICM, more severe HF status (NYHA class III/IV, lower EF), associated comorbidities (diabetes mellitus and chronic obstructive pulmonary disease), presentation with VT storm, and use of general anesthesia.	

Study name or acronym; Author; Year published; PMID	Aim of study; Endpoints; Study type (RCT, observational — multicenter or single center; case series or other [if RCT include intervention and comparator]); Study size (N)	Patient population with inclusion and exclusion criteria	Results (absolute event rates, *P* values; OR or RR; 95% CI)	Other relevant findings or adverse events	Limitations; Other comments; Conclusions
Khan et al. Effect of radiofrequency catheter ablation of ventricular tachycardia on left ventricular function in patients with prior myocardial infarction.<br>• Year published: 2002<br>• PMID: 12510135	Size: 62<br>Retrospective observational study	Sixty-two patients who underwent ablation for recurrent episodes of spontaneous SVT late after MI met the following inclusion criteria for this study: a preprocedural echocardiogram was performed between 1 and 7 days before ablation, a postablation echocardiogram was performed within 72 hours after ablation, and both were available.	Although the LVEF did not change for the group as a whole, 12 (19.4%) patients had an improvement in LVEF postablation of ≥5% (range 5.2%–12.0%) and 14 (22.5%) patients had a decrease in LVEF postablation of ≥5% (range −5.7% to −13%).	1. The potential for damage to contracting myocardium is an important concern in patients undergoing ablation of VT, most of whom have depressed ventricular function prior to ablation.<br>2. Despite the lack of change in EF for the entire group, 14 of 62 (22.5%) patients did have a decline in EF when a repeat echocardiogram was performed within 7 days of the ablation.	

Study name or acronym; Author; Year published; PMID	Aim of study; Endpoints; Study type (RCT, observational — multicenter or single center; case series or other [if RCT include intervention and comparator]); Study size (N)	Patient population with inclusion and exclusion criteria	Results (absolute event rates, *P* values; OR or RR; 95% CI)	Other relevant findings or adverse events	Limitations; Other comments; Conclusions
Santangeli et al. Early mortality after catheter ablation of ventricular tachycardia in patients with structural heart disease.<br>• Year published: 2017<br>• PMID: 28449770	Size: 2061<br>Retrospective study	The International VT Ablation Center Collaborative Group database includes data from a retrospective review of consecutive VT ablation procedures between 2002 and 2013 in patients with the following inclusion criteria: (1) SHD with ICM and/or NICM with LVEF <55% (LVEF >55% was included in cases of RV and hypertrophic cardiomyopathy); (2) RFCA for monomorphic VT; (3) evidence of myocardial scar, as identified with predefined criteria at electroanatomic voltage mapping (7–9); and (4) clinical follow-up for VT recurrence, transplant, and mortality.	1. A total of 100 patients (5%; 95% CI 4%–6%) died early after the procedure.<br>2. Of the 100 EM cases, 48 (48%) patients had early recurrent VT preceding death, although the time course from time of first VT recurrence to death was highly variable.<br>3. Refractory VT was the cause of death in 22% of cases.<br>4. Another 39% died of other cardiac causes (most commonly advanced HF).	1. In a contemporary cohort of patients with scar-related VT undergoing RFCA, EM occurred in 5% of cases.<br>2. Clinical and procedural variables indicating poorer clinical status (low LVEF, chronic kidney disease, VT storm, and unmappable VTs) and postprocedural VT recurrence may predict EM.	

**Study name or acronym; Author; Year published; PMID**	**Aim of study; Endpoints; Study type (RCT, observational — multicenter or single center; case series or other [if RCT include intervention and comparator]); Study size (N)**	**Patient population with inclusion and exclusion criteria**	**Results (absolute event rates, *P* values; OR or RR; 95% CI)**	**Other relevant findings or adverse events**	**Limitations; Other comments; Conclusions**
Turagam et al. Hemodynamic support in ventricular tachycardia ablation: an international VT ablation center collaborative group study.<br>• Year published: 2017<br>• PMID: 29759835	Size: 1655<br>Multicenter, retrospective study	Patients from the shared database of the IVTCC group from 12 specialized arrhythmia management centers worldwide, who underwent VT ablation between 2002 and 2015, were included. VT ablation was performed in 105 patients receiving HS devices.	1. A total of 1655 patients were referred for catheter ablation for VT. Of these, 105 (6.3%) patients underwent VT ablation while receiving HS.<br>2. The mean LVEF was significantly lower (25.4 ± 11.9% vs 35.6 ± 12.9%; *P*<.001) and there were more patients with NYHA functional class III (53.8% vs 30.0%; *P*<.001) and NYHA functional class IV (22.1% vs 3.9%; *P*<.001) symptoms in the HS group versus the no-HS group.<br>3. One-year mortality was significantly greater (34.7% vs 9.3%) in the HS group compared with the no-HS group.	1. Patients requiring HS had more advanced disease with multiple comorbidities and, as expected, had a significantly higher 1-year mortality than did those in the no-HS group.<br>2. In patients with LVEF ≤20% and NYHA functional class III to IV, there was also no significant difference in clinical outcomes compared with no HS.	

Study name or acronym; Author; Year published; PMID	Aim of study; Endpoints; Study type (RCT, observational — multicenter or single center; case series or other [if RCT include intervention and comparator]); Study size (N)	Patient population with inclusion and exclusion criteria	Results (absolute event rates, *P* values; OR or RR; 95% CI)	Other relevant findings or adverse events	Limitations; Other comments; Conclusions
Yokokawa et al. Reasons for recurrent ventricular tachycardia after catheter ablation of post-infarction ventricular tachycardia.<br>• Year published: 2013<br>• PMID: 23122796	Size: 98<br>Single center, retrospective study	Ninety-eight patients with postinfarction VT and an implanted ICD were referred for catheter ablation of VT. All patients had a history of MI.	A total of 725 VTs were induced during the ablation procedure. All VTs were targeted. In 76 patients, 105 clinical VTs were inducible.<br>Critical sites were identified with entrainment mapping and pace mapping (10 of 12 matching leads) for 75 of 105 (71%) clinical VTs and for 278 of 620 (45%) nonclinical VTs.<br>Postablation, the clinical VT was not inducible in any patient, and all VTs were rendered noninducible in 63% of the patients. Over a mean follow-up period of 3 5± 23 months, 65 of 98 (66%) patients had no recurrent VTs and 33 (34%) had VT recurrence.	A new VT occurred in 26 of 33 (79%) patients, and a prior clinical VT recurred in 7 (21%) patients. Patients with recurrent VT had a larger scar area as assessed by EAM compared with patients without recurrent VTs (93 ± 40 cm^2 vs 69 ± 30 cm^2; *P*=.002). In patients with repeat procedures, the majority of inducible VTs for which a critical area could be identified were at a distance of 6 ± 3 mm from the prior ablation lesions.	Left-sided programmed stimulation was not performed in all patients in whom the clinical VT could not be induced. In addition, programmed stimulation with isoproterenol was not performed in all patients due to incomplete revascularization. This might have reduced the rate of noninducible patients and might have resulted in a lower recurrence rate. Because only a near- or a far-field ICD electrogram of the clinical VT was available in one-third of patients for the initial procedure, a few clinical VTs might not have been correctly identified.

Follow-up of Patients Post Catheter Ablation of Ventricular Tachycardia

Study name or acronym; Author; Year published; PMID	Aim of study; Endpoints; Study type (RCT, observational — multicenter or single center; case series or other [if RCT include intervention and comparator]); Study size (N)	Patient population with inclusion and exclusion criteria	Results (absolute event rates, *P* values; OR or RR; 95% CI)	Other relevant findings or adverse events	Limitations; Other comments; Conclusions
Oloriz et al. Defining the outcome of ventricular tachycardia ablation: timing and value of programmed ventricular stimulation.<br>• Year published: 2018<br>• PMID: 29545359	Study design: This was an observational study performed at a single center<br>Aim of study: To describe experience in the systematic use of NIPS to assess procedural outcomes and to inform ICD programming.<br>Endpoints: Recurrent VT<br>Sample size: 286 patients	Consecutive patients with SHD undergoing VT ablation	Concordance between PVS at the end of the procedure and PVS at day 6 after the ablation was 67%. PPV and NPV were higher for PVS day 6 (53% and 88% vs 43% and 71%). Patients with ischemia and preserved EF had the highest NPV (91% and 96%). Among 46 of 174 (26%) noninducible patients on PVS at the end of the procedure, but inducible at day 6, 59% had VT recurrence at 1-year follow-up; recurrences were 9% when both studies were negative. There were no inappropriate shocks; incidence of syncope was 3% with no harm. The rate of appropriate shocks per patient per month according to NIPS results was significantly reduced, comparing the month before and after ablation (class A: 2 [0.75–4] versus 0; class B: 2 [1–4] versus 0; class C: 2 [1–4] versus 0; *P*<.001).		Limitations: Retrospective, single-center; NIPS was not performed in all patients, limiting its applicability to a selected cohort of patients after VT ablation. The results of the study might not be applicable to patients with advanced functional class or hemodynamic deterioration, given PVS at day 6 was performed in only half of them. ICD programming was empirical and not compared with a standard ICD programming. Several ICD manufacturers were used in this study, leading to a heterogeneous ICD programming.<br><br>Conclusions: NIPS can be considered in the days after VT ablation to further define the risk of VT recurrence. A quarter of patients noninducible at the end of ablation have inducible VT just a few days after the procedure, with more than half of them recurring at 1-year follow-up. In case of an inducible NIPS study, AAD therapy fails to prevent VT recurrence; redo ablation should be an option in selected patients.

Study name or acronym; Author; Year published; PMID	Aim of study; Endpoints; Study type (RCT, observational — multicenter or single center; case series or other [if RCT include intervention and comparator]); Study size (N)	Patient population with inclusion and exclusion criteria	Results (absolute event rates, *P* values; OR or RR; 95% CI)	Other relevant findings or adverse events	Limitations; Other comments; Conclusions
Frankel et al. Noninvasive programmed ventricular stimulation early after ventricular tachycardia ablation to predict risk of late recurrence.<br>• Year published: 2012<br>• PMID: 22516442	Study design: Observational single center<br>Aim of study: To evaluate the ability of NIPS after VT ablation to identify patients at high risk of VT recurrence.<br>Endpoints: VT recurrence<br>Sample size: 200 patients	Consecutive patients with SHD undergoing VT ablation	Some 59 (44.7%) patients had no inducible VT at NIPS; 49 (37.1%) had inducible nonclinical VT only; and 24 (18.2%) had inducible clinical VT. Patients with inducible clinical VT at NIPS had markedly poorer 1-year VT-free survival compared with those in whom no VT was inducible (30% vs 80%; *P*=.001), including 33% recurring with VT storm. Patients with inducible nonclinical VT only had intermediate 1-year VT-free survival (65%).		Limitations: Observational and single center study. Not all patients underwent NIPS, and differences exist between those who did and did not undergo NIPS. The distinction between clinical and nonclinical VT can be problematic, given some patients do not have 12-lead ECGs of spontaneous VT, and nonclinical VTs can subsequently occur spontaneously. Study looked at inducibility of VT at NIPS at one point in time (ie, before discharge from the hospital). It would be interesting to study inducibility over time, as VT substrate and antiarrhythmic milieu continue to evolve. The multivariable analysis was overfitted.<br><br>Conclusions: When patients with VT and SHD have no inducible VT or inducible nonclinical VT only at the end of ablation or are too unstable to undergo final programmed stimulation, NIPS should be considered in the following days in the absence of spontaneous VT to further define the risk of arrhythmia recurrence. If clinical VT is inducible at NIPS, repeat ablation might be indicated because recurrence over the following year is high, including ICD shocks and VT storm.

AAA, antiarrhythmic agent; AAD, antiarrhythmic drug; ACHD, adult congenital heart disease; ACT, activated clotting time; AF, atrial fibrillation; ALPAP, anterolateral papillary muscle; AMC, aortomitral continuity; APM, anterior papillary muscle; ARR, absolute risk reduction; ASC, aortic sinus cusp; ASOV, aortic sinus of Valsalva; ATP, antitachycardia pacing; AUC, area under the curve; AV, atrioventricular; AVA, atrioventricular annulus; AVB, atrioventricular block; BB, beta blocker; BBB, bundle branch block; BBR, bundle branch reentry; BBRVT, bundle branch reentrant ventricular tachycardia; BMI, body mass index; BP, blood pressure; BrS, Brugada syndrome; BSM, body surface mapping; BZ, border zone; CABG, coronary artery bypass graft; CAD, coronary artery disease; CAG, coronary angiography; CAVEs, combined adverse vascular events; CCB, calcium channel blocker; CEVTSA, complete endocardial ventricular tachycardia substrate ablation; CF, contact force; CFA, common femoral artery; CHD, congenital heart disease; CHF, congestive heart failure; CI, confidence interval; CMR, cardiac magnetic resonance imaging; CP, chest pain; CRT, cardiac resynchronization therapy; CSAE, cardiovascular specific adverse events; CVS, coronary venous system; DAPT, dual antiplatelet therapy; DCE, delayed contrast enhancement; DCM, dilated cardiomyopathy; DEEP, decrement-evoked potential; DVT, deep vein thrombosis; DWI-MRI, diffusion-weighted magnetic resonance imaging; EAM, electroanatomic mapping; ECG, electrocardiogram; EF, ejection fraction; E-IDC, electrogram displaying isolated, delayed component; EPS, electrophysiology study; ES, electrical storm; EUS, electrically unexcitable scar; EVM, electroanatomical voltage mapping; FVT, fascicular ventricular tachycardia; GA, general anesthesia; GCV, great cardiac vein; HB, His bundle; HCM, hypertrophic cardiomyopathy; HF, heart failure; HFH, heart failure hospitalization; HPS, His-Purkinje system; HR, hazard ratio; HS, hemodynamic support; IABP, intra-aortic balloon pump; ICD, implantable cardioverter defibrillator; ICE, intracardiac echocardiography; ICM, ischemic cardiomyopathy; IDC, isolated delayed component; IDCM, idiopathic dilated cardiomyopathy; IFLVT, idiopathic fascicular left ventricular tachycardia; IHD, ischemic heart disease; ILVT, idiopathic left ventricular tachycardia; IVA, idiopathic ventricular arrhythmia; IVCD, intraventricular conduction delay; IVCS, intravenous conscious sedation; IVT, idiopathic ventricular tachycardia; IVT-LAF, idiopathic ventricular tachycardia of left anterior fascicular origin; LA, left atrial; LAE, life-threatening arrhythmic events; LAVA, local abnormal ventricular activity; LBBB, left bundle branch block; LGE, late gadolinium enhancement; LMCA, left main coronary artery; LMWH, low-molecular-weight heparin; LOS, length of stay; LP, late potential; LPF, left posterior fascicle; LPFVT, left posterior fascicular ventricular tachycardia; LUSVT, left upper septal fascicular ventricular tachycardia; LV, left ventricle; LVA, low-voltage area; LVEF, left ventricular ejection fraction; LVCM, left ventricular cardiomyopathy; LVEDD, left ventricular end diastolic diameter; LVESD, left ventricular end systolic diameter; LVH, left ventricular hypertrophy; MACE, major adverse cardiac events; MAP, mean arterial pressure; MC, manual compression; MCC, manual catheter control; MEM, multielectrode mapping; MEMC, multielectrode mapping catheter; MI, myocardial infarction; MMVT, monomorphic ventricular tachycardia; MNS, magnetic navigation system; MRI, magnetic resonance imaging; MVT, monomorphic ventricular tachycardia; NCC, noncoronary cusp; NICM, nonischemic cardiomyopathy; NIDCM, nonischemic dilated cardiomyopathy; NIPS, noninvasive programmed stimulation; NOAC, non-vitamin K antagonist oral anticoagulant; NPV, negative predictive value; NSAID, nonsteroidal anti-inflammatory drug; NSVT, nonsustained ventricular tachycardia; NYHA, New York Heart Association; OR, odds ratio; PAP, papillary muscle; PCI, percutaneous coronary intervention; PE, pulmonary embolism; PET, positron emission tomography; PM, pace mapping; PMVT, polymorphic ventricular tachycardia; PP, Purkinje potential; PPM, point-by-point mapping; PPV, positive predictive value; PSC, pulmonary sinus cusp; PSS, purse-string suture; PV, pulmonary valve; PVAD, percutaneous ventricular assist device; PVAI, pulmonary vein antrum isolation; PVC, premature ventricular complex; PVI, pulmonary vein isolation; PVR, pulmonary valve replacement; PVS, programed ventricular stimulation; PV, pulmonary valve; QOL, quality of life; RA, right atrial; RBBB, right bundle branch block; RCC, right coronary cusp; RCT, randomized controlled trial; RF, radiofrequency; RFCA, radiofrequency catheter ablation; RMN, remote magnetic navigation; RMVT, repetitive monomorphic ventricular tachycardia; ROC, receiver operating characteristic; RR, relative risk; RV, right ventricle; RVEDV, right ventricular end diastolic volume; RVI, right ventricular inflow; RVOT, right ventricular outflow tract; SBP, systolic blood pressure; SCD, sudden cardiac death; SHD, structural heart disease; SMVT, sustained monomorphic ventricular tachycardia; SOO, site of origin; SR, sinus rhythm; SVT, sustained ventricular tachycardia; TEE, transesophageal echocardiography; TGA, transposition of the great arteries; TIA, transient ischemic attack; ToF, tetralogy of Fallot; TTE, transthoracic echocardiogram; TVA, tricuspid valve annulus; US, ultrasound; VA, ventricular arrhythmia; VCD, vascular closure device; VF, ventricular fibrillation; VOTA, ventricular outflow tract arrhythmia; VPC, ventricular premature complex; VPD, ventricular premature depolarization; VSD ventricular septal defect; VT, ventricular tachycardia; WCT, wide complex tachycardia; WT, wall thinning.
